# Copper‐Catalyzed Borylation of Acyl Chlorides with an Alkoxy Diboron Reagent: A Facile Route to Acylboron Compounds

**DOI:** 10.1002/chem.202201329

**Published:** 2022-06-13

**Authors:** Xiaolei Zhang, Alexandra Friedrich, Todd B. Marder

**Affiliations:** ^1^ Institut für Anorganische Chemie and Institute for Sustainable Chemistry & Catalysis with Boron Julius-Maximilians-Universität Würzburg Am Hubland 97074 Würzburg Germany

**Keywords:** boronate, borylation, carbonyl, catalysis, copper

## Abstract

Herein, the copper‐catalyzed borylation of readily available acyl chlorides with bis(pinacolato)diboron, (B_2_pin_2_) or bis(neopentane glycolato)diboron (B_2_neop_2_) is reported, which provides stable potassium acyltrifluoroborates (KATs) in good yields from the acylboronate esters. A variety of functional groups are tolerated under the mild reaction conditions (room temperature) and substrates containing different carbon‐skeletons, such as aryl, heteroaryl and primary, secondary, tertiary alkyl are applicable. Acyl *N*‐methyliminodiacetic acid (MIDA) boronates can also been accessed by modification of the workup procedures. This process is scalable and also amenable to the late‐stage conversion of carboxylic acid‐containing drugs into their acylboron analogues, which have been challenging to prepare previously. A catalytic mechanism is proposed based on in situ monitoring of the reaction between *p*‐toluoyl chloride and an NHC‐copper(I) boryl complex as well as the isolation of an unusual lithium acylBpinOBpin compound as a key intermediate.

## Graphical abstract

**Copper‐catalyzed borylation of acyl chlorides** with B_2_pin_2_ in the presence of LiO*t*‐Bu at room temperature provides a convenient synthetic approach to various acylboron compounds with good functional group tolerance. The synthetic utility is demonstrated by the late‐stage derivatization of drug molecules and the ease of scale‐up. A catalytic mechanism is proposed based on in situ reaction monitoring and the structural characterization of a key acylboronate intermediate and a novel Cu complex byproduct.

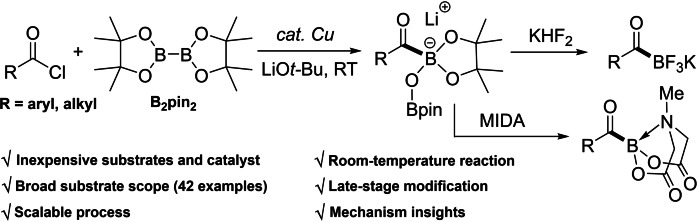

## Introduction

**Scheme 1:**
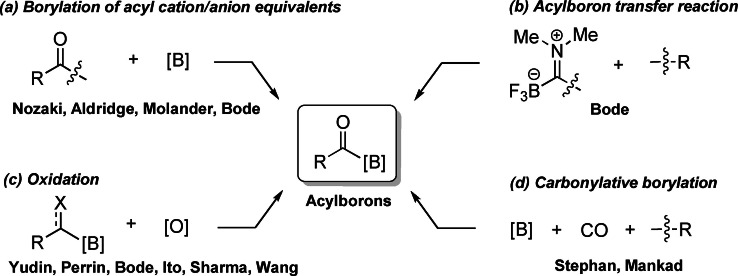
General illustration for the synthesis of acylboron compounds.

Acylboron compounds represent a class of organoboranes^[1]^ which has attracted increasing interest during the last decade. In early studies, they were proposed as intermediates in some chemical transformations (e. g., carbonylation of organoboranes)^[2]^ and were first isolated by Yamashita, Nozaki and co‐workers in 2007.^[3]^ Acylboron compounds,^[4]^ especially the stable and readily isolable potassium acyltrifluoroborates (KATs) and acyl *N*‐methyliminodiacetic acid (MIDA) boronates, have become increasingly useful compounds in organic synthesis^[5]^ and bio‐synthesis, being particularly prominent as coupling partners for amide‐bond‐forming reactions to functionalize proteins and peptides.^[6]^ To this end, several strategies for the synthesis of acylboron compounds have been reported (Scheme 1). Initially, Nozaki reported the synthesis of diamino‐stabilized acylboron compounds by reacting boryllithium or borylmagnesium nucleophiles with benzoyl chloride or benzaldehyde.^[3]^ Later, Molander and Bode independently reported the generation of acyl anion equivalents from the lithiation of enol ethers or *N*,*O*‐acetals followed by trapping with electrophilic B(OR)_3_ reagents to produce KATs.^[7]^ Acylboron transfer reagents have been developed by Bode to incorporate a stable acylboryl moiety into desired organic functionalities through either stoichiometric or catalytic transformations (Scheme 1b).^[8]^ Furthermore, several groups including Yudin,^[9]^ Perrin,^[10]^ Ito,^[11]^ Sharma,^[12]^ and Wang^[13]^ have utilized oxidation strategies as key steps to access KATs or acyl MIDA boronates from α‐hydroxy alkylboronates, alkenyl boronates, or their precursors (Scheme 1c). Carbonylative borylation reactions have also been reported to provide acylboron species under CO pressure (Scheme 1d).[^2d^, ^14^] In spite of these advances, improved routes are highly desirable as the established methods often require expensive or toxic starting materials, harsh reaction conditions or multi‐step synthesis.

Copper(I) boryl complexes are of wide interest as key intermediate to target unsaturated carbon−carbon multiple bonds, C=X (X=N, O) bonds and carbon−halide bonds.^[15]^ Catalytic processes mediated by copper boryl species feature the low cost of the metal catalyst, and often mild reaction conditions and good functional group tolerance. Following the first report of the Pt‐catalyzed 1,4‐diboration of α,β‐unsaturated carbonyl compounds by Marder and Norman,^[16]^ the groups of Hosomi^[17a]^ and Miyaura[^17b^, ^17c^] both reported examples of the Cu‐promoted reaction which, in the case of Cu, involves a copper boryl species in which the boryl group is nucleophilic.^[15a]^

**Scheme 2:**
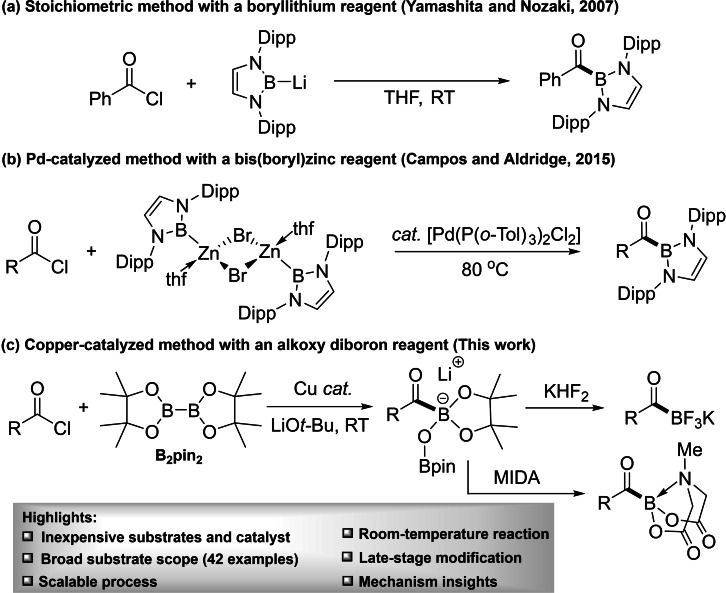
Synthesis of acylboron compounds from acyl chlorides.

The first well‐defined copper(I) boryl complex [(IDipp)CuBpin] [IDipp=1,3‐bis‐(2,6‐diisopropylphenyl)imidazole‐2‐ylidene, pin=pinacolato=2,3‐dimethyl‐2,3‐butane‐diolate] was isolated and characterized by the Sadighi group in 2005,^[18a]^ and their structures and stabilities were subsequently studied in detail by Kleebgerg et al.[^18b^, ^18c^, ^18d^] Their reactivities toward carbonyl compounds (CO_2_,[^18a^, ^19^] aldehydes,^[20]^ ketones,^[21]^ imines^[22]^) were explored experimentally and theoretically by several groups including Sadighi, Ito, Liu, Hou, and Lin and Marder. The insertion of a C=X (X=N, O) unit into the Cu−B bond initially gave a Cu−X−C−B linkage and finally led to reduction of CO_2_ to CO or diboration of these organic carbonyls. Interestingly, in the absence of added B_2_pin_2_, stoichiometric reaction of an NHC−Cu‐boryl complex with aldehydes led to the isolation of a species containing a Cu−C−O−B linkage. However, it was shown, both experimentally[^20a^, ^22c^] and theoretically^[20b]^ that the nucleophilicity of the boryl moiety led kinetically to the Cu−O−C−B species which can either react with B_2_pin_2_ (if present) to give the diboration product, or in the absence of B_2_pin_2_, rearrange rapidly to the more thermodynamically stable Cu−C−O−B isomer. Furthermore, organohalides (chlorides, bromides, iodides) have also been shown to react with copper boryl complexes,^[23]^ allowing the development of copper‐catalyzed methods for the generation of alkyl‐ and aryl‐boronic esters from the respective halides. Distinct from these substrates containing a single functionality, acyl chlorides possess dual functionality with both a C−Cl bond and a C=O bond, and the reactivity of copper(I) boryl complexes with acyl chlorides has not been reported. Based on the above knowledge, we envisaged that the controlled reaction of LCu−Bpin with acyl chlorides would generate acylBpin compounds. However, such a process requires two issues to be addressed: i) boryl addition to the C=O bond as a side reaction; and ii) the low stability of acylBpin compounds. However, as acyl chlorides are inexpensive substrates which can be conveniently generated from naturally abundant carboxylic acids, their transformation to acylboron compounds would significantly reduce the costs and expand the diversity of these unique carbonyl compounds. Herein, we report the room temperature, Cu‐catalyzed borylation of acyl chlorides containing aryl, heteroaryl, alkyl‐substituents and drug‐like moieties with B_2_pin_2_ to produce acylboron compounds (Scheme 2c) which can subsequently provide either KATs or acyl MIDA boronates in good yields. Only two examples have been reported for the synthesis of acylboron compounds from acyl chlorides. In 2007, Yamashita, Nozaki and co‐workers reported the stoichiometric reaction of a boryllithium nucleophile with benzoyl chloride to produce cyclic diamino‐stabilized acylboron compounds (Scheme 2a).^[3b]^ In 2015, Campos and Aldridge reported a palladium‐catalyzed Negishi‐type coupling of an acyl chloride with a bis(boryl) zinc reagent, again producing cyclic diamino‐stabilized acylboron species (Scheme 2b).^[24]^ However, the multiple‐step synthesis of the boryllithium or borylzinc reagents restricted their use. In contrast, our method employs easy to handle B_2_pin_2_, which is commercially available in bulk quantities at low cost. During the preparation of our manuscript, Bode and Mankad reported the synthesis of acylboron compounds from mixed anhydrides,^[25]^ representing a significant advance. However, their method is not compatible with acyl chlorides and requires high catalyst loading. Our approach utilizes acyl chlorides as substrates which are generally more reactive than anhydrides, thus leading to high catalytic efficiency and good chemoselectivity including substrates containing aryl halide moieties, and other carbonyl functionalities. Our mechanistic studies also provide insight into the reactivity of acyl chlorides with copper(I) boryl complexes; a new type of Bpin‐derived acylboronate ester (lithium acylBpinOBpin) has been isolated which further elucidates the catalytic mechanism.

## Results and Discussion

We initially investigated the reaction of *p*‐toluoyl chloride **1 a** with B_2_pin_2_ using a catalytic amount (5 mol %) of (NHC)CuCl complexes (Table 1) and the product was converted to the air‐stable KAT **2 a** after work‐up with aqueous KHF_2_ for facile isolation and storage. As (NHC)CuBpin can be generated by reaction of (NHC)CuO*t*‐Bu with B_2_pin_2_,^[16]^ we attempted to complete the catalytic cycle by adding a stoichiometric amount of an alkali metal *tert*‐butoxide with slow addition of 1**a** in solution. Using 1.0 equiv. of LiO*t*‐Bu led to **2 a** in moderate yield (37 %, Table 1, entry 1), whereas NaO*t*‐Bu failed to afford any product (entry 2). Other alkali metal alkoxides such as KO*t*‐Bu, LiOMe, KOMe also gave the product under our conditions, albeit with decreased yields (8 % to 15 %, entries 3–5). No product was observed under base‐free or catalyst‐free conditions (entries 6–7). Clearly, the cation of the base plays an important role in the process. Increasing the loading of LiO*t*‐Bu significantly improved the yield, leading to the isolation of KAT **2 a** in 65 % yield with 1.5 equiv. of LiO*t*‐Bu and up to 82 % yield with 2.0 equiv. (entries 8–9). Solvent screening (entry 10‐13) indicated that toluene was optimal. While the utilization of (IDipp)CuCl (5 mol %) afforded **2 a** in only 28 % yield, (ICy)CuCl, bearing 1–3‐dicyclohexylimidazol‐2‐ylidene, proved most effective (entries 14–16). Elevating the temperature to 60 °C or changing the alkoxy diboron reagent to bis(neopentylglycolato)diboron (B_2_neop_2_) gave the product, but in somewhat decreased yields (55 % to 61 % yields, entries 17–18) and the reaction was not tolerant to air (entry 19).

**Table 1:** Reaction optimization.^[a]^

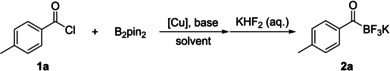				
Entry	[Cu] (mol %)	Base (equiv.)	solvent	Isolated yield of **2 a** ^[b]^
1	[(ICy)CuCl] (5)	LiO*t*‐Bu (1.0)	toluene	37
2	[(ICy)CuCl] (5)	NaO*t*‐Bu (1.0)	toluene	n.d.^[c]^
3	[(ICy)CuCl] (5)	KO*t*‐Bu (1.0)	toluene	15
4	[(ICy)CuCl] (5)	LiOMe (1.0)	toluene	11
5	[(ICy)CuCl] (5)	KOMe (1.0)	toluene	8
6	[(ICy)CuCl] (5)	none	toluene	n.d.
7	none	LiO*t*‐Bu (1.0)	toluene	n.d.
8	[(ICy)CuCl] (5)	LiO*t*‐Bu (1.5)	toluene	65
9	[(ICy)CuCl] (5)	LiO*t*‐Bu (2.0)	toluene	82
10	[(ICy)CuCl] (5)	LiO*t*‐Bu (2.0)	Et_2_O	64
11	[(ICy)CuCl] (5)	LiO*t*‐Bu (2.0)	THF	44
12	[(ICy)CuCl] (5)	LiO*t*‐Bu (2.0)	dioxane	52
13	[(ICy)CuCl] (5)	LiO*t*‐Bu (2.0)	MeCN	15
14	[(IDipp)CuCl] (5)	LiO*t*‐Bu (2.0)	toluene	28
15	[(IMes)CuCl] (5)	LiO*t*‐Bu (2.0)	toluene	35
16	[(I*i*‐Pr)CuCl] (5)	LiO*t*‐Bu (2.0)	toluene	75
17^[d]^	[(ICy)CuCl] (5)	LiO*t*‐Bu (2.0)	toluene	52
18^[e]^	[(ICy)CuCl] (5)	LiO*t*‐Bu (2.0)	toluene	61
19^[f]^	[(ICy)CuCl] (5)	LiO*t*‐Bu (2.0)	toluene	n.d.

[a] Reaction conditions, unless otherwise stated: *p*‐toluoyl chloride **1 a** (0.33 mmol, 1.1 equiv.), B_2_pin_2_ (1.0 equiv.), solvent (4 mL), room temperature, 6 h, argon atmosphere; a solution of **1 a** in the corresponding solvent (2 mL) was added dropwise. Workup: KHF_2_ (9.0 equiv., 2.7 M in H_2_O, 1.0 mL), THF (4 mL), 24 h. [b] Isolated yield. [c] Product not detected. [d] Heating at 60 °C. [e] Using B_2_neop_2_ as the boron source. [f] Reaction conducted in air. ICy: 1,3‐dicyclohexylimidazol‐2‐ylidene. IDipp: 1,3‐bis‐(2,6‐diisopropylphenyl)imidazole‐2‐ylidene. IMes: 1,3‐dimesitylimidazole‐2‐ylidene. I*i*‐Pr: 1,3‐diisopropylimidazole‐2‐ylidene.

**Scheme 3:**
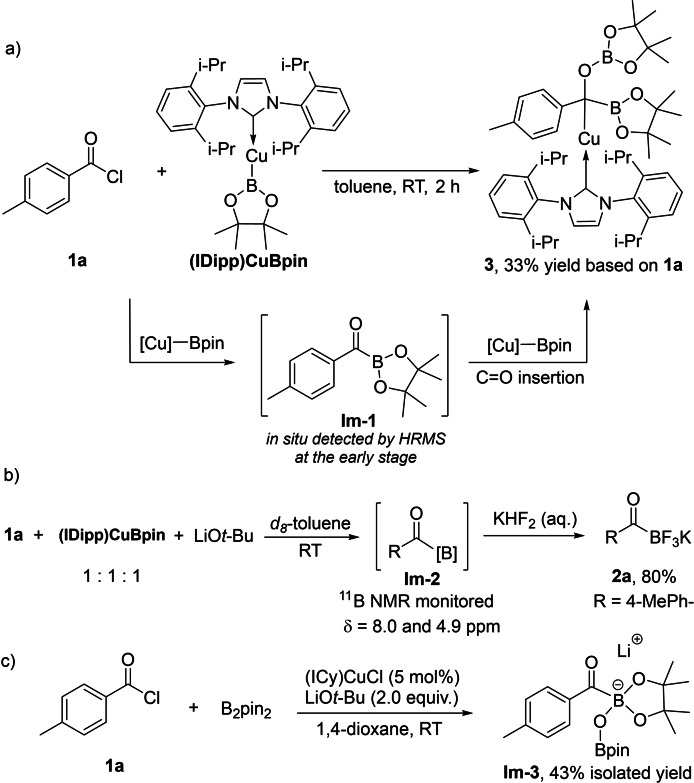
Reactivity of **1 a** with the copper(I) boryl complex [(IDipp)CuBpin] without and with LiO*t*‐Bu and synthesis of a lithium acylboronate ester.

**Figure 1:**
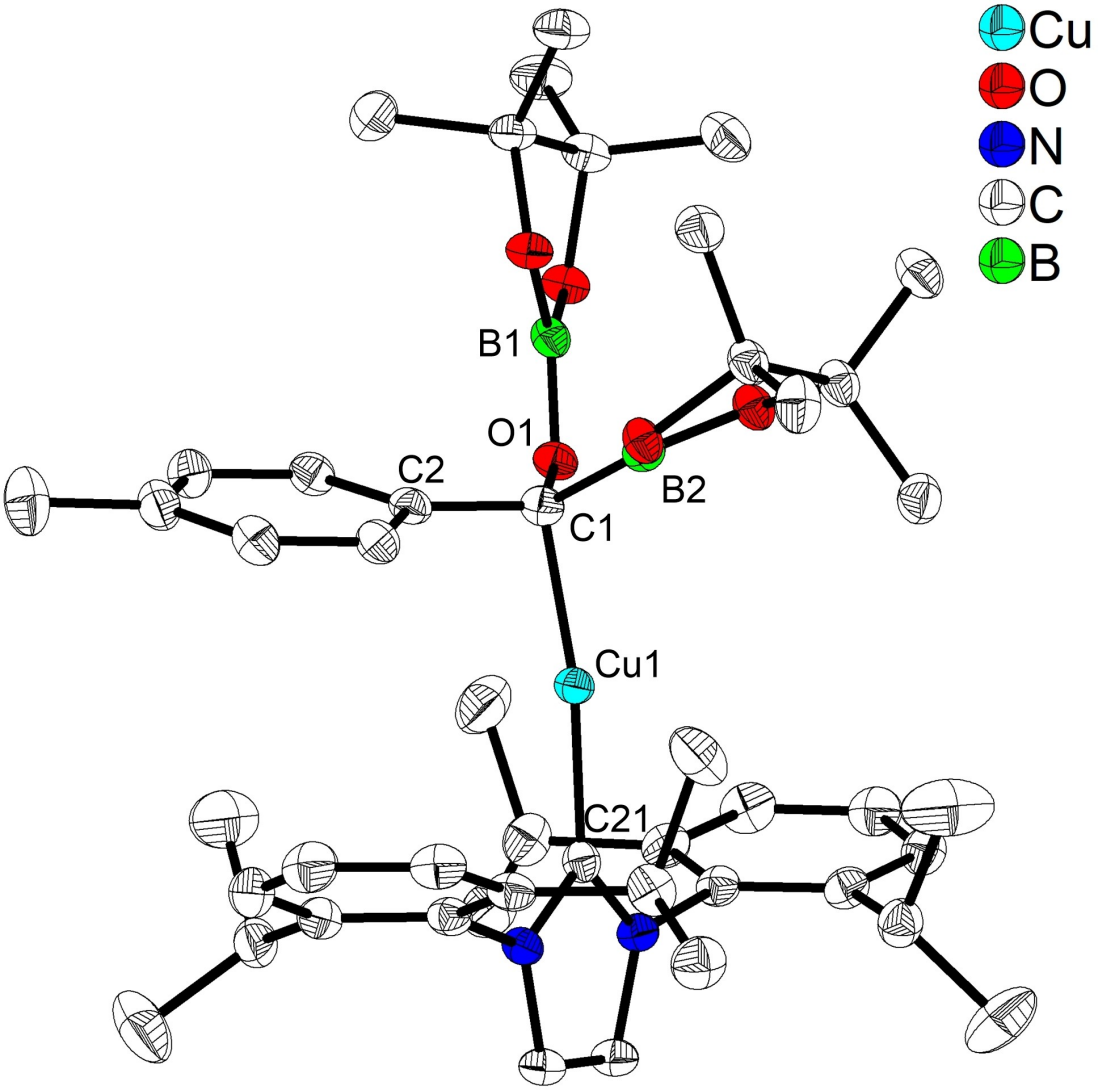
Molecular structure of **3**. (Ellipsoids drawn at 50 % probability and H atoms omitted for clarity). Selected bond distances [Å] and angles [deg]: Cu1−C1 1.9723(2), Cu1−C21 1.8987(2), C1−O1 1.4750(2), C1−B2 1.534(2), B1−O1 1.344(2), C1−C2 1.485(2), C1−Cu1−C21 171.76(6), Cu1−C1−C2 99.72(9), Cu1−C1−B2 104.46(1), Cu1−C1−O1 107.78(9), C2−C1−O1 111.55(1), C2−C1−B2 121.06(1), O1−C1−B2 110.64(1).

To gain insight into the reaction mechanism, stoichiometric reactions and control experiments were carried out (Scheme 3). Reaction of **1 a** with 1.0 equiv. of (IDipp)CuBpin in toluene occurred readily at room temperature, leading to the isolation of Cu complex **3** in 33 % yield based on **1 a** (Scheme 3a). The crystal structure of **3** confirmed the formation of an sp^3^C(1) center along with C−B, Cu−C and B−O bonds (Figure 1). The isolation of **3** suggests the initial formation of the desired acylBpin intermediate which subsequently undergoes C=O insertion into a second equivalent of the copper(I) boryl complex as a side reaction. Thus, slow addition of a toluene solution of (IDipp)CuBpin to an excess of **1 a** (5.0 equiv.) at room temperature allowed the detection of a 3‐coordinate acylBpin **Im‐1** (Scheme 3) at the early stage of the reaction (<10 min) by high resolution mass spectrometry (HRMS) analysis (Supporting Information‐Figure S1), accompanied by the precipitation of (IDipp)CuCl. Isolation of **Im‐1** was unsuccessful due to its high reactivity. Our results are consistent with previous studies on the reactivity of (IDipp)CuBpin with aldehydes and imines.[^20a^, ^20b^, ^22c^] The carbonyl insertion into Cu−B most likely generates a Cu−O−C−B complex kinetically, which isomerizes to the thermodynamically preferred Cu−C−O−B isomer, as shown in **3**, in the absence of a diboron reagent (e. g. B_2_pin_2_).

**Figure 2:**
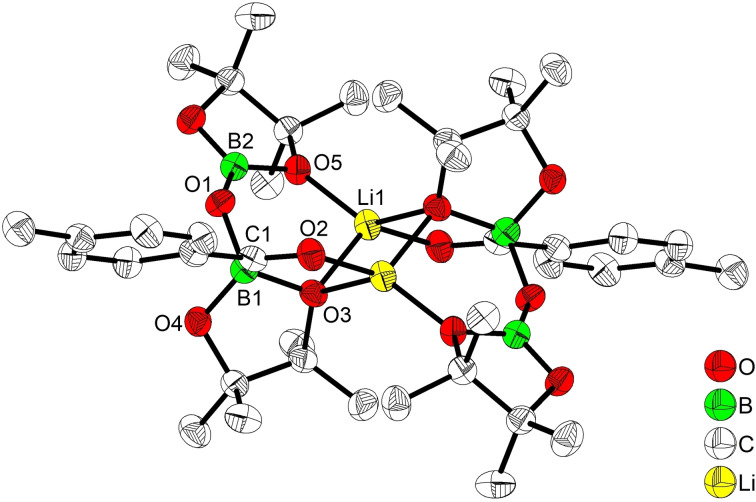
Molecular structure of the dimer of **Im‐3**. (Ellipsoids drawn at 50 % probability and H atoms omitted for clarity). Selected bond distances [Å] and angles [deg]: C1−B1 1.664(3), C1−O2 1.254(2), B1−O1 1.481(3), B2−O1 1.326(5), Li1−O3 1.994(4), Li1−O5 1.916(6), Li1−O2 1.907(4), B1−C1−O2 119.46(2), B1−O1−B2 134.0(3).

In order to prevent the side reaction of the product and to produce the desired acylboronate, a 3‐component reaction of **1 a** with (IDipp)CuBpin in the presence of LiO*t*‐Bu was monitored in *d_8_*‐toluene, and we found no evidence for the formation of **3**. Instead, two new ^11^B NMR resonances at 4.9 and 8.0 ppm in the chemical shift range of 4‐coordinate boron were observed (Supporting Information‐Figure S2). Isolation of the compound **Im‐2** from 1,4‐dioxane/*n*‐hexane also shows a 4‐coordinate ^11^B NMR signal at 3.6 ppm in *d_8_*‐THF. The product turned out to be a mixture instead of a single compound (Supporting Information‐Figure S3), and this mixture was readily converted to KAT **2 a** in 80 % yield after treatment with aqueous KHF_2_ (Scheme 3b). To our surprise, analysis of the catalytic reaction under standard conditions before treatment with aqueous KHF_2_ indicated results distinct from those of the stoichiometric reactions. From toluene, an acylboronate ester can be obtained and analyzed by ^11^B and ^1^H NMR spectroscopy with characteristic aromatic peaks at 8.47 and 7.20 ppm in *d_8_*‐THF, shifted to low‐field by ca. 0.2 ppm compared with **Im‐2** obtained from the aforementioned stoichiometric reaction. This new lithium acylBpinOBpin salt was isolated from 1,4‐dioxane/*n*‐hexane and characterized by a combination of NMR spectroscopy, HRMS, and single‐crystal X‐ray diffraction analysis (Figure 2). In the solid‐state, a dimeric structure was observed containing anionic 4‐coordinate boron centers with pinBO^−^ attached to the acylboronate moiety. The chelation between the lithium cations and the oxygen atoms of the carbonyl, Bpin and OBpin groups led to two 4‐coordinate, tetrahedral lithium cores with Li−O bond lengths ranging from 1.907(4) to 1.994(4) Å, contributing to the stability of the Bpin‐derived lithium acylboronate **Im‐3**. The pinBO^−^ anion was most probably generated in situ from *t*‐BuOBpin via an E1 elimination process^[26]^ in the presence of 3‐coordinate acylBpin **Im‐1** as a Lewis acid. Detection of isobutene by GC‐MS and HRMS from the reaction mixture supported this proposal (Supporting Information‐Figure S4).

LiO*t*‐Bu can serve as a nucleophile to react with acyl chlorides to form esters. This side reaction may be more competitive when excess LiO*t*‐Bu was used. Interestingly, ^1^H NMR monitoring of the esterification of **1 a** with LiO*t*‐Bu in *d_8_*‐toluene indicated only 14 % conversion in 30 min (Supporting Information‐Figure S6), while ^11^B NMR monitoring of the reaction of **1 a** with (IDipp)CuBpin in the presence of LiO*t*‐Bu showed that the reaction was complete within 10 min as inidcated by the consumption of (IDipp)CuBpin (δ=42.1 ppm) (Supporting Information‐Figure S2). These control experiments illustrated that the reaction of the acyl chloride with (IDipp)CuBpin is much faster than that with LiO*t*‐Bu, and thus kinetic control by slow addition of a solution of acyl chloride can suppress this side reaction.

**Scheme 4:**
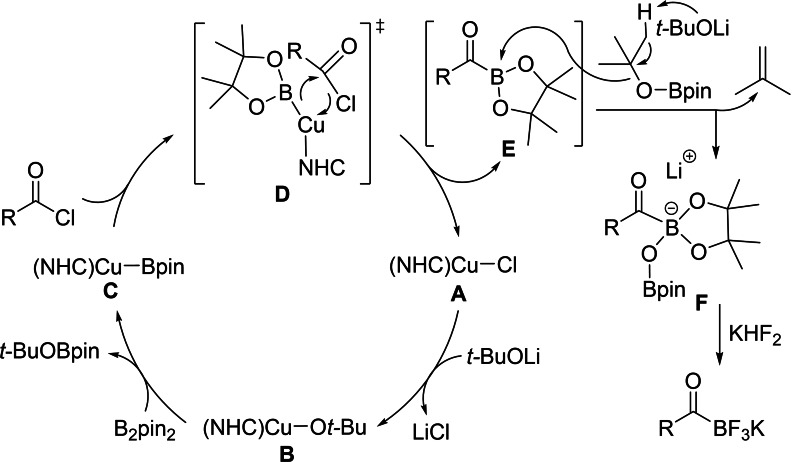
Proposed mechanism of the catalytic borylation of acyl chlorides.

A postulated catalytic reaction pathway is shown in Scheme 4. The key C−B bond formation step involves nucleophilic attack of (NHC)Cu−Bpin (**C**) on the electrophilic carbon of the acyl chloride, accompanied by the release of (NHC)Cu−Cl (**A**), to generate 3‐coordinate acylBpin **E**. This could involve either a σ‐bond methathesis process or an ‘oxidatively added transition state’ (OATS) as proposed for the Cu(I) catalyzed borylation of ArX systems.^[23b]^ The *t*‐BuOBpin, generated by the reaction of (NHC)CuO*t*‐Bu with B_2_pin_2_ as a side product, readily undergoes E1 elimination^[26]^ in the presence of acidic acylBpin **E** to form BpinO^−^, which then coordinates to **E** to form acylBpinOBpin **F**. In situ treatment of **F** with aqueous KHF_2_ gave the air stable KAT products. Other steps, (**A** to **B**)^[27]^ and (**B** to **C**),^[16]^ are likely rapid, and related stoichiometric reactions have been reported previously.

We then evaluated the scope of this transformation using electronically and structurally diverse acyl chlorides under standard conditions. We first evaluated acyl chlorides bearing aryl substituents (Table 2). Generally, electron‐donating groups on the aryl ring resulted in higher yields of the KATs than did electron‐withdrawing substituents. Products (**2 m**–**2 o**) bearing electron‐withdrawing trifluoromethoxy, trifluoromethyl, and cyano groups were isolated as trifluoroborate iminium zwitterions (TIMs) after column chromatography. An acyl chloride bearing an *o*‐tolyl group gave the desired product **2 c** in comparable yield with the *p*‐ and *m*‐tolyl‐substituted ones whereas sterically hindered 2,4,6‐trimethylbenzoyl chloride failed to deliver the desired product. In addition, π‐extended substrates such as 4‐biphenyl, 2‐naphthyl, and 1‐fluorenyl carbonyl chlorides were well‐tolerated under the standard conditions, affording **2 f**–**2 h** in good yields (65 % to 78 %). Halides (−F, −Cl, −Br and −I) on the phenyl ring (**2 i**–**2 l**) also survived during the reaction, providing potential for further derivatizations such as cross‐couplings. As the Cu‐catalyzed borylation of ArX (X=I, Br, Cl) is known to provide aryl boronates, the chemo‐selectivity observed clearly indicates the faster reaction between the nucleophilic copper boryl species and the more electrophilic acyl chloride moiety. Heterocycles such as 3‐furyl, 2‐thiophenyl and 3‐pyridyl were also compatible. All of the KAT and TIM products were isolated and characterized by NMR spectroscopy and HRMS analysis. The single‐crystal structure of **2 a** is shown in Table 2.

**Table 2:**
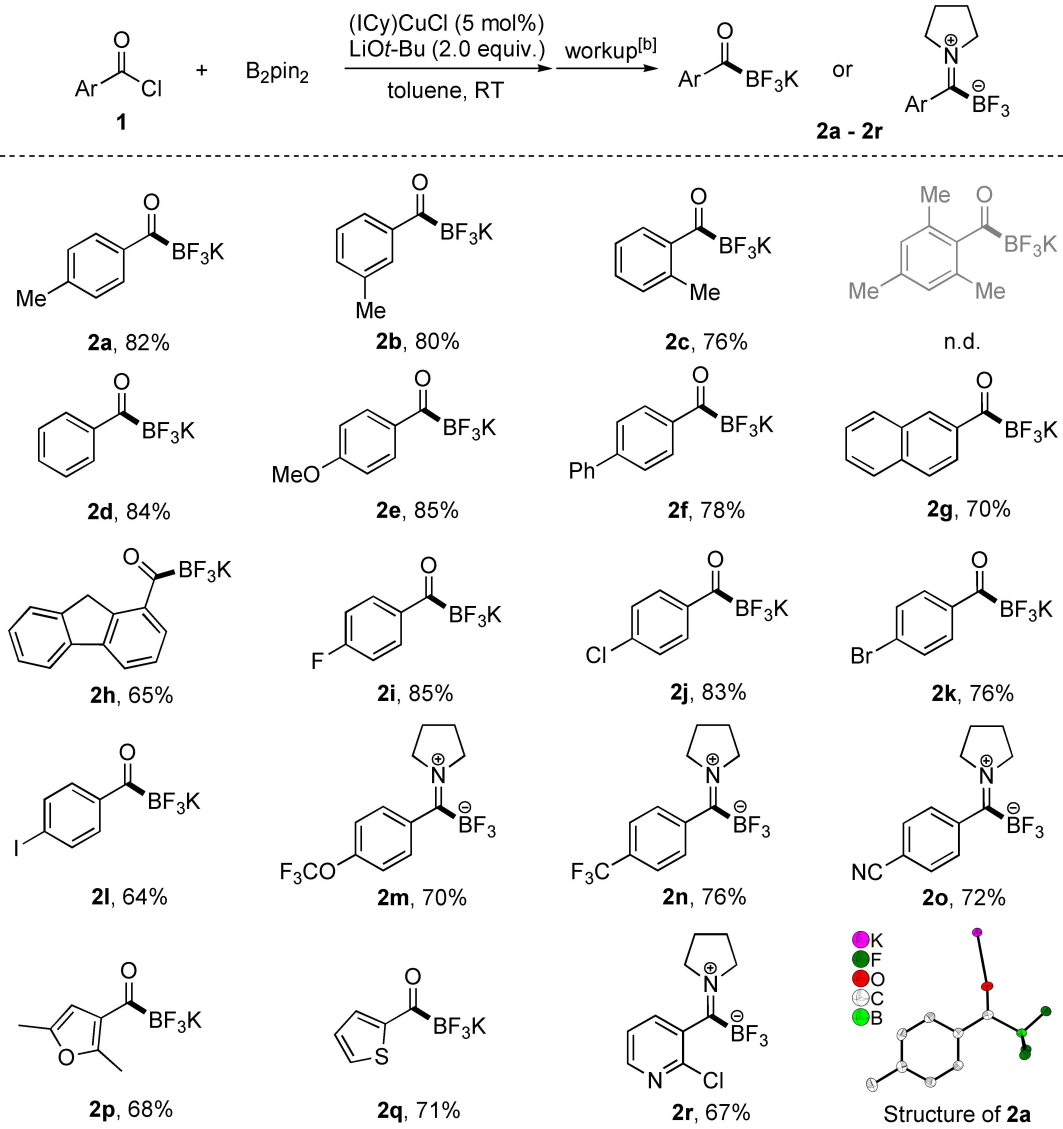
Substrate scope of acyl chlorides with aryl‐substituents.^[a]^

[a] Reactions were conducted on a 0.3 mmol scale. All yields are isolated yields. [b] Synthesis and workup of KATs according to general procedure‐1 in the experimental section and Supporting Information; synthesis and workup of trifluoroborate iminiums (TIMs) according to general procedure‐2 in the Supporting Information.

We then examined alkyl‐substituted acyl chlorides, and the room‐temperature reaction conditions allowed for a wide choice of such substrates with good chemo‐selectivity providing alkyl‐substituted acylboron compounds containing various carbon skeletons and different functional groups (Table 3). A series of primary alkyl‐substituted acyl chlorides provided the corresponding products in high yields (**4 a**–**4 h**). In particular, chloroalkyl, bromoalkyl, terminal alkene and carbonyl groups were compatible with our reaction conditions (**4 f**–**4 i**), whereas a terminal alkyne moiety was not tolerated, probably due to its high reactivity with copper boryl complexes.^[28]^ Secondary alkyl‐substituted acyl chlorides were also tolerated, providing both acylic and cyclic secondary alkyl acylboron compounds in moderate to good yields (**4 j**–**4 n**). The products can be isolated as MIDA boronates in moderate yields after reaction with MIDA in DMSO at 100 °C, as exemplified by **4 n**. Tertiary alkyl carbonyl chlorides were also compatible with the borylation process, providing otherwise challenging tertiary alkyl‐substituted KATs in high yields (**4 o**–**4 r**). An oxygen‐free atmosphere is necessary for the reaction set up and workup with aqueous KHF_2_ as the in situ generated acylboronate intermediates are air‐sensitive, especially the alkyl‐substituted ones.

**Table 3:**
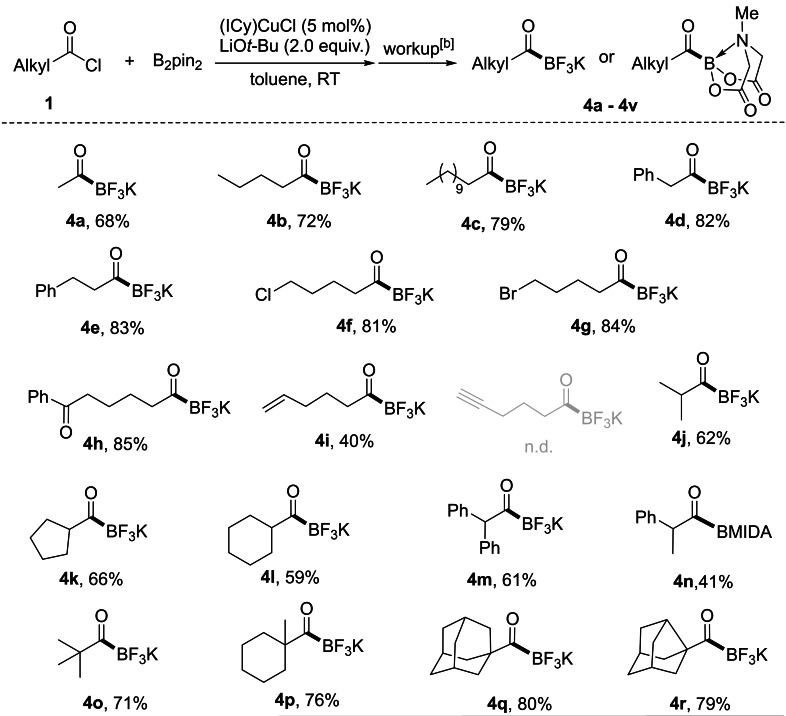
Substrate scope of acyl chlorides with alkyl‐substituents.^[a]^

[a] Reactions were conducted on 0.3 mmol scale. All yields are isolated yields. [b] Synthesis and workup of KATs according to general procedure‐1; synthesis and workup of acyl MIDA boronates according to general procedure‐3 in the Supporting Information.

The copper‐catalyzed reaction is amenable for the synthesis of drug‐derived acylboron compounds (**5 a**–**5 e**; Table 4), as evidenced by the late‐stage derivatization of Ibuprofen, Flurbiprofen, Naproxen and Ketoprofen via a 3‐step, 1‐pot tandem reaction from the corresponding carboxylic acids. Furthermore, the anti‐cancer drug Chlorambucil can be conveniently converted into the corresponding acyl MIDA boronate **5 f** in 43 % yield. The applicability of this borylation procedure is further illustrated by the gram‐scale (5 mmol) synthesis of **5 a** and **5 c** in 64 % and 71 % yields, respectively (Supporting Information‐Scheme S9). The reaction can be performed, without using the glove‐box, by pre‐mixing the carbene precursor (ICy⋅HBF_4_), copper(I) chloride and LiO*t*‐Bu in THF to generate the (ICy)CuO*t*‐Bu in situ. Slow addition of the acyl chloride under an argon atmosphere during the reaction and KHF_2_ workup are essential in the scale‐up reaction under current conditions. The successful synthesis of drug‐derived acylboron compounds may provide new opportunities for the efficient conjugation of small molecule drugs with large and complicated molecules, such as peptides or proteins.^[6]^

**Table 4:**
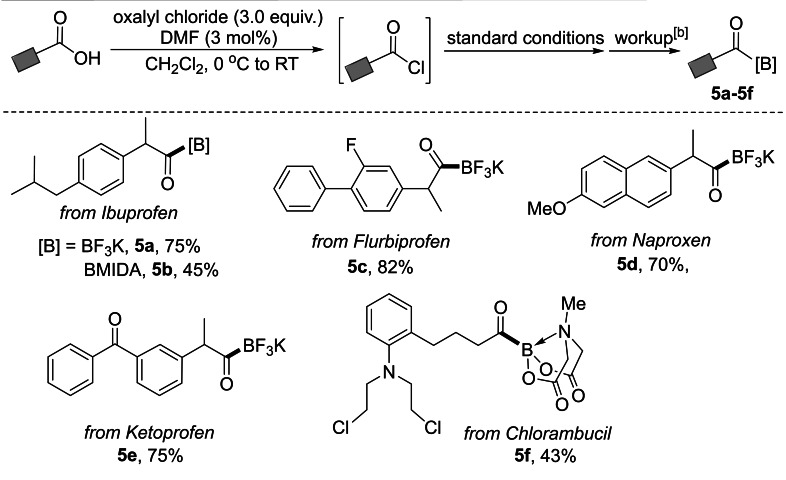
Late‐stage modification of carboxylic‐acid containing drugs.^[a]^

[a] Reactions were conducted on 0.3 mmol scale. All yields are isolated yields. [b] Synthesis and workup of KATs according to general procedure‐1; synthesis and workup of acyl MIDA boronates according to general procedure‐3 in the Supporting Information.

## Conclusions

We have developed a convenient catalytic process for the borylation of acyl chlorides to provide KATs or acyl MIDA boronates in good yields. This process features an inexpensive metal (Cu) catalyst, mild reaction conditions and good functional group tolerance. Considering the abundance of carboxylic acid derivatives and the large‐scale commercial availability of diboron reagents (e. g. B_2_pin_2_ and B_2_neop_2_), the consequent cost reduction to produce acylboron compounds makes this process very attractive.

## Experimental Section

**General procedure‐1 for the synthesis of potassium acyltrifluoroborates (KATs)**: In an argon‐filled glove box, B_2_pin_2_ (1.0 equiv., 0.3 mmol, 77 mg), LiO*t*‐Bu (2.0 equiv., 0.6 mmol, 48 mg), [(ICy)CuCl] (5 mol %, 5 mg) and toluene (2 mL) were added to a 20 mL thick‐walled reaction tube equipped with a magnetic stirring bar. The reaction tube was sealed with a crimped septum cap, removed from the glove box, and stirred at room temperature for 0.5 h. The acyl chloride (1.1 equiv., 0.33 mmol) dissolved in 2 mL of toluene was added dropwise to the tube via syringe under an argon atmosphere. The reaction mixture was stirred at room temperature for 6 h. Upon completion, aqueous KHF_2_ (2.7 M, 2.7 mmol in 1.0 mL of H_2_O, 9.0 equiv.) was added at room temperature under argon, followed by addition of THF (4 mL), and the reaction was stirred at room temperature for another 24 h. The resulting mixture was opened to air and concentrated under reduced pressure. Acetone was added to dissolve the organic solid residue and the solution was filtered through a pad of celite. The filtrate was concentrated under reduced pressure and the resulting solid was washed with Et_2_O and dried *in vacuo* to afford the corresponding potassium acyltrifluoroborates (KATs).

### X‐ray crystallography

Deposition Numbers 2129577 (**3**), 2129578 (**2a**) and 2152003 **(Im‐3**) contain the supplementary crystallographic data for this paper. These data are provided free of charge by the joint Cambridge Crystallographic Data Centre and Fachinformationszentrum Karlsruhe Access Structures service www.ccdc.cam.ac.uk/structures.

## Conflict of interest

The authors declare no conflict of interest.

1

## Supporting information

As a service to our authors and readers, this journal provides supporting information supplied by the authors. Such materials are peer reviewed and may be re‐organized for online delivery, but are not copy‐edited or typeset. Technical support issues arising from supporting information (other than missing files) should be addressed to the authors.

Supporting Information

## Data Availability

The data that support the findings of this study are available in the supplementary material of this article.
