# Supplementary material for: Copper‐Catalyzed Borylation of Acyl Chlorides with an Alkoxy Diboron Reagent: A Facile Route to Acylboron Compounds
Source: Chemistry. 2022 Jun 13;28(42):e202201329. doi: 10.1002/chem.202201329 (PMC9400893; doi:10.1002/chem.202201329)
Supplement: Supplementary file 1 — Supporting Information [file CHEM-28-0-s001.pdf]

Chemistry–A European Journal

Supporting Information

Copper-Catalyzed Borylation of Acyl Chlorides with an Alkoxy Diboron Reagent: A Facile Route to Acylboron Compounds

Xiaolei Zhang,* Alexandra Friedrich, and Todd B. Marder*

Supporting Information

Table of contents

1. General experimental details…………………………..……………………..….S1-S3

2. Experimental section………………………………………..……….…...…S4-S41

2.1 Reaction optimization…………………………………………………….…..…S4-S5

2.2 Mechanistic studies…………………………………………………….…..….S6-S14

2.3 Substrate scope…………………………………………........................…S15-S40

2.4 Scale-up reaction……….…..………………………………………………….. S40-S42

3. Crystallographic data……………………………………………………….. S43-S46

4. References…………………………………………..………………….....…S47

5. NMR spectra…………………………………………………………………S48-S145

1. General experimental details

Unless otherwise noted, the following conditions were applied. All syntheses were carried out using standard Schlenk and glovebox techniques under an argon atmosphere. The solvents used were dried using a solvent purification system (SPS) from Innovative Technology and were degassed and stored under argon. Deuterated solvents ($CDCl_3$, *d_6*-acetone, *d_6*-DMSO, *d_8*-toluene) used for NMR spectroscopy were purchased from Cambridge Isotope Laboratories. Unless other wisely noted, common commercial reagents were purchased from commercial sources and were used without further purification. *p*-Toluoyl chloride and copper(I) chloride were purchased from Sigma-Aldrich. LiO*t*-Bu was purchased from Alfa Aesar. B_2pin_2 and B_2neop_2 were kindly provided by AllylChem Co. Ltd. The carbene-ligated copper complexes [(IDipp)CuCl],[S1] [(ICy)CuCl],[S2] [(IMes)CuCl],[S1] [(I*i*-Pr)CuCl],[S1] [(IDipp)CuBpin][S3] were synthesized according literature procedures. Acyl chloride substrates were either purchased from commercial source or synthesized from the correspond carboxylic acid according to literature procedures.[S4]

Column chromatography was performed with Silica Gel 60 (40-63 μ) (purchased from VWR). Precoated TLC plates (Polygram@ Sil G/UV254) were purchased from Machery-Nagel. Solvents were generally removed using a rotary evaporator *in vacuo* at a maximum temperature of 55 ^{o}C.

NMR spectra were recorded on Brucker Avance III HD 300 NMR (operating at ^{1}H: 300 MHz, ^{11}B{^{1}H}: 96 MHz, ^{13}C{^{1}H}: 75 MHz), Bruker Avance 500 FT NMR (operating at ^{1}H: 500 MHz, ^{11}B{^{1}H}: 160 MHz, ^{13}C{^{1}H}: 126 MHz, ^{19}F{^{1}H}: 471 MHz) or Bruker Avance Neo I 600 NMR (operating at ^{1}H: 600 MHz, ^{11}B{^{1}H}: 192 MHz, ^{13}C{^{1}H}: 151 MHz, ^{19}F{^{1}H}: 565 MHz) spectrometers. Chemical shifts (δ) are given in ppm and are referenced to external $BF_3{\cdot}Et_2O$ (^{11}B{^{1}H}), $CFCl_3$ (^{19}F{^{1}H}). ^{1}H NMR spectra were referenced via residual proton resonances of $CDCl_3$ (^{1}H, 7.26 ppm), *d_6*-acetone (^{1}H, 2.05 ppm), *d_6*-DMSO (^{1}H, 2.50 ppm), *d_8*-toluene (^{1}H, 2.09 ppm), *d_8*-THF (^{1}H, 3.58 ppm). ^{13}C{^{1}H} spectra were referenced to $CDCl_3$ (^{13}C{^{1}H}, 77.16 ppm), *d_6*-acetone (^{13}C{^{1}H}, 206.26 ppm), *d_6*-DMSO (^{13}C{^{1}H}, 39.52 ppm), *d_8*-toluene (^{13}C{^{1}H}, 20.40 ppm), *d_8*-THF (^{13}C{^{1}H}, 67.21 ppm).

High resolution mass spectrometry (HRMS) was recorded using a Thermo Scientific Exactive Plus Orbitrap MS system for electrospray ionization-mass spectrometry (ESI-MS), atmospheric pressure chemical ionization (APCI) and atmospheric solids analysis probe (ASAP). **GC-MS analyses** were performed using an Agilent 7890A gas chromatograph (column: HP-5MS 5% phenyl methyl siloxane, 30 m, Ø 0.25 mm, film 0.25 µm; injector: 250 °C; oven: 40 °C (2 min), 40 °C to 280 °C (20 °C min– 1); carrier gas: He (1.2 mL min^{-1}) equipped with an Agilent 5975C inert MSD with triple-axis detector operating in EI mode and an Agilent 7693A series auto sampler/injector. **Elemental analyses** were performed on a Leco CHNS-932 Elemental Analyzer. **Melting Points** were determined using a B-540 Melting Point device of BÜCHI. **Infrared (IR)** data were obtained on Brucker Alpha II compact FT-IR spectrometer.

Single-crystal X-ray diffraction: Crystals suitable for single-crystal X-ray diffraction were selected, coated in perfluoropolyether oil, and mounted on a polyimide microloop. Diffraction data of **2a**, **3** and **Im-3** were collected on a Rigaku Oxford Diffraction XTALAB SYNERGY-R diffractometer with a semiconductor HPA area detector (HyPix-Arc150) using a rotating-anode X-ray tube for X-ray generation and multi-layer mirror monochromated Cu-K_{α} radiation. The crystals were cooled using an Oxford Cryostreams 800 low-temperature device. Data were collected at 100 K. The images were processed and corrected for Lorentz-polarization effects and absorption as implemented in the $CrysAlis^{Pro}$ software. The structures were solved using the intrinsic phasing method[S5] and Fourier expansion technique. All non-hydrogen atoms were refined in anisotropic approximation, with hydrogen atoms 'riding' on idealised positions by full-matrix least squares against F^2 of all data, using SHELXL software[S6] and the SHELXLE graphical user interface.[S7] Full structural information for **2a**, **3** and **Im-3** have been deposited with Cambridge Crystallographic Data Centre. CCDC 2129577, 2129578 and 2152003.

2. Experimental section

2.1 Reaction optimization

Procedure for reaction optimization. In an argon-filled glovebox, B_2pin_2 (0.3 mmol, 1.0 equiv.), base (x equiv.), copper catalyst (5 mol%), and solvent (2 mL) were added to a 20 mL thick-walled reaction tube equipped with a magnetic stirring bar. The reaction tube was sealed with a crimped septum cap, removed from the glovebox and stirred at room temperature for 0.5 h. *p*-Toluoyl chloride **1a** (1.1 equiv., 0.33 mmol, 43 μL) dissolved in 2 mL of solvent was added dropwise to the tube via syringe under an argon atmosphere. The reaction mixture was stirred at room temperature or otherwise noted temperature for 6 h. Upon completion, aqueous KHF_2 (2.7 M, 2.7 mmol in 1.0 mL of H_2O, 9.0 equiv.) was added at room temperature under argon, followed by addition of THF (4 mL), and the reaction was stirred at room temperature for another 24 h. The resulting mixture was opened to air and the reaction mixture was concentrated under reduced pressure. Acetone was added to dissolve the organic residue and the solution was filtered through a pad of celite. The filtrate was concentrated under reduced pressure and the resulting solid was washed with Et_2O, dried *in vacuo* to afford **2a** as a white solid.

Table S1. Optimization of reaction conditions. [a]

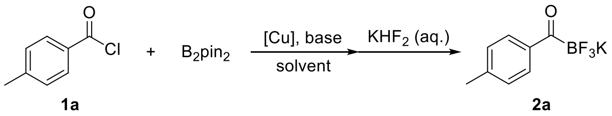

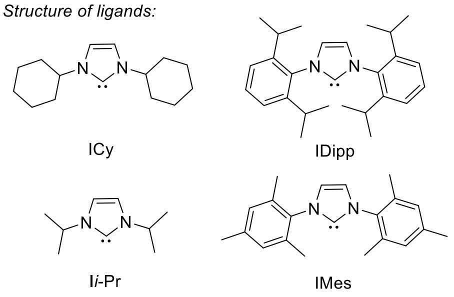

Entry	[Cu] (mol%)	Base (equiv.)	Solvent	Isolated yield of **2a**[b]
1	[(ICy)CuCl] (5)	NaO*t*-Bu (0.1)	toluene	n.d.[c]
2	[(ICy)CuCl] (5)	NaO*t*-Bu (1.0)	toluene	n.d.
3	[(ICy)CuCl] (5)	LiO*t*-Bu (1.0)	toluene	37
4	[(ICy)CuCl] (5)	KO*t*-Bu (1.0)	toluene	8
5	[(ICy)CuCl] (5)	LiOMe (1.0)	toluene	11
6	[(ICy)CuCl] (5)	KOMe (1.0)	toluene	8
7	[(ICy)CuCl] (5)	Li_2CO_3 (1.0)	toluene	n.d.
8	[(ICy)CuCl] (5)	K_2CO_3 (1.0)	toluene	n.d.
9	[(ICy)CuCl] (5)	KF (1.0)	toluene	trace
10	[(ICy)CuCl] (5)	LiO*t*-Bu (1.5)	toluene	65
11	[(ICy)CuCl] (5)	LiO*t*-Bu (2.0)	toluene	82
12	[(ICy)CuCl] (5)	none	toluene	n.d.
13	none	LiO*t*-Bu (2.0)	toluene	n.d.
14	[(IDipp)CuCl] (5)	LiO*t*-Bu (2.0)	toluene	28
15	[(IMes)CuCl] (5)	LiO*t*-Bu (2.0)	toluene	35
16	[(I*i*-Pr)CuCl] (5)	LiO*t*-Bu (2.0)	toluene	75
17	[(ICy)CuCl] (5)	LiO*t*-Bu (2.0)	Et_2O	64
18	[(ICy)CuCl] (5)	LiO*t*-Bu (2.0)	THF	44
19	[(ICy)CuCl] (5)	LiO*t*-Bu (2.0)	1,4-dioxane	52
20	[(ICy)CuCl] (5)	LiO*t*-Bu (2.0)	MeCN	15
21	[(ICy)CuCl] (5)	LiO*t*-Bu (2.0)	*n*-hexane	47
22	[(ICy)CuCl] (5)	LiO*t*-Bu (2.0)	CH_2Cl_2	40
23[d]	[(ICy)CuCl] (5)	LiO*t*-Bu (2.0)	toluene	55
24[e]	[(ICy)CuCl] (5)	LiO*t*-Bu (2.0)	toluene	52
25[f]	[(ICy)CuCl] (5)	LiO*t*-Bu (2.0)	toluene	61
26[g]	[(ICy)CuCl] (5)	LiO*t*-Bu (2.0)	toluene	complicated
27[h]	[(ICy)CuCl] (5)	LiO*t*-Bu (2.0)	toluene	n.d.

[a] Reaction conditions, unless otherwise stated: *p*-toluoyl chloride **1a** (0.33 mmol, 1.1 equiv.), B_2pin_2 (1.0 equiv.), solvent (4 mL), room temperature, 6 h, argon atmosphere, a solution of **1a** in the corresponding solvent (2 mL) was added dropwise. Workup: KHF_2 (9.0 equiv., 2.7 M in H_2O, 1.0 mL), THF (4 mL), 24 h. [b] Isolated yield. [c] Product not detected. [d] **1a** Was added in one portion. [e] Heating to 60 ^oC. [f] Using B_2neop_2 as the borylating reagent. [g] Using

B_2cat_2 as the borylating reagent. [h] Reaction conducted under an air atmosphere.

2.2 Mechanistic studies

Notes: [1,3-Bis(2,6-diisopropylphenyl)imidazol-2-ylidene]copper(I) pinacolatoboryl, [(IDipp)CuBpin] was synthesized according to the literature procedure and the product typically contained ~5% of an –OBpin related impurity which is consistent with the literature report.[S3]

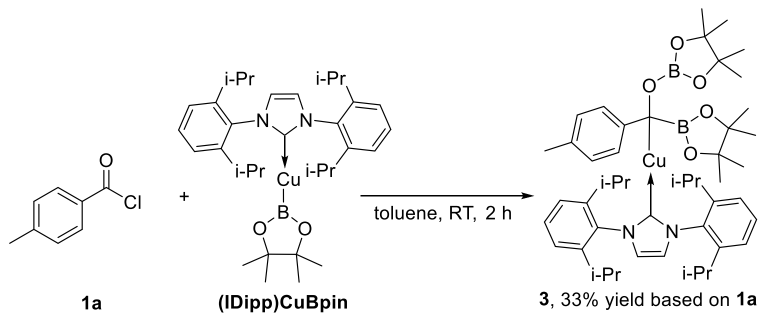

Scheme S1. Reaction of **1a** with the copper(I) boryl complex [(IDipp)CuBpin].

2.2.1 Synthesis of 3.

In an argon-filled glovebox, *p*-toluoyl chloride **1a** (0.2 mmol, 31 mg) and toluene (2 mL) were added to a 10 mL reaction tube equipped with a magnetic stir bar. Then, [(IDipp)CuBpin] (0.2 mmol, 116 mg) and toluene (2 mL) were added to another tube (10 mL) and shaken to form a solution. The toluene solution of [(ImDipp$_2$)CuBpin] was added to the toluene solution of **1a** and the mixture was stirred for 2 h at room temperature. The resulting brown suspension was filtered through a pad of cotton and the solid was washed with toluene (3 mL). The solid was collected, dried and shown to be [(IDipp)CuCl] by ^{1}H NMR comparison with a known sample. The solvent of the filtrate was concentrated to 1 mL under reduced pressure and was layered with *n*-hexane at –30 °C giving **3** as a crystalline solid. The solid was collected and washed with *n*-hexane (1 mL), and dried *in vacuo*. A single crystal of **3** suitable for X-ray diffraction analysis was obtained by slow diffusion of *n*-hexane into a toluene solution of **3** at –30 °C.

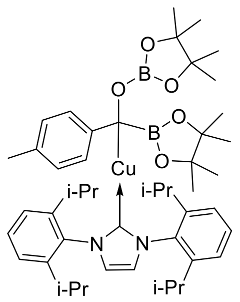

3, white solid, 55 mg, yield 33% based on **1a**.

^{1}H NMR (600 MHz, *d_8*-toluene): δ 7.20 (t, *J* = 8 Hz, 2H, Ar–*H*), 7.05 (dd, *J* = 8, 1 Hz, 2H, Ar–*H*), 7.02 – 7.00 (m, overlapped with toluene, 4H, Ar–*H*), 6.74 (d, *J* = 7.8 Hz, 2H, Ar–*H*), 6.29 (s, 2H, Imidazole-*H*), 2.52 (sep, *J* = 7 Hz, 2H, *i*-Pr–*H*), 2.42 (sep, *J* = 7 Hz, 2H, *i*-Pr-*H*), 2.29 (s, 3H, *p*-tolyl–C*H*$_3$), 1.44 (d, 7 Hz, 2H, *i*-Pr–*CH$_3$*), 1.15 (d, 7 Hz, 2H, *i*-Pr–*CH$_3$*), 1.06 – 1.03 (m, overlapped, 30H), 0.99 (s, 6H) ppm.

^{11}B{^{1}H} NMR (96 MHz, *d_8*-toluene): δ 21.9 (br) ppm.

^{13}C{^{1}H} NMR (151 MHz, *d_8*-toluene): δ 184.4 (imidazole-*C*), 147.5 (Ar–*C*), 145.7 (Ar–*C*), 145.6 (Ar–*C*), 135.2 (Ar–*C*), 130.3 (Ar–*C*), 128.4 (Ar–*C*), 124.2 (Ar–*C*), 124.1 (Ar–*C*), 119.7 (Ar–*C*), 122.5 (imidazole-*C*H), 81.1 (Bpin–*C*), 80.8 (Bpin–*C*), 29.1 (*i*-Pr–*C*), 29.0 (*i*-Pr–*C*), 25.4 (Bpin–*C*H$_3$), 25.1 (Bpin–*C*H$_3$), 24.8 (Bpin–*C*H$_3$), 24.6 (*i*-Pr–*C*H$_3$), 24.4 (*i*-Pr–*C*H$_3$), 24.0 (*i*-Pr–*C*H$_3$), 23.8 (*i*-Pr–*C*H$_3$), 21.7 (*p*-tolyl–*C*H$_3$) ppm. The carbon (C1) directly attached to boron was not detected in the ^{13}C NMR spectrum, likely due to quadrupolar broadening.

HRMS (APCI): m/z calcd for $C_{41}H_{55}BCuN_2O_3$ [M–Bpin]: 697.3602. Found: 697.3578.

Elem. Anal.: Calc. (%) for $C_{47}H_{67}B_2CuN_2O_5$: C 68.41, H 8.18. Found: C 68.67, H 8.23.

2.2.2 HRMS detection of the formation of a 3-coordinate acylBpin compound at the early stage of the reaction

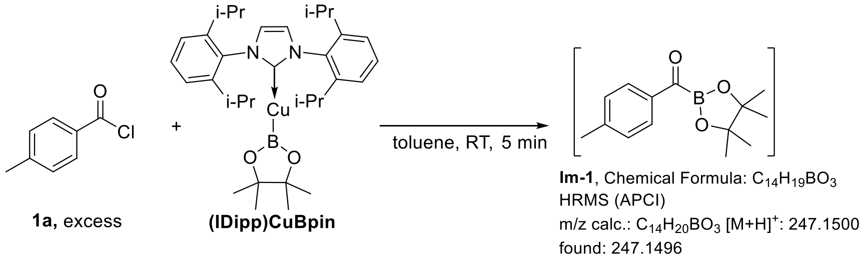

Scheme S2. *In situ* detection of a 3-coordinate acylBpin compound by HRMS after 5 min.

In an argon-filled glovebox, *p*-toluoyl chloride **1a** (0.1 mmol, 15 mg, 5.0 equiv.) and toluene (1 mL) were added to a reaction tube equipped with a magnetic stir bar. Then, [(IDipp)CuBpin] (0.02 mmol, 12 mg) and toluene (1 mL) were added to another tube. The toluene solution of [(IDipp)CuBpin] was added to the toluene solution of **1a** and the mixture was stirred for 2 min at room temperature. The resulting suspension was filtered and transferred into a vial and the solution was immediately analyzed by HRMS (ASAP). The 3-coordinate acylboron compound with the formula of $C_{14}H_{19}BO_3$ was detected as its protonated cation (Figure S1). m/z Calc. $C_{14}H_{20}BO_3$ $[M+H]^+$: 247.1500. Found: 247.1496.

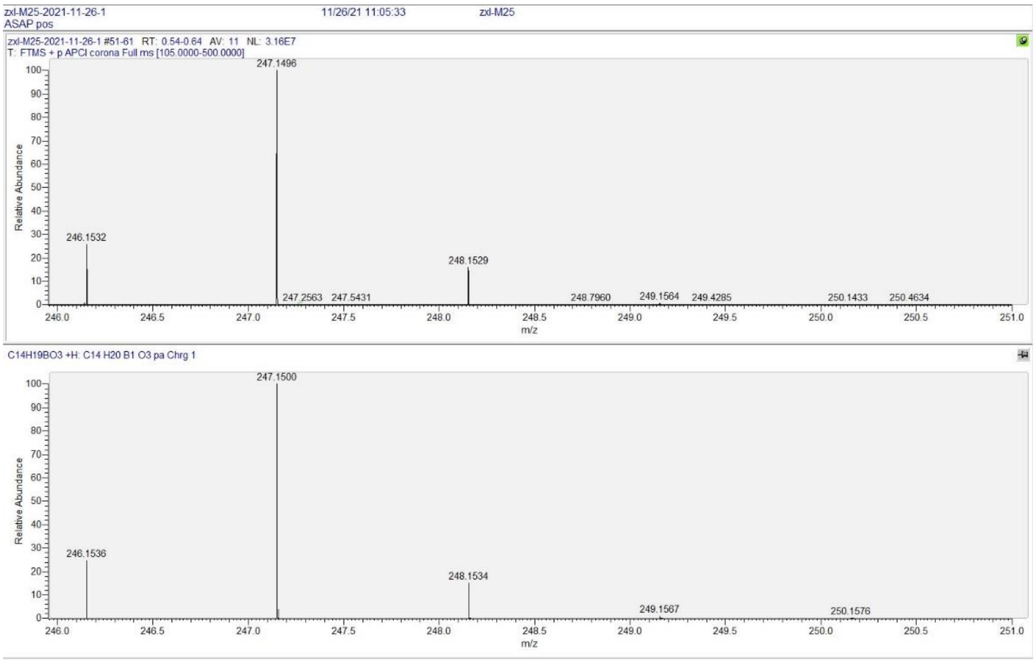

Figure S1. HRMS spectra of the *in situ* generated three-coordinated acylboron species. (top: found; bottom: calculated)

2.2.3 *In situ* ^{11}B{^{1}H} NMR monitoring of the reaction of 1a, [(IDipp)CuBpin], and LiO*t*-Bu

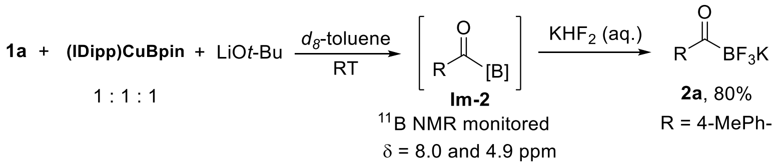

Scheme S3. Reaction of **1a** with the copper(I) boryl complex [(IDipp)CuBpin] and LiOt-Bu.

Step-1: In an argon-filled glovebox, [(IDipp)CuBpin] (0.05 mmol, 29 mg) was added to a J Young's NMR tube, followed by the addition of d_8-toluene (0.6 mL). The NMR tube was sealed and removed from the glovebox. The sample was analyzed by $^{11}B\{^1H\}$ NMR spectroscopy.

Step-2: The NMR tube was returned to the glovebox and LiO*t*-Bu (1.0 equiv., 0.05 mmol, 4 mg) was added at room temperature. The NMR tube was sealed and removed from the glovebox. The sample was shaken and analyzed by $^{11}B\{^1H\}$ NMR spectroscopy. The resulting spectrum indicated that no reaction occurred between [(IDipp)CuBpin] and LiO*t*-Bu.

Step-3: The NMR tube was returned to the glovebox and *p*-toluoyl chloride (**1a**) (1.0 equiv., 0.05 mmol, 7 μL) was added using a microsyringe at room temperature. The NMR tube was sealed and removed from the glovebox. The sample was shaken and submitted for $^{11}B\{^1H\}$ NMR analysis. Locking, shimming and data acquisition required ca. 10 min, and the resulting $^{11}B\{^1H\}$ NMR spectrum indicated that [(IDipp)CuBpin] was fully consumed with the appearance of two new boron signals at δ = 8.0 and 4.9 ppm (Figure S2).

Step-4: After the reaction, a precipitate formed. The NMR tube was returned to the glovebox and the suspension was filtered through a pad of cotton and washed with *n*-hexane (1 mL) to afford a brown residue which was washed with 10 mL of toluene. The filtrate was collected and concentrated under reduced pressure to form a white residue. To this white residue was added dioxane (1 mL) and *n*-hexane (1 mL) and the mixture was stirred for 5 min during which time a white solid formed. The suspension was filtered through a pad of cotton and the white solid was collected and analyzed by $^{11}B\{^1H\}$ and ^1H NMR spectroscopy in d_8-THF. The $^{11}B\{^1H\}$ NMR spectrum indicated a major peak in 3.6 ppm and a minor peak at 8.5 ppm (Figure S2). The ^1H NMR spectrum indicated a mixture containing *p*-toluoyl, pinacolato, and *tert*-butoxy moieties (Figure S3). Together with the $^{11}B\{^1H\}$ NMR spectra, we deduced that the reaction of **1a** with [(IDipp)CuBpin] in the presence of LiO*t*-Bu generated 4-coordinate acylboron species containing an sp^3 boron

center. However, the formation of a mixture prohibited further isolation and characterization.

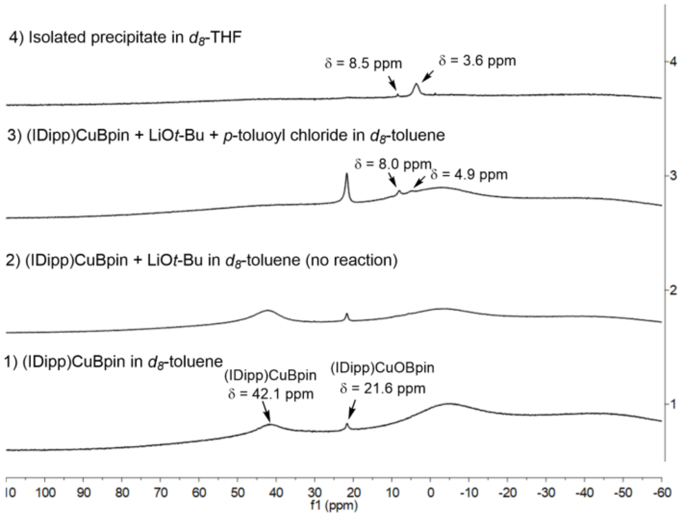

Figure S2. Monitoring of the reaction of **1a** with [(IDipp)CuBpin] in the presence of LiO*t*-Bu by $^{11}B\{^1H\}$ NMR spectroscopy (96 MHz) in d_8-toluene at room temperature.

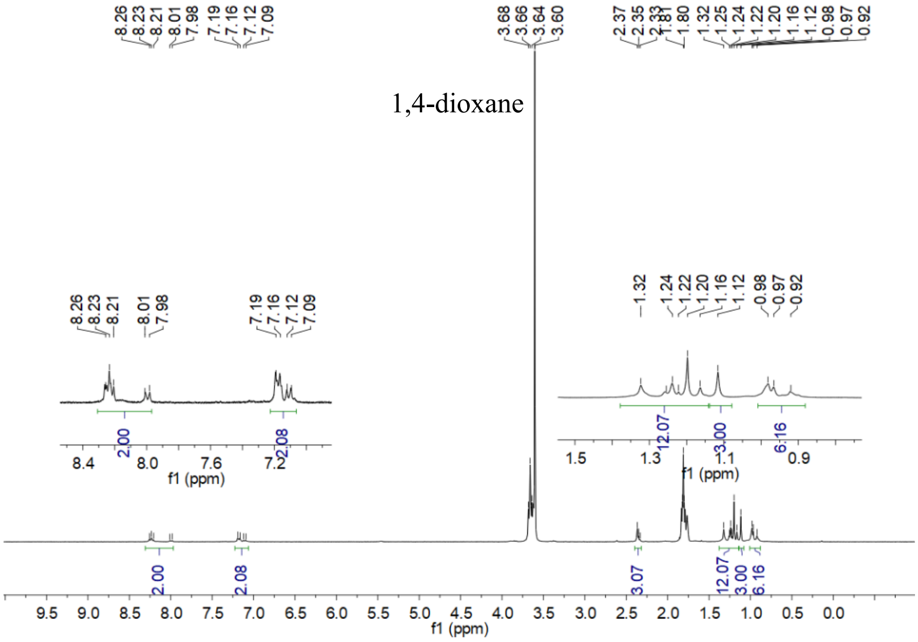

Figure S3. ^1H NMR (300 MHz) spectrum of the isolated 4-coordinate acylboron compounds in d_8-THF.

2.2.4 Synthesis of KAT 2a from the reaction of 1a, [(IDipp)CuBpin], and LiO*t*-Bu

In an argon-filled glovebox, [(IDipp)CuBpin] (0.2 mmol, 120 mg), LiO*t*-Bu (1.0 equiv., 0.2

mmol, 16 mg) and toluene (4 mL) were added to a 10 mL reaction tube equipped with a magnetic stir bar. *p*-Toluoyl chloride (**1a**) (1.0 equiv., 0.2 mmol, 26 μL) was added using a microsyringe at room temperature. The reaction mixture was stirred for 10 min. The suspension was filtered through a pad of cotton and washed with toluene. The filtrate was removed the glovebox and concentrated to 3 mL. Aqueous KHF_2 solution (1.0 M, 1.0 mmol in 1.0 mL of H_2O, 5.0 equiv.) was added at room temperature under an argon atmosphere, followed by addition of THF (4 mL) and the reaction was stirred at room temperature for another 24 h. The resulting mixture was concentrated under reduced pressure. Acetone was added to dissolve the organic solid residue and the solution was filtered through a pad of celite. The filtrate was concentrated under reduced pressure and the resulting solid was washed with Et_2O, dried *in vacuo* to afford the corresponding KAT **2a** (36 mg, 80% yield).

2.2.5 Synthesis of a lithium acylBpinOBpin compound: a new type of acylboronate species

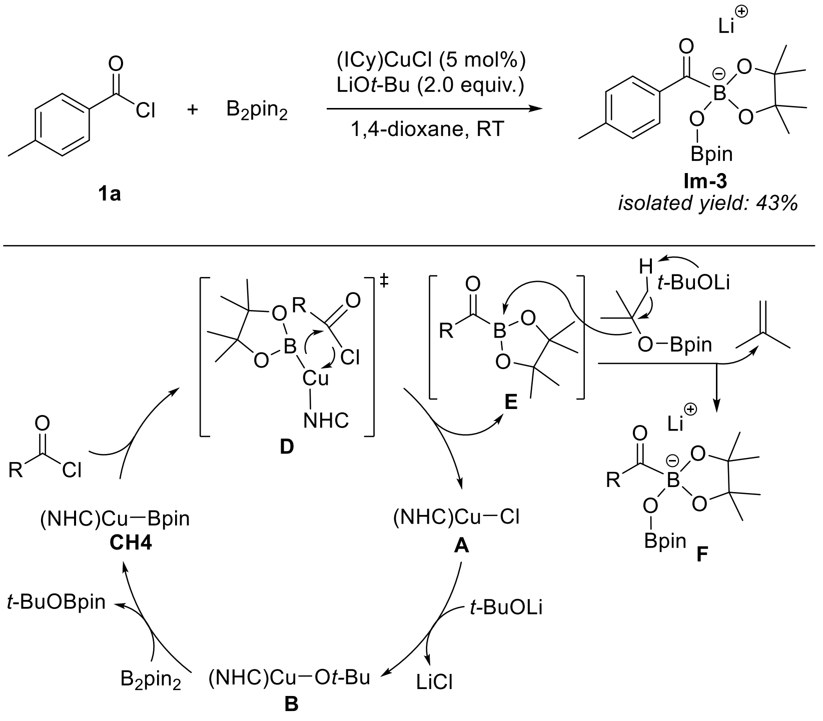

Scheme S4. [(ICy)CuCl]-catalyzed reaction of **1a** with B_2pin_2 yielding **Im-3** and the catalytic mechanism showing the formation of **Im-3**.

In an argon-filled glovebox, B_2pin_2 (0.3 mmol, 1.0 equiv., 77 mg), LiO*t*-Bu (2.0 equiv., 0.6 mmol, 48 mg), [(ICy)CuCl] (5 mol%, 5 mg) and 1,4-dioxane (2 mL) were added to a 20 mL thick-walled reaction tube equipped with a magnetic stirring bar. The reaction tube was sealed with a crimped septum cap, removed from the glovebox and stirred at room temperature for 0.5 h. *p*-Toluoyl chloride **1a** (1.1 equiv., 0.33 mmol, 43 μL) dissolved in 2 mL of 1,4-dioxane was added dropwise to the tube via syringe under an argon atmosphere. The reaction mixture was stirred at room temperature for 3 h. Upon completion, *n*-hexane (4 mL) was added to the reaction mixture, during which time a white precipitate formed. The resulting mixture was filtrated under argon atmosphere and the white precipitate was washed with toluene (4 mL) and re-dissolved in THF. The THF solution was concentrated under reduced pressure and dried *in vacuo* to afford **Im-3** as a white solid. A single crystal suitable for X-ray diffraction analysis was obtained via slow diffusion of *n*-hexane into a 1,4-dioxane solution of **Im-3**. HRMS and GC-MS analysis of the reaction mixture indicated the formation of isobutene (Figure S4), which supported the E1 elimination from *t*-BuOBpin to form **Im-3**. **HRMS** (APCI): m/z calcd for C_4H_7 [M–H]: 55.0542. Found: 55.0551. Furthermore, the formation of *t*-BuOH can also be detected by GC-MS analysis by comparison with a known sample.

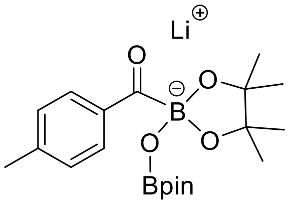

Im-3, white solid, 50 mg, yield 43%.

^{1}H NMR (300 MHz, *d*$_8$-THF): δ 8.43 (d, *J* = 7.8 Hz, 2H, Ar–*H*), 7.15 (d, *J* = 7.8 Hz, 2H, Ar–*H*), 2.33 (s, 3H, *p*-toluoyl–C*H*$_3$), 1.23 (s, 6H, Bpin–C*H*$_3$), 1.12 (s, 3H, Bpin–C*H*$_3$), 1.07 (s, 9H, Bpin–C*H*$_3$), 0.95 (s, 6H, Bpin–C*H*$_3$) ppm.

^{11}B{^{1}H} NMR (96 MHz, *d*$_8$-THF): δ 21.2 (br), 3.79 ppm.

^{13}C{^{1}H} NMR (75 MHz, *d*$_8$-THF): δ 142.3, 130.7, 128.9, 120.7, 79.5, 32.2, 21.4 ppm. The carbon directly attached to boron was not detected in the ^{13}C NMR spectrum, likely due to quadrupolar broadening.

HRMS (APCI): m/z calcd for $C_{20}H_{31}B_2O_6$ [M–Li]$^-$: 389.2312; Found: 389.2312.

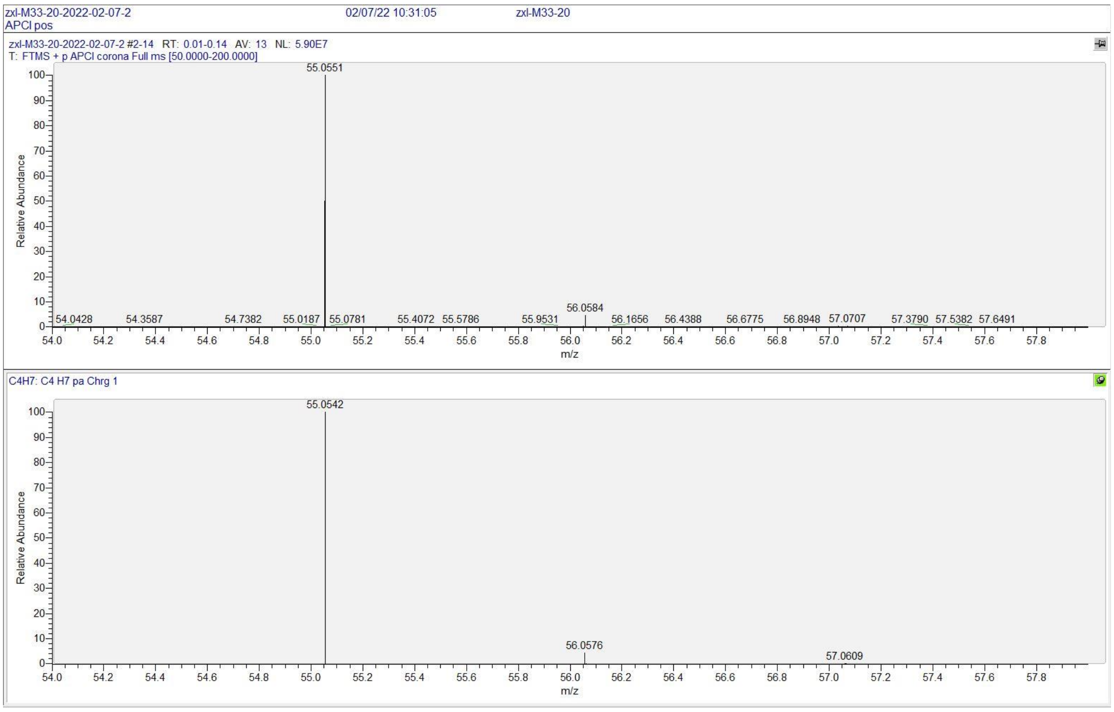

Figure S4. HRMS analysis of the reaction mixture suggesting the formation of isobutene. (top: found; bottom: calculated)

2.2.6 ^{1}H NMR monitoring of the reaction of 1a and LiO*t*-Bu in *d*$_8$-toluene

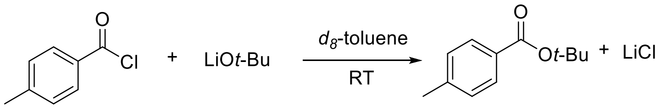

Scheme S5. Reaction of **1a** with LiOt-Bu in *d*$_8$-toluene at room temperature.

In an argon-filled glovebox, LiO*t*-Bu (1.0 equiv., 0.05 mmol, 4 mg) was added to a J Young's NMR tube, followed by the addition of *d*$_8$-toluene (0.6 mL). The NMR tube was shaken to dissolve the solid. Then, *p*-toluoyl chloride (**1a**) (1.0 equiv., 0.05 mmol, 7 μL) was added using a microsyringe at room temperature. The reaction mixture was shaken and monitored at room temperature by ^{1}H NMR analysis, initiallyafter 30 min and then every 2 h. During the reaction, only the ester product was detected. The yield was determined by the ratio of the integrals of the ester product and unconsumed **1a** in the ^{1}H NMR spectra (Figures S5 and S6).

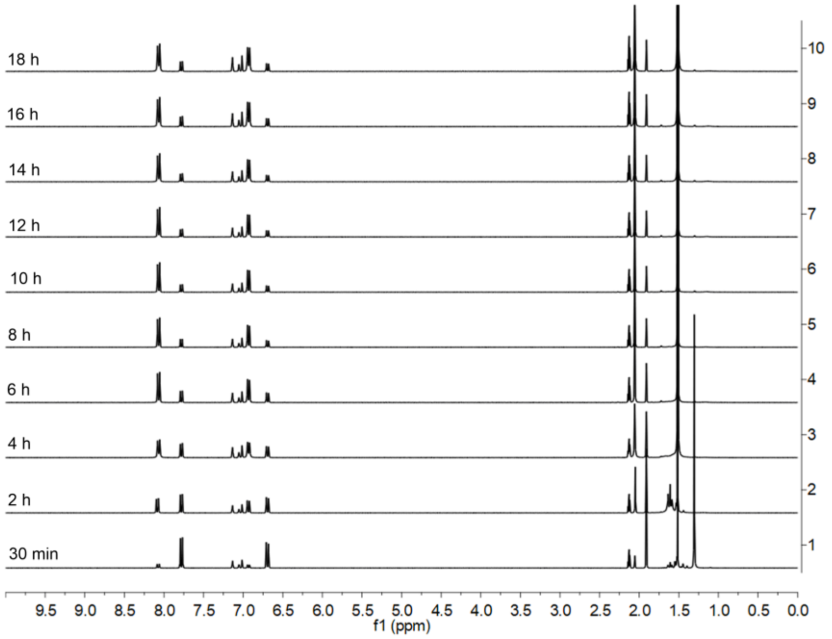

Figure S5. ^{1}H NMR (300 MHz) monitoring of the reaction of **1a** with LiO*t*-Bu in d_8-toluene at room temperature.

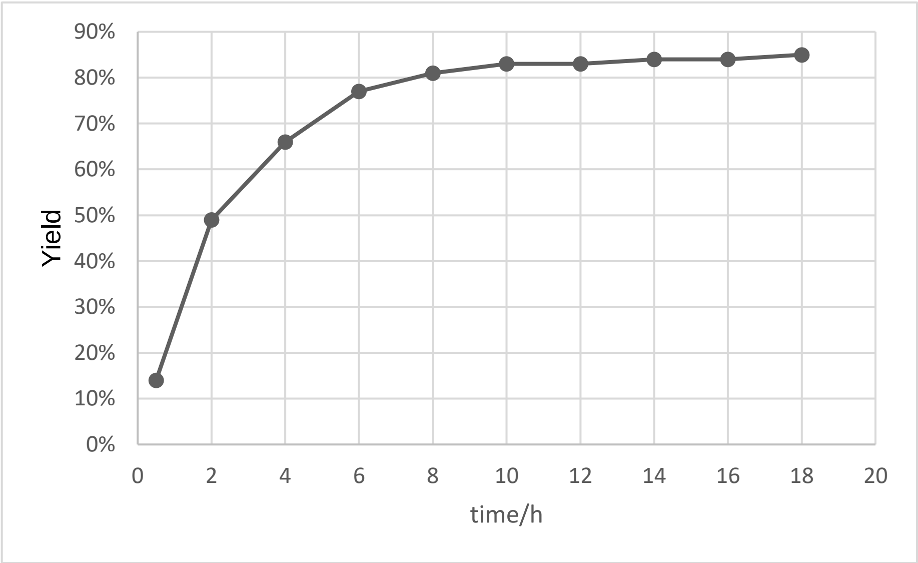

Figure S6. Yield of the ester product as a function of time.

2.3 Substrate scope

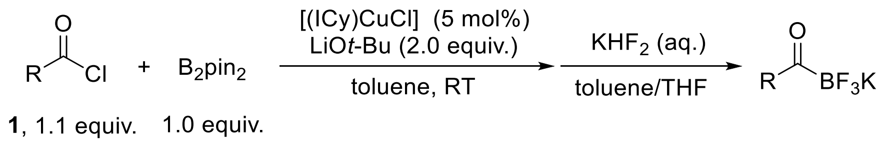

Scheme S6. Copper-catalyzed borylation of acyl chlorides with B_2pin_2 to synthesize KATs.

General procedure-1 for the synthesis of potassium acyltrifluoroborates (KATs). In an argon-filled glovebox, B_2pin_2 (1.0 equiv., 0.3 mmol, 77 mg), LiO*t*-Bu (2.0 equiv., 0.6 mmol, 48 mg), [(ICy)CuCl] (5 mol%, 5 mg) and toluene (2 mL) were added to a 20 mL thick-walled reaction tube equipped with a magnetic stirring bar. The reaction tube was sealed with a crimped septum cap, removed from the glovebox, and stirred at room temperature for 0.5 h. The acyl chloride (1.1 equiv., 0.33 mmol) dissolved in 2 mL of toluene was added dropwise to the tube via syringe under an argon atmosphere. The reaction mixture was stirred at room temperature for 6 h. Upon completion, aqueous KHF_2 (2.7 M, 2.7 mmol in 1.0 mL of H_2O, 9.0 equiv.) was added at room temperature under argon, followed by addition of THF (4 mL), and the reaction was stirred at room temperature for another 24 h. The resulting mixture was opened to air and the reaction mixture was concentrated under reduced pressure. Acetone was added to dissolve the organic solid residue and the solution was filtered through a pad of celite. The filtrate was concentrated under reduced pressure and the resulting solid was washed with Et_2O and dried *in vacuo* to afford the corresponding potassium acyltrifluoroborates (KATs).

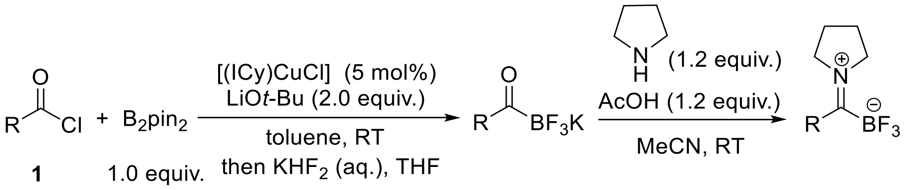

Scheme S7. Copper-catalyzed borylation of acyl chlorides with B_2pin_2 to synthesize TIMs.

General procedure-2 for the synthesis of trifluoroborate iminiums (TIMs).

In an argon-filled glovebox, B_2pin_2 (1.0 equiv., 0.3 mmol, 77 mg), LiO*t*-Bu (2.0 equiv., 0.6

mmol, 48 mg), [(ICy)CuCl] (5 mol%, 5 mg) and toluene (2 mL) were added to a 20 mL thick-walled reaction tube equipped with a magnetic stirring bar. The reaction tube was sealed with a crimped septum cap, removed from the glovebox and stirred at room temperature for 0.5 h. The acyl chloride (1.1 equiv., 0.33 mmol) dissolved in 2 mL of toluene was added dropwise to the tube via syringe under an argon atmosphere. The reaction mixture was stirred at room temperature for 6 h. Upon completion, aqueous KHF_2 (2.7 M, 2.7 mmol in 1.0 mL of H_2O, 9.0 equiv.) was added at room temperature under argon, followed by addition of THF (4 mL), and the reaction was stirred at room temperature for another 24 h. The resulting mixture was opened to air and the reaction mixture was concentrated under reduced pressure. Acetone was added to dissolve the organic solid residue and the solution was filtered through a pad of celite. The filtrate was concentrated under reduced pressure and the resulting solid was washed with Et_2O and dried *in vacuo* to afford the corresponding KATs. Then, MeCN (4 mL) was added to the KAT product, followed by addition of AcOH (1.2 equiv.) and pyrrolidine (1.2 equiv.). The reaction mixture was stirred at room temperature for 12 h. The solvent was evaporated and the trifluoroborate iminium (TIM) compound was purified by silica gel chromatography (DCM/ethyl acetate = 1/1 (v/v) to ethyl acetate gradient).

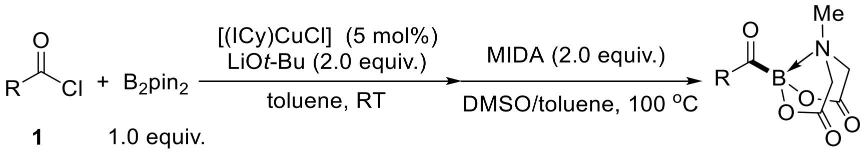

Scheme S8. Copper-catalyzed borylation of acyl chlorides with B_2pin_2 to synthesize acyl MIDA boronates.

General procedure-3 for the synthesis of acyl MIDA boronates. In an argon-filled glovebox, B_2pin_2 (1.0 equiv., 0.3 mmol, 77 mg), LiO*t*-Bu (2.0 equiv., 0.6 mmol, 48 mg), [(ICy)CuCl] (5 mol%, 5 mg) and toluene (2 mL) were added to a 20 mL thick-walled reaction tube equipped with a magnetic stirring bar. The reaction tube was sealed with a crimped septum cap, removed from the glovebox and stirred at room temperature for 0.5 h. The acyl chloride (1.1 equiv., 0.33 mmol) dissolved in 2 mL of toluene was added

dropwise to the tube via syringe under an argon atmosphere. The reaction mixture was stirred at room temperature for 6 h. The reaction tube was returned to the glovebox. N-Methyliminodiacetic acid (90 mg, 0.6 mmol, 2.0 equiv.) and DMSO (4 mL) were added to the reaction mixture at room temperature. The reaction tube was removed from the glovebox and heated at 100 °C for 18 h. Upon completion, the reaction mixture was concentrated *in vacuo* and the crude residue was purified by silica gel chromatography (DCM/ethyl acetate = 1/1 (v/v) to ethyl acetate gradient) to afford the corresponding acyl MIDA boronates.

Compound data:

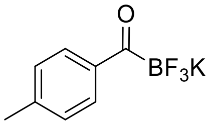

2a

2a was synthesized according to general procedure-1 and obtained as a white solid (56 mg, 0.25 mmol, 82%).

^{1}H NMR (300 MHz, *d_6*-acetone): δ 7.98 (d, *J* = 7.8 Hz, 2H, Ar–*H*), 7.18 (d, *J* = 7.8 Hz, 2H, Ar–*H*), 2.34 (s, 3H, Me–*H*) ppm.

^{11}B{^{1}H} NMR (96 MHz, *d_6*-acetone): δ –0.80 (q, *J* = 52 Hz) ppm.

^{13}C{^{1}H} NMR (126 MHz, *d_6*-acetone): δ 141.9, 139.9 (br), 129.4 (q, *J* = 2 Hz), 129.1, 21.5 ppm. The carbonyl carbon directly attached to boron was not detected in the ^{13}C NMR spectrum, likely due to quadrupolar broadening.

^{19}F{^{1}H} NMR (471 MHz, *d_6*-acetone): δ –144.5 (q, *J* = 52 Hz) ppm.

The NMR spectra are consistent with data previously reported. Reference: A. M. Dumas, J. W. Bode, *Org. Lett.* **2012**, *14*, 2138–2141.

HRMS (ESI): m/z calcd for $C_8H_7BF_3O$ [M–K]$^-$: 187.0548. Found: 187.0538.

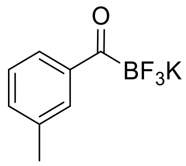

2b

2b was synthesized according to general procedure-1 and obtained as a white solid (54 mg, 0.24 mmol, 80%).

^{1}H NMR (500 MHz, *d_6*-acetone): δ 7.88 (m, 2H, Ar–*H*), 7.28 – 7.22 (m, 2H, Ar–*H*), 2.34 (s, 3H, Me–*H*) ppm.

^{11}B{^{1}H} NMR (160 MHz, *d_6*-acetone): δ –0.84 (q, *J* = 53 Hz) ppm.

^{13}C{^{1}H} NMR (126 MHz, *d_6*-acetone): δ 142.5 (br), 137.8, 132.4, 129.7 (q, *J* = 2 Hz), 128.4, 126.6 (q, *J* = 2 Hz), 21.5 ppm. The carbonyl carbon directly attached to boron was not detected in the ^{13}C NMR spectrum, likely due to quadrupolar broadening.

^{19}F{^{1}H} NMR (471 MHz, *d_6*-acetone): δ –144.5 (q, *J* = 53 Hz) ppm.

The NMR spectra are consistent with data previously reported. Reference: D. Wu, N. A. Fohn, J. W. Bode, *Angew. Chem. Int. Ed.* **2019**, *58*, 11058–11062; *Angew. Chem.* **2019**, *131*, 11174–11178.

HRMS (ESI): m/z calcd for $C_8H_7BF_3O$ [M–K]$^-$: 187.0548. Found: 187.0539.

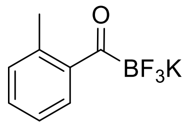

2c

2c was synthesized according to general procedure-1 and obtained as a white solid (51 mg, 0.23 mmol, 76%).

^{1}H NMR (500 MHz, *d_6*-acetone): δ 8.02 (d, *J* = 7.0 Hz, 1H, Ar–*H*), 7.23 – 7.16 (m, 2H, Ar–*H*), 7.11 – 7.09 (m, 1H, Ar–*H*), 2.39 (s, 3H, Me–*H*) ppm.

^{11}B{^{1}H} NMR (160 MHz, *d_6*-acetone): δ –1.17 (q, *J* = 52 Hz) ppm.

^{13}C{^{1}H} NMR (126 MHz, *d_6*-acetone): δ 143.7 (br), 136.5, 131.7 (q, *J* = 3 Hz), 131.6, 129.9, 125.7, 21.1 ppm. The carbonyl carbon directly attached to boron was not detected in the ^{13}C NMR spectrum, likely due to quadrupolar broadening.

^{19}F{^{1}H} NMR (471 MHz, d_6-acetone): δ –145.6 (q, J = 52 Hz) ppm.

The NMR spectra are consistent with data previously reported. Reference: A. M. Dumas, J. W. Bode, *Org. Lett.* **2012**, *14*, 2138–2141.

HRMS (ESI): m/z calcd for $C_8H_7BF_3O$ [M–K]$^-$: 187.0548. Found: 187.0538.

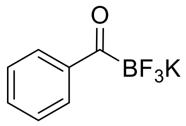

2d

2d was synthesized according to general procedure-1 and obtained as a white solid (53 mg, 0.25 mmol, 84%).

^{1}H NMR (500 MHz, d_6-acetone): δ 8.09 – 8.07 (m, 2H, Ar–*H*), 7.44 – 7.41 (m, 1H, Ar–*H*), 7.40 – 7.36 (m, 2H, Ar–*H*) ppm.

^{11}B{^{1}H} NMR (160 MHz, d_6-acetone): δ –0.80 (q, J = 52 Hz) ppm.

^{13}C{^{1}H} NMR (126 MHz, d_6-acetone): δ 142.2 (br), 131.8, 129.2 (q, J = 2 Hz), 128.5 ppm. The carbonyl carbon directly attached to boron was not detected in the ^{13}C NMR spectrum, likely due to quadrupolar broadening.

^{19}F{^{1}H} NMR (471 MHz, d_6-acetone): δ –144.6 (q, J = 52 Hz) ppm.

The NMR spectra are consistent with data previously reported. Reference: A. M. Dumas, J. W. Bode, *Org. Lett.* **2012**, *14*, 2138–2141.

HRMS (ESI): m/z calcd for $C_7H_5BF_3O$ [M–K]$^-$: 173.0391. Found: 173.0380.

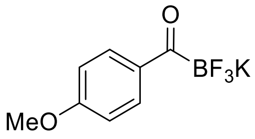

2e

2e was synthesized according to general procedure-1 and obtained as a white solid (62 mg, 0.26 mmol, 85%).

^{1}H NMR (500 MHz, d_6-acetone): δ 8.07 (d, J = 8.5 Hz, 2H, Ar–*H*), 6.90 (d, J = 8.5 Hz, 2H, Ar–*H*), 3.83 (s, 3H, OMe–*H*) ppm.

^{11}B{^{1}H} NMR (160 MHz, d_6-acetone): δ –0.78 (q, J = 50 Hz) ppm.

^{13}C{^{1}H} NMR (126 MHz, d_6-acetone): δ 163.0, 136.0 (br), 131.4 (q, J = 2 Hz), 113.6, 55.6 ppm. The carbonyl carbon directly attached to boron was not detected in the ^{13}C NMR spectrum, likely due to quadrupolar broadening.

^{19}F{^{1}H} NMR (471 MHz, d_6-acetone): δ –144.3 (q, J = 50 Hz) ppm.

The NMR spectra are consistent with data previously reported. Reference: G. Erös, Y. Kushida, J. W. Bode, *Angew. Chem., Int. Ed.* **2014**, *53*, 7604–7607; *Angew. Chem.* **2014**, *126*, 7734–7737.

HRMS (ESI): m/z calcd for $C_8H_7BF_3O_2$ [M–K]$^-$: 203.0497. Found: 203.0488.

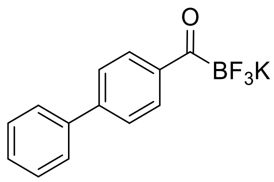

2f

2f was synthesized according to general procedure-1 and obtained as a white solid (66 mg, 0.23 mmol, 78%).

^{1}H NMR (500 MHz, d_6-acetone): δ 8.17 (d, J = 8 Hz, 2H, Ar–*H*), 7.71 – 7.67 (m, 4H, Ar–*H*), 7.49 – 7.46 (m, 2H, Ar–*H*), 7.39 – 7.36 (m, 1H, Ar–*H*) ppm.

^{11}B{^{1}H} NMR (160 MHz, d_6-acetone): δ –0.78 (q, J = 54 Hz) ppm.

^{13}C{^{1}H} NMR (126 MHz, d_6-acetone): δ 144.2, 141.7, 129.9 (q, J = 2 Hz), 129.8, 129.5, 127.9, 127.2 ppm. The carbonyl carbon directly attached to boron was not detected in the ^{13}C NMR spectrum, likely due to quadrupolar broadening.

^{19}F{^{1}H} NMR (471 MHz, d_6-acetone): δ –144.6 (q, J = 54 Hz) ppm.

The NMR spectra are consistent with data previously reported. Reference: A. Schuhmacher, S. J. Ryan, J. W. Bode, *Angew. Chem., Int. Ed.* **2021**, *60*, 3918–3922; *Angew. Chem.* **2021**, *133*, 3964–3968.

HRMS (ESI): m/z calcd for $C_{13}H_9BF_3O$ [M–K]$^-$: 249.0704. Found: 249.0700.

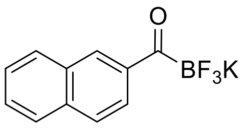

2g

2g was synthesized according to general procedure-1 and obtained as a white solid (55 mg, 0.21 mmol, 70%).

^{1}H NMR (500 MHz, d_6-acetone): δ 8.79 (s, 1H, Ar–*H*), 8.06 (dd, *J* = 8.5, 1.5 Hz, 1H, Ar–*H*), 8.02 – 8.01 (m, 1H, Ar–*H*), 7.90 – 7.88 (m, 1H, Ar–*H*), 7.85 (d, *J* = 8.5 Hz, 1H, Ar–*H*), 7.56 – 7.50 (m, 2H, Ar–*H*) ppm.

^{11}B{^{1}H} NMR (160 MHz, d_6-acetone): δ –0.69 (q, *J* = 51 Hz) ppm.

^{13}C{^{1}H} NMR (126 MHz, d_6-acetone): δ 139.7 (br), 136.0, 134.2, 132.0 (q, *J* = 3 Hz), 130.4, 128.5, 128.1, 128.0, 126.8, 124.9 ppm. The carbonyl carbon directly attached to boron was not detected in the ^{13}C NMR spectrum, likely due to quadrupolar broadening.

^{19}F{^{1}H} NMR (471 MHz, d_6-acetone): δ –144.3 (q, *J* = 51 Hz) ppm.

The NMR spectra are consistent with data previously reported. Reference: P. Tung, A. Schuhmacher, P. E. Schilling, J. W. Bode, N. P. Mankad, *Angew. Chem. Int. Ed.* **2022**, *61*, e202114513.

HRMS (ESI): m/z calcd for $C_{11}H_7BF_3O$ [M–K]$^-$: 223.0548. Found: 223.0542.

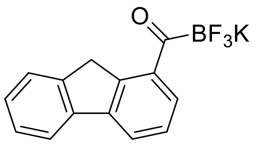

2h

2h was synthesized according to general procedure-1 and obtained as a light-yellow solid (60 mg, 0.20 mmol, 65%).

m.p.: 304 ^{o}C (decomp).

^{1}H NMR (500 MHz, d_6-acetone): δ 8.41 (d, *J* = 7.5, 1H, Ar–*H*), 7.94 (dd, *J* = 7.5, 1.5 Hz, 1H, Ar–*H*), 7.86 (d, *J* = 7.5, 1H, Ar–*H*), 7.54 (dq, *J* = 7.5, 1.0 Hz, 1H, Ar–*H*), 7.48 (tt, *J* = 8.0, 1.0 Hz, 1H, Ar–*H*), 7.35 (m, 1H, Ar–*H*), 7.28 (td, *J* = 7.5, 1.5 Hz, 1H, Ar–*H*), 4.18 (s, 2H, C*H$_2$*) ppm.

^{11}B{^{1}H} NMR (160 MHz, *d_6*-acetone): δ –0.91 (q, *J* = 54 Hz) ppm.

^{13}C{^{1}H} NMR (126 MHz, *d_6*-acetone): δ 145.4, 143.0, 142.1, 141.8, 138.8 (br), 132.3 (q, *J* = 3 Hz), 127.5, 127.3, 125.7, 122.9, 120.5, 39.1 ppm. The carbonyl carbon directly attached to boron was not detected in the ^{13}C NMR spectrum, likely due to quadrupolar broadening.

^{19}F{^{1}H} NMR (471 MHz, *d_6*-acetone): δ –144.7 (q, *J* = 54 Hz) ppm.

IR (ν/cm^{-1}, neat): 1624, 1570, 1048, 1011, 983, 931, 751, 711, 627.

HRMS (ESI): m/z calcd for $C_{14}H_9BF_3O$ [M–K]$^-$: 261.0704. Found: 261.0703.

Elem. Anal.: Calc. (%) for $C_{14}H_9BF_3KO$: C 56.03, H 3.02. Found: C 55.87, H 2.97.

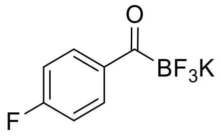

2i

2i was synthesized according to general procedure-1 and obtained as a white solid (59 mg, 0.26 mmol, 85%).

^{1}H NMR (500 MHz, *d_6*-acetone): δ 8.13 (m, 2H, Ar–*H*), 7.11 (m, 2H, Ar–*H*) ppm.

^{11}B {^{1}H} NMR (160 MHz, *d_6*-acetone): δ –0.87 (q, *J* = 53 Hz) ppm.

^{13}C {^{1}H} NMR (126 MHz, *d_6*-acetone): δ 166.4 (d, *J* = 249 Hz), 139.1 (br), 131.8 (dq, J^1 = 9 Hz, J^2 = 2 Hz,), 115.2 (d, *J* = 21 Hz) ppm. The carbonyl carbon directly attached to boron was not detected in the ^{13}C NMR spectrum, likely due to quadrupolar broadening.

^{19}F{^{1}H} NMR (471 MHz, *d_6*-acetone): δ –111.5, –144.7 (q, *J* = 53 Hz) ppm.

The NMR spectra are consistent with data previously reported. Reference: H. Noda, G. Erös, J. W. Bode, *J. Am. Chem. Soc.* **2014**, *136*, 5611–5614.

HRMS (ESI): m/z calcd for $C_7H_4BF_4O$ [M–K]$^-$: 191.0297. Found: 191.0287.

Elem. Anal.: Calc. (%) for $C_7H_4BF_4KO$: C 36.55, H 1.75. Found: C 36.27, H 1.70.

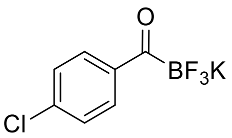

2j

2j was synthesized according to general procedure-1 and was obtained as a white solid (62 mg, 0.25 mmol, 83%).

^{1}H NMR (300 MHz, d_6-DMSO): δ 7.91 (d, J = 8.5 Hz, 2H, Ar–H), 7.45 (d, J = 8.5 Hz, 2H, Ar–H) ppm.

^{11}B{^{1}H} NMR (96 MHz, d_6-acetone): δ –0.92 (q, J = 52 Hz) ppm.

^{13}C{^{1}H} NMR (126 MHz, d_6-DMSO): δ 139.6 (br), 135.6, 129.6 (q, J = 2 Hz), 128.0 ppm. The carbonyl carbon directly attached to boron was not detected in the ^{13}C NMR spectrum, likely due to quadrupolar broadening.

^{19}F{^{1}H} NMR (471 MHz, d_6-DMSO): δ –144.8 (q, J = 52 Hz) ppm.

The NMR spectra are consistent with data previously reported. Reference: A. M. Dumas, J. W. Bode, *Org. Lett.* **2012**, *14*, 2138–2141.

HRMS (ESI): m/z calcd for $C_7H_4ClBF_3O$ [M–K]$^-$: 207.0001. Found: 206.9993.

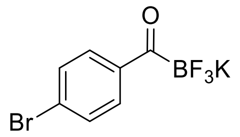

2k

2k was synthesized according to general procedure-1 and obtained as a white solid (66 mg, 0.23 mmol, 76%).

^{1}H NMR (500 MHz, d_6-acetone): δ 7.98 (d, J = 8.5 Hz, 2H, Ar–H), 7.57 (d, J = 8.5 Hz, 2H, Ar–H) ppm.

^{11}B{^{1}H} NMR (160 MHz, d_6-acetone): δ –0.92 (q, J = 51 Hz) ppm.

^{13}C{^{1}H} NMR (126 MHz, d_6-acetone): δ 141.2 (br), 131.8, 131.2 (q, J = 2 Hz), 126.0 ppm. The carbonyl carbon directly attached to boron was not detected in the ^{13}C NMR spectrum, likely due to quadrupolar broadening.

^{19}F{^{1}H} NMR (471 MHz, d_6-acetone): δ –144.8 (q, J = 51 Hz) ppm.

The NMR spectra are consistent with data previously reported. Reference: G. Erös, Y. Kushida, J. W. Bode, *Angew. Chem., Int. Ed.* **2014**, *53*, 7604–7607; *Angew. Chem.* **2014**, *126*, 7734–7737.

HRMS (ESI): m/z calcd for $C_7H_4BrBF_3O$ [M–K]$^-$: 250.9496. Found: 250.9495.

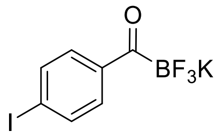

2l

2l was synthesized according to general procedure-1 and obtained as a white solid (65 mg, 0.19 mmol, 64%).

^{1}H NMR (500 MHz, d_6-acetone): δ 7.84 (d, *J* = 8.5 Hz, 2H, Ar–*H*), 7.79 (d, *J* = 8.5 Hz, 2H, Ar–*H*) ppm.

^{11}B{^{1}H} NMR (160 MHz, d_6-acetone): δ –0.93 (q, *J* = 52 Hz) ppm.

^{13}C{^{1}H} NMR (126 MHz, d_6-acetone): δ 137.9, 131.2 (q, *J* = 2 Hz), 98.7 ppm. The carbonyl carbon directly attached to boron was not detected in the ^{13}C NMR spectrum, likely due to quadrupolar broadening.

^{19}F{^{1}H} NMR (471 MHz, d_6-acetone): δ –144.8 (q, *J* = 52 Hz) ppm.

The NMR spectra are consistent with data previously reported. Reference: G. Erös, Y. Kushida, J. W. Bode, *Angew. Chem., Int. Ed.* **2014**, *53*, 7604–7607; *Angew. Chem.* **2014**, *126*, 7734–7737.

HRMS (ESI): m/z calcd for $C_7H_4IBF_3O$ [M–K]$^-$: 298.9357. Found: 298.9356.

Elem. Anal.: Calc. (%) for $C_7H_4IBF_3KO$: C 24.88, H 1.19. Found: C 24.79, H 1.19.

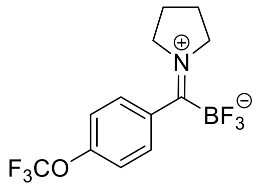

2m

2m was synthesized according to general procedure-2 and obtained as a colorless oil (65 mg, 0.21 mmol, 70%).

^{1}H NMR (500 MHz, d_6-acetone): δ 7.53 – 7.51 (m, 2H, Ar–*H*), 7.48 – 7.46 (m, 2H, Ar–*H*), 4.32 (t, *J* = 7.5 Hz, 2H, C*H$_2$*), 3.85 (t, *J* = 7.0 Hz, 2H, C*H$_2$*), 2.20 (quint, *J* = 7.5 Hz, 2H, C*H$_2$*), 2.08 (quint, *J* = 7.0 Hz, 2H, C*H$_2$*) ppm.

^{11}B{^{1}H} NMR (160 MHz, d_6-acetone): δ –0.39 (q, *J* = 39 Hz) ppm.

$^{13}C\{^1H\}$ NMR (126 MHz, d_6-acetone): δ 150.6, 138.0, 128.5 121.8, 121.4 (q, J = 257 Hz), 56.8, 56.7, 25.2, 24.8 ppm. The carbonyl carbon directly attached to boron was not detected in the ^{13}C NMR spectrum, likely due to quadrupolar broadening.

$^{19}F\{^1H\}$ NMR (471 MHz, d_6-acetone): δ –58.5, –143.6 (q, J = 39 Hz) ppm.

IR (ν/cm^{-1}, neat): 1707, 1622, 1510, 1250, 1160, 1011.

HRMS (ASAP): m/z calcd for $C_{12}H_{12}BF_6NONa$ $[M+Na]^+$: 334.0808. Found: 334.0802.

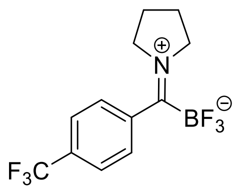

2n

2n was synthesized according to general procedure-2 and obtained as a colorless oil (67 mg, 0.23 mmol, 76%).

^1H NMR (500 MHz, d_6-acetone): δ 7.86 (d, J = 8.5 Hz, 2H, Ar–H), 7.57 (d, J = 8.5 Hz, 2H, Ar–H), 4.34 (t, J = 7.0 Hz, 2H, CH_2), 3.82 (t, J = 7.5 Hz, 2H, CH_2), 2.22 (quint, J = 7.0 Hz, 2H, CH_2), 2.09 (quint, J = 7.5 Hz, 2H, CH_2) ppm.

$^{11}B\{^1H\}$ NMR (160 MHz, d_6-acetone): δ –0.40 (q, J = 38 Hz) ppm.

$^{13}C\{^1H\}$ NMR (126 MHz, d_6-acetone): δ 143.1, 131.5 (q, J = 33 Hz), 126.3 (q, J = 4 Hz), 125.1 (q, J = 272 Hz), 56.9, 25.3, 24.7 ppm. The carbonyl carbon directly attached to boron was not detected in the ^{13}C NMR spectrum, likely due to quadrupolar broadening.

$^{19}F\{^1H\}$ NMR (471 MHz, d_6-acetone): δ –63.4, –143.7 (q, J = 38 Hz) ppm.

The NMR spectra are consistent with data previously reported. Reference: M. K. Jackl, A. Schuhmacher, T. Shiro, J. W. Bode, *Org. Lett.* **2018**, *20*, 4044–4047.

HRMS (ASAP): m/z calcd for $C_{12}H_{12}BF_6NNa$ $[M+Na]^+$: 318.0859. Found: 318.0854.

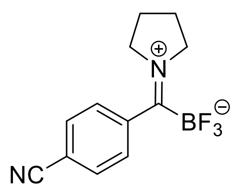

2o

2o was synthesized according to general procedure-2 and obtained as a colorless oil (54 mg, 0.22 mmol, 72%).

^{1}H NMR (500 MHz, d_6-acetone): δ 7.92 (d, *J* = 8.5 Hz, 2H, Ar–*H*), 7.55 (d, *J* = 8.5 Hz, 2H, Ar–*H*), 4.33 (t, *J* = 7.5 Hz, 2H, C*H$_2$*), 3.82 (t, *J* = 7.0 Hz, 2H, C*H$_2$*), 2.22 (quint, *J* = 7.0 Hz, 2H, C*H$_2$*), 2.09 (quint, *J* = 7.5 Hz, 2H, C*H$_2$*) ppm.

^{11}B{^{1}H} NMR (160 MHz, d_6-acetone): δ –0.44 (q, *J* = 38 Hz) ppm.

^{13}C{^{1}H} NMR (126 MHz, d_6-acetone): δ 143.4, 133.2, 126.6, 118.8, 113.8, 56.8, 25.2, 24.7 ppm. The carbonyl carbon directly attached to boron was not detected in the ^{13}C NMR spectrum, likely due to quadrupolar broadening.

^{19}F{^{1}H} NMR (471 MHz, d_6-acetone): δ –143.6 (q, *J* = 38 Hz) ppm.

The NMR spectra are consistent with data previously reported. Reference: M. K. Jackl, A. Schuhmacher, T. Shiro, J. W. Bode, *Org. Lett.* **2018**, *20*, 4044–4047.

HRMS (ESI): m/z calcd for $C_{12}H_{12}BF_3N_2Na$ [M+Na]$^+$: 275.0938. Found: 275.0934.

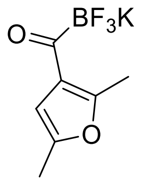

2p

2p was synthesized according to general procedure-1 and obtained as a yellow solid (47 mg, 0.20 mmol, 68%).

m.p.: 240 ^{o}C (decomp).

^{1}H NMR (500 MHz, d_6-acetone): δ 6.45 (s, 1H, Ar–*H*), 2.46 (s, 3H, Me–*H*), 2.17 (s, 3H, Me–*H*) ppm.

^{11}B{^{1}H} NMR (160 MHz, d_6-acetone): δ –1.21 (q, *J* = 53 Hz) ppm.

^{13}C{^{1}H} NMR (126 MHz, d_6-acetone): δ 154.3, 148.9, 127.2 (br), 108.4 (q, *J* = 2 Hz), 14.0, 13.2 ppm. The carbonyl carbon directly attached to boron was not detected in the ^{13}C NMR spectrum, likely due to quadrupolar broadening.

^{19}F{^{1}H} NMR (471 MHz, d_6-acetone): δ –147.6 (q, *J* = 53 Hz) ppm.

IR (ν/cm^{-1}, neat): 1619, 1560, 1013, 928, 866, 711.

HRMS (ESI): m/z calcd for $C_7H_7BF_3O_2$ [M–K]$^-$: 191.0497. Found: 191.0489.

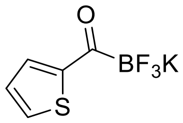

2q

2q was synthesized according to general procedure-1 and obtained as a yellow solid (46 mg, 0.21 mmol, 71%).

^{1}H NMR (500 MHz, d_6-acetone): δ 8.02 (m, 1H, Ar–*H*), 7.58 (dd, *J* = 5.0, 1.0 Hz, 1H, Ar–*H*), 7.11 (dd, *J* = 5.0, 4.0 Hz, 1H, Ar–*H*) ppm.

^{11}B{^{1}H} NMR (160 MHz, d_6-acetone): δ –1.04 (q, *J* = 51 Hz) ppm.

^{13}C{^{1}H} NMR (126 MHz, d_6-acetone): δ 134.2 (q, *J* = 3 Hz), 131.0, 128.7 ppm. One thienyl carbon cannot be observed in ^{13}C{^{1}H} NMR but can be extracted in ^{13}C ^{1}H HMBC spectrum (δ = 151.5 ppm). The carbonyl carbon directly attached to boron was not detected in the ^{13}C NMR spectrum, likely due to quadrupolar broadening.

^{19}F{^{1}H} NMR (471 MHz, d_6-acetone): δ –146.4 (q, *J* = 51 Hz) ppm.

The NMR spectra are consistent with data previously reported. Reference: G. Erös, Y. Kushida, J. W. Bode, *Angew. Chem., Int. Ed.* **2014**, *53*, 7604–7607; *Angew. Chem.* **2014**, *126*, 7734–7737.

HRMS (ESI): m/z calcd for $C_5H_3BF_3OS$ $[M–K]^–$: 178.9955. Found: 178.9947.

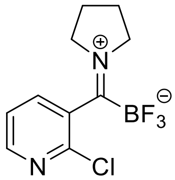

2r

2r was synthesized according to general procedure-2 and obtained as a light-yellow oil (53 mg, 0.20 mmol, 67%).

^{1}H NMR (500 MHz, $CDCl_3$): δ 8.48 (d, *J* = 3 Hz, 1H, Ar–*H*), 7.60 (d, *J* = 8 Hz, 1H, Ar–*H*), 7.39 (dd, *J* = 8, 3 Hz, 1H, Ar–*H*), 4.46 (m, 2H, C*H$_2$*), 3.76 (m, 1H, C*H$_2$*), 3.51 (m, 1H, C*H$_2$*), 2.25 (m, 2H, C*H$_2$*), 2.12 (m, 2H, C*H$_2$*) ppm.

^{11}B{^{1}H} NMR (160 MHz, $CDCl_3$): δ –0.56 (q, *J* = 38 Hz) ppm.

$^{13}C\{^1H\}$ NMR (126 MHz, $CDCl_3$): δ 150.8, 144.3, 135.0, 132.8, 122.7, 56.3, 55.9, 24.8, 24.1 ppm. The carbonyl carbon directly attached to boron was not detected in the ^{13}C NMR spectrum, likely due to quadrupolar broadening.

$^{19}F\{^1H\}$ NMR (471 MHz, $CDCl_3$): δ –145.3 (q, *J* = 38 Hz) ppm.

IR (v/cm^{-1}, neat): 2952, 1639, 1560, 1397, 1081, 1013, 968.

HRMS (ESI): m/z calcd for $C_{10}H_{11}BClF_3N_2Na$ $[M+Na]^+$: 285.0548. Found: 285.0542.

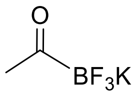

4a

4a was synthesized according to general procedure-1 and obtained as a white solid (30 mg, 0.20 mmol, 68%).

^1H NMR (500 MHz, d_6-acetone): δ 1.93 (s, 3H, Me–*H*) ppm.

$^{11}B\{^1H\}$ NMR (160 MHz, d_6-acetone): δ –1.76 (q, *J* = 54 Hz) ppm.

$^{13}C\{^1H\}$ NMR (126 MHz, d_6-acetone): δ 32.2 (br) ppm. The carbonyl carbon directly attached to boron was not detected in the ^{13}C NMR spectrum, likely due to quadrupolar broadening.

$^{19}F\{^1H\}$ NMR (471 MHz, d_6-acetone): δ –151.4 (q, *J* = 54 Hz) ppm.

The NMR spectra are consistent with data previously reported. Reference: S. M. Liu, D. Wu, J. W. Bode, *Org. Lett.* **2018**, *20*, 2378–2381.

HRMS (ESI): m/z calcd for $C_2H_3BF_3O$ $[M–K]^–$: 111.0235. Found: 111.0220.

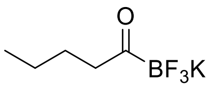

4b

4b was synthesized according to general procedure-1 and obtained as a white solid (40 mg, 0.21 mmol, 72%).

^1H NMR (500 MHz, d_6-acetone): δ 2.38 (t, *J* = 7.5 Hz, 2H, CH_2), 1.41 (quint, *J* = 7.5 Hz, 2H, CH_2), 1.22 (sext, *J* = 7.5 Hz, 2H, CH_2), 0.85 (t, *J* = 7.5 Hz, 3H, CH_3) ppm.

$^{11}B\{^1H\}$ NMR (160 MHz, d_6-acetone): δ –1.68 (q, *J* = 54 Hz) ppm.

$^{13}C\{^1H\}$ NMR (126 MHz, d_6-acetone): δ 45.0 (br), 25.5, 23.6, 14.5 ppm. The carbonyl carbon directly attached to boron was not detected in the ^{13}C NMR spectrum, likely due to quadrupolar broadening.

$^{19}F\{^1H\}$ NMR (471 MHz, d_6-acetone): δ –150.8 (q, J = 54 Hz) ppm.

The NMR spectra are consistent with data previously reported. Reference: S. M. Liu, D. Wu, J. W. Bode, *Org. Lett.* **2018**, *20*, 2378–2381.

HRMS (ESI): m/z calcd for $C_5H_9BF_3O$ $[M–K]^–$: 153.0704. Found: 153.0693.

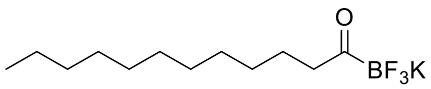

4c

4c was synthesized according to general procedure-1 and obtained as a white solid (70 mg, 0.24 mmol, 79%).

m.p.: 220 °C (decomp).

^1H NMR (300 MHz, d_6-acetone): δ 2.38 (t, J = 7.5 Hz, 2H, C*H₂*), 1.43 (m, 2H, C*H₂*), 1.28 (br, 16H, C*H₂*), 0.88 (t, J = 7.2 Hz, 3H, C*H₃*) ppm.

$^{11}B\{^1H\}$ NMR (160 MHz, d_6-acetone): δ –1.67 (q, J = 55 Hz) ppm.

$^{13}C\{^1H\}$ NMR (126 MHz, d_6-acetone): δ 45.3 (br), 32.7, 32.6, 23.4, 23.3, 14.4 ppm. The carbonyl carbon directly attached to boron was not detected in the ^{13}C NMR spectrum, likely due to quadrupolar broadening.

$^{19}F\{^1H\}$ NMR (471 MHz, d_6-acetone): δ –150.8 (q, J = 55 Hz) ppm.

IR (ν/cm^{-1}, neat): 2917, 2854, 1652, 1470, 1056, 913.

HRMS (ESI): m/z calcd for $C_{12}H_{23}BF_3O$ $[M–K]^–$: 251.1800. Found: 251.1796.

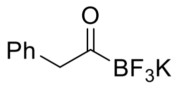

4d

4d was synthesized according to general procedure-1 and obtained as a white solid (54 mg, 0.24 mmol, 82%).

^1H NMR (500 MHz, d_6-acetone): δ 7.22 – 7.19 (m, 2H, Ar–*H*), 7.13 – 7.10 (m, 1H, Ar–*H*), 7.08 – 7.06 (m, 2H, Ar–*H*), 3.72 (s, 2H, C*H₂*) ppm.

^{11}**B{^{1}H} NMR** (160 MHz, *d$_6$*-acetone): δ –1.55 (q, *J* = 54 Hz) ppm.

^{13}**C{^{1}H} NMR** (126 MHz, *d$_6$*-acetone): δ 138.0, 130.9, 128.4, 126.1, 51.6 (br) ppm. The carbonyl carbon directly attached to boron was not detected in the ^{13}C NMR spectrum, likely due to quadrupolar broadening.

^{19}**F{^{1}H} NMR** (471 MHz, *d$_6$*-acetone): δ –150.9 (q, *J* = 54 Hz) ppm.

The NMR spectra are consistent with data previously reported. Reference: G. A. Molander, J. Raushel, N. M. Ellis, *J. Org. Chem.* **2010**, *75*, 4304–4306.

HRMS (ESI): m/z calcd for $C_8H_7BF_3O$ [M–K]$^-$: 187.0548. Found: 187.0538.

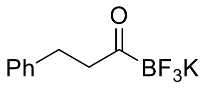

4e

4e was synthesized according to general procedure-1 and obtained as a white solid (60 mg, 0.25 mmol, 83%).

^{1}**H NMR** (500 MHz, *d$_6$*-acetone): δ 7.23 – 7.19 (m, 2H, Ar–*H*), 7.17 – 7.16 (m, 2H, Ar–*H*), 7.12 – 7.09 (m, 1H, Ar–*H*), 2.74 (s, 4H, C*H$_2$*) ppm.

^{11}**B{^{1}H} NMR** (160 MHz, *d$_6$*-acetone): δ –1.59 (q, *J* = 54 Hz) ppm.

^{13}**C{^{1}H} NMR** (126 MHz, *d$_6$*-acetone): δ 144.2, 129.0, 128.9, 126.1, 47.0 (br), 29.2 ppm. The carbonyl carbon directly attached to boron was not detected in the ^{13}C NMR spectrum, likely due to quadrupolar broadening.

^{19}**F{^{1}H} NMR** (471 MHz, *d$_6$*-acetone): δ –150.8 (q, *J* = 54 Hz) ppm.

The NMR spectra are consistent with data previously reported. Reference: G. A. Molander, J. Raushel, N. M. Ellis, *J. Org. Chem.* **2010**, *75*, 4304–4306.

HRMS (ESI): m/z calcd for $C_9H_9BF_3O$ [M–K]$^-$: 201.0704. Found: 201.0696.

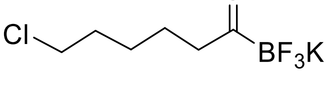

4f

4f was synthesized according to general procedure-1 and obtained as a white solid (58 mg, 0.24 mmol, 81%).

^{1}H NMR (500 MHz, d_6-acetone): δ 3.57 (t, *J* = 7 Hz, 2H, CH_2), 2.39 (t, *J* = 7 Hz, 2H, CH_2), 1.73 (quint, *J* = 7 Hz, 2H, CH_2), 1.47 (quint, *J* = 7 Hz, 2H, CH_2), 1.36 (quint, *J* = 7 Hz, 2H, CH_2) ppm.

^{11}B{^{1}H} NMR (160 MHz, d_6-acetone): δ –1.67 (q, *J* = 53 Hz) ppm.

^{13}C{^{1}H} NMR (126 MHz, d_6-acetone): δ 45.7, 44.8 (br), 33.4, 27.6, 22.3 ppm. The carbonyl carbon directly attached to boron was not detected in the ^{13}C NMR spectrum, likely due to quadrupolar broadening.

^{19}F{^{1}H} NMR (471 MHz, d_6-acetone): δ –150.8 (q, *J* = 53 Hz) ppm.

The NMR spectra are consistent with data previously reported. Reference: A. M. Dumas, J. W. Bode, *Org. Lett.* **2012**, *14*, 2138–2141.

HRMS (ESI): m/z calcd for $C_6H_{10}BClF_3O$ $[M–K]^-$: 201.0471. Found: 201.0465.

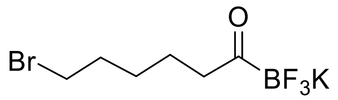

4g

4g was synthesized according to general procedure-1 and obtained as a white solid (71 mg, 0.25 mmol, 84%).

m.p.: 235 °C (decomp).

^{1}H NMR (500 MHz, d_6-acetone): δ 3.47 (t, *J* = 7 Hz, 2H, CH_2), 2.40 (t, *J* = 7 Hz, 2H, CH_2), 1.82 (quint, *J* = 7 Hz, 2H, CH_2), 1.47 (quint, *J* = 7 Hz, 2H, CH_2), 1.36 (quint, *J* = 7 Hz, 2H, CH_2) ppm.

^{11}B{^{1}H} NMR (160 MHz, d_6-acetone): δ –1.67 (q, *J* = 55 Hz) ppm.

^{13}C{^{1}H} NMR (126 MHz, d_6-acetone): δ 44.7 (br), 34.7, 33.6, 28.8, 22.2 ppm. The carbonyl carbon directly attached to boron was not detected in the ^{13}C NMR spectrum, likely due to quadrupolar broadening.

^{19}F{^{1}H} NMR (471 MHz, d_6-acetone): δ –150.8 (q, *J* = 55 Hz) ppm.

IR (ν/cm^{-1}, neat): 2869, 1652, 1577, 1023, 923, 879, 739.

HRMS (ESI): m/z calcd for $C_6H_{10}BF_3OBr$ $[M–K]^-$: 244.9966. Found: 244.9964.

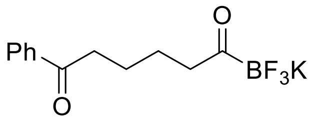

4h

4h was synthesized according to general procedure-1 and obtained as a white solid (77 mg, 0.26 mmol, 85%).

m.p.: 250 ^{o}C (decomp).

^{1}H NMR (500 MHz, d_6-acetone): δ 8.00 (d, J = 7.5 Hz, 2H, Ar–H), 7.59 (t, J = 7.5 Hz, 1H, Ar–H), 7.49 (t, J = 7.5 Hz, 2H, Ar–H), 2.98 (t, J = 7.5 Hz, 2H, CH_2), 2.46 (t, J = 7.5 Hz, 2H, CH_2), 1.62 (quint, J = 7.5 Hz, 2H, CH_2), 1.53 (quint, J = 7.5 Hz, 2H, CH_2) ppm.

^{11}B{^{1}H} NMR (160 MHz, d_6-acetone): δ –1.56 ppm.

^{13}C{^{1}H} NMR (126 MHz, d_6-acetone): δ 200.5, 138.1, 133.5, 129.4, 128.8, 44.9 (br), 39.1, 25.1, 22.9 ppm. The carbonyl carbon directly attached to boron was not detected in the ^{13}C NMR spectrum, likely due to quadrupolar broadening.

^{19}F{^{1}H} NMR (471 MHz, d_6-acetone): δ –150.6 ppm.

IR (ν/cm^{-1}, neat): 2939, 1682, 1587, 1447, 1392, 993.

HRMS (ESI): m/z calcd for $C_{12}H_{13}BF_3O_2$ [M–K]$^{-}$: 257.0966. Found: 257.0966.

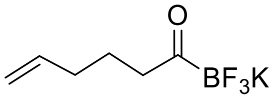

4i

4i was synthesized according to general procedure-1 and obtained as a white solid (24 mg, 0.12 mmol, 40%).

^{1}H NMR (500 MHz, d_6-acetone): δ 5.79 (m, 1H, C=CH), 4.98 – 4.94 (m, 1H, C=CH), 4.90 – 4.87 (m, 1H, C=CH), 2.40 (t, J = 7.5 Hz, 2H, CH_2), 1.97 (q, J = 7.5 Hz, 2H, CH_2), 1.52 (quint, J = 7.5 Hz, 2H, CH_2) ppm.

^{11}B{^{1}H} NMR (160 MHz, d_6-acetone): δ –1.68 (q, J = 55 Hz) ppm.

^{13}C{^{1}H} NMR (126 MHz, d_6-acetone): δ 140.2, 114.6, 44.6 (br), 34.7, 22.8 ppm. The carbonyl carbon directly attached to boron was not detected in the ^{13}C NMR spectrum, likely due to quadrupolar broadening.

^{19}F{^{1}H} NMR (471 MHz, d_6-acetone): δ –150.9 (q, J = 55 Hz) ppm.

The NMR spectra are consistent with data previously reported. Reference: A. M. Dumas, J. W. Bode, *Org. Lett.* **2012**, *14*, 2138–2141.

HRMS (ESI): m/z calcd for $C_6H_9BF_3O$ [M–K]$^-$: 165.0704. Found: 165.0694.

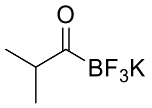

4j

4j was synthesized according to general procedure-1 and obtained as a white solid (34 mg, 0.19 mmol, 62%).

^{1}H NMR (500 MHz, d_6-acetone): δ 2.75 (sept, J = 7 Hz, 1H, CH), 0.90 (d, J = 7 Hz, 6H, Me–H) ppm.

^{11}B{^{1}H} NMR (160 MHz, d_6-acetone): δ –1.54 (q, J = 56 Hz) ppm.

^{13}C{^{1}H} NMR (126 MHz, d_6-acetone): δ 41.6 (br), 17.4 ppm. The carbonyl carbon directly attached to boron was not detected in the ^{13}C NMR spectrum, likely due to quadrupolar broadening.

^{19}F{^{1}H} NMR (471 MHz, d_6-acetone): δ –148.7 (q, J = 56 Hz) ppm.

The NMR spectra are consistent with data previously reported. Reference: S. M. Liu, D. Wu, J. W. Bode, *Org. Lett.* **2018**, *20*, 2378–2381.

HRMS (ESI): m/z calcd for $C_4H_7BF_3O$ [M–K]$^-$: 139.0548. Found: 139.0535.

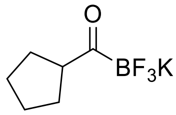

4k

4k was synthesized according to general procedure-1 and obtained as a white solid (39 mg, 0.19 mmol, 66%).

^{1}H NMR (500 MHz, d_6-acetone): δ 3.05 (m, 1H), 1.78 – 1.70 (m, 2H), 1.60 – 1.54 (m, 2H), 1.48 – 1.43 (m, 4H) ppm.

^{11}B{^{1}H} NMR (160 MHz, d_6-acetone): δ –1.51 (q, J = 55 Hz) ppm.

^{13}C{^{1}H} NMR (126 MHz, d_6-acetone): δ 53.1 (br), 27.6, 26.9 ppm. The carbonyl carbon directly attached to boron was not detected in the ^{13}C NMR spectrum, likely due to quadrupolar broadening.

^{19}F{^{1}H} NMR (471 MHz, d_6-acetone): δ –148.9 (q, J = 55 Hz) ppm.

The NMR spectra are consistent with data previously reported. Reference: S. M. Liu, D. Wu, J. W. Bode, *Org. Lett.* **2018**, *20*, 2378–2381.

HRMS (ESI): m/z calcd for $C_6H_9BF_3O$ $[M–K]^–$: 165.0704. Found: 165.0692.

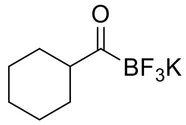

4l

4l was synthesized according to general procedure-1 and obtained as a white solid (39 mg, 0.18 mmol, 59%).

^{1}H NMR (500 MHz, d_6-acetone): δ 2.53 (tt, J = 11.5, 3.5 Hz, 1H), 1.80 (m, 2H), 1.71 – 1.65 (m, 2H), 1.63 – 1.57 (m, 1H), 1.29 – 1.20 (m, 2H), 1.17 – 1.08 (m, 3H) ppm.

^{11}B{^{1}H} NMR (160 MHz, d_6-acetone): δ –1.58 (q, J = 55 Hz) ppm.

^{13}C{^{1}H} NMR (126 MHz, d_6-acetone): δ 52.3 (br), 28.1, 27.4, 27.0 ppm. The carbonyl carbon directly attached to boron was not detected in the ^{13}C NMR spectrum, likely due to quadrupolar broadening.

^{19}F{^{1}H} NMR (471 MHz, d_6-acetone): δ –148.9 (q, J = 55 Hz) ppm.

The NMR spectra are consistent with data previously reported. Reference: S. M. Liu, D. Wu, J. W. Bode, *Org. Lett.* **2018**, *20*, 2378–2381.

HRMS (ESI): m/z calcd for $C_7H_{11}BF_3O$ $[M–K]^–$: 179.0861. Found: 179.0850.

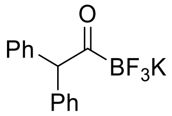

4m

4m was synthesized according to general procedure-1 and obtained as a white solid (54 mg, 0.18 mmol, 61%).

m.p.: 340 ^{o}C (decomp).

^{1}H NMR (500 MHz, d_6-acetone): δ 7.26 – 7.24 (m, 4H, Ar–*H*), 7.21 – 7.18 (m, 4H, Ar–*H*), 7.13 – 7.09 (m, 2H, Ar–*H*), 5.71 (s, 1H, C*H*) ppm.

^{11}B{^{1}H} NMR (160 MHz, d_6-acetone): δ –1.52 (q, J = 53 Hz) ppm.

$^{13}C\{^1H\}$ **NMR** (126 MHz, d_6-acetone): δ 142.1, 130.3, 128.5, 126.4, 63.9 (br) ppm. The carbonyl carbon directly attached to boron was not detected in the ^{13}C NMR spectrum, likely due to quadrupolar broadening.

$^{19}F\{^1H\}$ **NMR** (471 MHz, d_6-acetone): δ –150.2 (q, J = 53 Hz) ppm.

IR (v/cm^{-1}, neat): 1664, 1602, 1495, 1445, 1365, 1006, 956, 739.

HRMS (ESI): m/z calcd for $C_{14}H_{11}BF_3O$ [M–K]$^-$: 263.0861. Found: 263.0858.

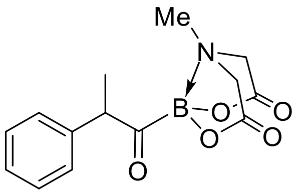

4n

4n was synthesized according to general procedure-3 and obtained as a white solid (42 mg, 0.12 mmol, 41%).

m.p.: 160 ^oC.

^1H **NMR** (300 MHz, $CDCl_3$): δ 7.32 – 7.28 (m, 2H, Ar–*H*), 7.26 – 7.20 (m, 3H, Ar–*H*), 4.29 (q, J = 7.0 Hz, 1H, C*H*), 3.83 – 3.72 (m, 3H, MIDA-CH_2), 3.21 (d, J = 16.5 Hz, 1H, MIDA-CH_2), 2.37 (s, 3H, Me–*H*), 1.37 (d, J = 7.0 Hz, 3H, Me–*H*) ppm.

$^{11}B\{^1H\}$ **NMR** (96 MHz, $CDCl_3$): δ 4.78 ppm.

$^{13}C\{^1H\}$ **NMR** (75 MHz, $CDCl_3$): δ 167.3, 167.1, 137.9, 129.4, 129.0, 127.5, 62.3, 62.1, 55.1, 46.4, 15.4 ppm. The carbonyl carbon directly attached to boron was not detected in the ^{13}C NMR spectrum, likely due to quadrupolar broadening.

IR (v/cm^{-1}, neat): 2979, 1769, 1709, 1662, 1450, 1268, 1053.

HRMS (ESI): m/z calcd for $C_{18}H_{24}BNO_5Na$ [M+Na]$^+$: 368.1640. Found: 368.1635.

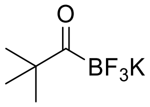

4o

4o was synthesized according to general procedure-1 and obtained as a white solid (40 mg, 0.21 mmol, 71%).

^1H **NMR** (500 MHz, d_6-acetone): δ 1.00 (s, 9H, Me–*H*) ppm.

$^{11}B\{^1H\}$ **NMR** (160 MHz, d_6-acetone): δ –1.40 (q, J = 55 Hz) ppm.

$^{13}C\{^1H\}$ NMR (126 MHz, d_6-acetone): δ 45.1 (br), 25.8 (q, J = 1 Hz) ppm. The carbonyl carbon directly attached to boron was not detected in the ^{13}C NMR spectrum, likely due to quadrupolar broadening.

$^{19}F\{^1H\}$ NMR (471 MHz, d_6-acetone): δ –144.1 (q, J = 55 Hz) ppm.

The NMR spectra are consistent with data previously reported. Reference: S. M. Liu, D. Wu, J. W. Bode, *Org. Lett.* **2018**, *20*, 2378–2381.

HRMS (ESI): m/z calcd for $C_5H_9BF_3O$ $[M–K]^–$: 153.0704. Found: 153.0692.

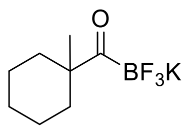

4p

4p was synthesized according to general procedure-1 and obtained as a white solid (53 mg, 0.23 mmol, 76%).

m.p.: 215 ^oC (decomp).

^1H NMR (500 MHz, d_6-acetone): δ 2.14 (m, 2H), 1.44 – 1.31 (m, 5H), 1.23 (m, 1H), 1.09 (m, 2H), 0.94 (s, 3H) ppm.

$^{11}B\{^1H\}$ NMR (160 MHz, d_6-acetone): δ –1.49 (q, J = 56 Hz) ppm.

$^{13}C\{^1H\}$ NMR (126 MHz, d_6-acetone): δ 49.6 (br), 34.6, 27.3, 24.0, 23.9 ppm. The carbonyl carbon directly attached to boron was not detected in the ^{13}C NMR spectrum, likely due to quadrupolar broadening.

$^{19}F\{^1H\}$ NMR (471 MHz, d_6-acetone): δ –143.9 (q, J = 56 Hz) ppm.

IR (ν/cm^{-1}, neat): 2927, 2884, 1655, 1465, 1133, 948.

HRMS (ESI): m/z calcd for $C_8H_{13}BF_3O$ $[M–K]^–$: 193.1017. Found: 193.1009.

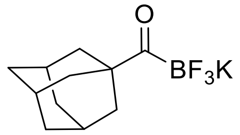

4q

4q was synthesized according to general procedure-1 and obtained as a white solid (65 mg, 0.24 mmol, 80%).

^{1}H NMR (500 MHz, d_6-acetone): δ 1.93 (m, 3H), 1.81 (d, *J* = 3 Hz, 6H), 1.73 – 1.66 (m, 6H) ppm.

^{11}B{^{1}H} NMR (160 MHz, d_6-acetone): δ –1.44 (q, *J* = 55 Hz) ppm.

^{13}C{^{1}H} NMR (126 MHz, d_6-acetone): δ 38.2, 38.1 (q, *J* = 1.1 Hz) ppm. The carbonyl carbon directly attached to boron was not detected in the ^{13}C NMR spectrum, likely due to quadrupolar broadening.

^{19}F{^{1}H} NMR (471 MHz, d_6-acetone): δ –143.9 (q, *J* = 55 Hz) ppm.

The NMR spectra are consistent with data previously reported. Reference: P. Tung, A. Schuhmacher, P. E. Schilling, J. W. Bode, N. P. Mankad, *Angew. Chem. Int. Ed.* **2022**, *61*, e202114513.

HRMS (ESI): m/z calcd for $C_{11}H_{15}BF_3O$ $[M–K]^–$: 231.1174. Found: 231.1170.

Elem. Anal.: Calc. (%) for $C_{11}H_{15}BF_3KO$: C 48.91, H 5.60. Found: C 48.93, H 5.69.

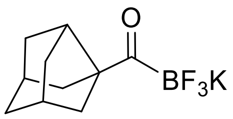

4r

4r was synthesized according to general procedure-1 and obtained as a white solid (61 mg, 0.24 mmol, 79%).

m.p.: 344 °C (decomp).

^{1}H NMR (500 MHz, d_6-acetone): δ 2.67 (t, *J* = 7.0 Hz, 1H), 2.19 (m, 2H), 2.15 – 2.12 (m, 2H), 1.76 – 1.72 (m, 2H), 1.62 – 1.53 (m, 4H), 1.48 (dd, *J* = 11.0, 3.0 Hz, 2H) ppm.

^{11}B{^{1}H} NMR (160 MHz, d_6-acetone): δ –1.40 (q, *J* = 55 Hz) ppm.

^{13}C{^{1}H} NMR (126 MHz, d_6-acetone): δ 63.3 (br), 45.6, 44.5, 41.4, 38.3, 36.6 ppm. The carbonyl carbon directly attached to boron was not detected in the ^{13}C NMR spectrum, likely due to quadrupolar broadening.

^{19}F{^{1}H} NMR (471 MHz, d_6-acetone): δ –145.6 (q, *J* = 55 Hz) ppm.

IR (ν/cm$^{–1}$, neat): 2922, 1622, 1043, 996, 821, 781.

HRMS (ESI): m/z calcd for $C_{10}H_{13}BF_3O$ $[M–K]^–$: 217.1017. Found: 217.1011.

Elem. Anal.: Calc. (%) for $C_{10}H_{13}BF_3KO$: C 46.90, H 5.12. Found: C 46.,93, H 5.13.

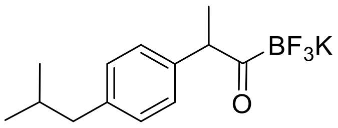

5a

5a was synthesized according to general procedure-1 and obtained as a white solid (68 mg, 0.23 mmol, 75%).

m.p.: 130 °C (decomp).

^{1}H NMR (500 MHz, *d$_6$*-acetone): δ 7.11 (d, *J* = 8 Hz, 2H, Ar–*H*), 7.00 (d, *J* = 8 Hz, 2H, Ar–*H*), 4.16 (q, *J* = 7 Hz, 1H, C*H*), 2.40 (d, *J* = 7 Hz, 2H, C*H$_2$*), 1.82 (hept, *J* = 7 Hz, 1H, C*H*), 1.21 (d, *J* = 7 Hz, 3H, Me–*H*), 0.87 (d, *J* = 7 Hz, 6H, Me–*H*) ppm.

^{11}B{^{1}H} NMR (160 MHz, *d$_6$*-acetone): δ –1.52 (q, *J* = 55 Hz) ppm.

^{13}C{^{1}H} NMR (126 MHz, *d$_6$*-acetone): δ 140.6, 139.1, 129.1, 129.0, 52.1 (br), 45.6, 30.9, 22.6, 17.1 ppm. The carbonyl carbon directly attached to boron was not detected in the ^{13}C NMR spectrum, likely due to quadrupolar broadening.

^{19}F{^{1}H} NMR (471 MHz, *d$_6$*-acetone): δ –149.1 (q, *J* = 55 Hz) ppm.

IR (ν/cm^{-1}, neat): 2954, 1634, 1058, 991, 906.

HRMS (ESI): m/z calcd for $C_{13}H_{17}BF_3O$ [M–K]$^-$: 257.1330. Found: 257.1327.

Elem. Anal.: Calc. (%) for $C_{13}H_{17}BF_3KO$: C 52.72, H 5.79. Found: C 52.58, H 5.90.

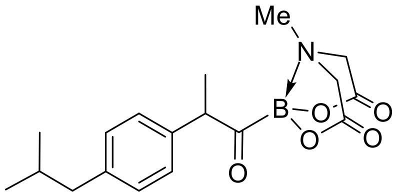

5b

5b was synthesized according to general procedure-3 and obtained as a white solid (45 mg, 0.13 mmol, 45%).

m.p.: 155 °C.

^{1}H NMR (300 MHz, $CDCl_3$): δ 7.16 (d, *J* = 8 Hz, 2H, Ar–*H*), 7.08 (d, *J* = 8 Hz, 2H, Ar–*H*), 4.27 (q, *J* = 7 Hz, 1H, C*H*), 3.84 – 3.69 (m, 3H, MIDA-C*H$_2$*), 3.12 (d, *J* = 16.5 Hz, 1H, MIDA-C*H$_2$*), 2.41 (d, *J* = 7 Hz, 2H, CH_2), 2.34 (s, 3H, Me–*H*), 1.81 (hept, *J* = 7 Hz, 1H, C*H*), 1.36 (d, *J* = 7 Hz, 3H, Me–*H*), 0.86 (d, *J* = 7 Hz, 6H, Me–*H*) ppm.

^{11}B{^{1}H} NMR (96 MHz, $CDCl_3$): δ 4.85 ppm.

$^{13}C\{^1H\}$ NMR (75 MHz, $CDCl_3$): δ 167.1, 141.1, 134.8, 129.7, 129.2, 62.3, 62.0, 54.7, 46.4, 45.0, 30.3, 22.4, 15.3 ppm. The carbonyl carbon directly attached to boron was not detected in the ^{13}C NMR spectrum, likely due to quadrupolar broadening.

IR (ν/cm^{-1}, neat): 2964, 1769, 1657, 1460, 1273, 1061.

HRMS (ESI): m/z calcd for $C_{18}H_{24}BNO_5Na$ $[M+Na]^+$: 368.1640. Found: 368.1635.

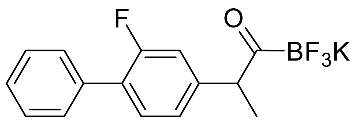

5c

5c was synthesized according to general procedure-1 and obtained as a white solid (80 mg, 0.24 mmol, 82%).

m.p.: 254 °C (decomp).

^1H NMR (500 MHz, d_6-acetone): δ 7.55 – 7.53 (m, 2H, Ar–*H*), 7.46 – 7.42 (m, 2H, Ar–*H*), 7.37 – 7.33 (m, 2H, Ar–*H*), 7.11 (dd, *J* = 7.5, 2.0 Hz, 1H, Ar–*H*), 7.05 (dd, *J* = 13.0, 2.0 Hz, 1H, Ar–*H*), 4.26 (q, *J* = 7.5 Hz, 1H, C*H*), 1.27 (d, *J* = 7.0 Hz, 3H, Me–H) ppm.

$^{11}B\{^1H\}$ NMR (160 MHz, d_6-acetone): δ –1.50 (q, *J* = 51 Hz) ppm.

$^{13}C\{^1H\}$ NMR (126 MHz, d_6-acetone): δ 160.1 (d, *J* = 246 Hz), 145.6, 136.9, 130.6, 129.6 (d, *J* = 4 Hz), 129.2, 128.2, 126.6 (d, *J* = 14 Hz), 125.7 (d, *J* = 3 Hz), 116.5, 52.1 (br), 16.9 ppm. The carbonyl carbon directly attached to boron was not detected in the ^{13}C NMR spectrum, likely due to quadrupolar broadening.

$^{19}F\{^1H\}$ NMR (471 MHz, d_6-acetone): δ –121.0, –149.2 (q, *J* = 51 Hz) ppm.

IR (ν/cm^{-1}, neat): 1657, 1624, 1485, 1415, 1028, 923, 888.

HRMS (ESI): m/z calcd for $C_{15}H_{12}BF_4O$ $[M–K]^-$: 295.0923. Found: 295.0919.

Elem. Anal.: Calc. (%) for $C_{15}H_{12}BF_4KO$: C 53.92, H 3.62. Found: C 54.03, H 3.41.

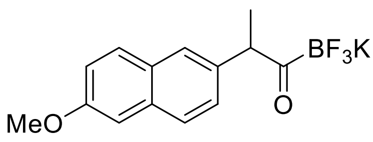

5d

5d was synthesized according to general procedure-1 and obtained as a white solid (67 mg, 0.21 mmol, 70%).

m.p.: 210 °C (decomp).

^{1}H NMR (500 MHz, d_6-acetone): δ 7.81 – 7.79 (m, 1H, Ar–*H*), 7.63 (d, *J* = 8.5 Hz, 1H, Ar–*H*), 7.58 (s, 1H, Ar–*H*), 7.34 (dd, *J* = 8.0, 1.5 Hz, 1H, Ar–*H*), 7.20 (d, *J* = 2.5 Hz, 1H, Ar–*H*), 7.07 (dd, *J* = 8.5, 2.5 Hz, 1H, Ar–*H*), 4.33 (q, *J* = 7.5 Hz, 1H, C*H*), 3.88 (s, 3H, OMe–*H*), 1.31 (d, *J* = 7.5 Hz, 3H, Me–*H*) ppm.

^{11}B{^{1}H} NMR (160 MHz, d_6-acetone): δ –1.47 (q, *J* = 53 Hz) ppm.

^{13}C{^{1}H} NMR (126 MHz, d_6-acetone): δ 158.0, 138.8, 134.1, 130.0, 129.8, 129.0, 127.1, 126.8, 119.0, 106.3, 55.5, 52.6 (br), 17.1 ppm. The carbonyl carbon directly attached to boron was not detected in the ^{13}C NMR spectrum, likely due to quadrupolar broadening.

^{19}F{^{1}H} NMR (471 MHz, d_6-acetone): δ –149.1 (q, *J* = 53 Hz) ppm.

IR (v/cm^{-1}, neat): 1632, 1604, 1262, 1214, 1020, 903.

HRMS (ESI): m/z calcd for $C_{14}H_{13}BF_3O_2$ $[M–K]^-$: 281.0966. Found: 281.0963.

Elem. Anal.: Calc. (%) for $C_{14}H_{13}BF_3KO_2$: C 52.52, H 4.09. Found: C 52.34, H 4.40.

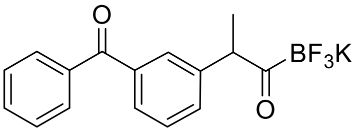

5e

5e was synthesized according to general procedure-1 and obtained as a white solid (76 mg, 0.22 mmol, 75%).

m.p.: 320 °C (decomp).

^{1}H NMR (500 MHz, d_6-acetone): δ 7.81 – 7.79 (m, 2H, Ar–*H*), 7.67 – 7.62 (m, 2H, Ar–*H*), 7.56 – 7.53 (m, 3H, Ar–*H*), 7.48 (m, 1H, Ar–*H*), 7.40 (t, *J* = 7.5 Hz, 1H, Ar–*H*), 4.28 (q, *J* = 7.0 Hz, 1H, C*H*), 1.28 (d, *J* = 7.0 Hz, 3H, Me–H) ppm.

^{11}B{^{1}H} NMR (96 MHz, d_6-acetone): δ –1.48 (br q, *J* = 53 Hz) ppm.

^{13}C{^{1}H} NMR (126 MHz, d_6-acetone): δ 196.5, 143.3, 138.4, 137.6, 133.3, 132.8, 130.6, 130.4, 128.9, 128.3, 127.7, 52.5 (br), 16.7 ppm. The carbonyl carbon directly attached to boron was not detected in the ^{13}C NMR spectrum, likely due to quadrupolar broadening.

^{19}F{^{1}H} NMR (471 MHz, d_6-acetone): δ–148.9 (q, *J* = 53 Hz) ppm.

IR (v/cm^{-1}, neat): 1662, 1595, 1026, 958, 751.

HRMS (ESI): m/z calcd for $C_{16}H_{13}BF_3O_2$ $[M–K]^-$: 305.0966. Found: 305.0965.

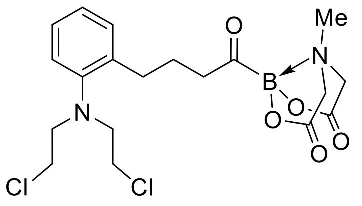

5f

5f was synthesized according to general procedure-3 and obtained as a colorless oil (57 mg, 0.13 mmol, 43%).

^{1}H NMR (300 MHz, $CDCl_3$): δ 7.07 (m, 2H, Ar–*H*), 6.67 (m, 2H, Ar–*H*), 3.88 (s, 4H, MIDA–CH_2), 3.70 – 3.63 (m, 8H, alkyl–*H*), 2.92 (s, 3H, MIDA–CH_3), 2.68 (t, *J* = 7 Hz, 2H, alkyl–*H*), 2.52 (t, *J* = 7 Hz, 2H, alkyl–*H*), 1.83 (quint, *J* = 7 Hz, 2H, alkyl–*H*) ppm.

^{11}B{^{1}H} NMR (96 MHz, $CDCl_3$): δ 4.45 ppm.

^{13}C{^{1}H} NMR (75 MHz, $CDCl_3$): δ 166.6, 129.9, 112.9, 63.4, 54.1, 46.1, 40.4, 34.3, 23.9 ppm. The carbonyl carbon directly attached to boron was not detected in ^{13}C NMR, likely due to quadrupolar broadening.

IR (ν/cm^{-1}, neat): 2932, 2862, 1724, 1649, 1614, 1023.

HRMS (APCI): m/z calcd for $C_{19}H_{25}BCl_2N_2O_5$ $[M+H]^+$: 443.1306. Found: 443.1298.

2.4 Scale-up reaction

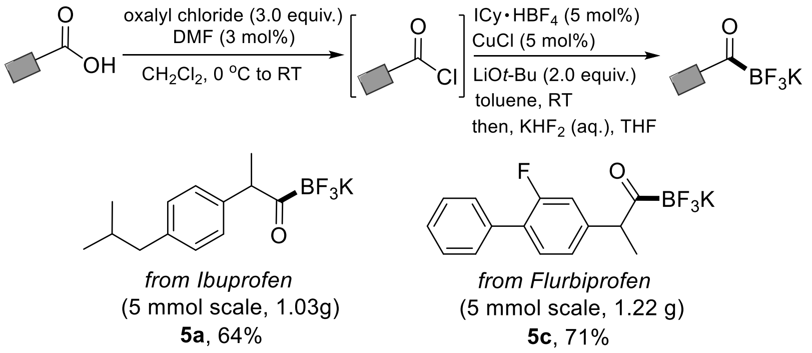

Scheme S9. Gram-scale copper-catalyzed borylation of acyl chlorides with B_2pin_2 to synthesize acylboron compounds from Ibuprofen and Flurbiprofen.

To a Schlenk flask equipped with a magnetic stirring bar was added with carboxylic acid-based drugs (5.0 mmol, Ibuprofen 1.03 g or Flurbiprofen 1.22 g), DMF (3 mol%, 11 mg) and CH_2Cl_2 (10 mL) under argon atmosphere. The reaction mixture was cooled to 0

^{o}C with an ice bath. A CH_2Cl_2 solution (10 mL) of oxalyl chloride (3.0 equiv., 1.9 g) was added dropwise at 0 ^{o}C and the mixture was allowed to warm to room temperature and stirred for 12 h. Upon completion, the solvent was removed under reduced pressure and the acyl chloride generated was used directly in the next step without further purification.

A two-necked Schlenk flask was equipped with a pressure equalizing dropping funnel and a magnetic stirring bar. The flask was degassed by evacuation and refilling with argon three times. $ICy{\cdot}HBF_4$ (5 mol%, 0.25 mmol, 80 mg), CuCl (5 mol%, 0.25 mmol, 25 mg), and LiO*t*-Bu (2.0 equiv., 10 mmol, 800 mg) were added, followed by the addition of THF (10 mL), and the reaction mixture was stirred for 1 h. Then, the THF was removed under reduced pressure and a toluene (20 mL) solution of B_2pin_2 (1.0 equiv., 5.0 mmol, 1.27g) was added. The mixture was stirred for 30 min at room temperature, followed by the dropwise addition of a toluene solution (20 mL) of acyl chloride (1.0 equiv.) generated in the previous step using a dropping funnel under an argon atmosphere. The reaction mixture was vigorously stirred at room temperature for 6 h (Note: during the addition of acyl chloride, a significant amount of precipitate formed for both reactions which may interfere with the stirring). Upon completion, aqueous KHF_2 solution (3.0 M, 45 mmol in 15 mL of H_2O, 9.0 equiv.) was added to the reaction mixture under an argon atmosphere, followed by the addition of THF (40 mL), and the reaction was stirred at room temperature for another 24 h. The resulting mixture was open to air and the solvents were concentrated under reduced pressure. Acetone was added to dissolve the organic residue and the solution was filtered. The filtrate was concentrated under reduced pressure and the KAT products were crystalized from the remaining residue using Et_2O and *n*-hexane and isolated as white solids **5a** (947 mg, 64%), **5c** (1.18 g, 71%). (Note: the pinacol byproduct in the residue may affect the crystallization and we suggest to removing it under reduced pressure at elevated temperature).

3. Crystallographic data

Table S2. Single-crystal X-ray diffraction data and refinement details of **2a**, **3** and **Im-3**.

Data	**2a**	**3**	**Im-3**
CCDC number	2129578	2129577	2152003
Empirical formula	$C_8H_7BF_3KO$	$C_{47}H_{67}B_2CuN_2O_5$	$0.846(C_{40}H_{62}B_4Li_2O_{12}) \cdot 0.154(C_{36}H_{52}B_2Li_2O_{12})$
Formula weight / g·mol^{-1}	226.05	825.18	779.90
T / K	100(2)	100(2)	100(2)
Radiation, λ / Å	Cu-K$_\alpha$ 1.54184	Cu-K$_\alpha$ 1.54184	Cu-K$_\alpha$ 1.54184
Crystal size / mm³	0.100×0.192×0.255	0.087×0.142×0.242	0.101×0.052×0.014
Crystal color, habit	colorless block	colorless block	colorless plate
μ / mm^{-1}	5.185	1.001	0.704
Crystal system	monoclinic	monoclinic	monoclinic
Space group	$P2_1/n$	$P2_1/c$	$P2_1/c$
a / Å	6.51663(5)	10.67881(10)	12.0494(4)
b / Å	8.69993(8)	26.1626(2)	15.2854(3)
c / Å	32.5622(2)	16.72558(16)	12.5879(4)
α / °	90	90	90
β / °	90.6185(7)	90.5670(8)	114.999(4)
γ / °	90	90	90
Volume / Å^3	1845.98(2)	4672.66(8)	2101.23(12)
Z	8	4	2
ρ_{calc} / g·cm^{-3}	1.627	1.173	1.233
F(000)	912	1768	835
θ range / °	5.263 – 74.487	3.136 – 74.483	4.048 – 74.495
Reflections collected	22838	50043	22722
Unique reflections	3732	9498	4275
Parameters / restraints	255 / 0	531 / 0	335 / 128
GooF on F^2	1.037	1.058	1.055
R_1 [$I>2\sigma(I)$]	0.0263	0.0354	0.0535
wR_2 (all data)	0.0720	0.0958	0.1320
Max. / min. residual electron density / e·Å^{-3}	0.423 / –0.289	0.394 / –0.492	0.422 / –0.210

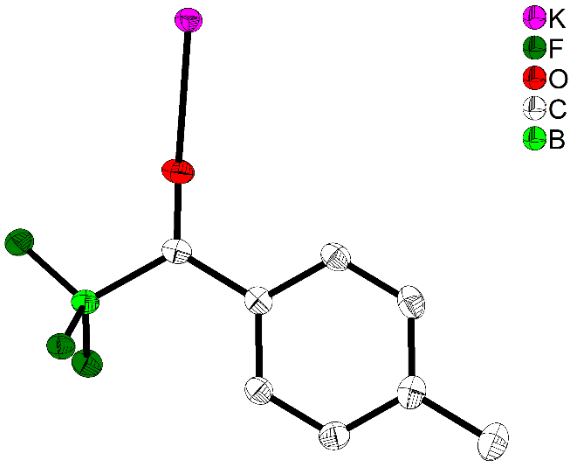

Figure S7. The solid-state molecular structure of **2a** determined by single-crystal X-ray diffraction at 100 K. All ellipsoids are drawn at the 50% probability level. H atoms are omitted for clarity. Only one of two independent molecules is shown.

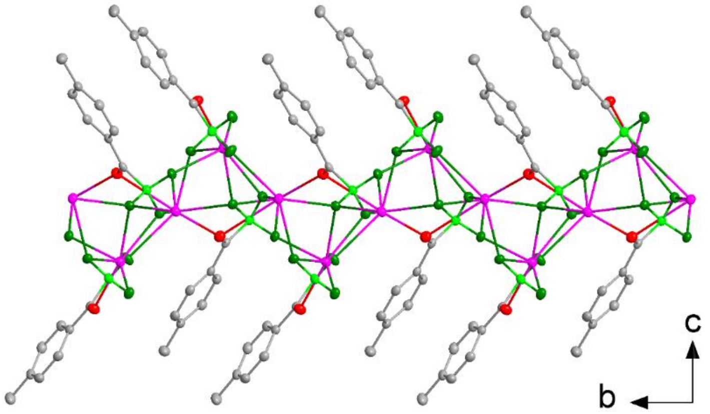

Figure S8. Selected section of the crystal structure of **2a** showing the 2-dimensional polymeric unit projected along the *a* axis. Data were collected at 100 K. All ellipsoids are drawn at the 50% probability level. H atoms are omitted for clarity.

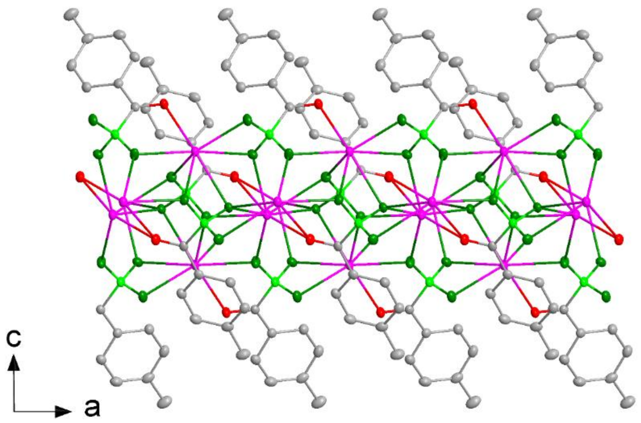

Figure S9. Selected section of the crystal structure of **2a** showing the 2-dimensional polymeric unit projected along the *b* axis. Data were collected at 100 K. All ellipsoids are drawn at the 50% probability level. H atoms are omitted for clarity.

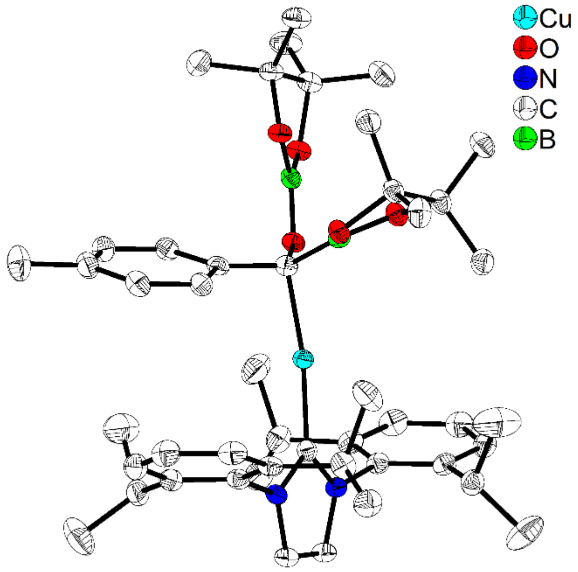

Figure S10. The solid-state molecular structure of **3** determined by single-crystal X-ray diffraction at 100 K. All ellipsoids are drawn at the 50% probability level. H atoms are omitted for clarity.

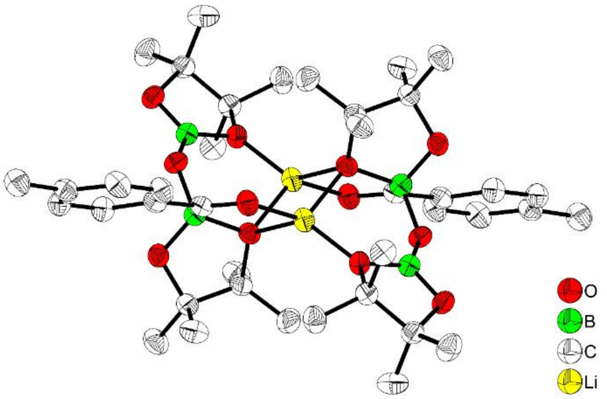

Figure S11. The major part (ca. 85%) of the solid-state molecular structure of **Im-3** determined by single-crystal X-ray diffraction at 100 K. All ellipsoids are drawn at the 50% probability level. H atoms are omitted for clarity.

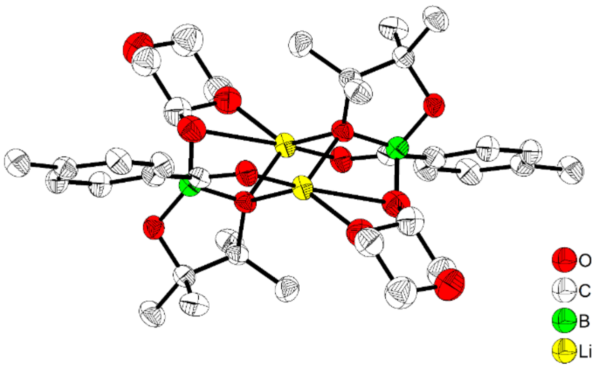

Figure S12. The minor part (ca. 15%) of the solid-state molecular structure of **Im-3** determined by single-crystal X-ray diffraction at 100 K. Here, two symmetry-equivalent Bpin moieties are replaced by O-dioxane. All ellipsoids are drawn at the 50% probability level. H atoms are omitted for clarity. This minor part of the structure is a 2-oxy-1,4-dioxane adduct of the acylBpin compound. The 2-oxy-1,4-dioxane is most probably derived from a trace amount of 2-hydroxy-1,4-dioxane in the 1,4-dioxane solvent.

4. References

[1] W. Xie, S. Chang, *Angew. Chem. Int. Ed.* **2016**, *55*, 1876–1880; *Angew. Chem.* **2016**, *128*, 1908–1912.

[2] M. L. McIntosh, C. M. Moore, T. B. Clark, *Org. Lett.* **2010**, *12*, 1996–1999.

[3] D. S. Laitar, P. Müller, J. P. Sadighi, *J. Am. Chem. Soc.* **2005**, *127*, 17196–17197.

[4] Y. H. Lee, B. Morandi, *Nature Chem.* **2018**, *10*, 1016–1022.

[5] G. M. Sheldrick, *Acta Crystallogr.* **2015**, *A71*, 3–8.

[6] G.M. Sheldrick, *Acta Crystallogr.* **2015**, *C71*, 3–8.

[7] C. Hübschle, G. M. Sheldrick, B. Dittrich, *J. Appl. Cryst.* **2011**, *44*, 1281–1284.

5. NMR Spectra

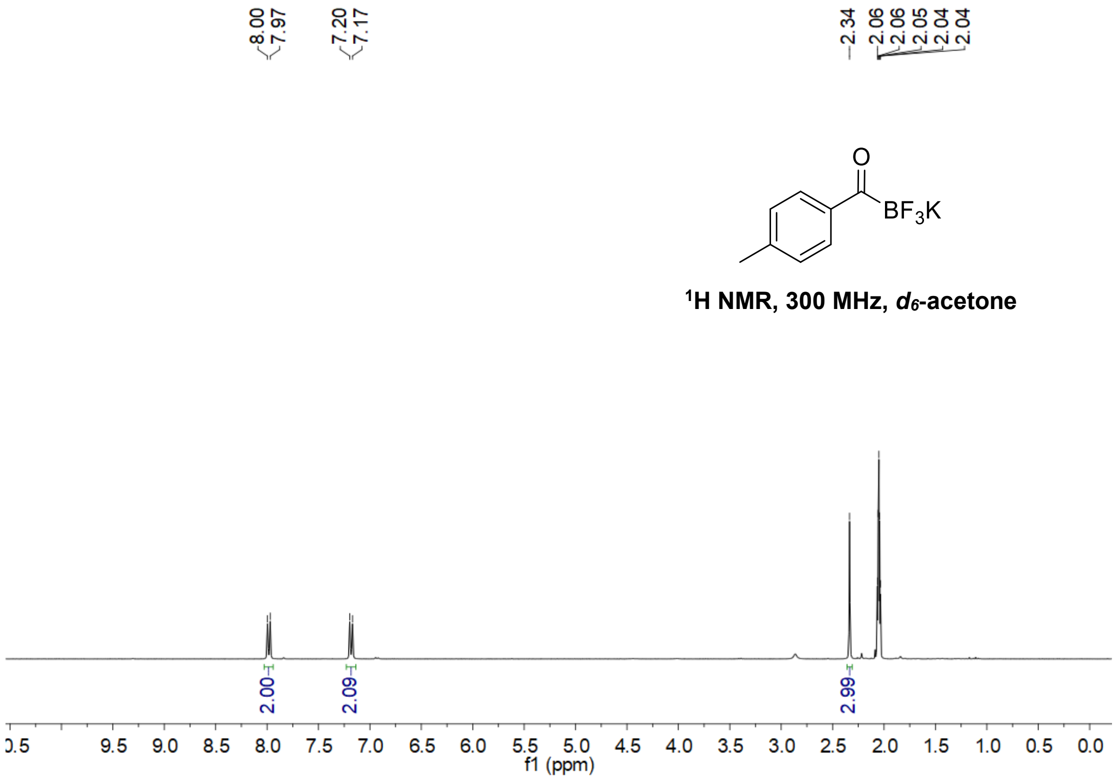

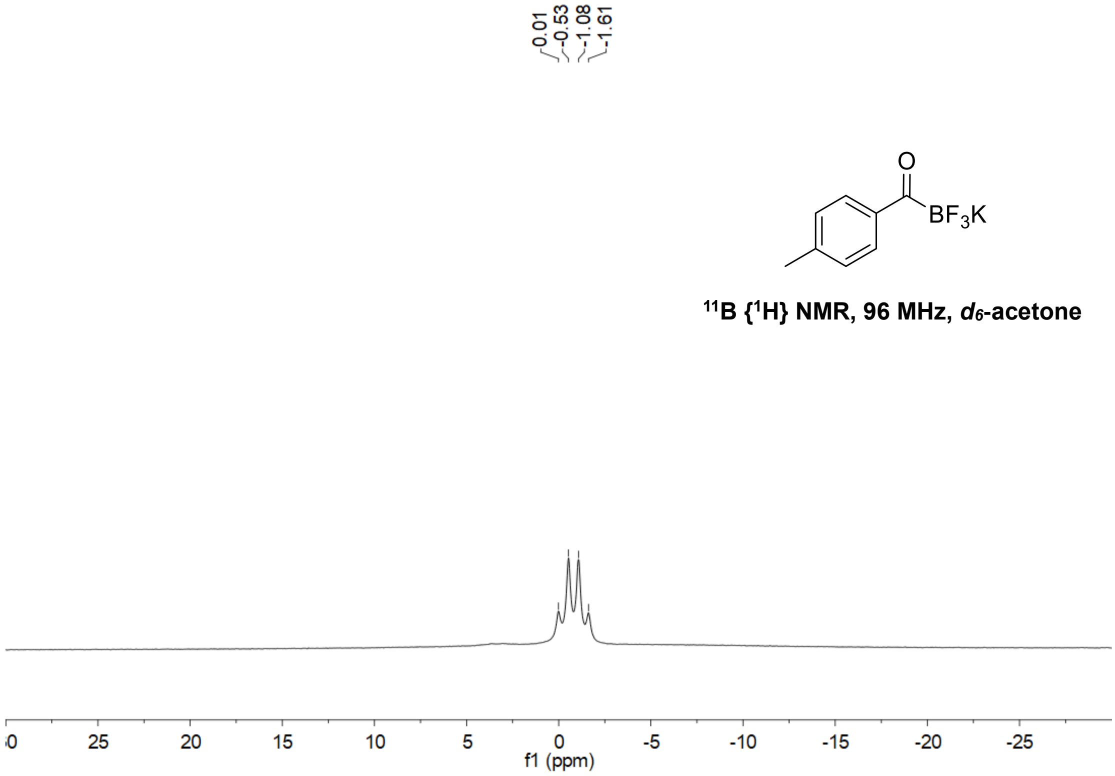

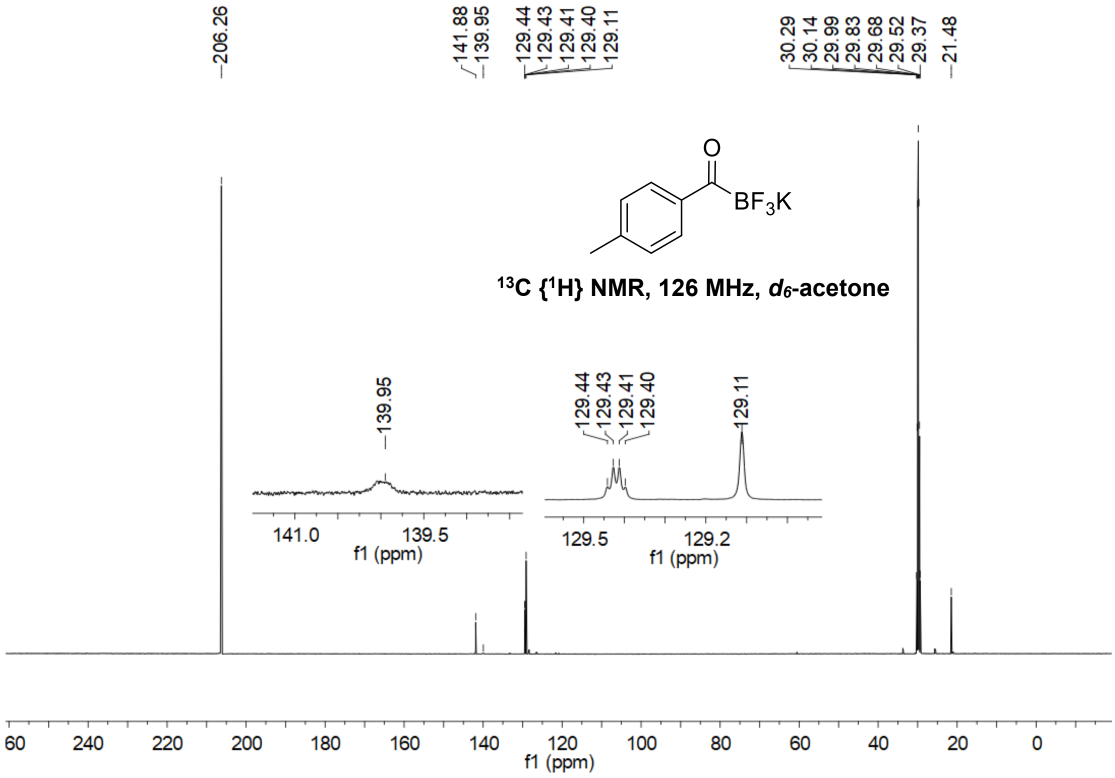
206.26
141.88
139.95
129.44
129.43
129.41
129.40
129.11
30.29
30.14
29.99
29.83
29.68
29.52
29.37
21.48
O
BF3K
13C {1H} NMR, 126 MHz, d6-acetone
139.95
141.0
139.5
f1 (ppm)
129.44
129.43
129.41
129.40
129.11
129.5
129.2
f1 (ppm)
60
240
220
200
180
160
140
120
100
80
60
40
20
0
f1 (ppm)

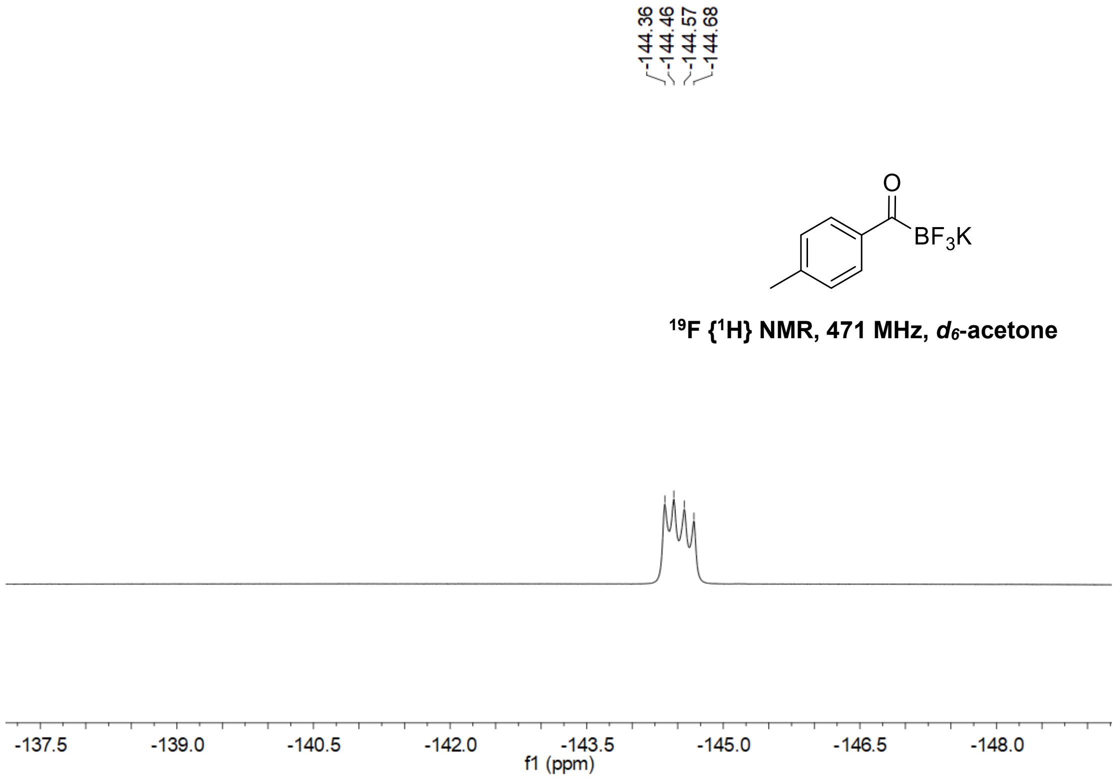
-144.36
-144.46
-144.57
-144.68
O
BF3K
19F {1H} NMR, 471 MHz, d6-acetone
-137.5
-139.0
-140.5
-142.0
-143.5
-145.0
-146.5
-148.0
f1 (ppm)

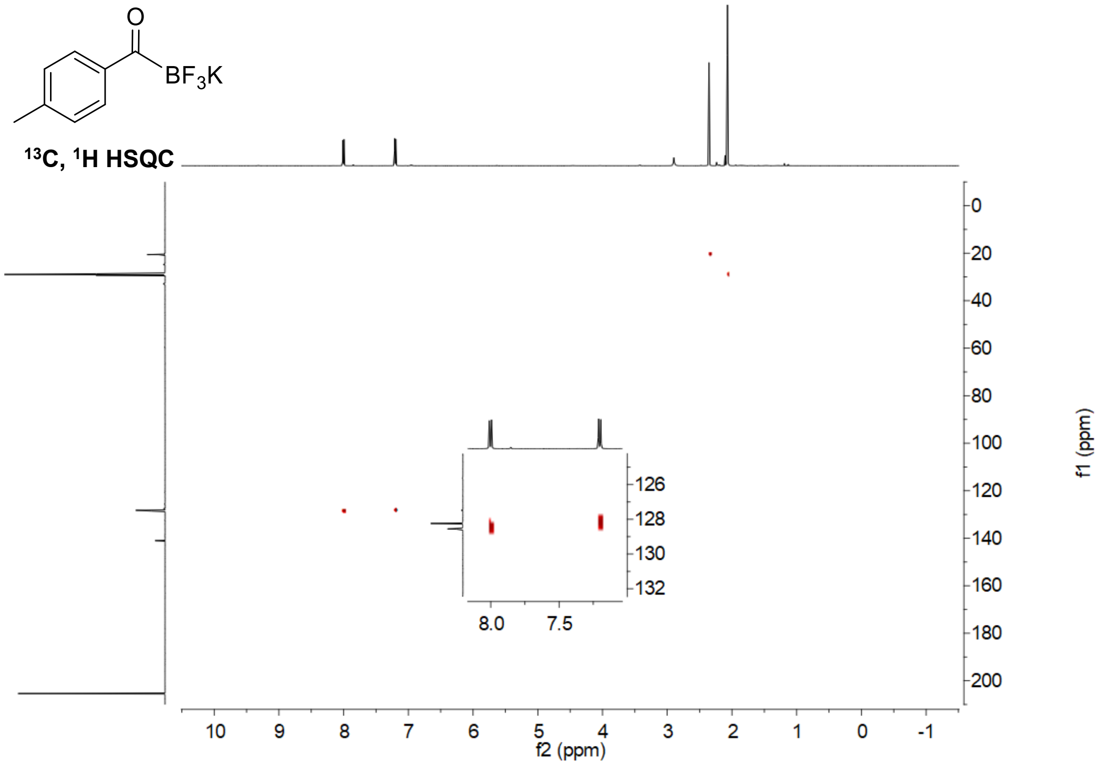

O
BF3K
13C, 1H HSQC
f1 (ppm)
f2 (ppm)

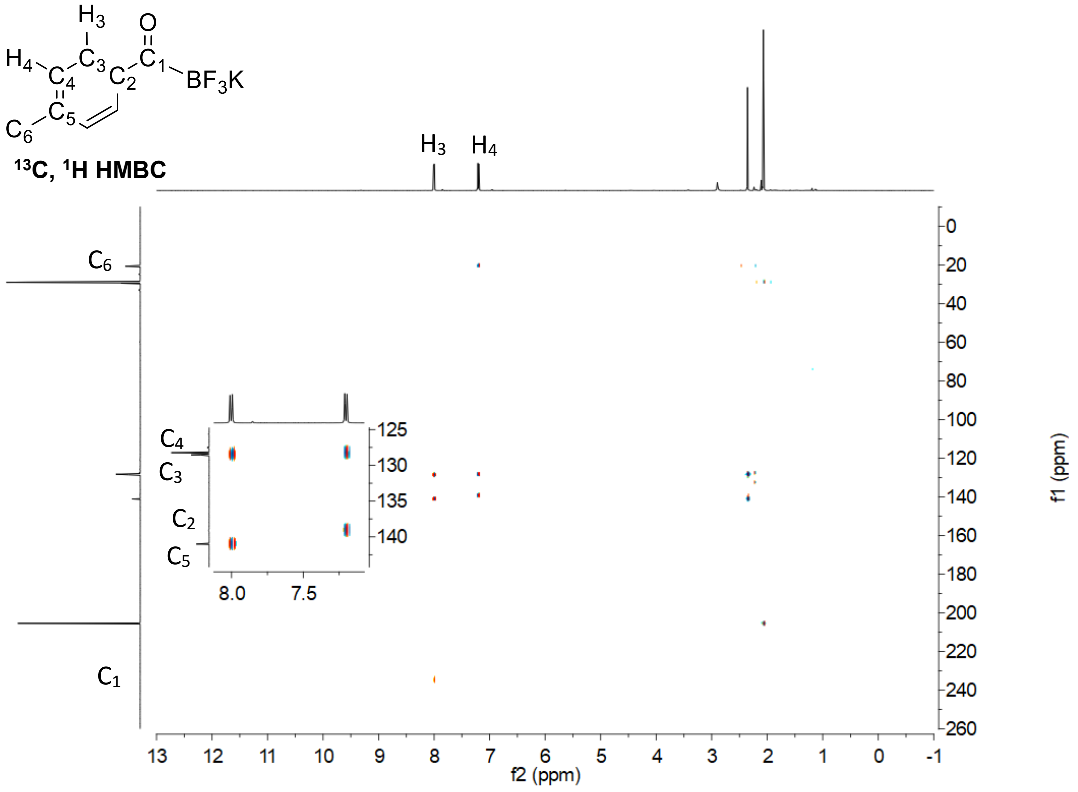

H3
H4
C3
C4
C2
C1
O
BF3K
C5
C6
13C, 1H HMBC
f1 (ppm)
f2 (ppm)

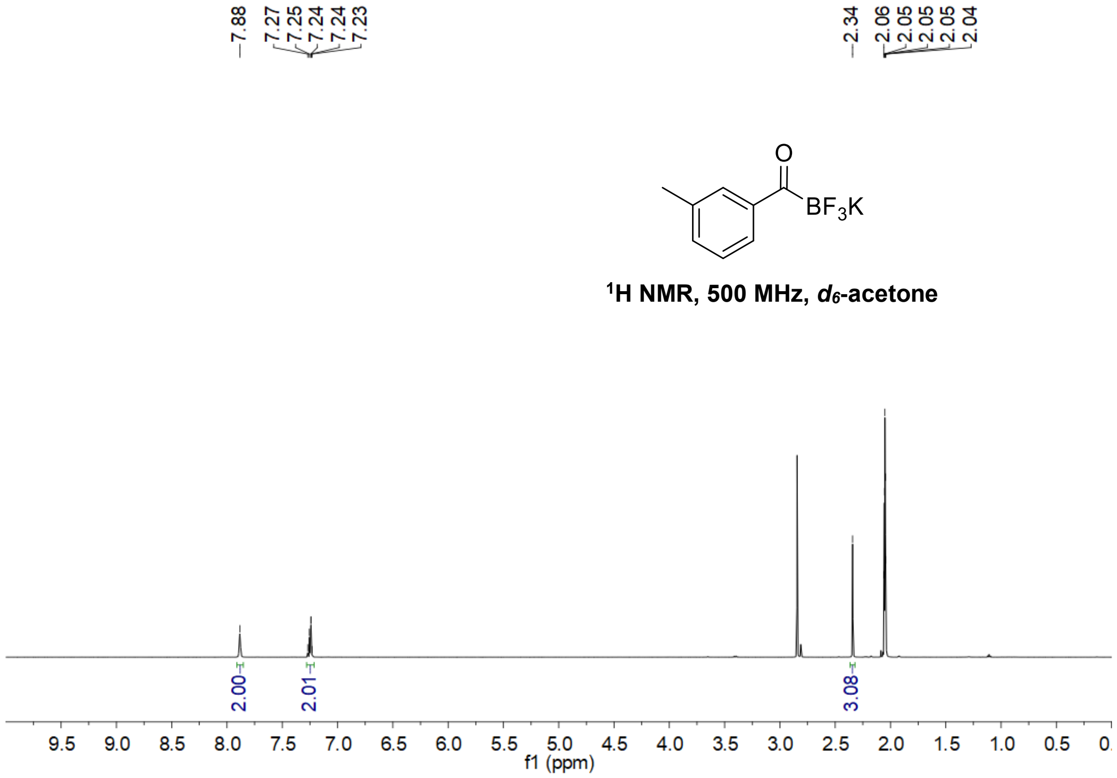
7.88
7.27
7.25
7.24
7.24
7.23
2.34
2.06
2.05
2.05
2.05
2.04
O
BF3K
1H NMR, 500 MHz, d6-acetone
2.00
2.01
3.08
9.5 9.0 8.5 8.0 7.5 7.0 6.5 6.0 5.5 5.0 4.5 4.0 3.5 3.0 2.5 2.0 1.5 1.0 0.5
f1 (ppm)

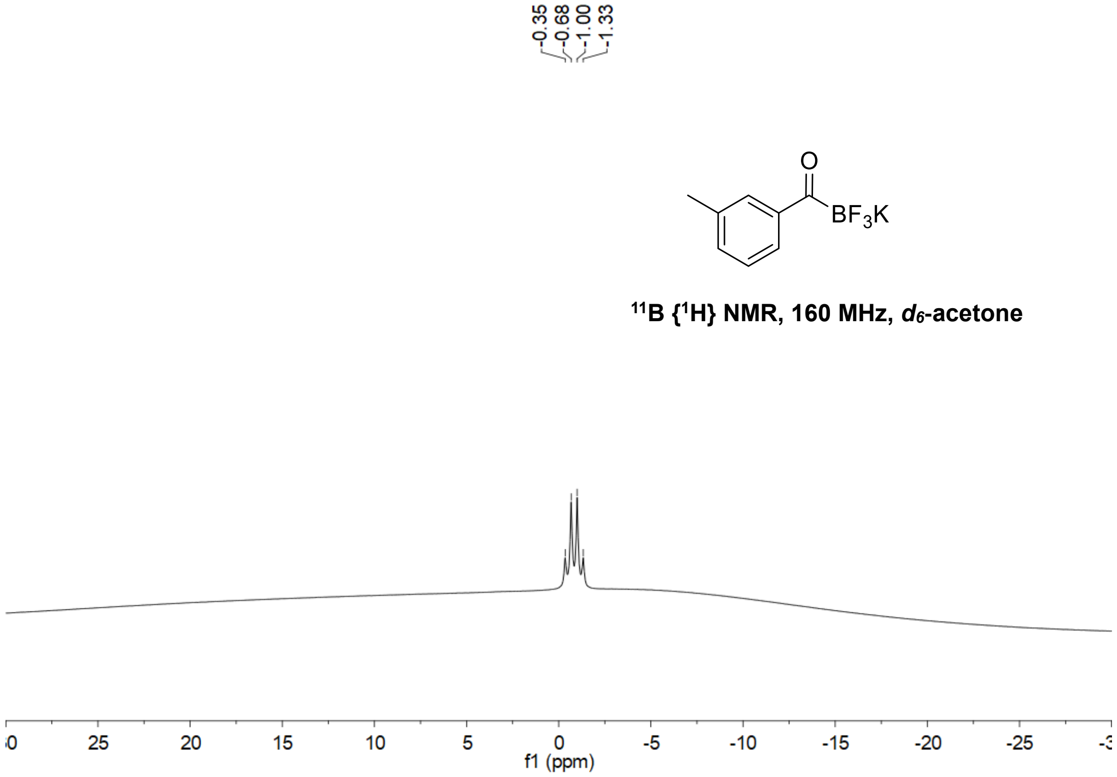
-0.35
-0.68
-1.00
-1.33
O
BF3K
11B {1H} NMR, 160 MHz, d6-acetone
25 20 15 10 5 0 -5 -10 -15 -20 -25
f1 (ppm)

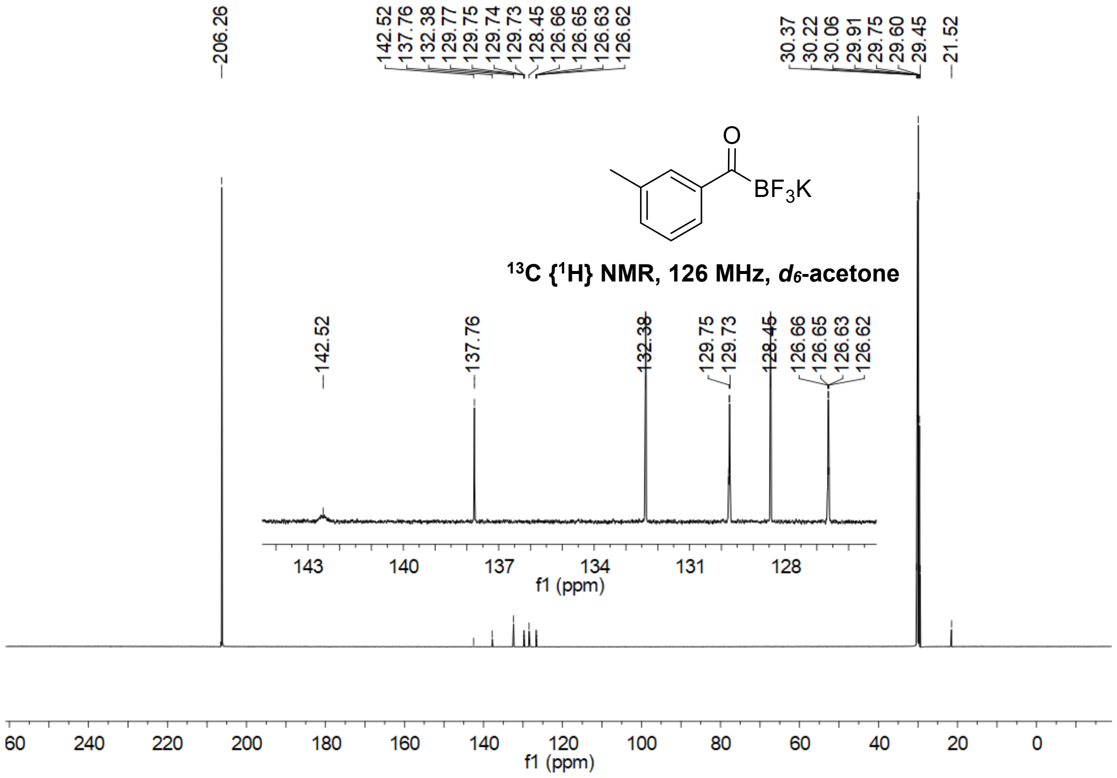
206.26
142.52
137.76
132.38
129.77
129.75
129.74
129.73
128.45
126.66
126.65
126.63
126.62
30.37
30.22
30.06
29.91
29.75
29.60
29.45
21.52
O
BF3K
13C {1H} NMR, 126 MHz, d6-acetone
f1 (ppm)

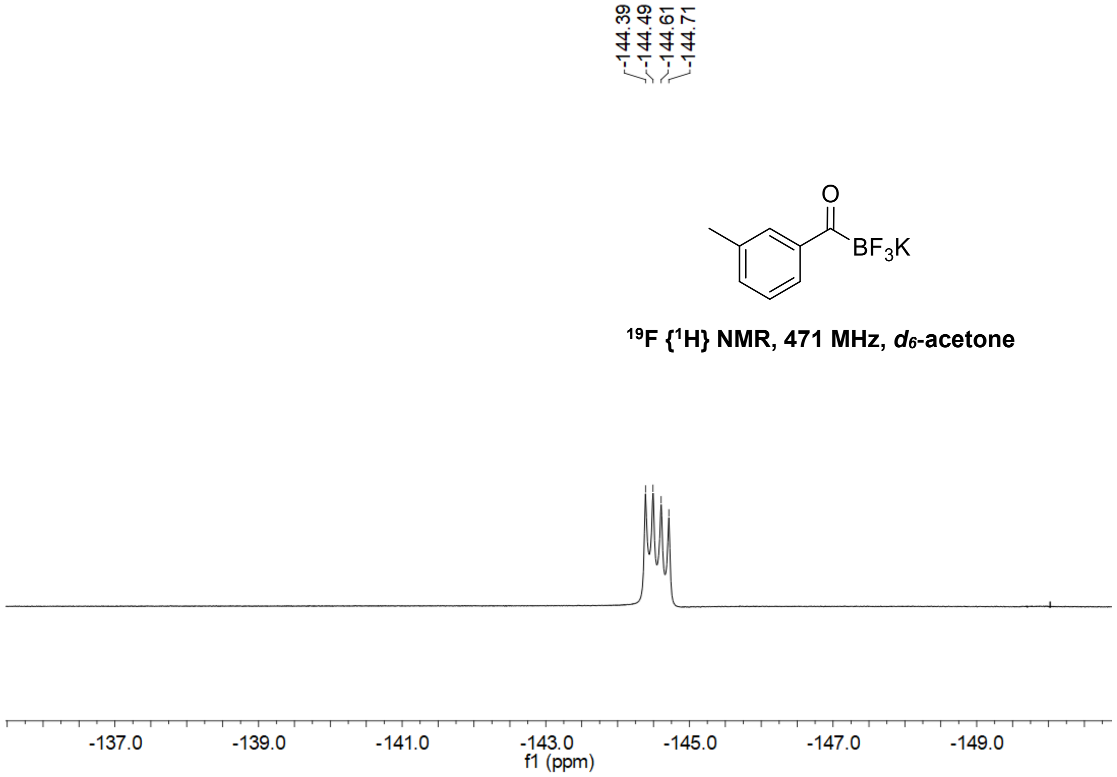
-144.39
-144.49
-144.61
-144.71
O
BF3K
19F {1H} NMR, 471 MHz, d6-acetone
f1 (ppm)

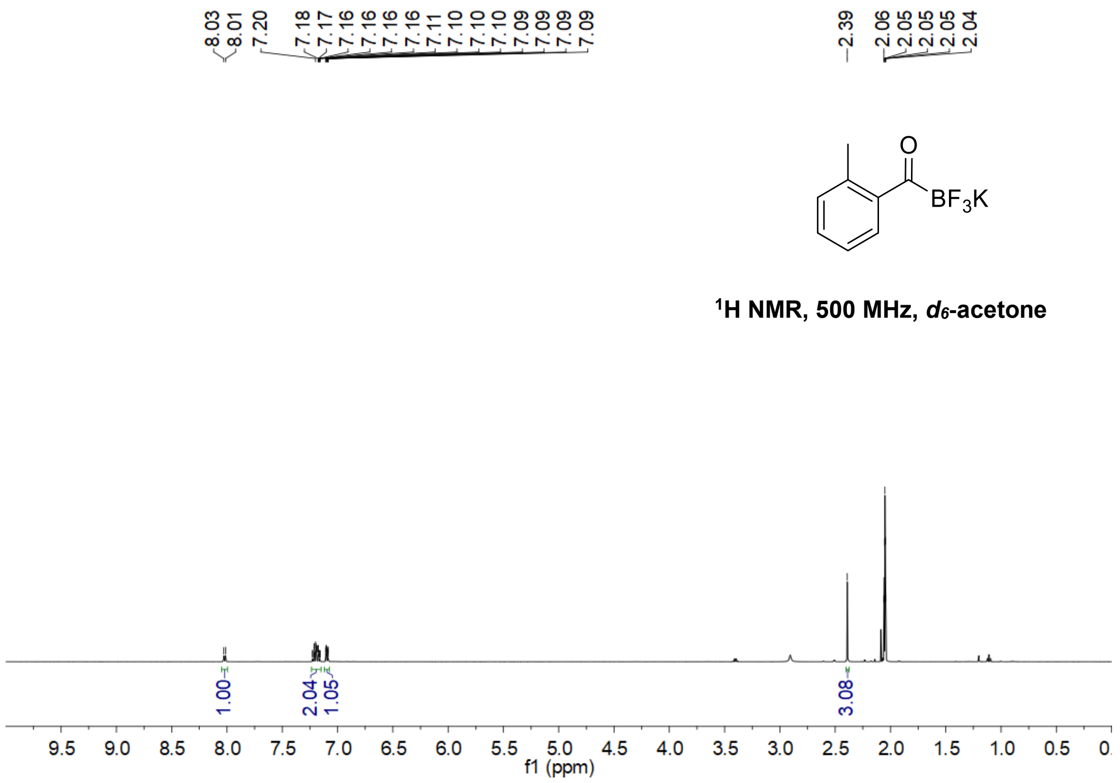
8.03
8.01
7.20
7.18
7.17
7.16
7.16
7.16
7.11
7.10
7.10
7.10
7.09
7.09
7.09
7.09
2.39
2.06
2.05
2.05
2.05
2.04
O
BF3K
1H NMR, 500 MHz, d6-acetone
1.00
2.04
1.05
3.08
f1 (ppm)

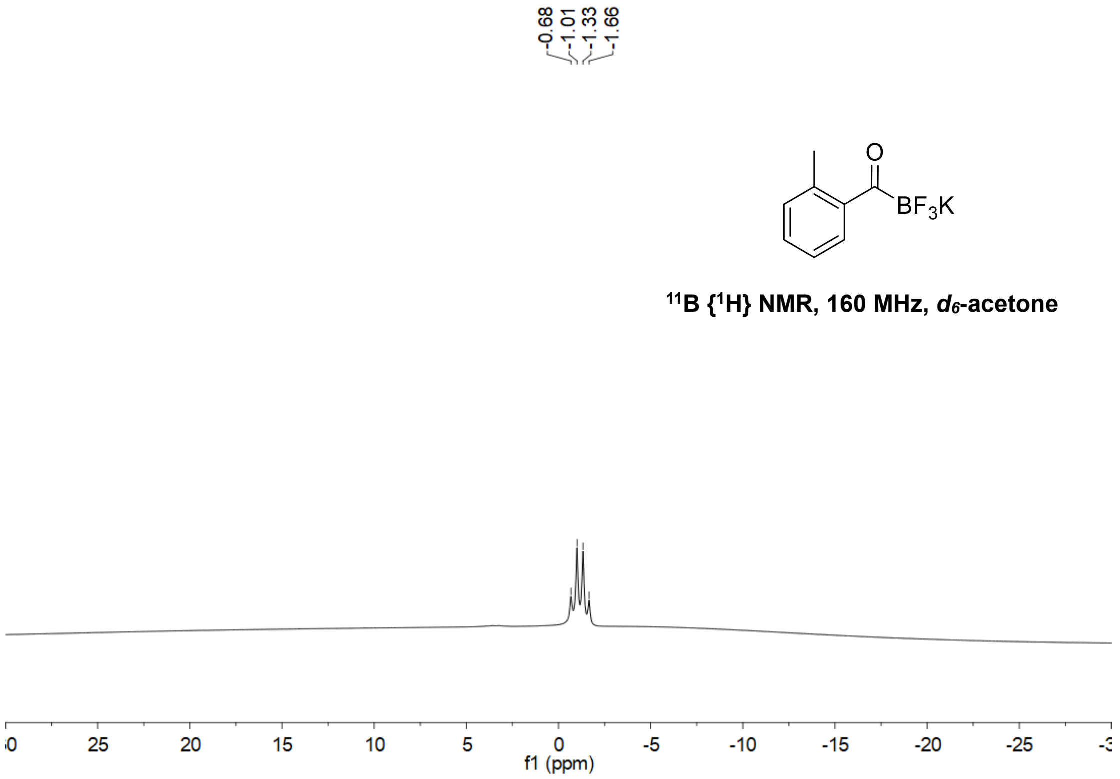
-0.68
-1.01
-1.33
-1.66
O
BF3K
11B {1H} NMR, 160 MHz, d6-acetone
f1 (ppm)

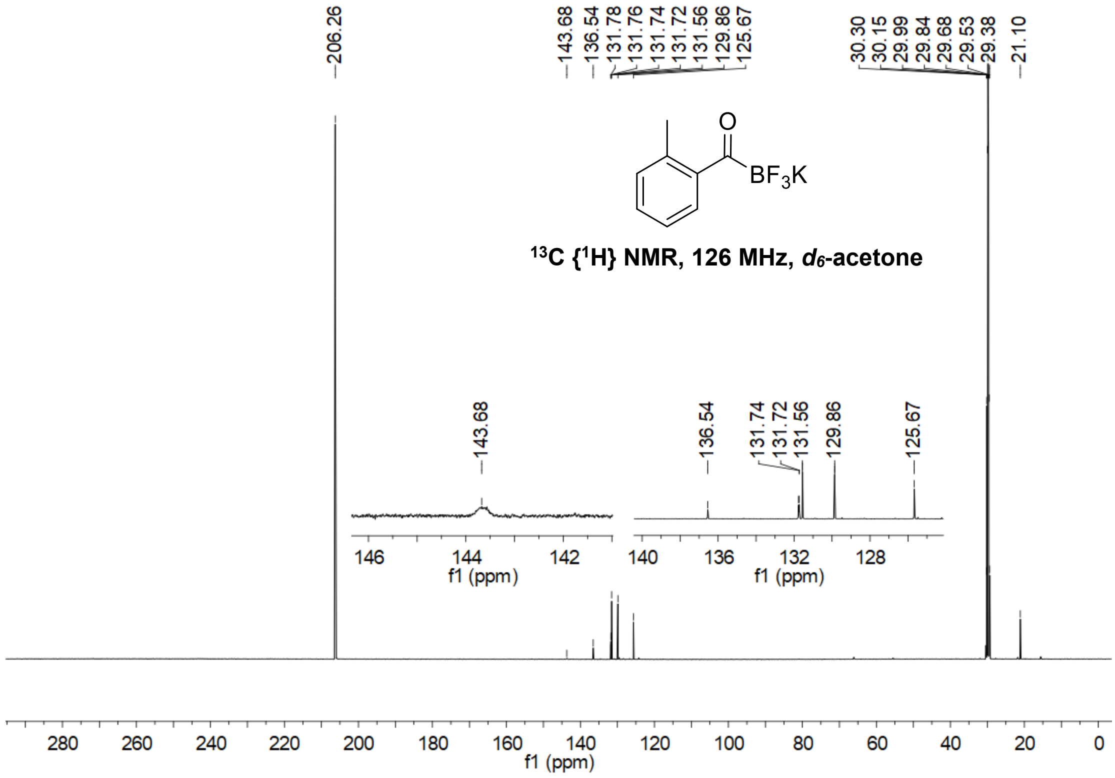

206.26
143.68
136.54
131.78
131.76
131.74
131.72
131.56
129.86
125.67
30.30
30.15
29.99
29.84
29.68
29.53
29.38
21.10
O
BF3K
13C {1H} NMR, 126 MHz, d6-acetone
f1 (ppm)

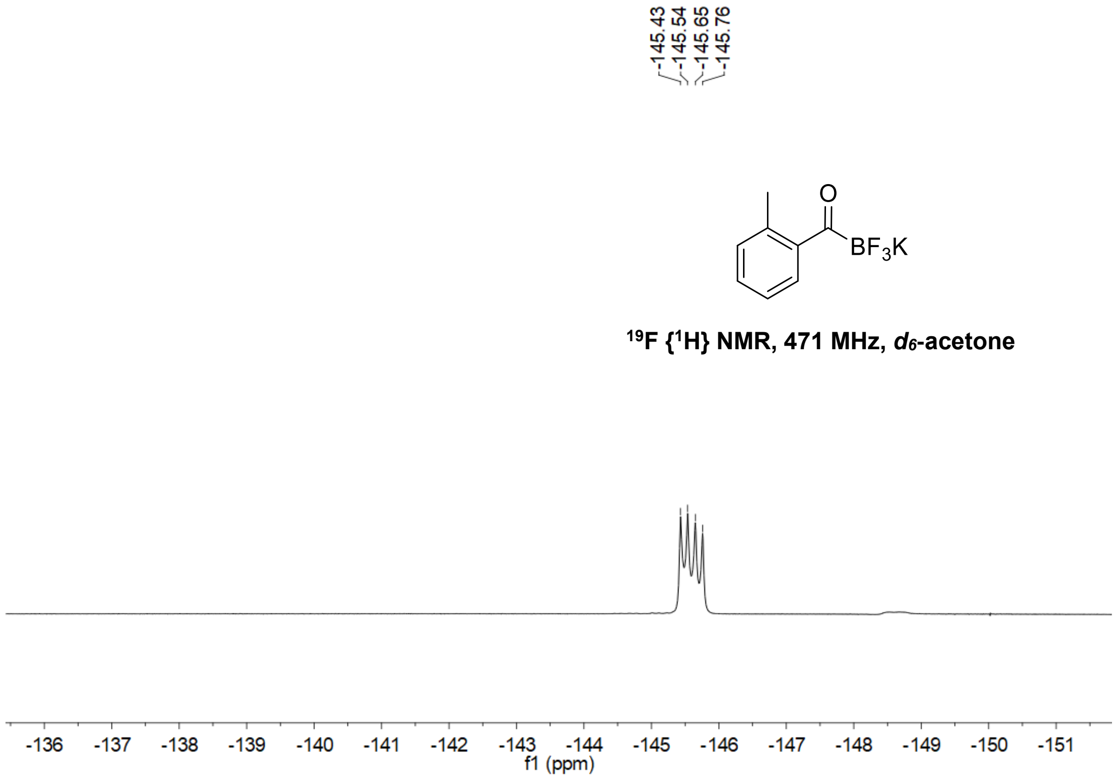

-145.43
-145.54
-145.65
-145.76
O
BF3K
19F {1H} NMR, 471 MHz, d6-acetone
f1 (ppm)

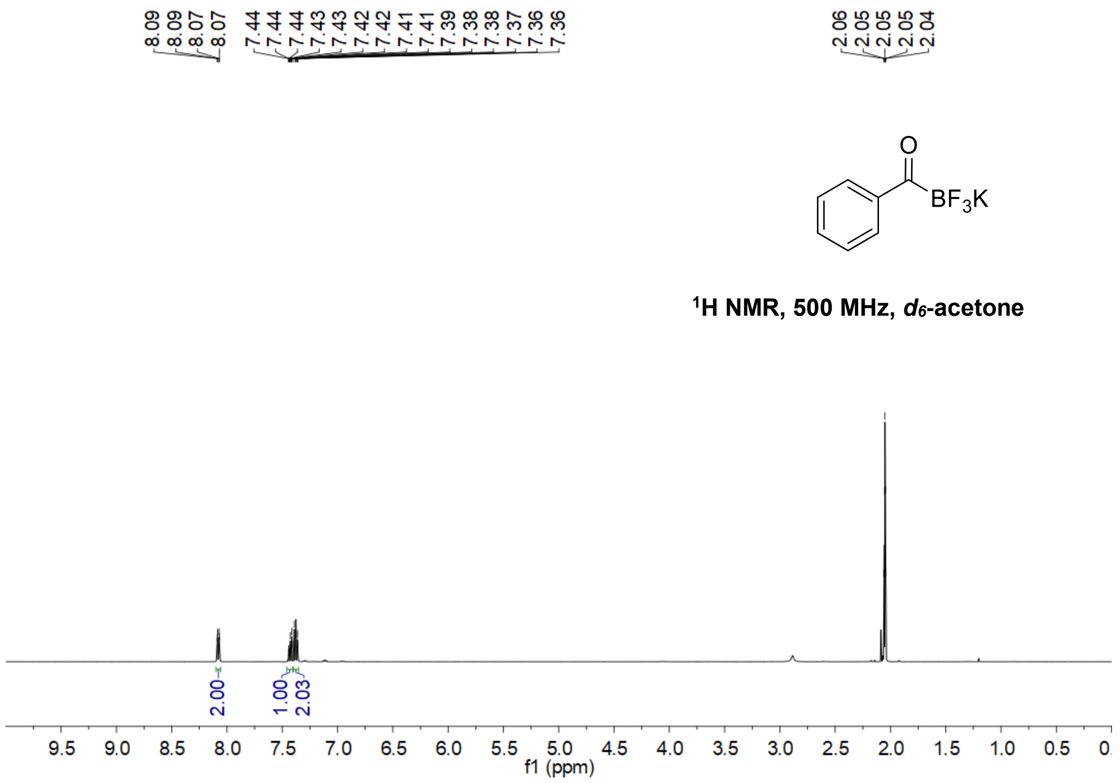
8.09
8.09
8.07
8.07
7.44
7.44
7.44
7.43
7.43
7.42
7.42
7.41
7.41
7.39
7.38
7.38
7.37
7.36
7.36
2.06
2.05
2.05
2.05
2.04
O
BF3K
1H NMR, 500 MHz, d6-acetone
2.00
1.00
2.03
9.5 9.0 8.5 8.0 7.5 7.0 6.5 6.0 5.5 5.0 4.5 4.0 3.5 3.0 2.5 2.0 1.5 1.0 0.5
f1 (ppm)

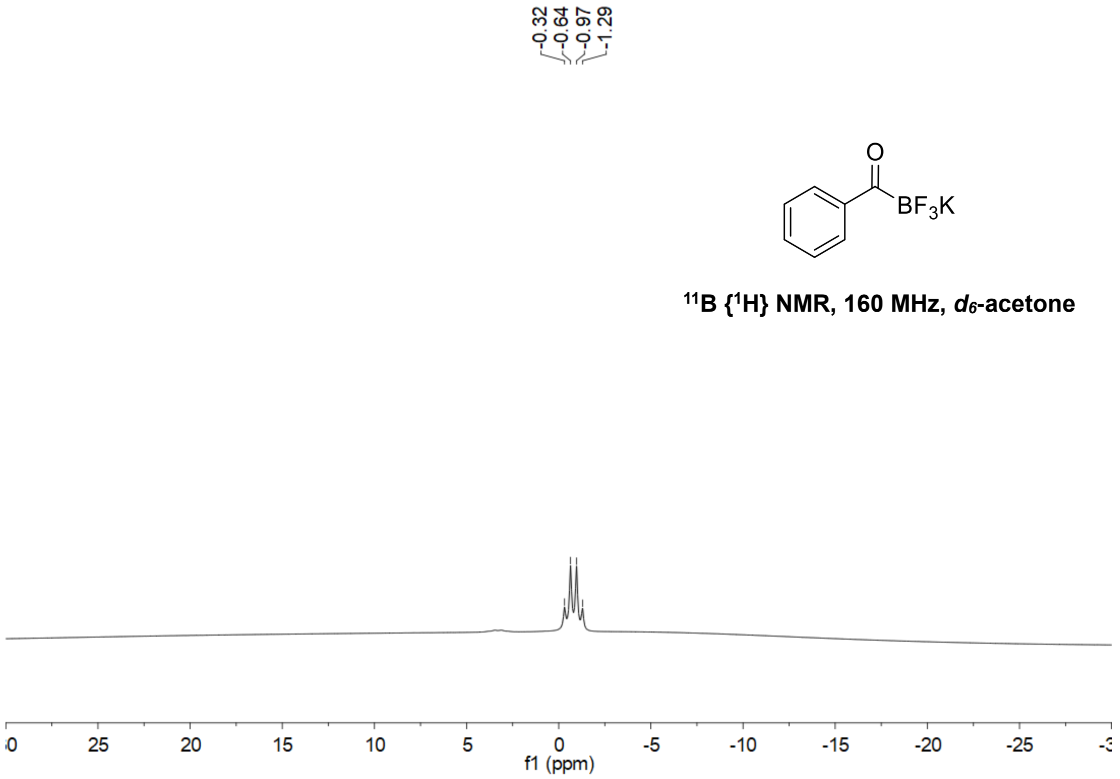
-0.32
-0.64
-0.97
-1.29
O
BF3K
11B {1H} NMR, 160 MHz, d6-acetone
25 20 15 10 5 0 -5 -10 -15 -20 -25
f1 (ppm)

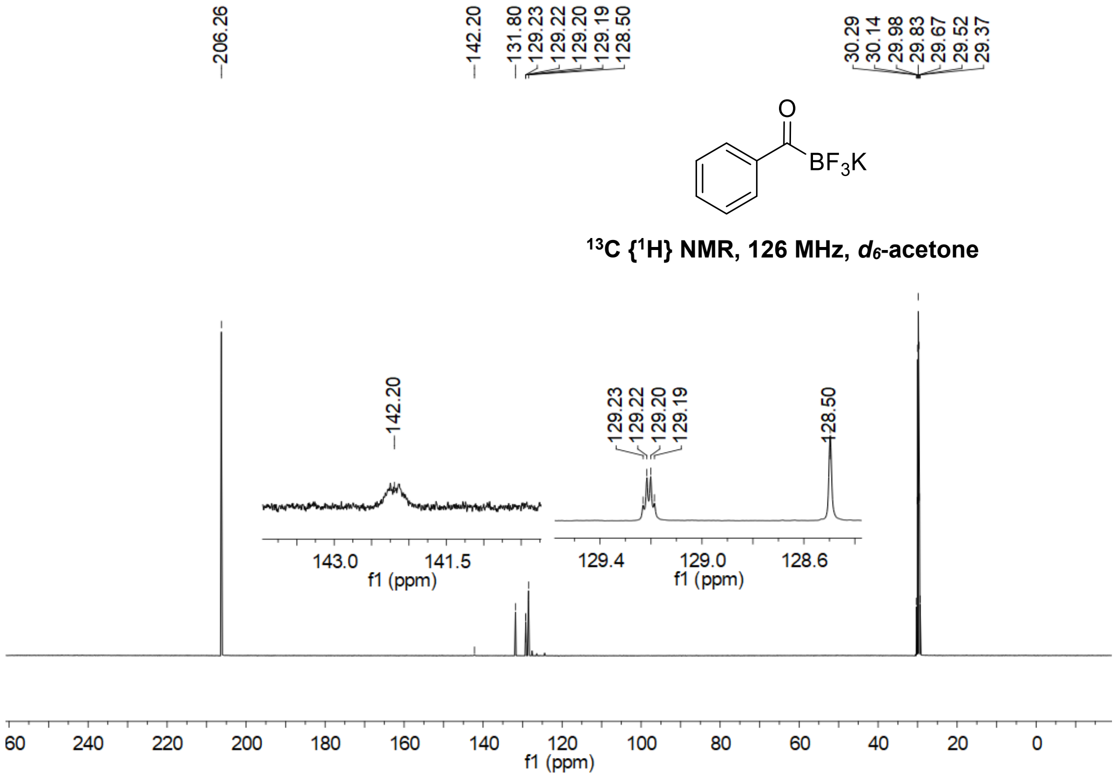
206.26
142.20
131.80
129.23
129.22
129.20
129.19
128.50
30.29
30.14
29.98
29.83
29.67
29.52
29.37
O
BF3K
13C {1H} NMR, 126 MHz, d6-acetone
f1 (ppm)

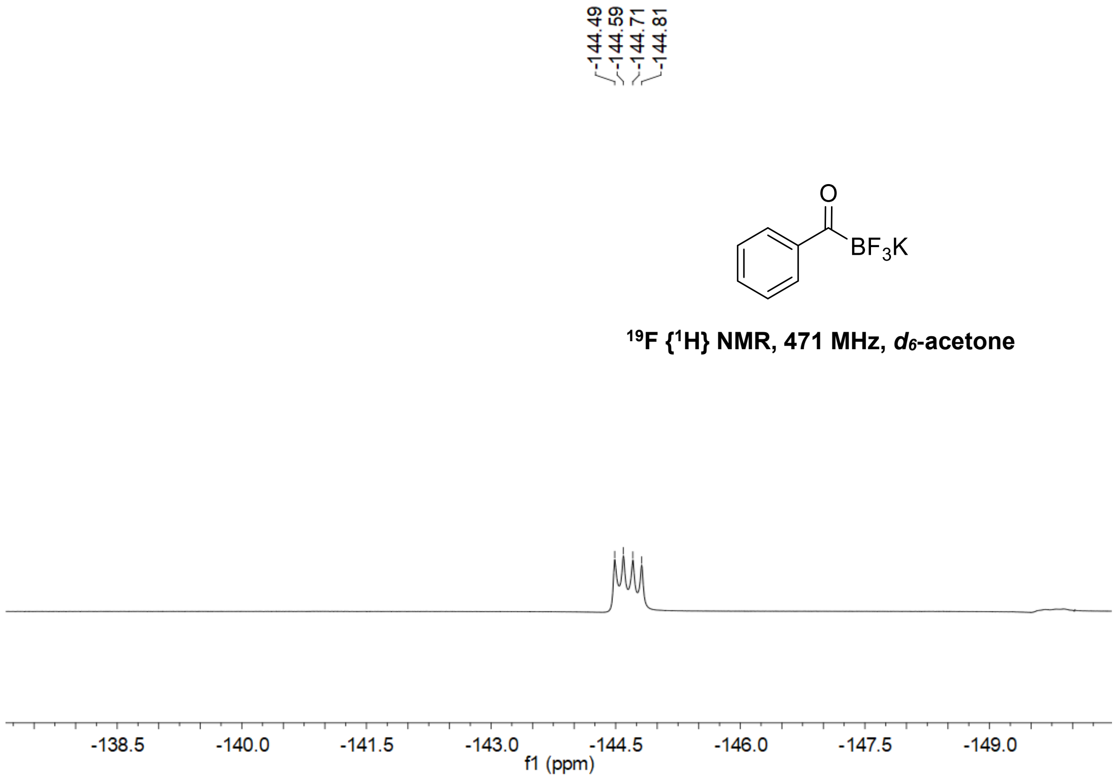
-144.49
-144.59
-144.71
-144.81
O
BF3K
19F {1H} NMR, 471 MHz, d6-acetone
f1 (ppm)

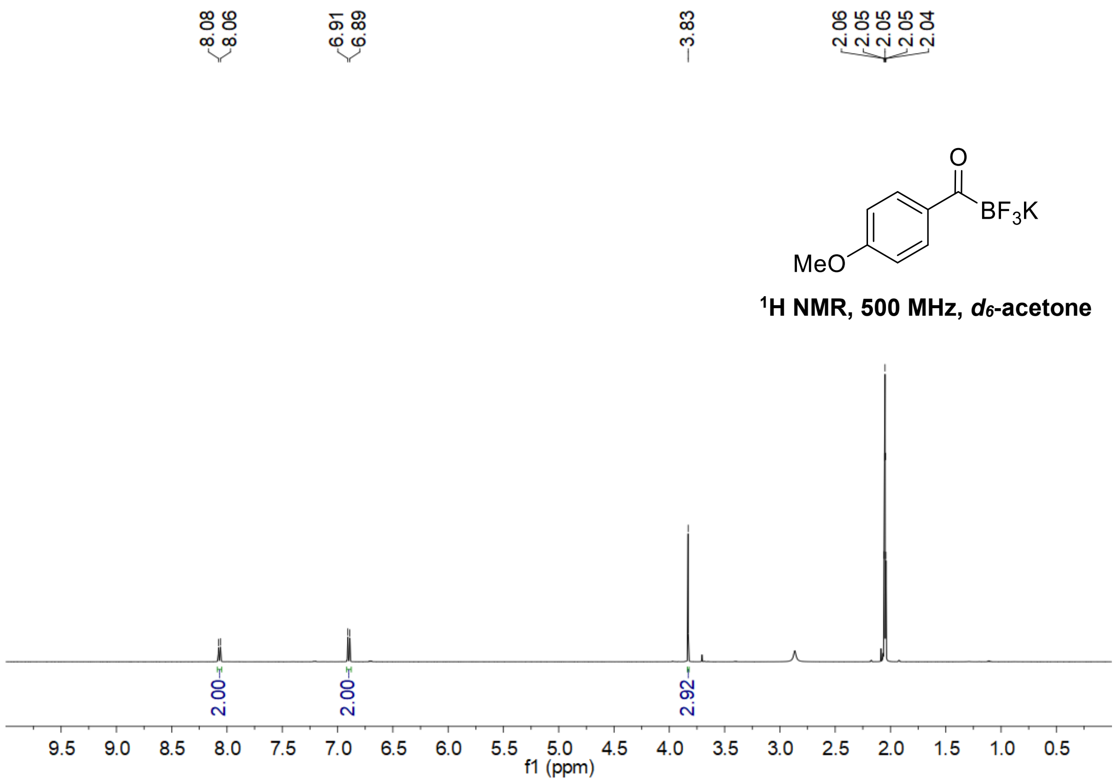
8.08
8.06
6.91
6.89
3.83
2.06
2.05
2.05
2.05
2.04
O
BF3K
MeO
1H NMR, 500 MHz, d6-acetone
2.00
2.00
2.92
9.5 9.0 8.5 8.0 7.5 7.0 6.5 6.0 5.5 5.0 4.5 4.0 3.5 3.0 2.5 2.0 1.5 1.0 0.5
f1 (ppm)

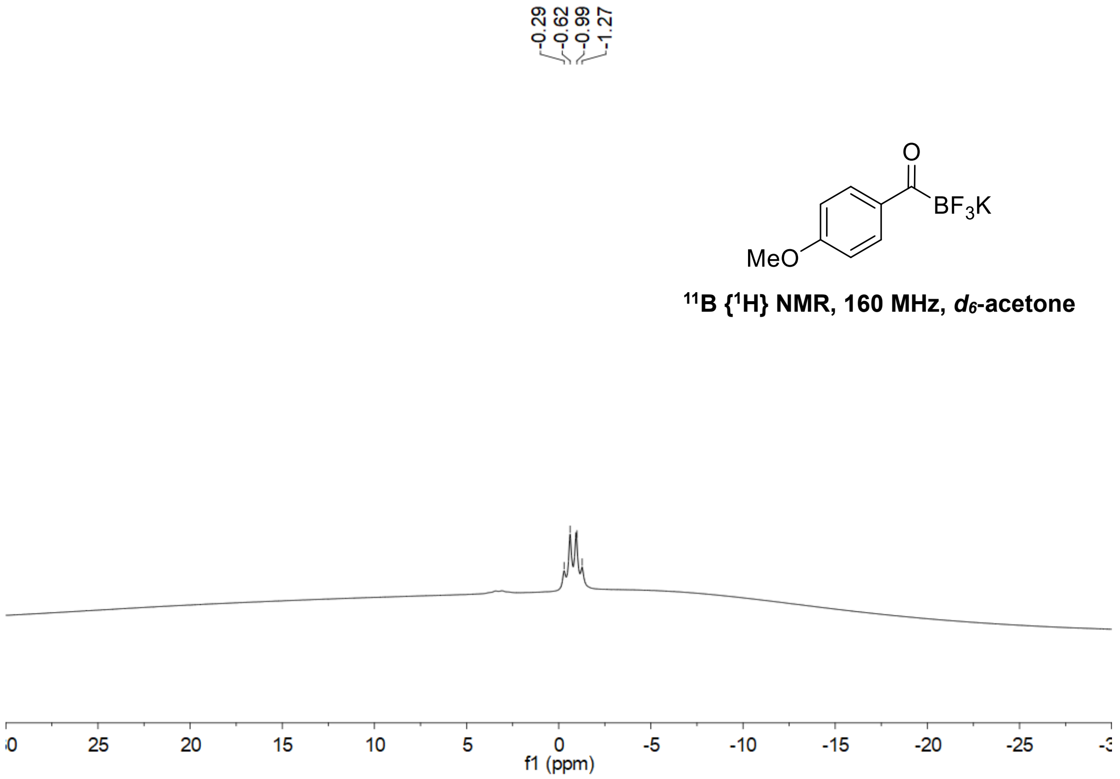
-0.29
-0.62
-0.99
-1.27
O
BF3K
MeO
11B {1H} NMR, 160 MHz, d6-acetone
25 20 15 10 5 0 -5 -10 -15 -20 -25
f1 (ppm)

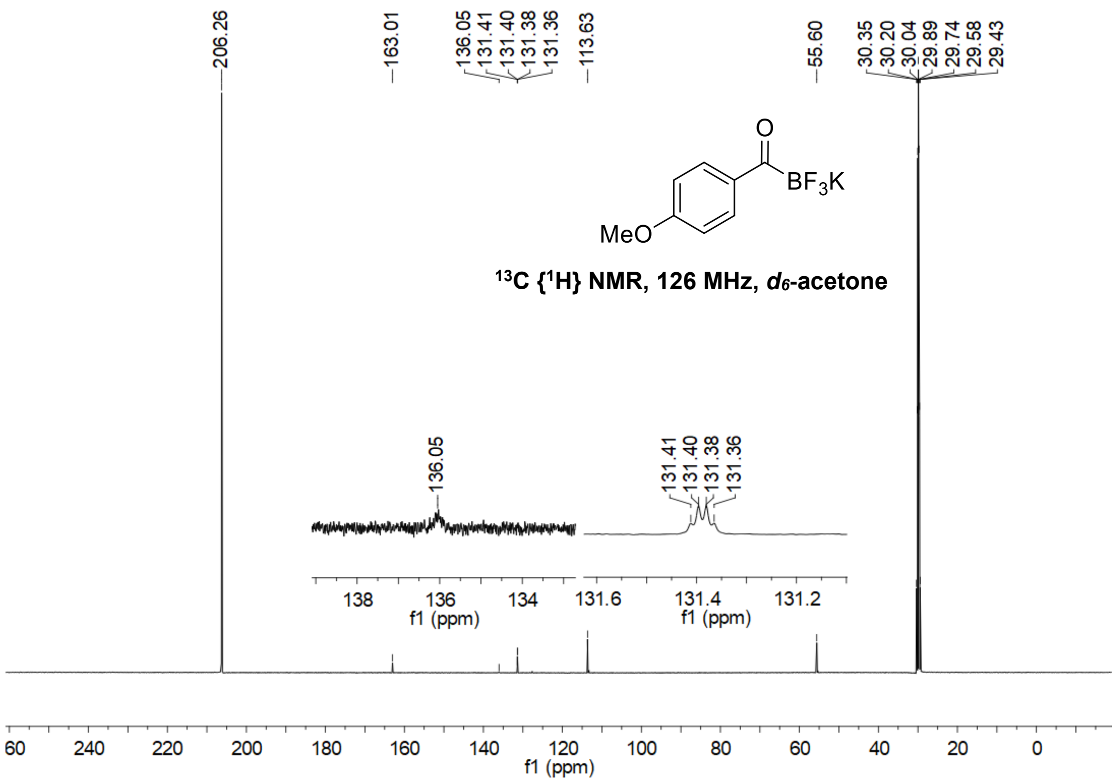
206.26
163.01
136.05
131.41
131.40
131.38
131.36
113.63
55.60
30.35
30.20
30.04
29.89
29.74
29.58
29.43
MeO
O
BF3K
13C {1H} NMR, 126 MHz, d6-acetone
138
136
134
131.6
131.4
131.2
f1 (ppm)
60
240
220
200
180
160
140
120
100
80
60
40
20
0
f1 (ppm)

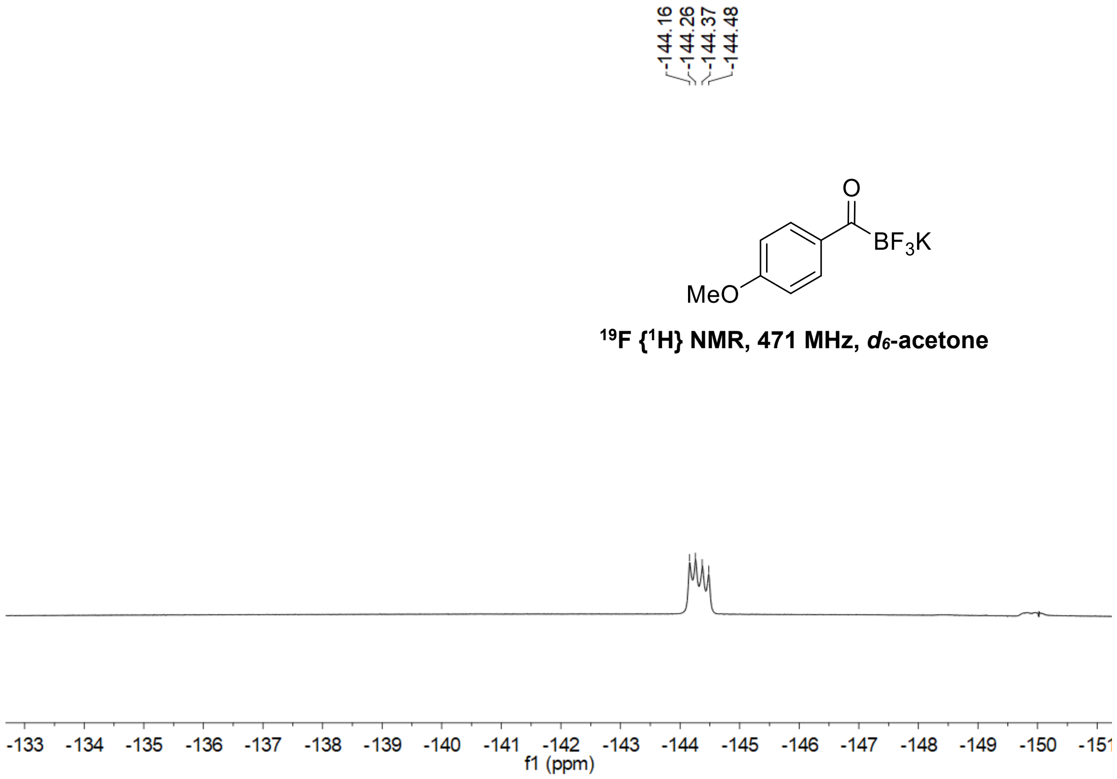
-144.16
-144.26
-144.37
-144.48
MeO
O
BF3K
19F {1H} NMR, 471 MHz, d6-acetone
-133
-134
-135
-136
-137
-138
-139
-140
-141
-142
-143
-144
-145
-146
-147
-148
-149
-150
-151
f1 (ppm)

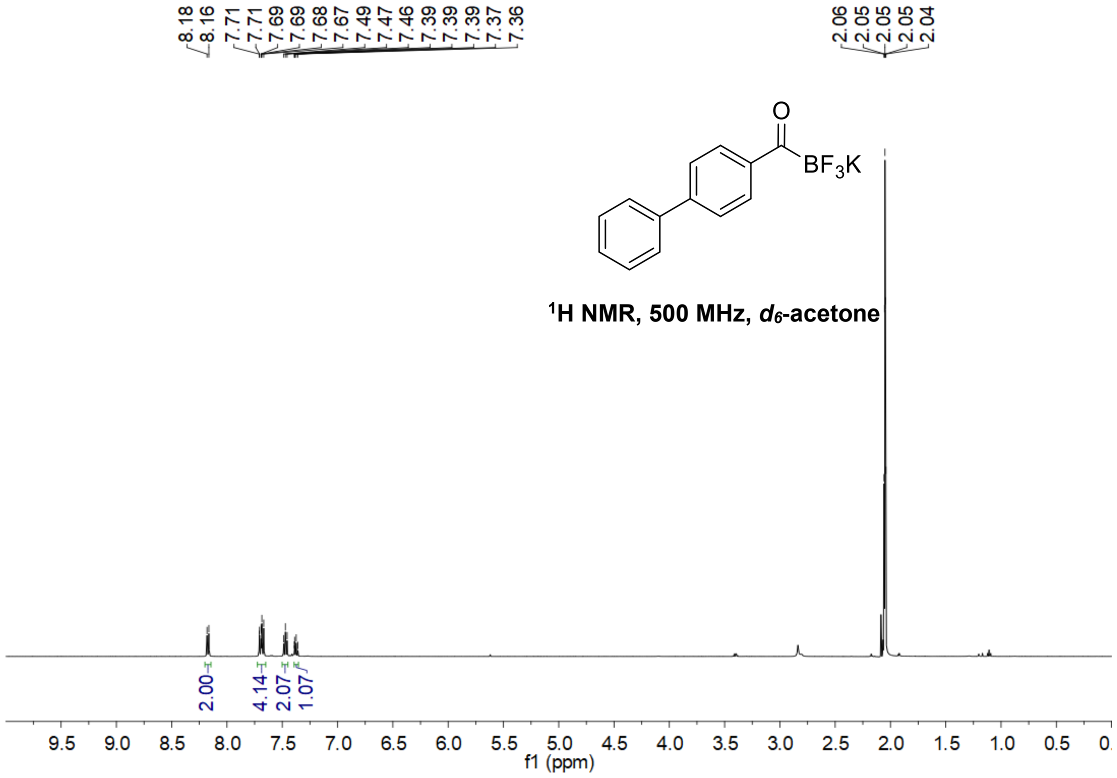
8.18
8.16
7.71
7.71
7.69
7.69
7.68
7.67
7.49
7.47
7.46
7.39
7.39
7.39
7.37
7.36
2.06
2.05
2.05
2.05
2.04
O
BF3K
1H NMR, 500 MHz, d6-acetone
2.00
4.14
2.07
1.07
9.5
9.0
8.5
8.0
7.5
7.0
6.5
6.0
5.5
5.0
4.5
4.0
3.5
3.0
2.5
2.0
1.5
1.0
0.5
f1 (ppm)

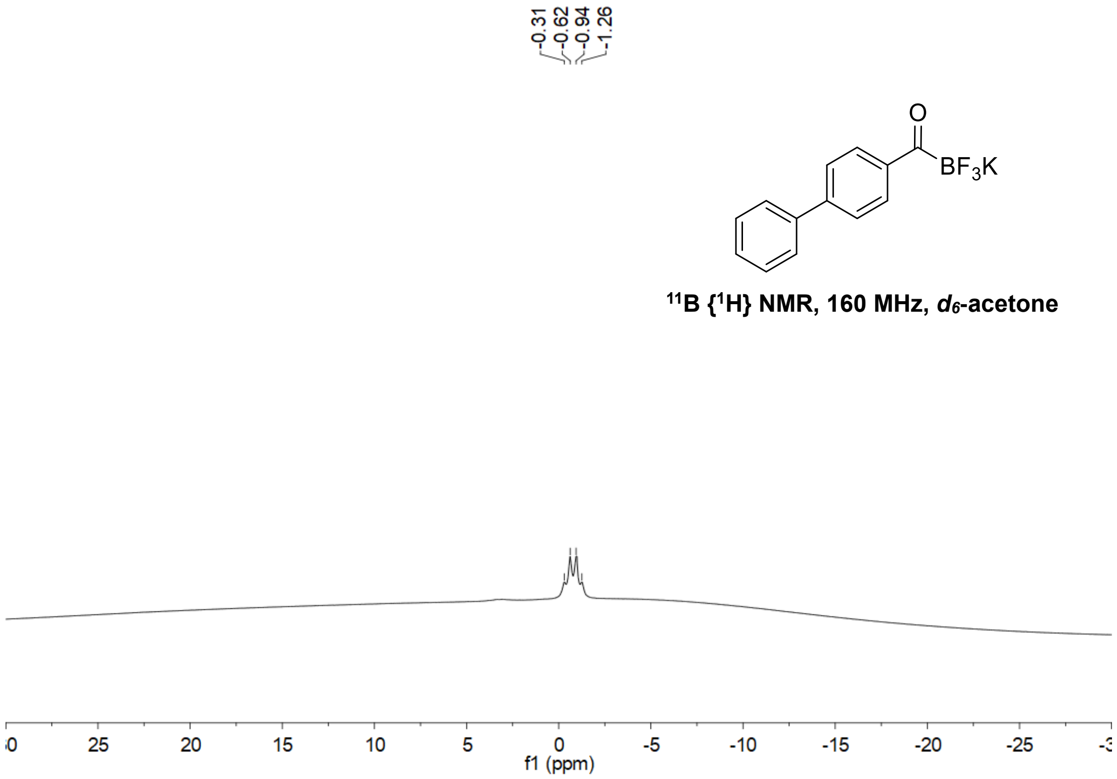
-0.31
-0.62
-0.94
-1.26
O
BF3K
11B {1H} NMR, 160 MHz, d6-acetone
25
20
15
10
5
0
-5
-10
-15
-20
-25
f1 (ppm)

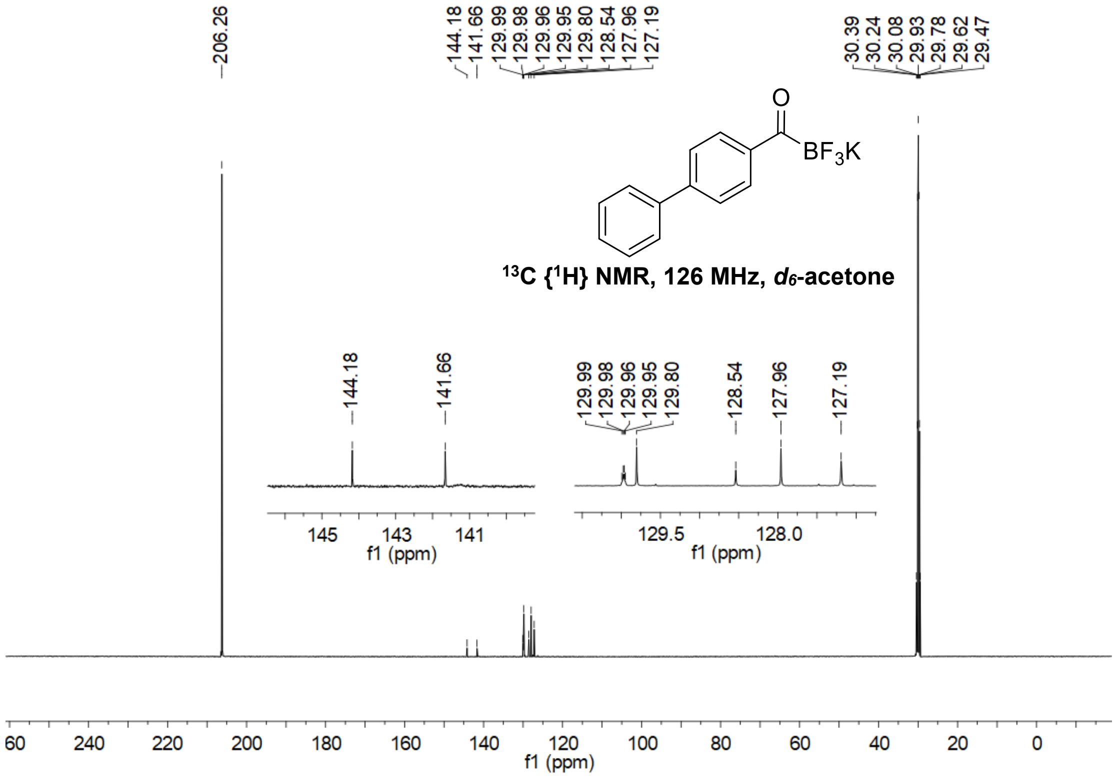
206.26
144.18
141.66
129.99
129.98
129.96
129.95
129.80
128.54
127.96
127.19
30.39
30.24
30.08
29.93
29.78
29.62
29.47
O
BF3K
13C {1H} NMR, 126 MHz, d6-acetone
145
143
141
f1 (ppm)
129.5
128.0
f1 (ppm)
60
240
220
200
180
160
140
120
100
80
60
40
20
0
f1 (ppm)

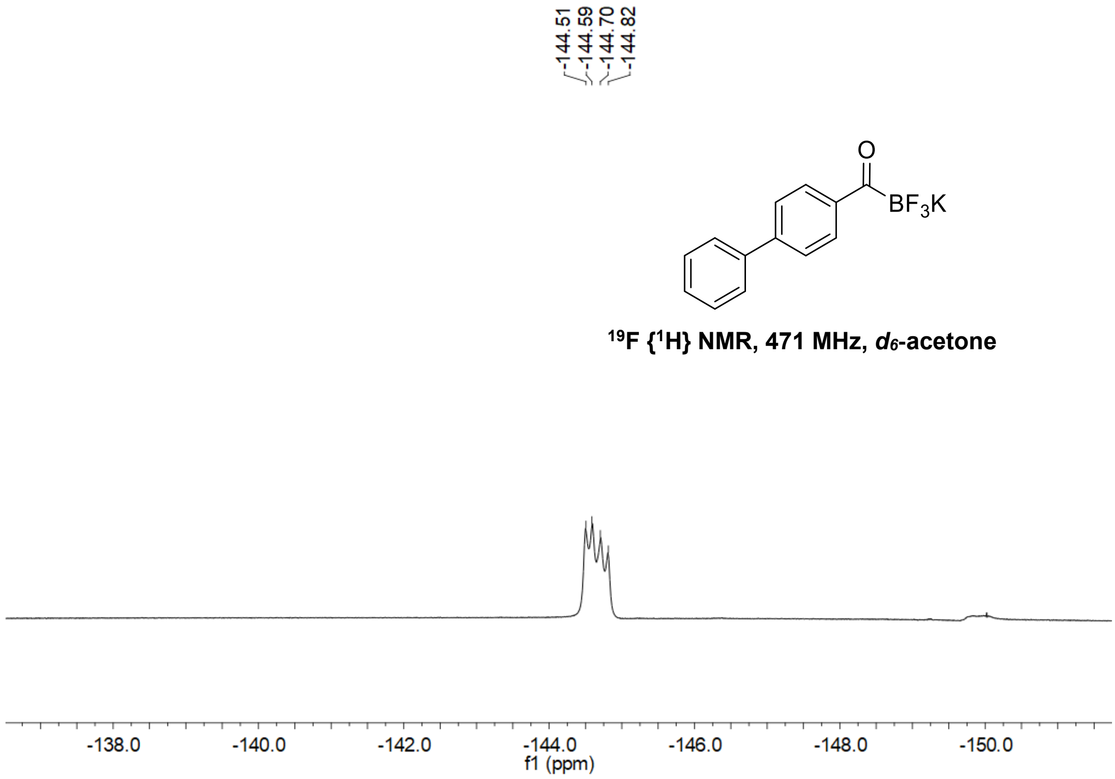
-144.51
-144.59
-144.70
-144.82
O
BF3K
19F {1H} NMR, 471 MHz, d6-acetone
-138.0
-140.0
-142.0
-144.0
-146.0
-148.0
-150.0
f1 (ppm)

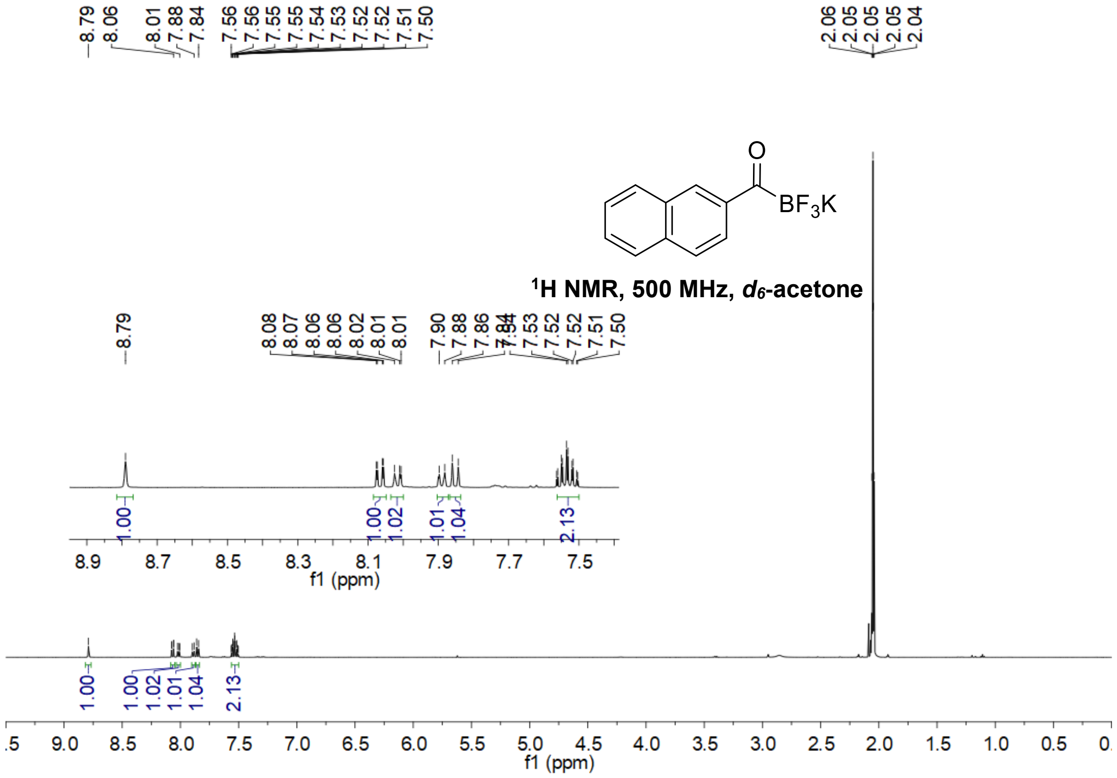
BF3K
O
1H NMR, 500 MHz, d6-acetone
f1 (ppm)

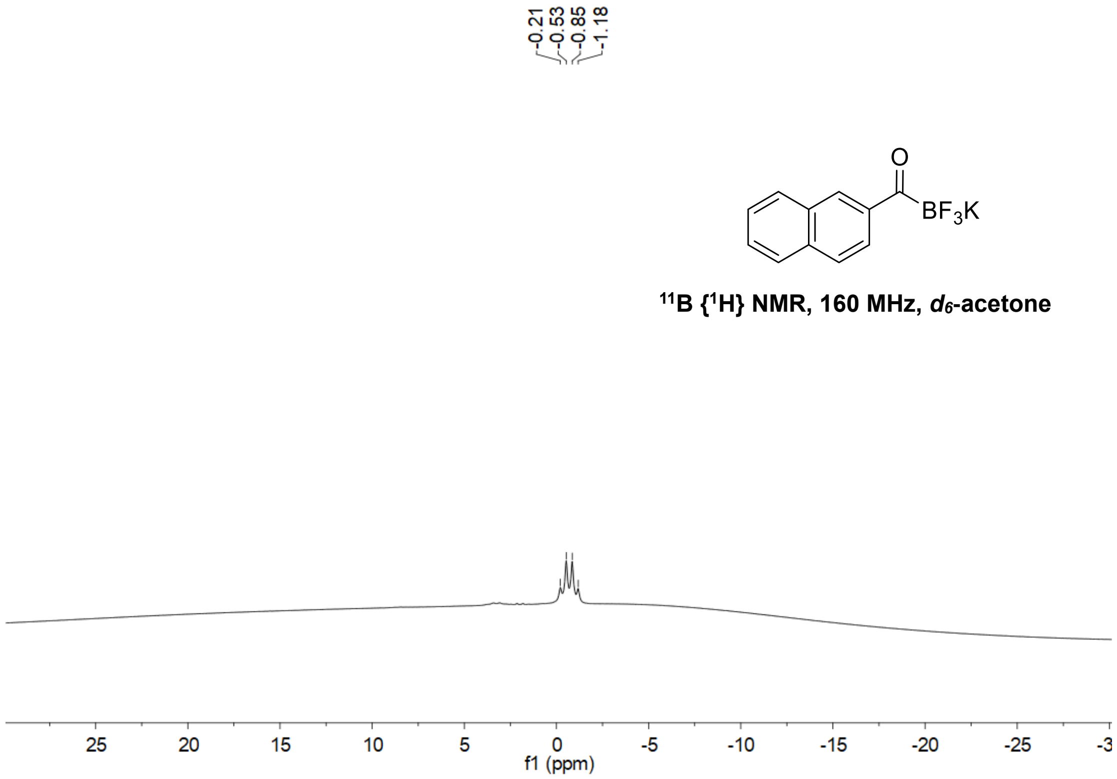
BF3K
O
11B {1H} NMR, 160 MHz, d6-acetone
f1 (ppm)

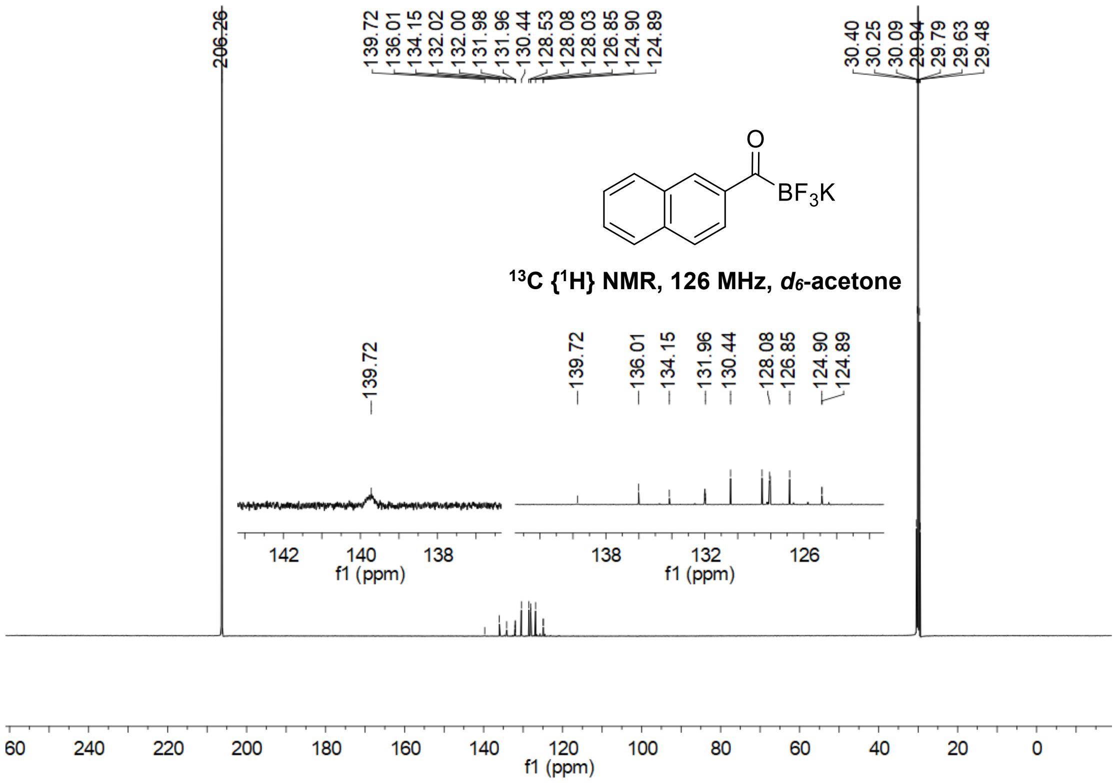
206.26
139.72
136.01
134.15
132.02
132.00
131.98
131.96
130.44
128.53
128.08
128.03
126.85
124.90
124.89
30.40
30.25
30.09
29.94
29.79
29.63
29.48
O
BF3K
13C {1H} NMR, 126 MHz, d6-acetone
142
140
138
f1 (ppm)
126
132
f1 (ppm)
60
240
220
200
180
160
140
120
100
80
60
40
20
0
f1 (ppm)

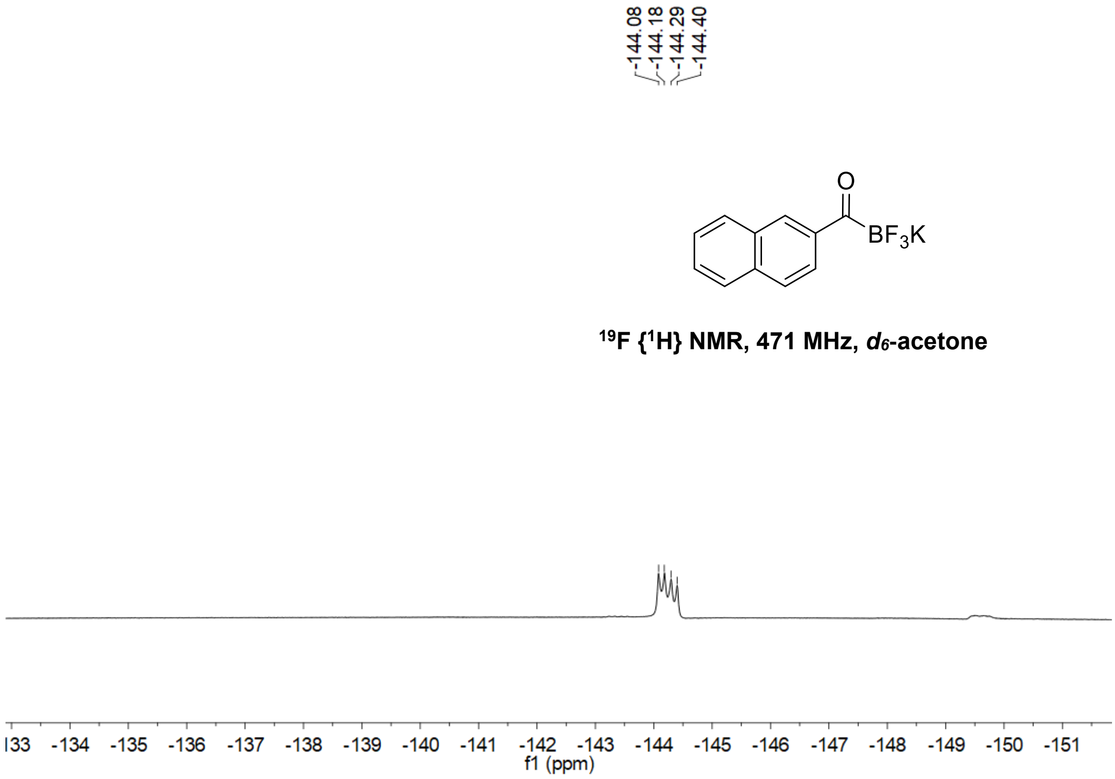
-144.08
-144.18
-144.29
-144.40
O
BF3K
19F {1H} NMR, 471 MHz, d6-acetone
133
-134
-135
-136
-137
-138
-139
-140
-141
-142
-143
-144
-145
-146
-147
-148
-149
-150
-151
f1 (ppm)

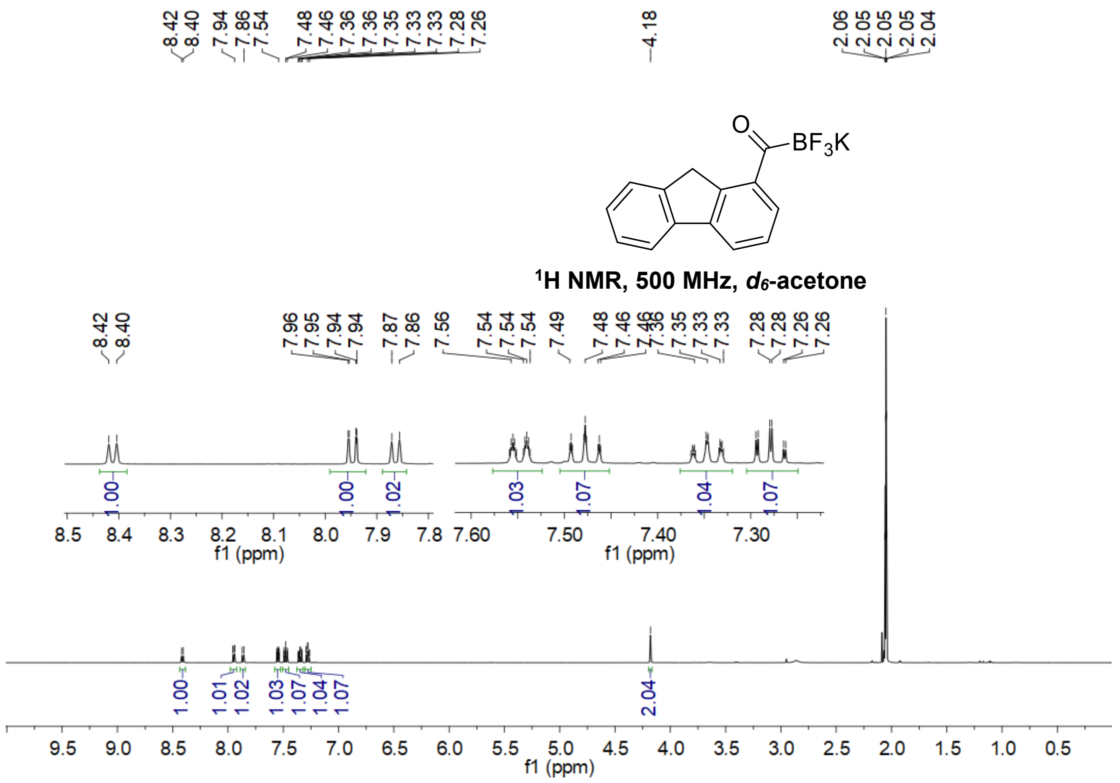

^{1}H NMR, 500 MHz, *d*$_6$-acetone

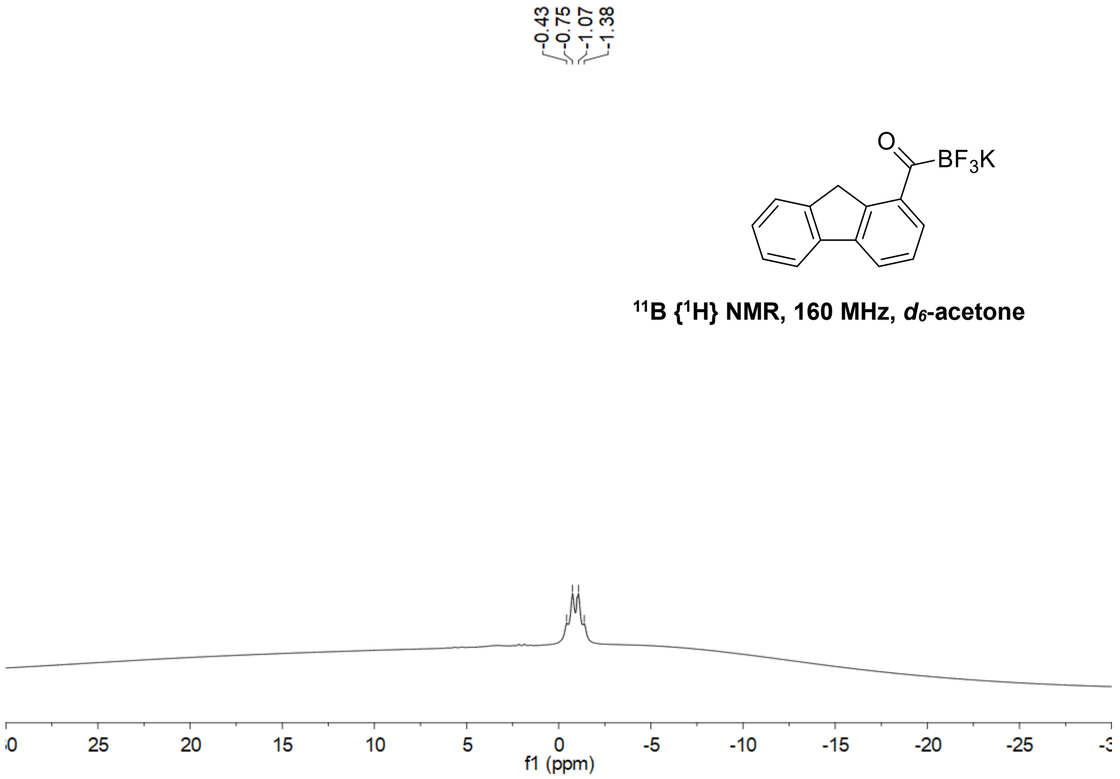

^{11}B {^{1}H} NMR, 160 MHz, *d*$_6$-acetone

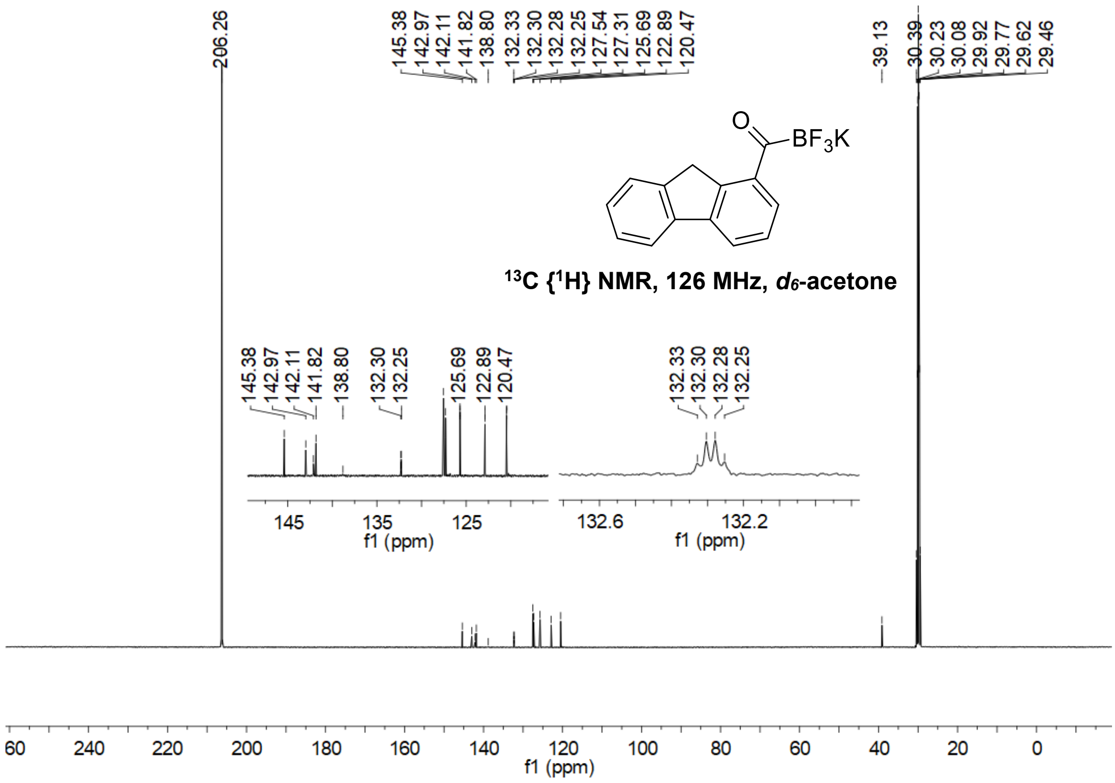
206.26
145.38
142.97
142.11
141.82
138.80
132.33
132.30
132.28
132.25
127.54
127.31
125.69
122.89
120.47
39.13
30.39
30.23
30.08
29.92
29.77
29.62
29.46
O
BF3K
13C {1H} NMR, 126 MHz, d6-acetone
f1 (ppm)

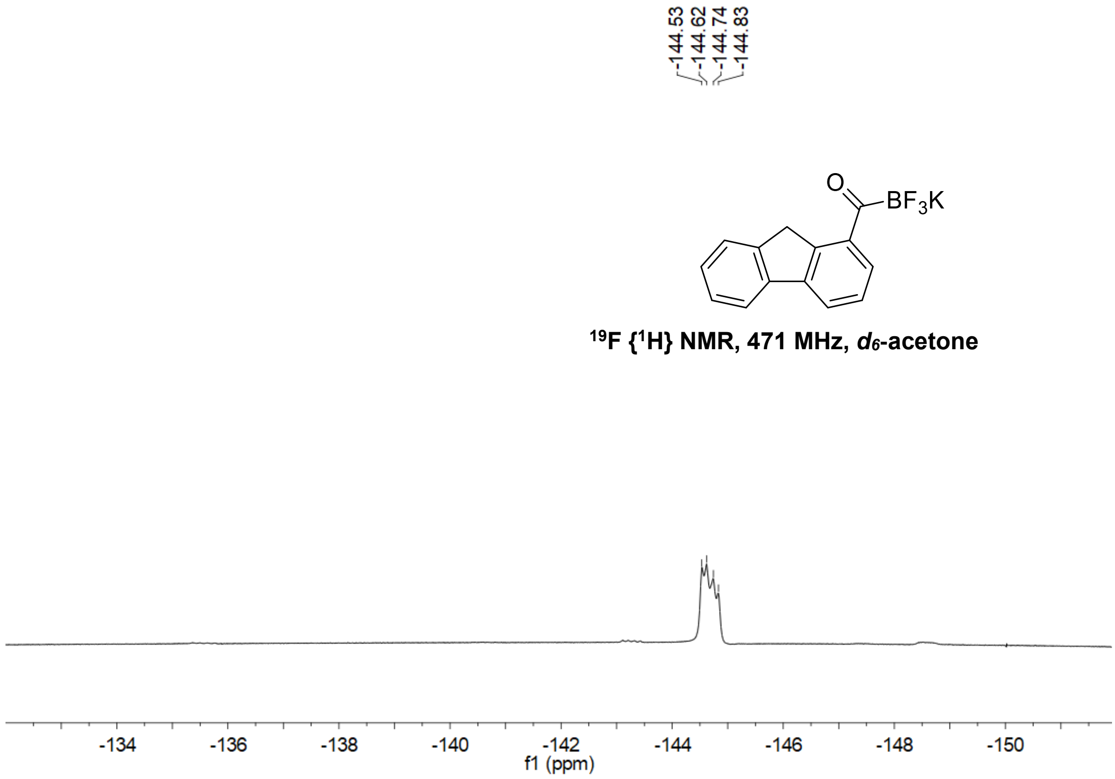
-144.53
-144.62
-144.74
-144.83
O
BF3K
19F {1H} NMR, 471 MHz, d6-acetone
f1 (ppm)

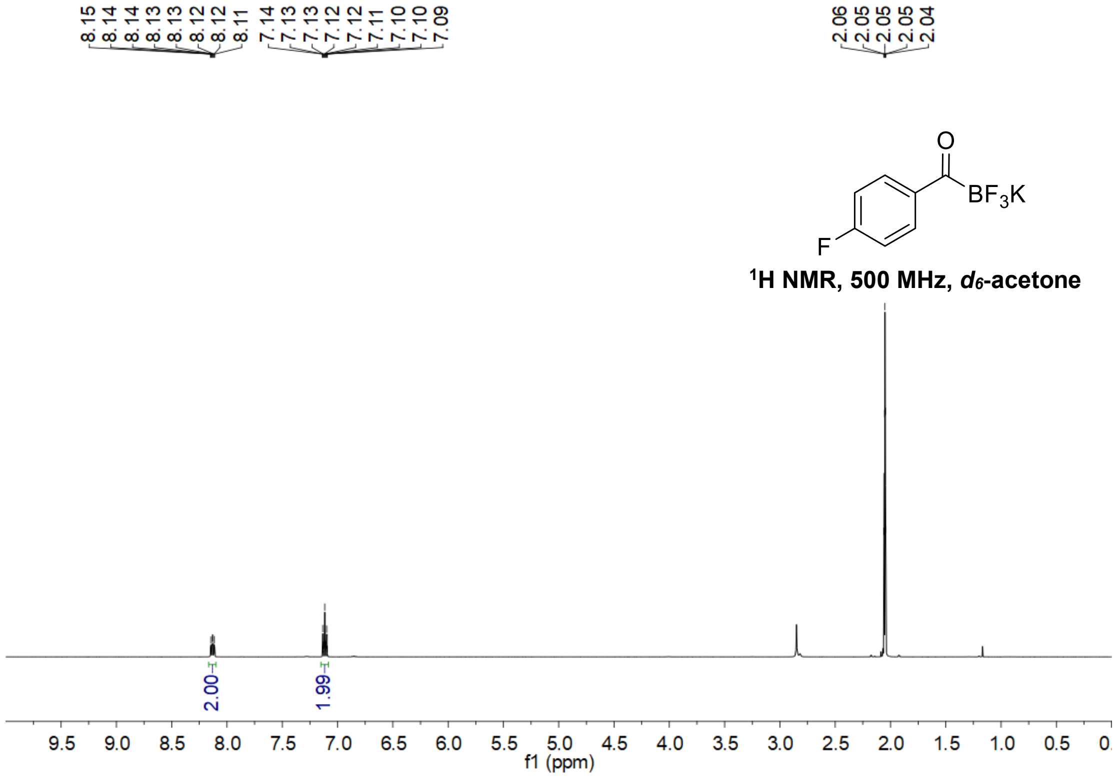
8.15
8.14
8.14
8.13
8.13
8.12
8.12
8.11
7.14
7.13
7.13
7.12
7.12
7.11
7.10
7.10
7.09
2.06
2.05
2.05
2.05
2.04
O
BF3K
F
1H NMR, 500 MHz, d6-acetone
2.00
1.99
f1 (ppm)

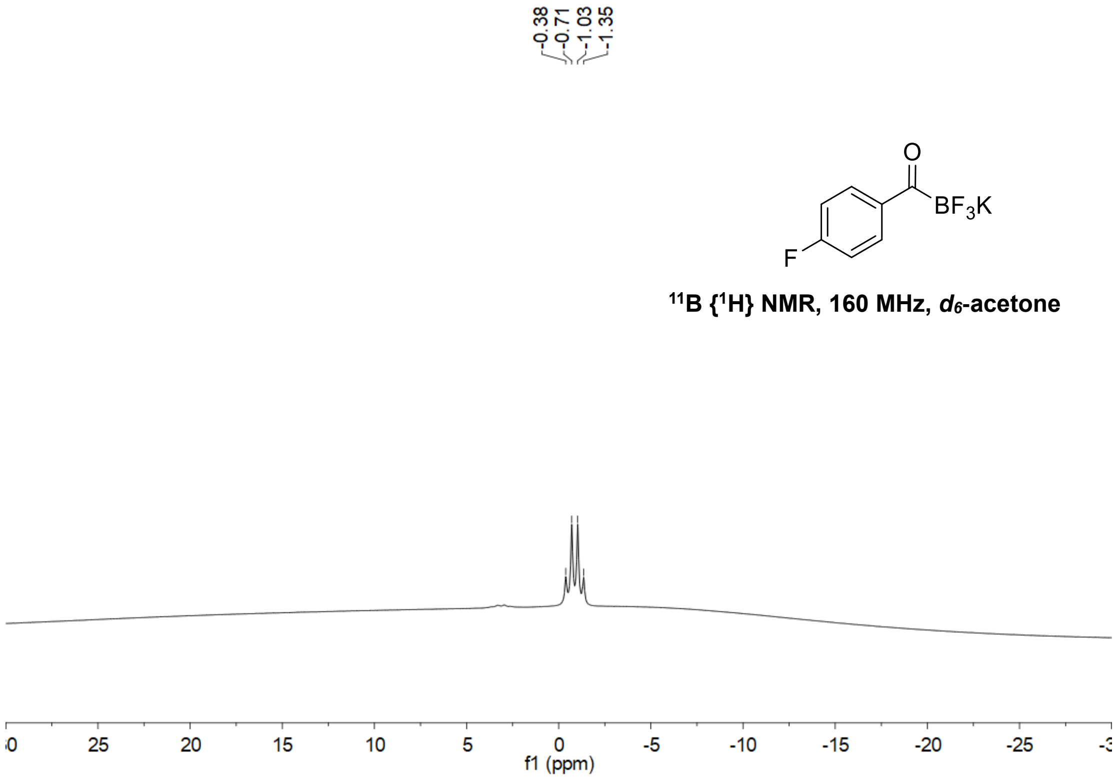
-0.38
-0.71
-1.03
-1.35
O
BF3K
F
11B {1H} NMR, 160 MHz, d6-acetone
f1 (ppm)

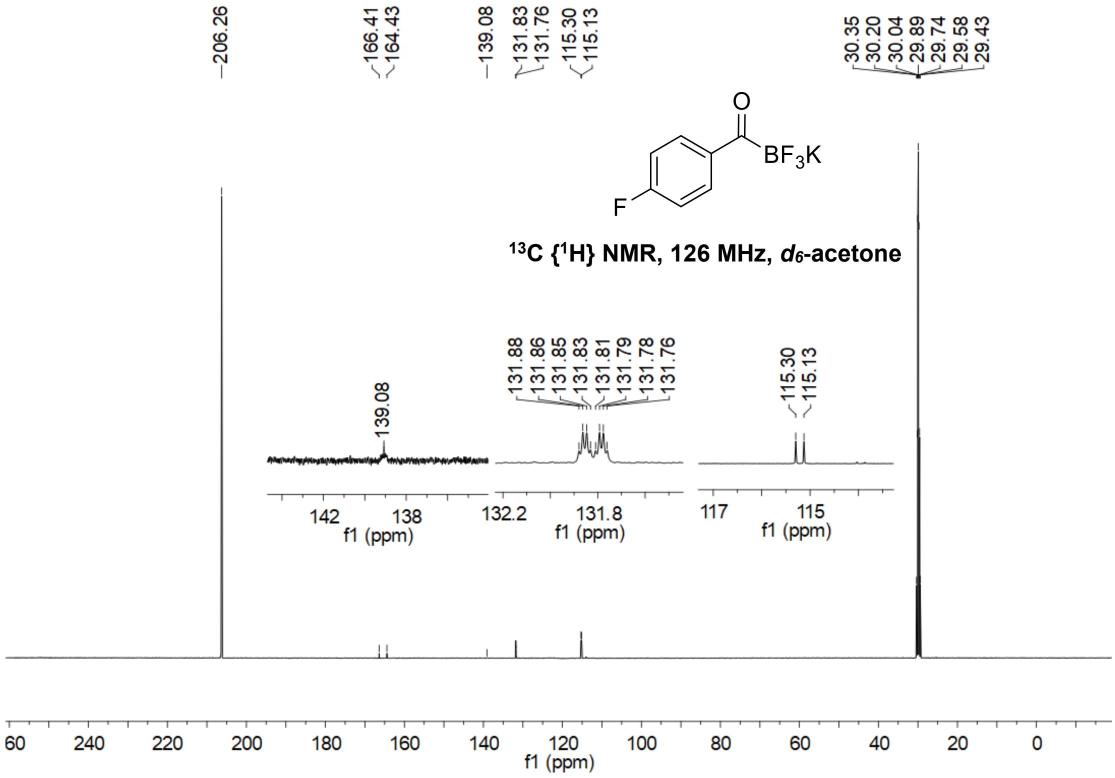
206.26
166.41
164.43
139.08
131.83
131.76
115.30
115.13
30.35
30.20
30.04
29.89
29.74
29.58
29.43
O
BF3K
F
13C {1H} NMR, 126 MHz, d6-acetone
131.88
131.86
131.85
131.83
131.81
131.79
131.78
131.76
142
138
f1 (ppm)
132.2
131.8
f1 (ppm)
117
115
f1 (ppm)
60
240
220
200
180
160
140
120
100
80
60
40
20
0
f1 (ppm)

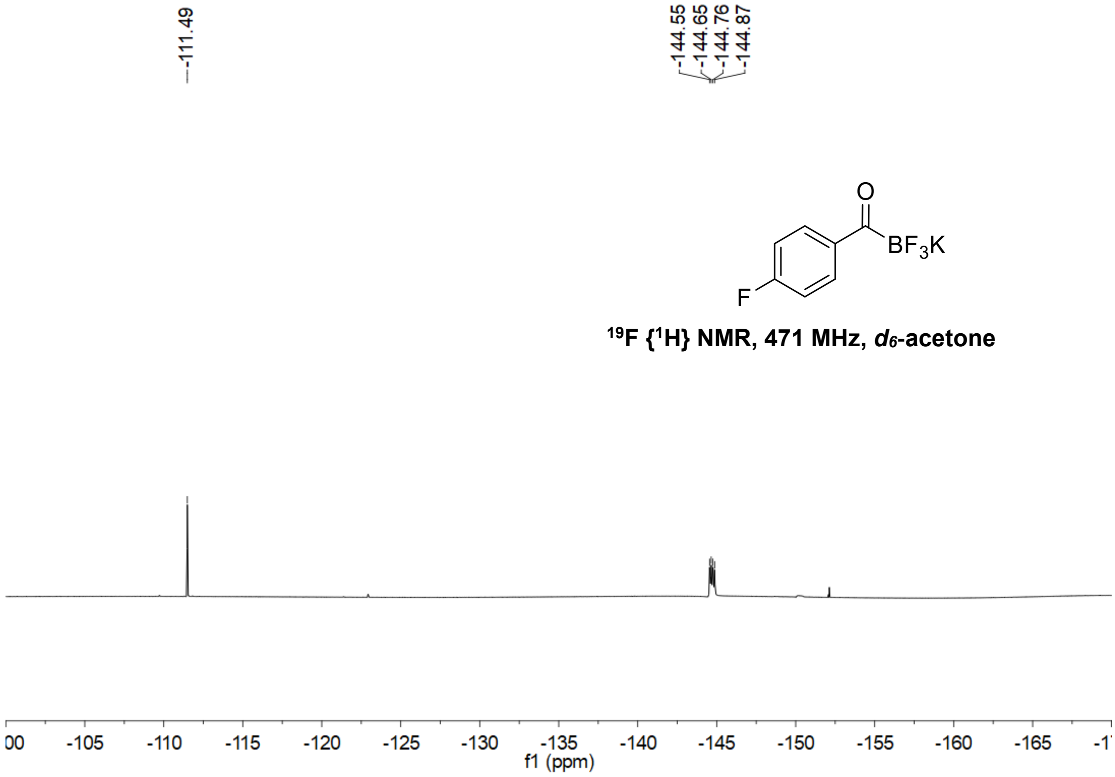
-111.49
-144.55
-144.65
-144.76
-144.87
O
BF3K
F
19F {1H} NMR, 471 MHz, d6-acetone
00
-105
-110
-115
-120
-125
-130
-135
-140
-145
-150
-155
-160
-165
f1 (ppm)

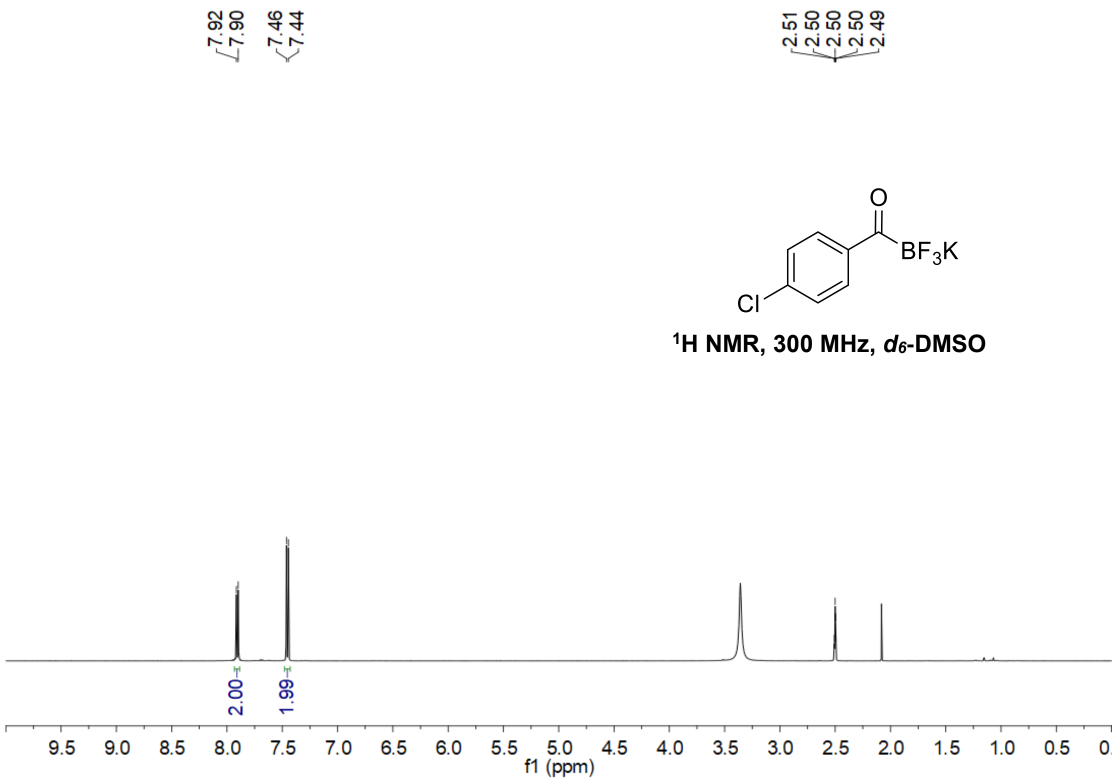
7.92
7.90
7.46
7.44
2.51
2.50
2.50
2.50
2.49
O
BF3K
Cl
1H NMR, 300 MHz, d6-DMSO
2.00
1.99
9.5 9.0 8.5 8.0 7.5 7.0 6.5 6.0 5.5 5.0 4.5 4.0 3.5 3.0 2.5 2.0 1.5 1.0 0.5 0.
f1 (ppm)

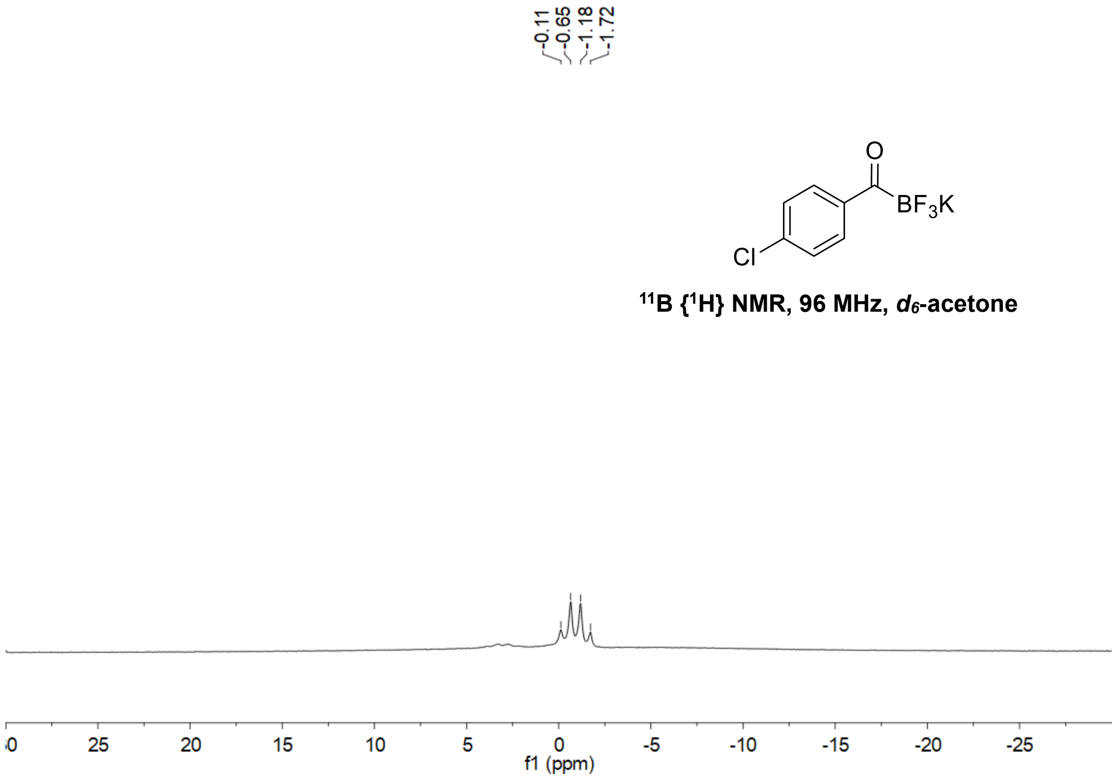
-0.11
-0.65
-1.18
-1.72
O
BF3K
Cl
11B {1H} NMR, 96 MHz, d6-acetone
25 20 15 10 5 0 -5 -10 -15 -20 -25
f1 (ppm)

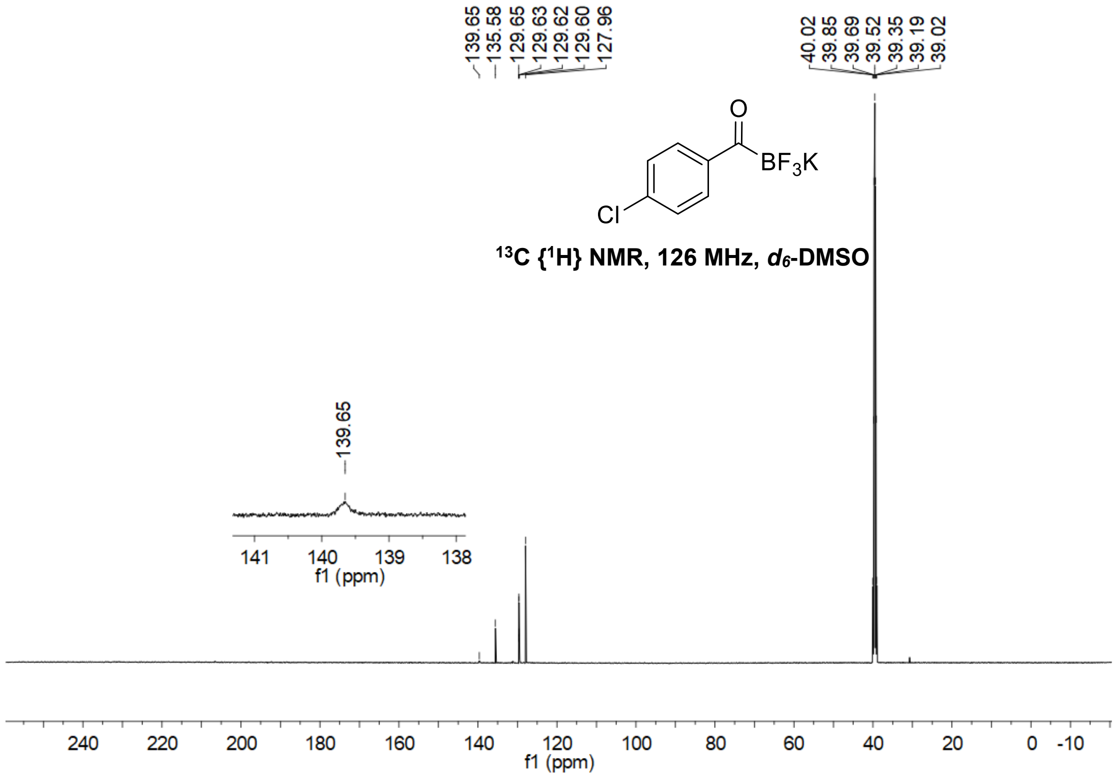
139.65
135.58
129.65
129.63
129.62
129.60
127.96
40.02
39.85
39.69
39.52
39.35
39.19
39.02
O
BF3K
Cl
13C {1H} NMR, 126 MHz, d6-DMSO
139.65
141
140
139
138
f1 (ppm)
240
220
200
180
160
140
120
100
80
60
40
20
0
-10
f1 (ppm)

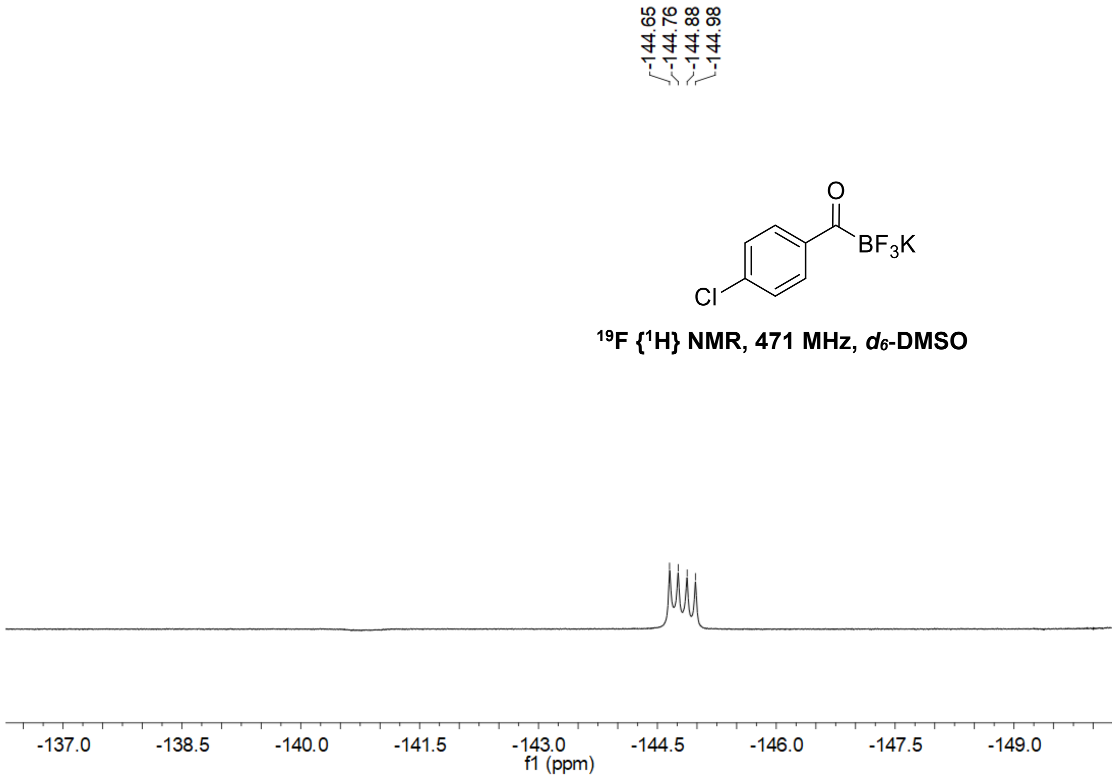
-144.65
-144.76
-144.88
-144.98
O
BF3K
Cl
19F {1H} NMR, 471 MHz, d6-DMSO
-137.0
-138.5
-140.0
-141.5
-143.0
-144.5
-146.0
-147.5
-149.0
f1 (ppm)

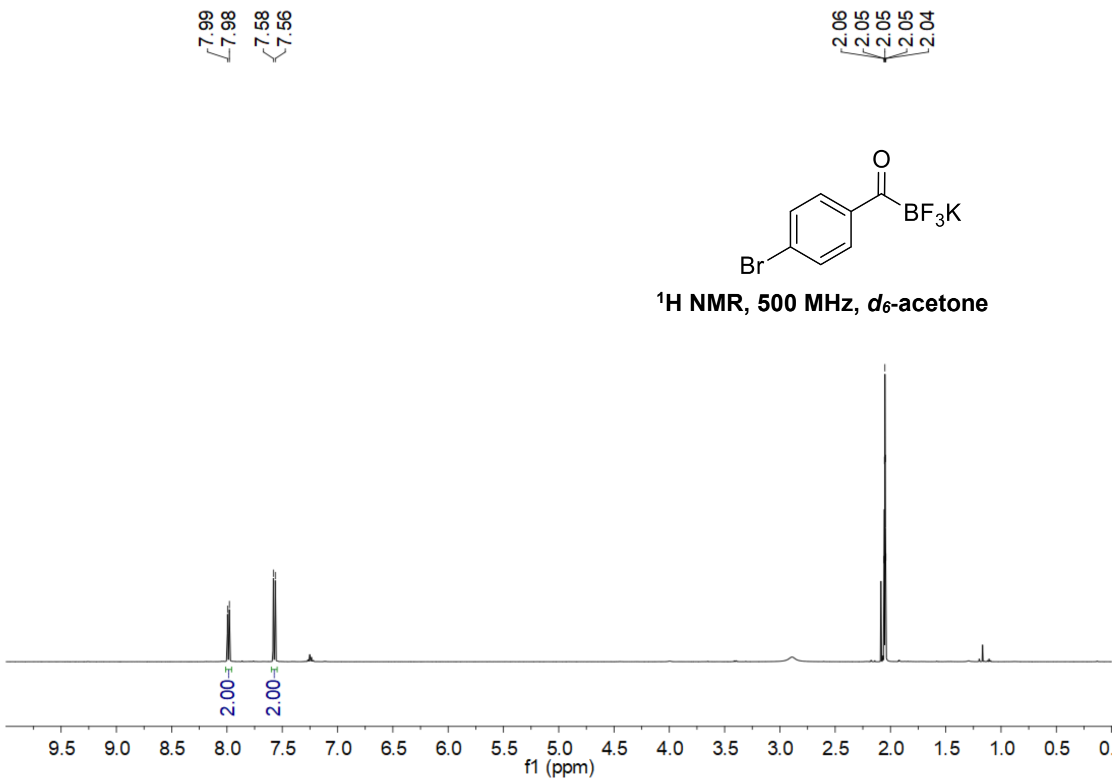
7.99
7.98
7.58
7.56
2.06
2.05
2.05
2.05
2.04
O
BF3K
Br
1H NMR, 500 MHz, d6-acetone
2.00
2.00
9.5 9.0 8.5 8.0 7.5 7.0 6.5 6.0 5.5 5.0 4.5 4.0 3.5 3.0 2.5 2.0 1.5 1.0 0.5
f1 (ppm)

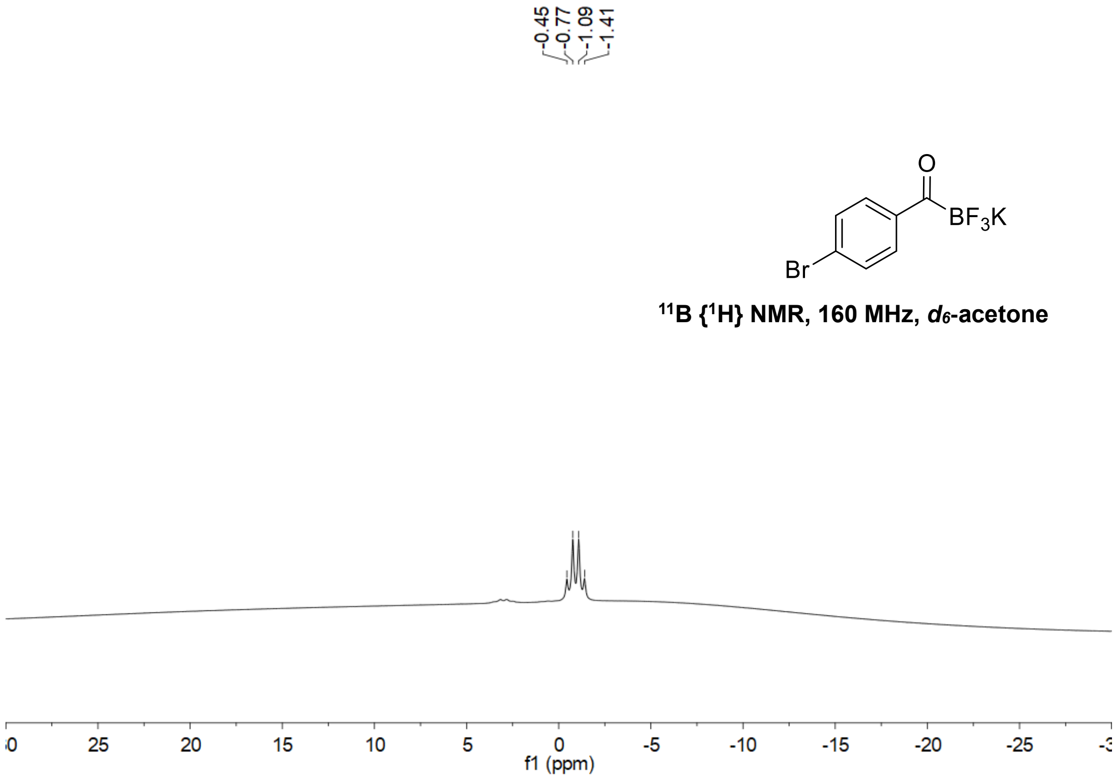
-0.45
-0.77
-1.09
-1.41
O
BF3K
Br
11B {1H} NMR, 160 MHz, d6-acetone
25 20 15 10 5 0 -5 -10 -15 -20 -25
f1 (ppm)

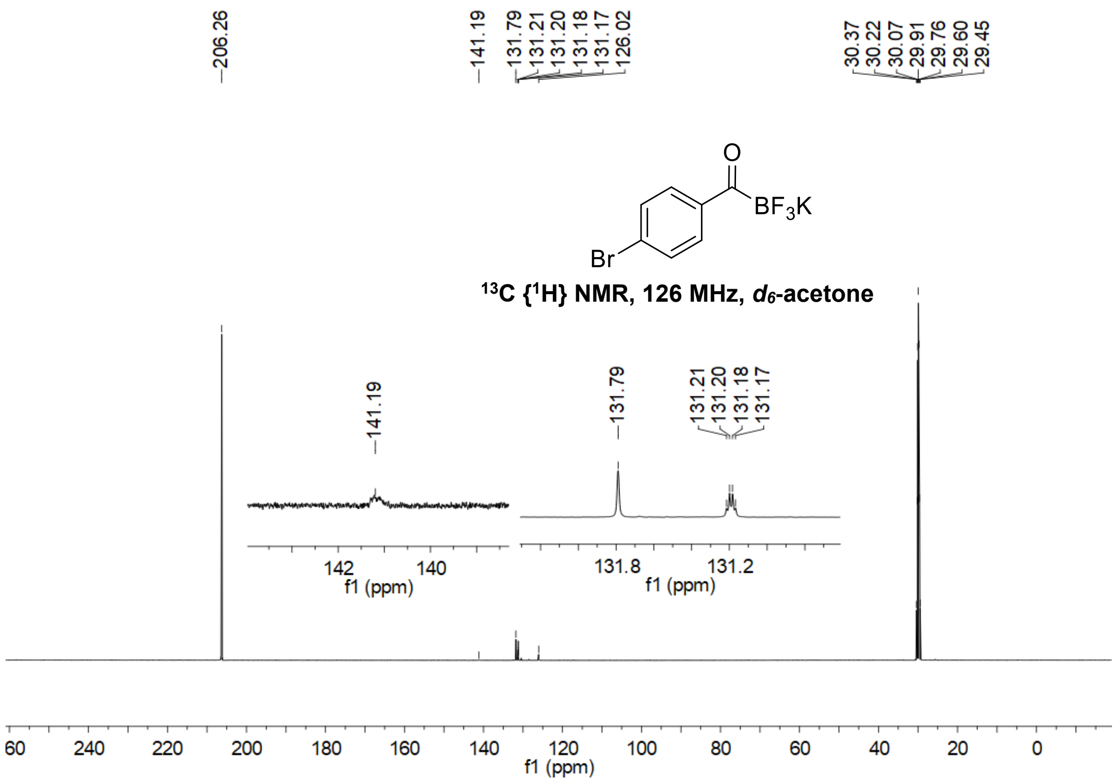
206.26
141.19
131.79
131.21
131.20
131.18
131.17
126.02
30.37
30.22
30.07
29.91
29.76
29.60
29.45
O
BF3K
Br
13C {1H} NMR, 126 MHz, d6-acetone
142
140
f1 (ppm)
131.8
131.2
f1 (ppm)
60
240
220
200
180
160
140
120
100
80
60
40
20
0
f1 (ppm)

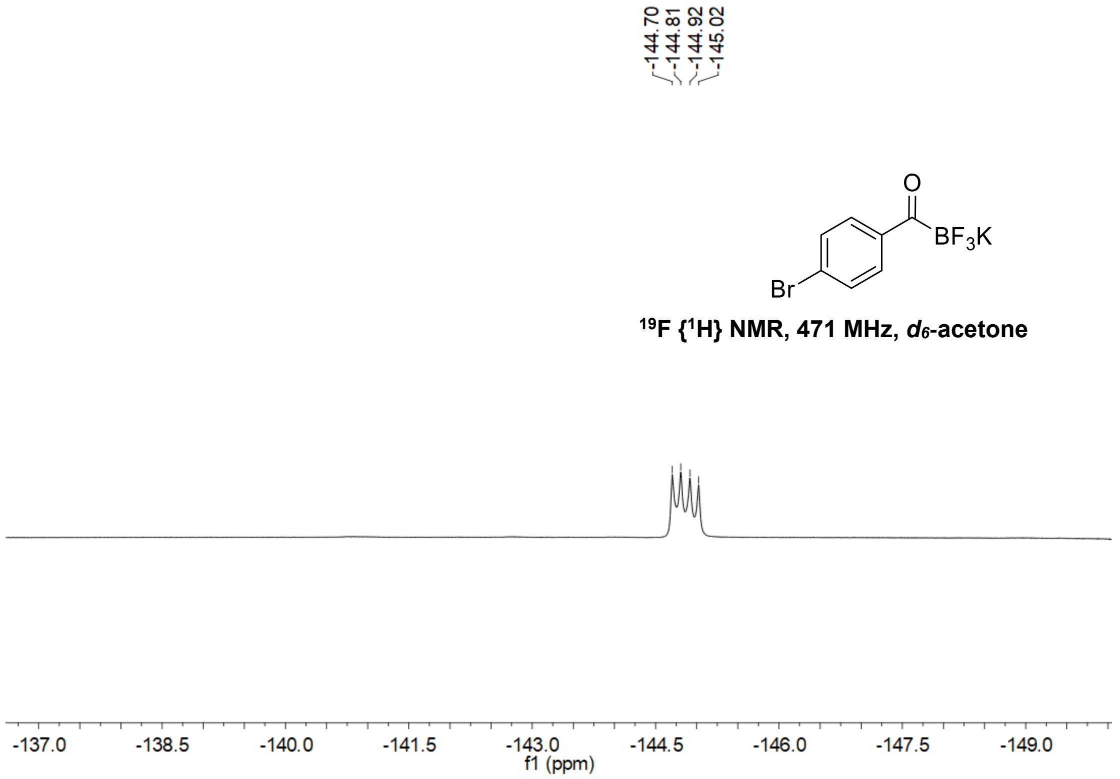
-144.70
-144.81
-144.92
-145.02
O
BF3K
Br
19F {1H} NMR, 471 MHz, d6-acetone
-137.0
-138.5
-140.0
-141.5
-143.0
-144.5
-146.0
-147.5
-149.0
f1 (ppm)

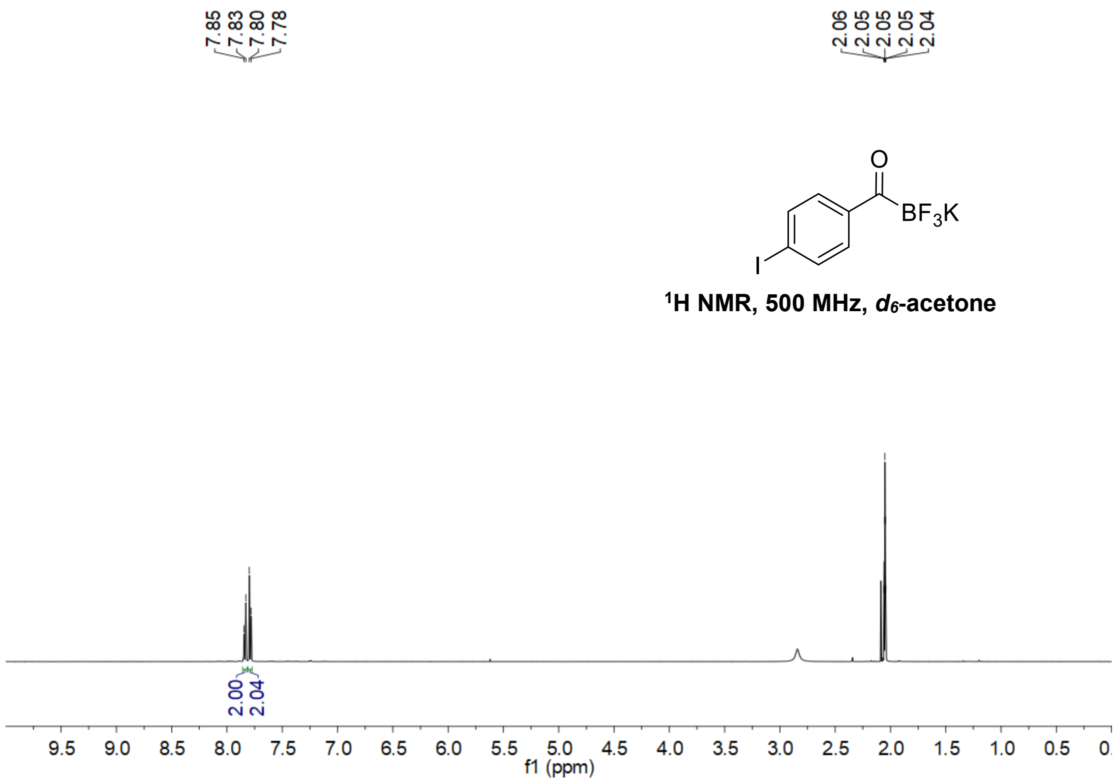
7.85
7.83
7.80
7.78
2.06
2.05
2.05
2.05
2.04
O
BF3K
I
1H NMR, 500 MHz, d6-acetone
2.00
2.04
9.5
9.0
8.5
8.0
7.5
7.0
6.5
6.0
5.5
5.0
4.5
4.0
3.5
3.0
2.5
2.0
1.5
1.0
0.5
f1 (ppm)

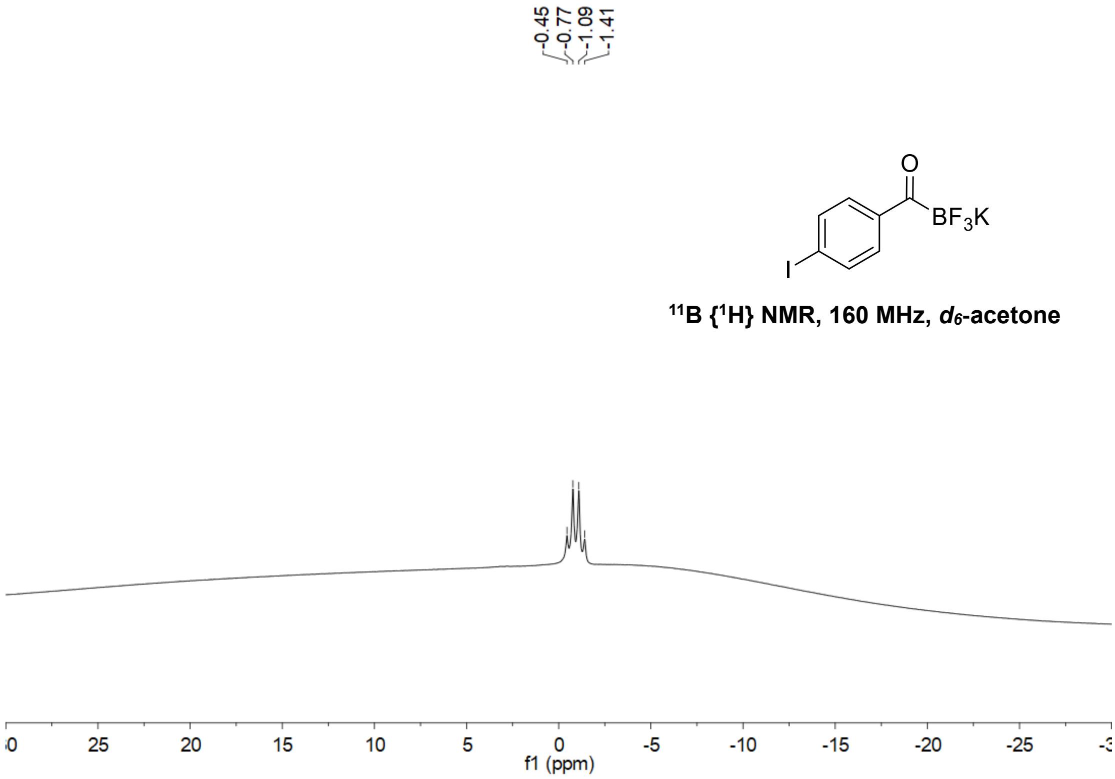
-0.45
-0.77
-1.09
-1.41
O
BF3K
I
11B {1H} NMR, 160 MHz, d6-acetone
25
20
15
10
5
0
-5
-10
-15
-20
-25
f1 (ppm)

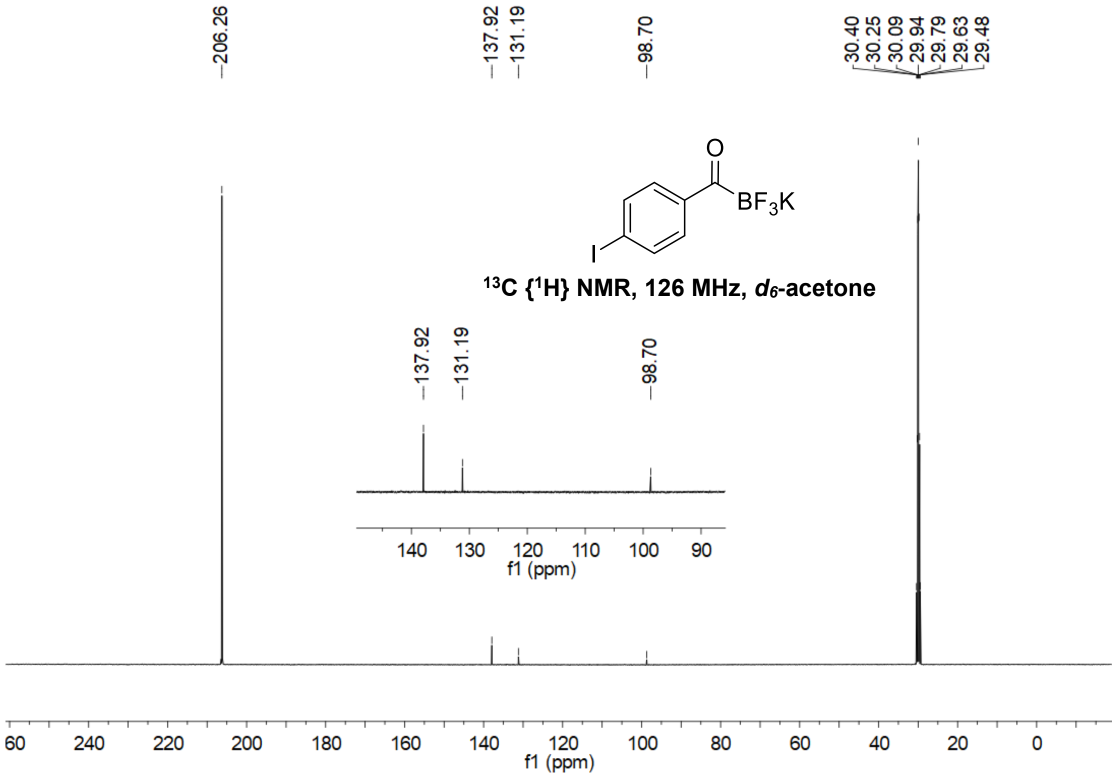
206.26
137.92
131.19
98.70
30.40
30.25
30.09
29.94
29.79
29.63
29.48
O
BF3K
I
13C {1H} NMR, 126 MHz, d6-acetone
140 130 120 110 100 90
f1 (ppm)
60 240 220 200 180 160 140 120 100 80 60 40 20 0
f1 (ppm)

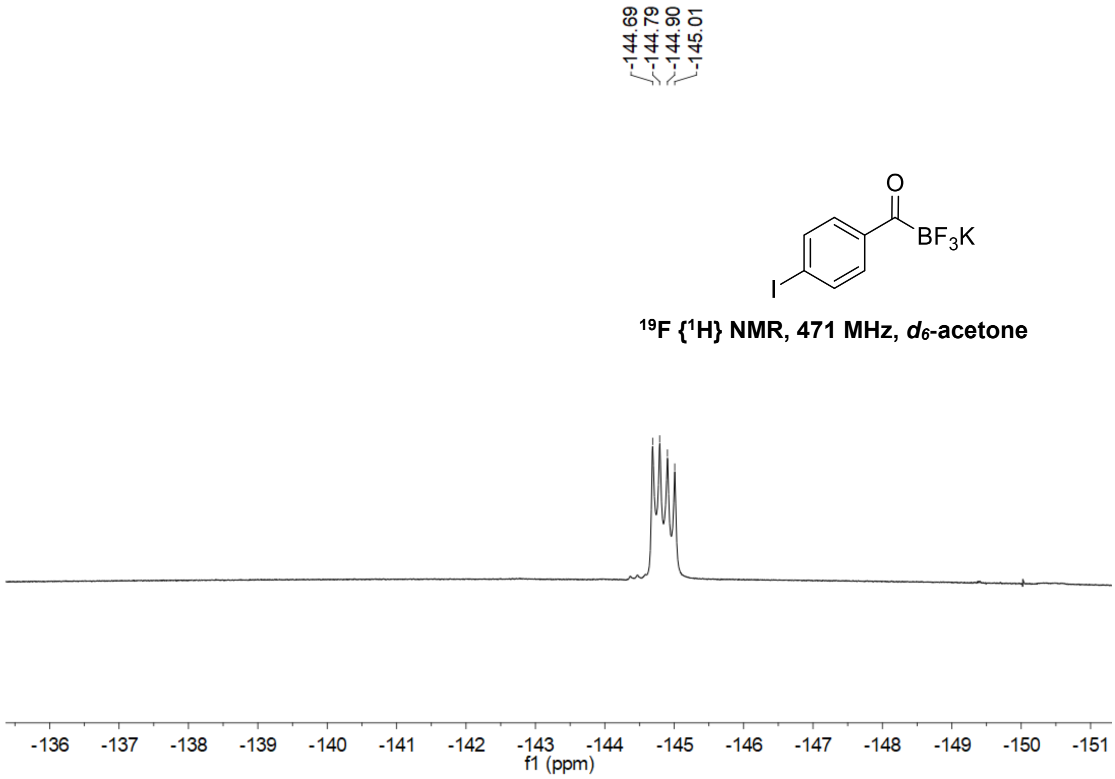
-144.69
-144.79
-144.90
-145.01
O
BF3K
I
19F {1H} NMR, 471 MHz, d6-acetone
-136 -137 -138 -139 -140 -141 -142 -143 -144 -145 -146 -147 -148 -149 -150 -151
f1 (ppm)

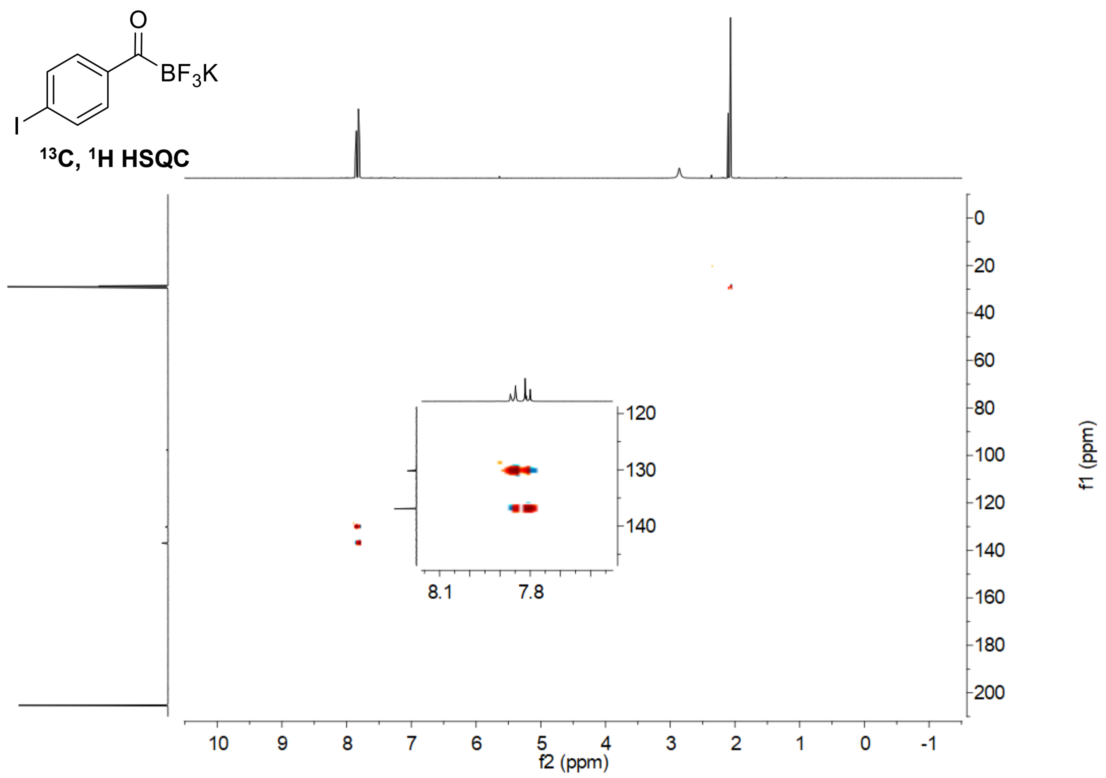
O
BF3K
I
13C, 1H HSQC
f1 (ppm)
f2 (ppm)

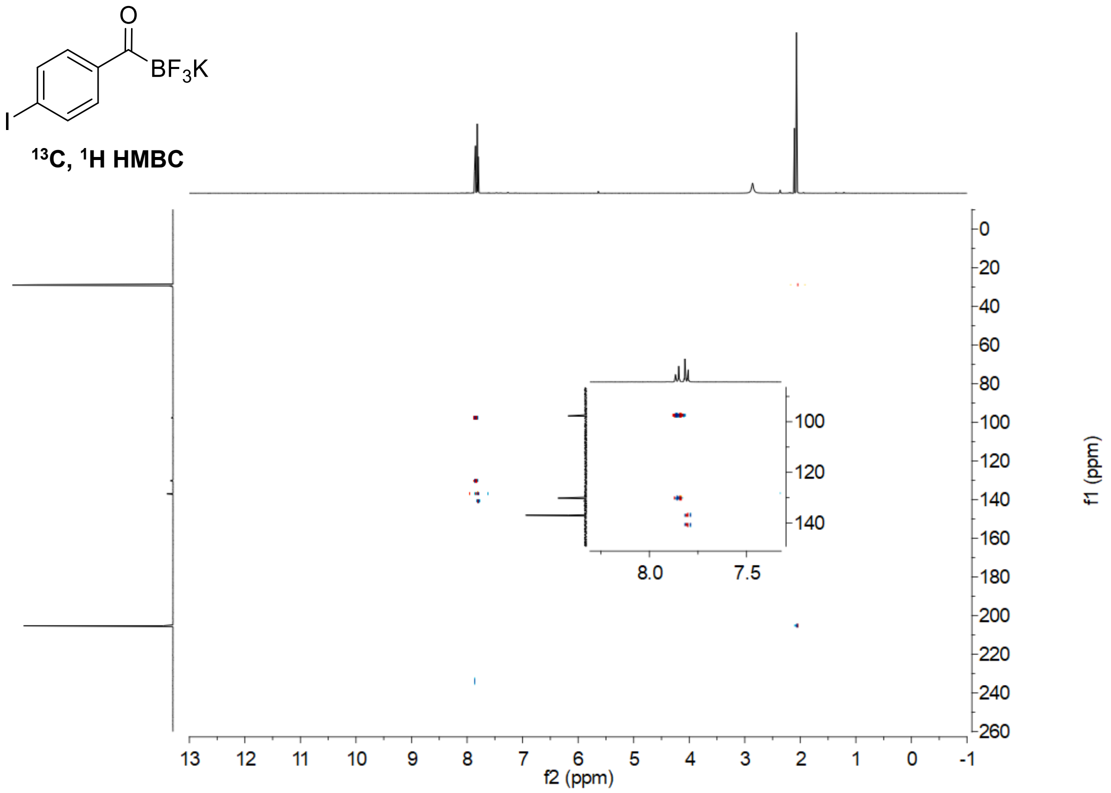
O
BF3K
I
13C, 1H HMBC
f1 (ppm)
f2 (ppm)

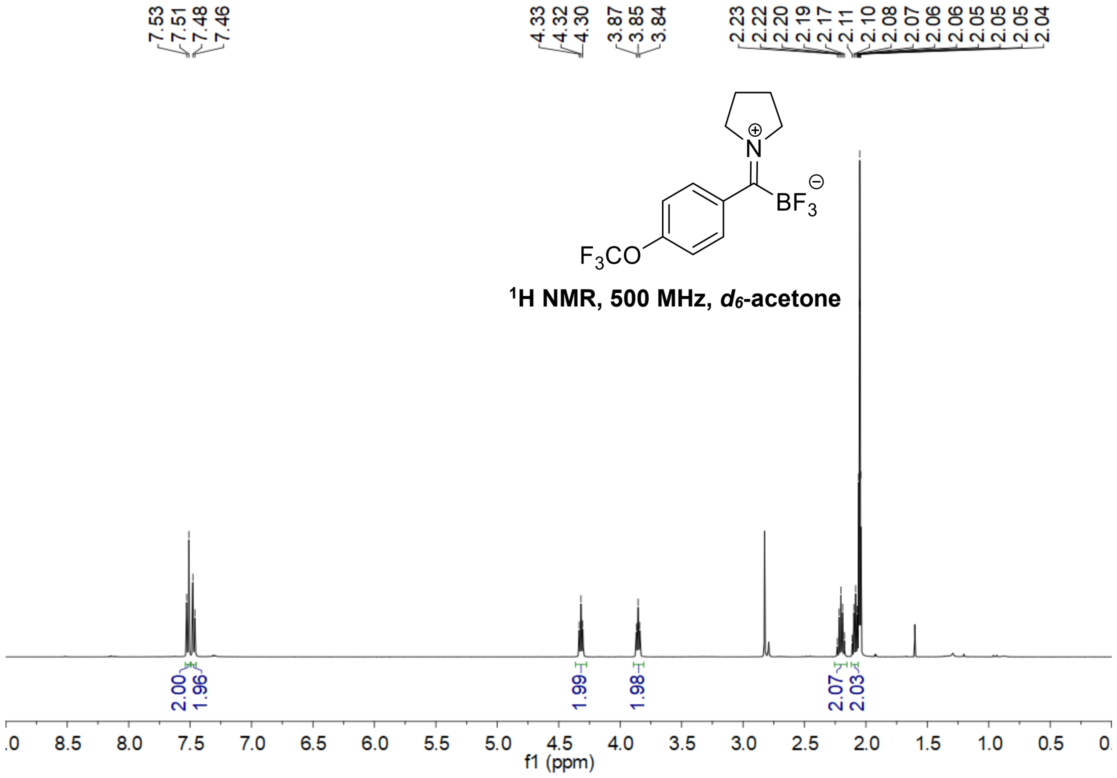
7.53
7.51
7.48
7.46
4.33
4.32
4.30
3.87
3.85
3.84
2.23
2.22
2.20
2.19
2.17
2.11
2.10
2.08
2.07
2.06
2.06
2.05
2.05
2.05
2.04
BF3
F3CO
1H NMR, 500 MHz, d6-acetone
2.00
1.96
1.99
1.98
2.07
2.03
f1 (ppm)

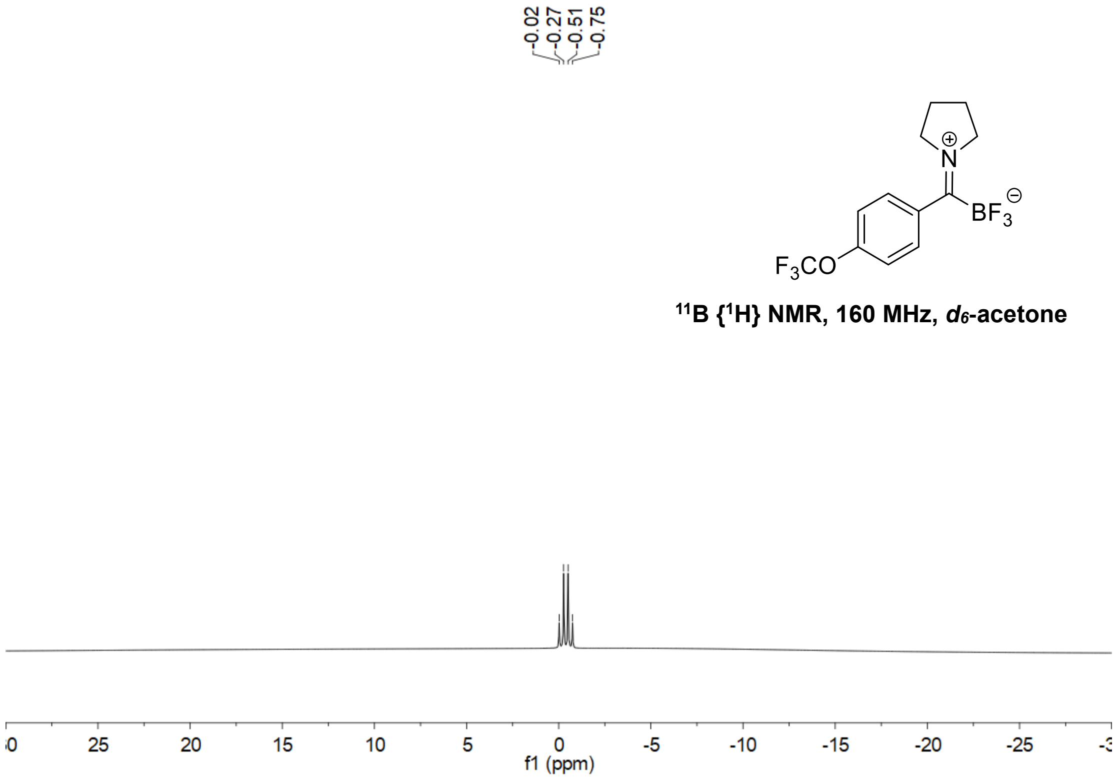
-0.02
-0.27
-0.51
-0.75
BF3
F3CO
11B {1H} NMR, 160 MHz, d6-acetone
f1 (ppm)

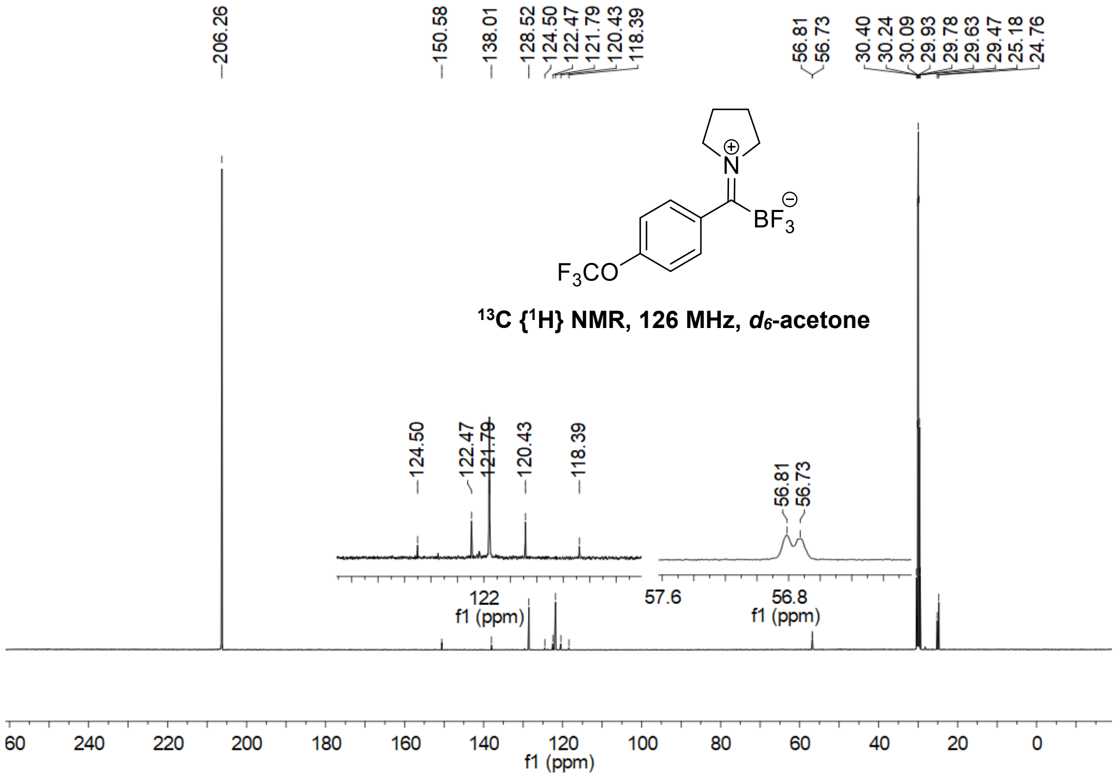

206.26
150.58
138.01
128.52
124.50
122.47
121.79
120.43
118.39
56.81
56.73
30.40
30.24
30.09
29.93
29.78
29.63
29.47
25.18
24.76
F3CO
BF3
N
13C {1H} NMR, 126 MHz, d6-acetone
122
f1 (ppm)
57.6
56.8
f1 (ppm)
60
240
220
200
180
160
140
120
100
80
60
40
20
0
f1 (ppm)

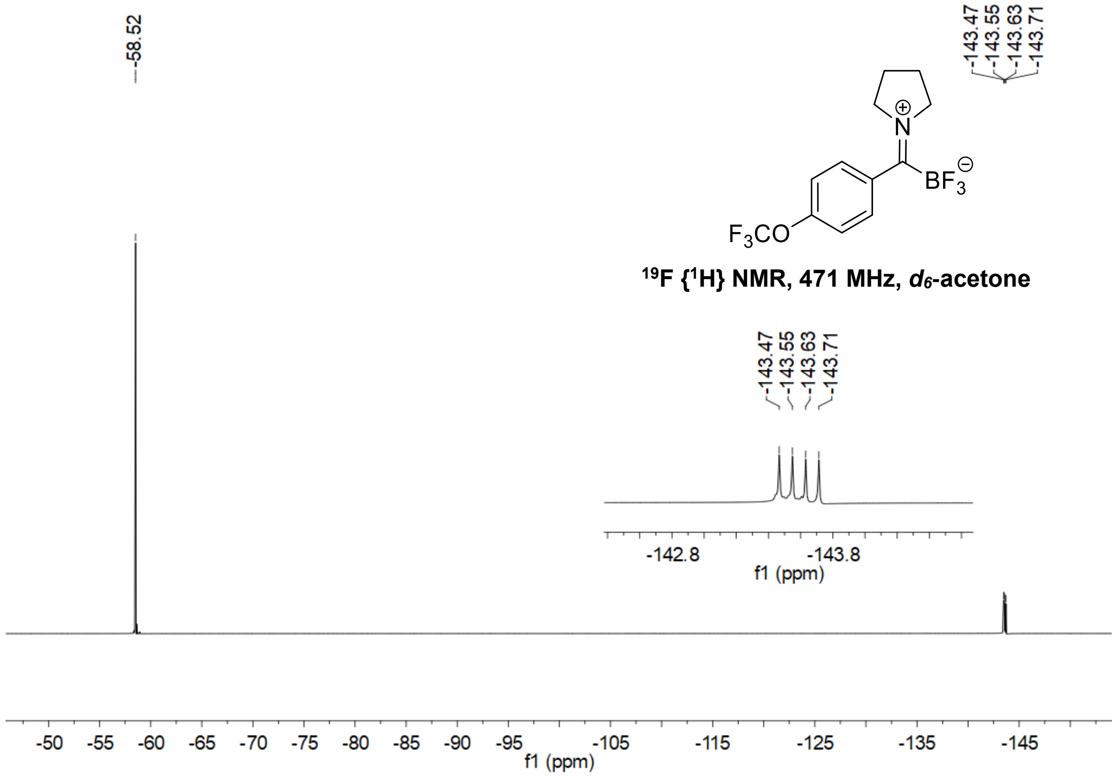

-58.52
-143.47
-143.55
-143.63
-143.71
F3CO
BF3
N
19F {1H} NMR, 471 MHz, d6-acetone
-142.8
-143.8
f1 (ppm)
-50
-55
-60
-65
-70
-75
-80
-85
-90
-95
-105
-115
-125
-135
-145
f1 (ppm)

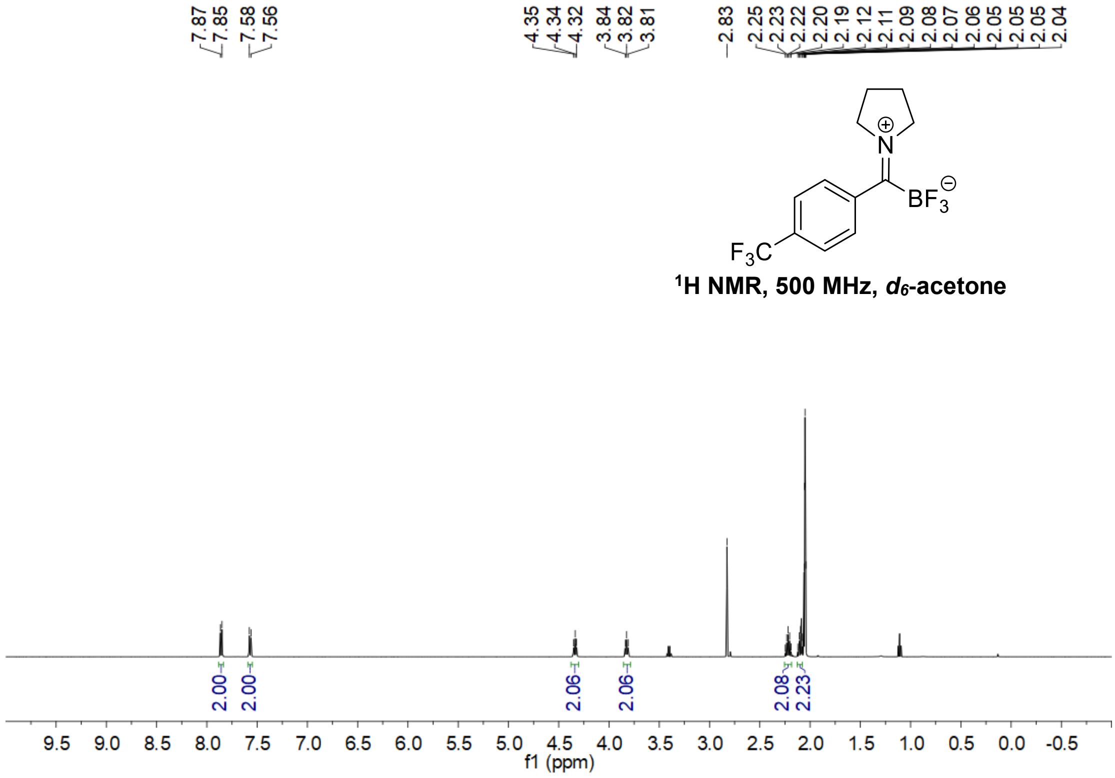
7.87
7.85
7.58
7.56
4.35
4.34
4.32
3.84
3.82
3.81
2.83
2.25
2.23
2.22
2.20
2.19
2.12
2.11
2.09
2.08
2.07
2.06
2.05
2.05
2.04
F3C
BF3
N
1H NMR, 500 MHz, d6-acetone
2.00
2.00
2.06
2.06
2.08
2.23
f1 (ppm)

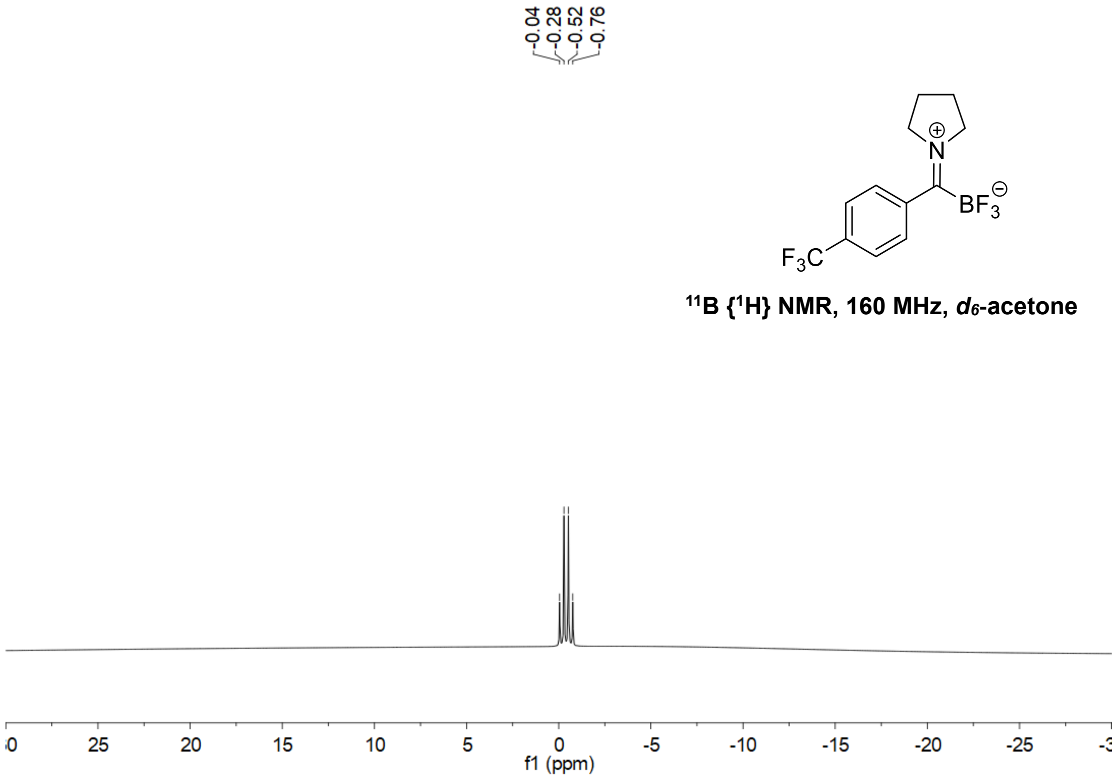
-0.04
-0.28
-0.52
-0.76
F3C
BF3
N
11B {1H} NMR, 160 MHz, d6-acetone
f1 (ppm)

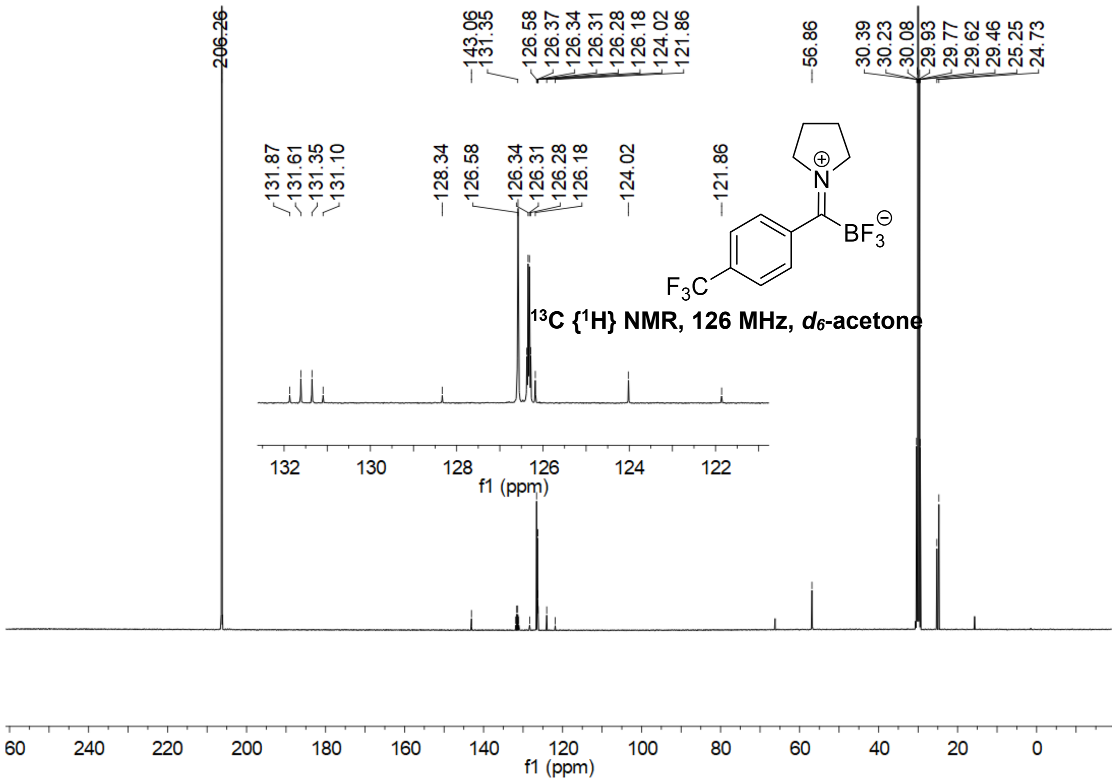
13C {1H} NMR, 126 MHz, d6-acetone
f1 (ppm)

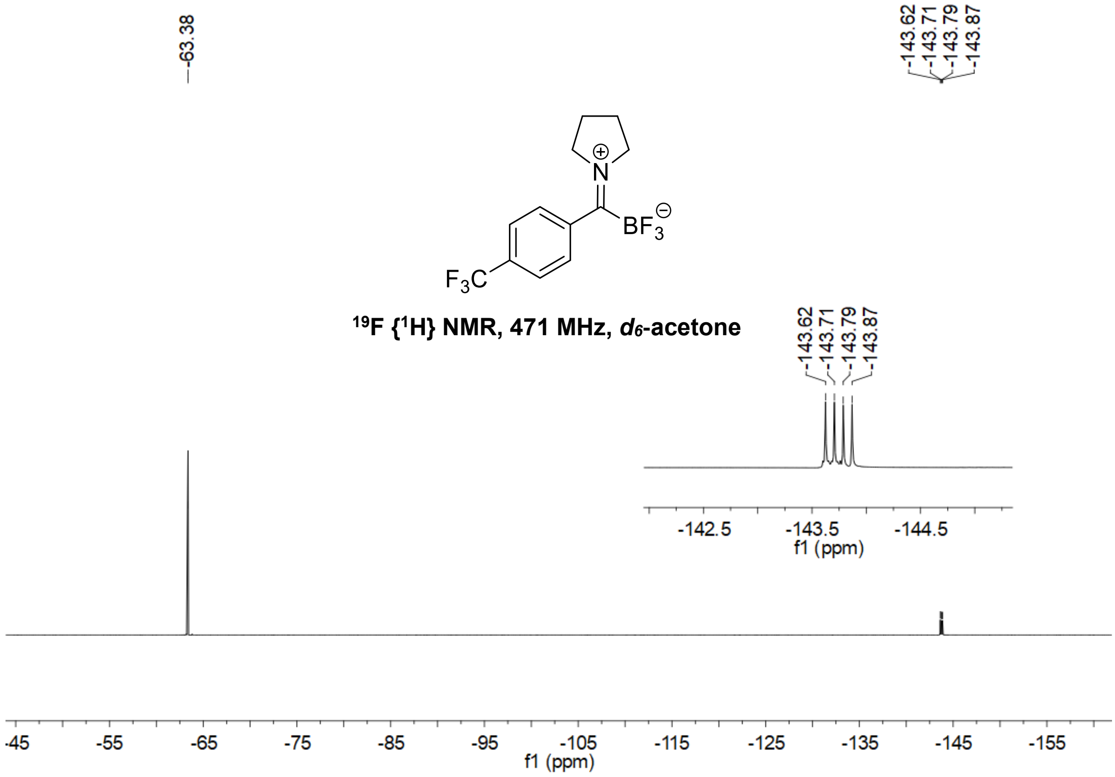
19F {1H} NMR, 471 MHz, d6-acetone
f1 (ppm)

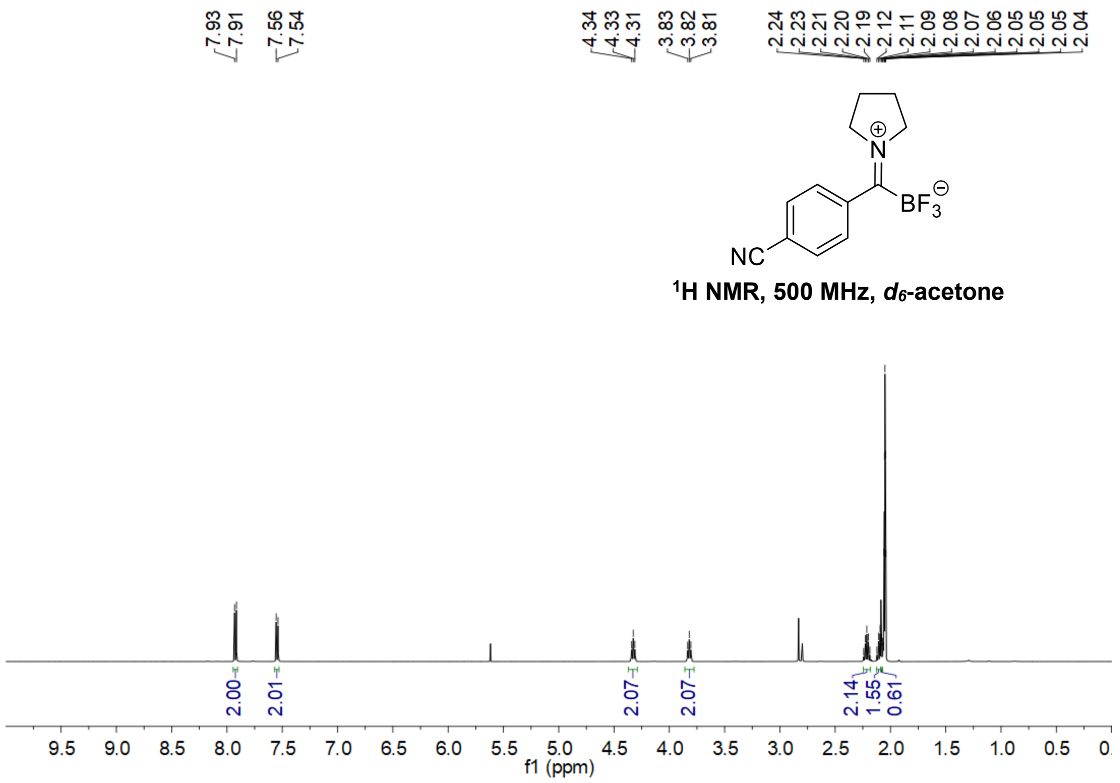

7.93
7.91
7.56
7.54
4.34
4.33
4.31
3.83
3.82
3.81
2.24
2.23
2.21
2.20
2.19
2.12
2.11
2.09
2.08
2.07
2.06
2.05
2.05
2.05
2.04
N
BF3
NC
1H NMR, 500 MHz, d6-acetone
2.00
2.01
2.07
2.07
2.14
1.55
0.61
f1 (ppm)

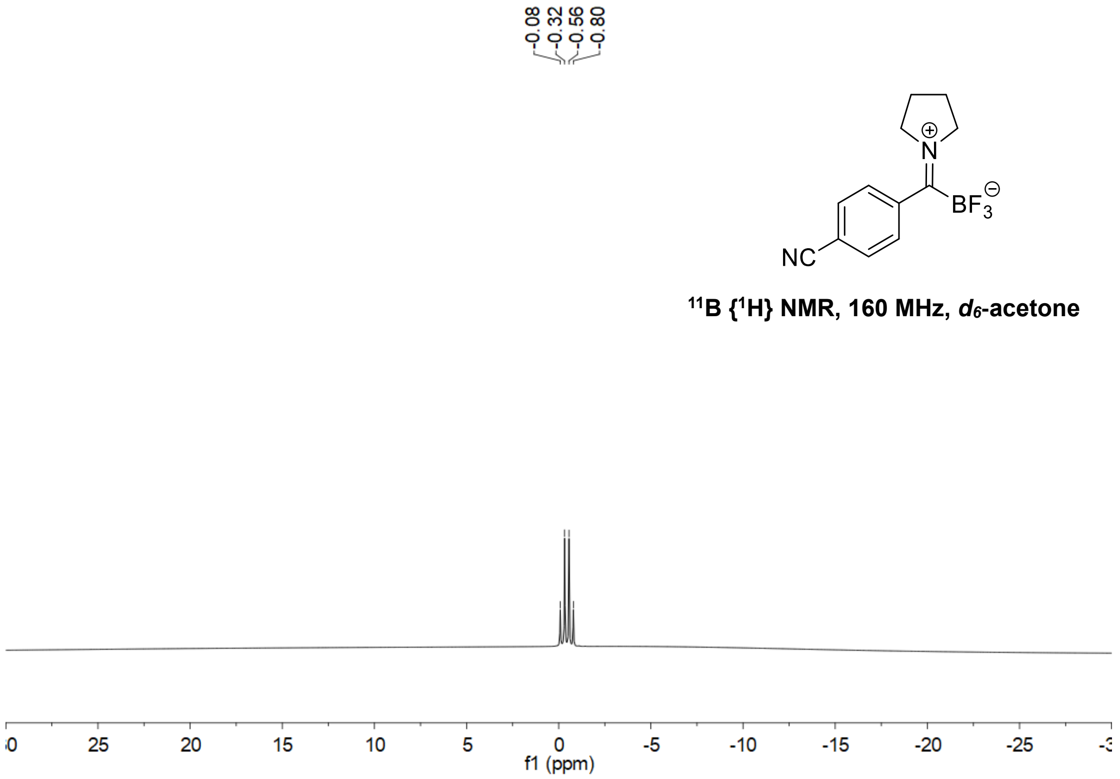

-0.08
-0.32
-0.56
-0.80
N
BF3
NC
11B {1H} NMR, 160 MHz, d6-acetone
f1 (ppm)

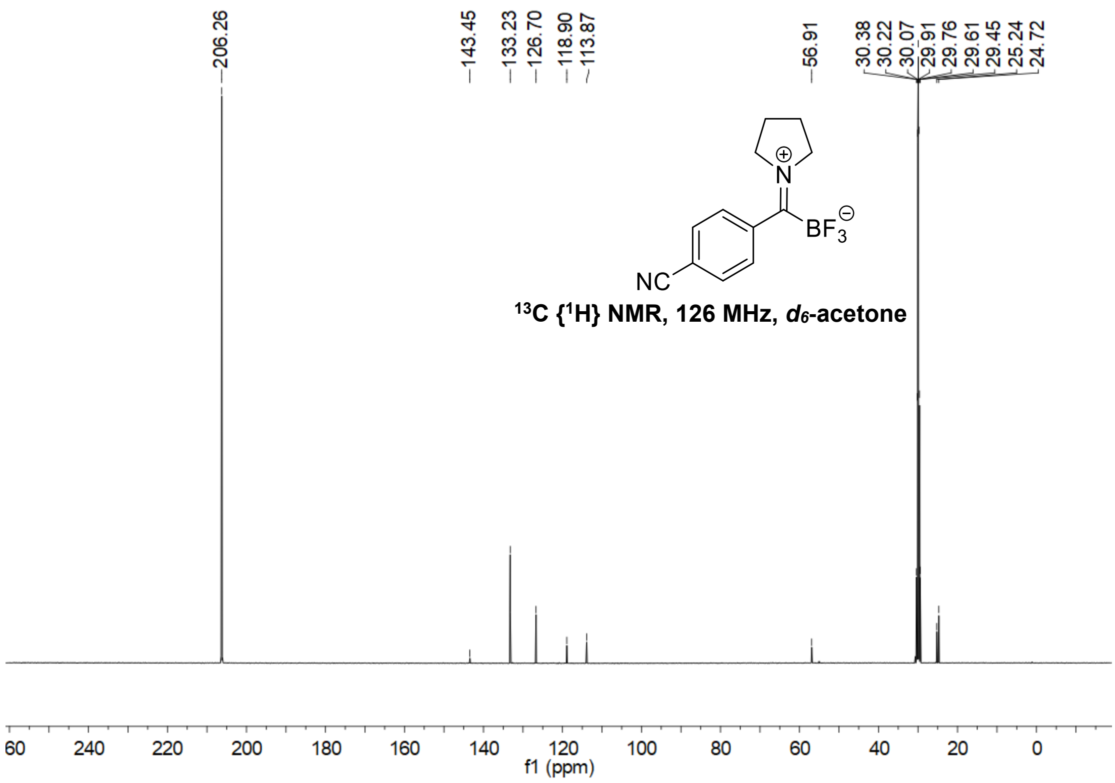
206.26
143.45
133.23
126.70
118.90
113.87
56.91
30.38
30.22
30.07
29.91
29.76
29.61
29.45
25.24
24.72
NC
BF3
13C {1H} NMR, 126 MHz, d6-acetone
f1 (ppm)

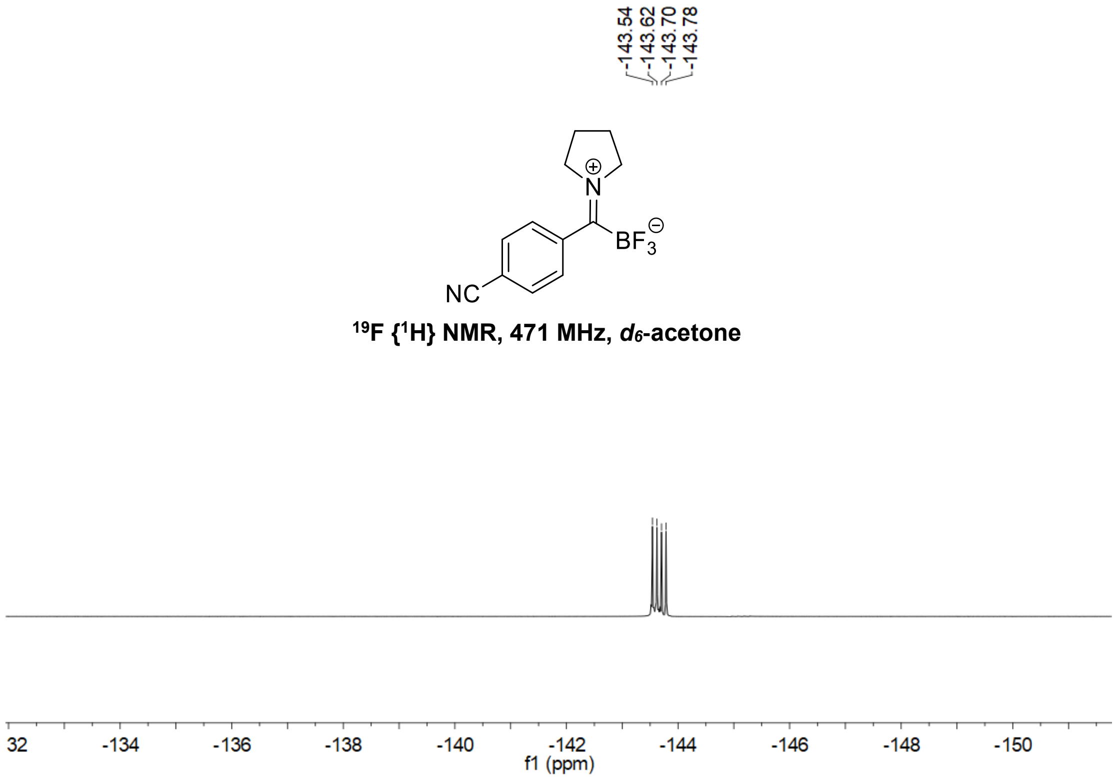
-143.54
-143.62
-143.70
-143.78
NC
BF3
19F {1H} NMR, 471 MHz, d6-acetone
f1 (ppm)

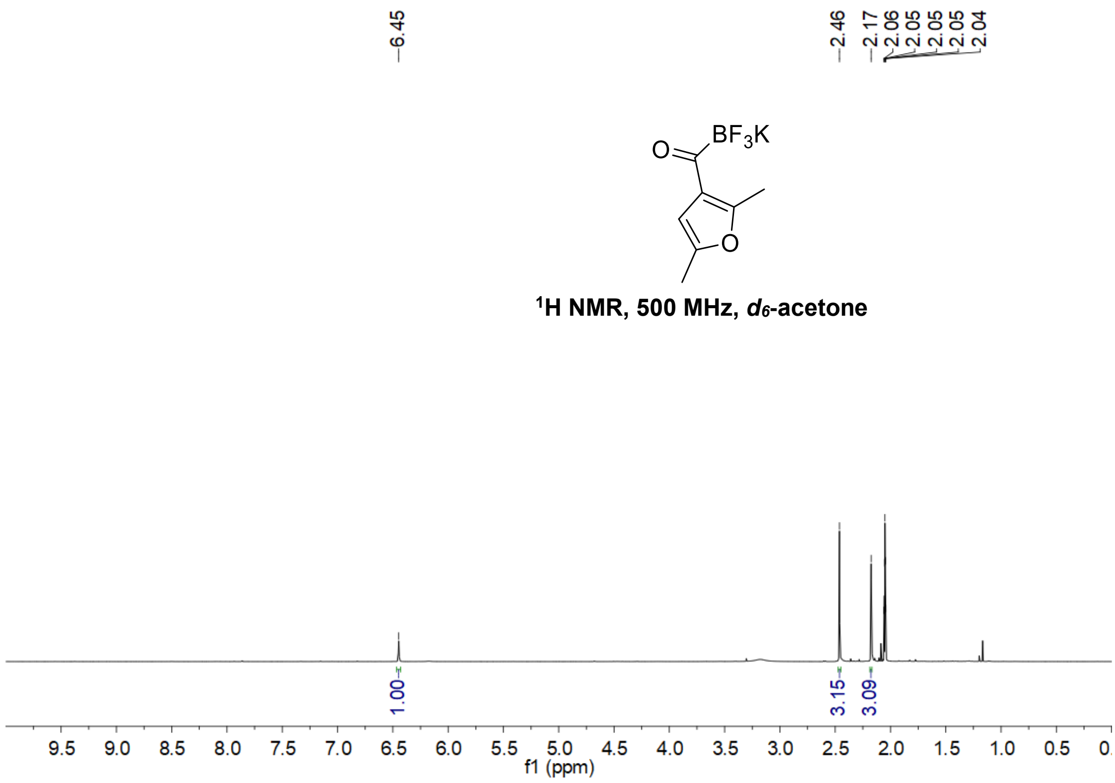
6.45
2.46
2.17
2.06
2.05
2.05
2.05
2.04
BF3K
O
O
1H NMR, 500 MHz, d6-acetone
1.00
3.15
3.09
9.5
9.0
8.5
8.0
7.5
7.0
6.5
6.0
5.5
5.0
4.5
4.0
3.5
3.0
2.5
2.0
1.5
1.0
0.5
f1 (ppm)

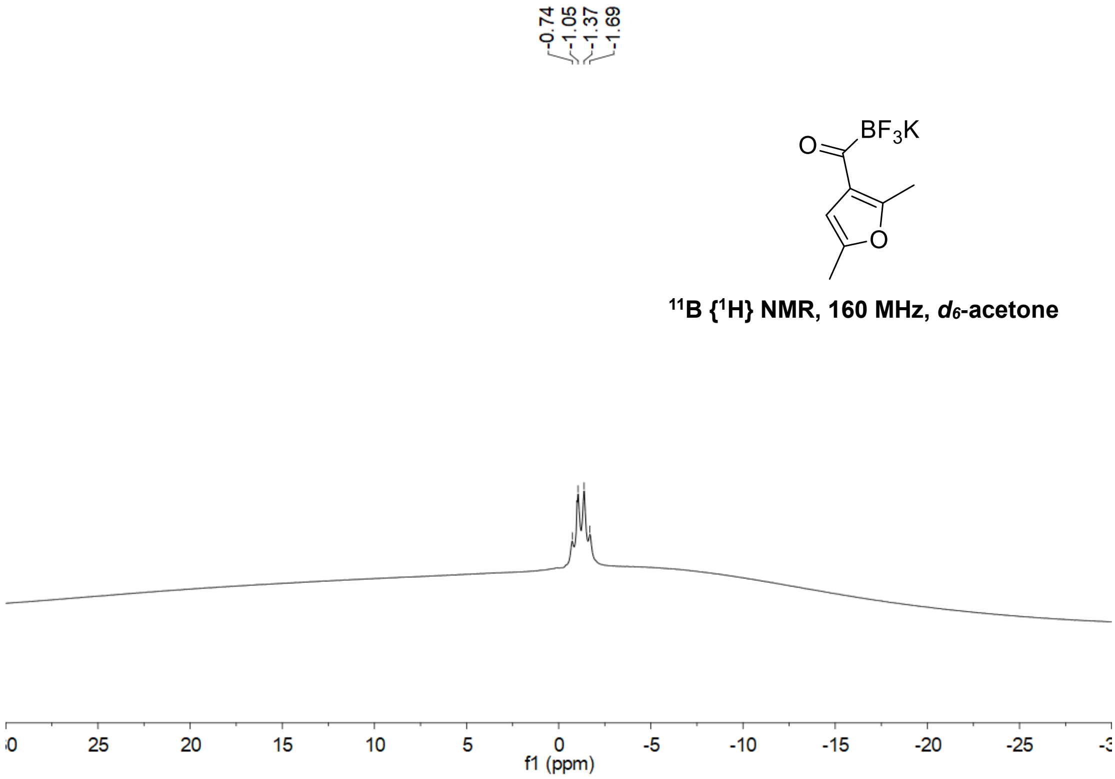
-0.74
-1.05
-1.37
-1.69
BF3K
O
O
11B {1H} NMR, 160 MHz, d6-acetone
25
20
15
10
5
0
-5
-10
-15
-20
-25
f1 (ppm)

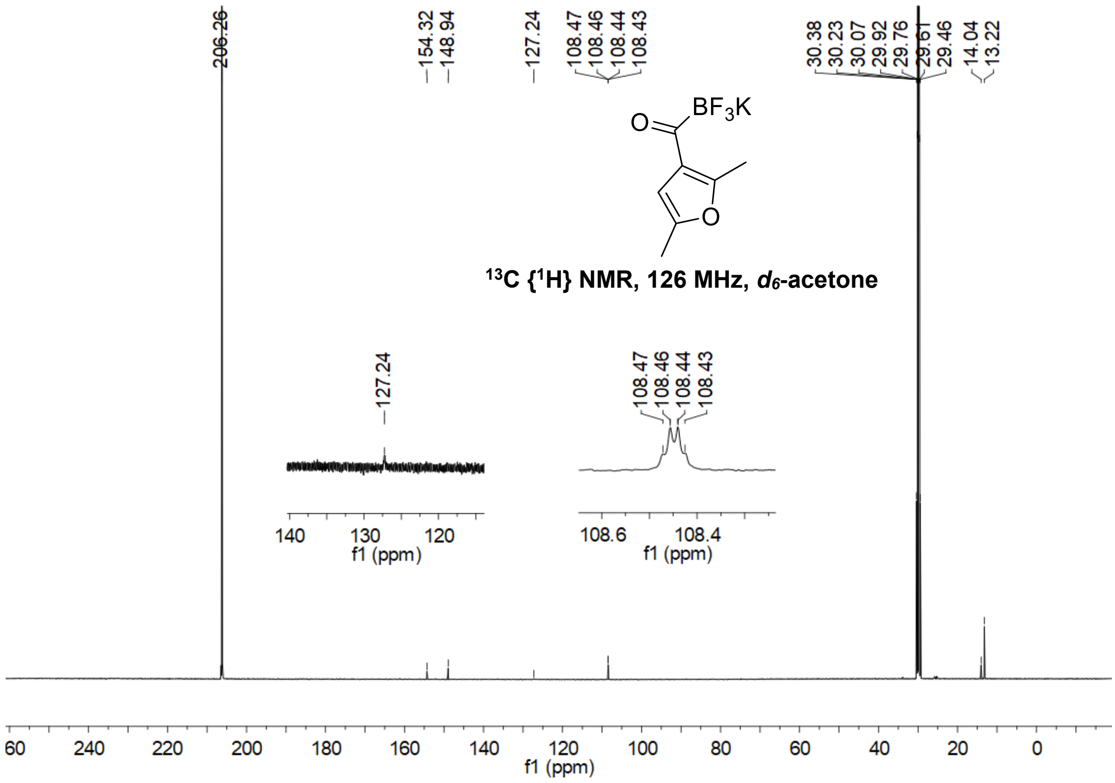
206.26
154.32
148.94
127.24
108.47
108.46
108.44
108.43
30.38
30.23
30.07
29.92
29.76
29.61
29.46
14.04
13.22
BF3K
O
O
13C {1H} NMR, 126 MHz, d6-acetone
140
130
120
f1 (ppm)
108.6
108.4
f1 (ppm)
60
240
220
200
180
160
140
120
100
80
60
40
20
0
f1 (ppm)

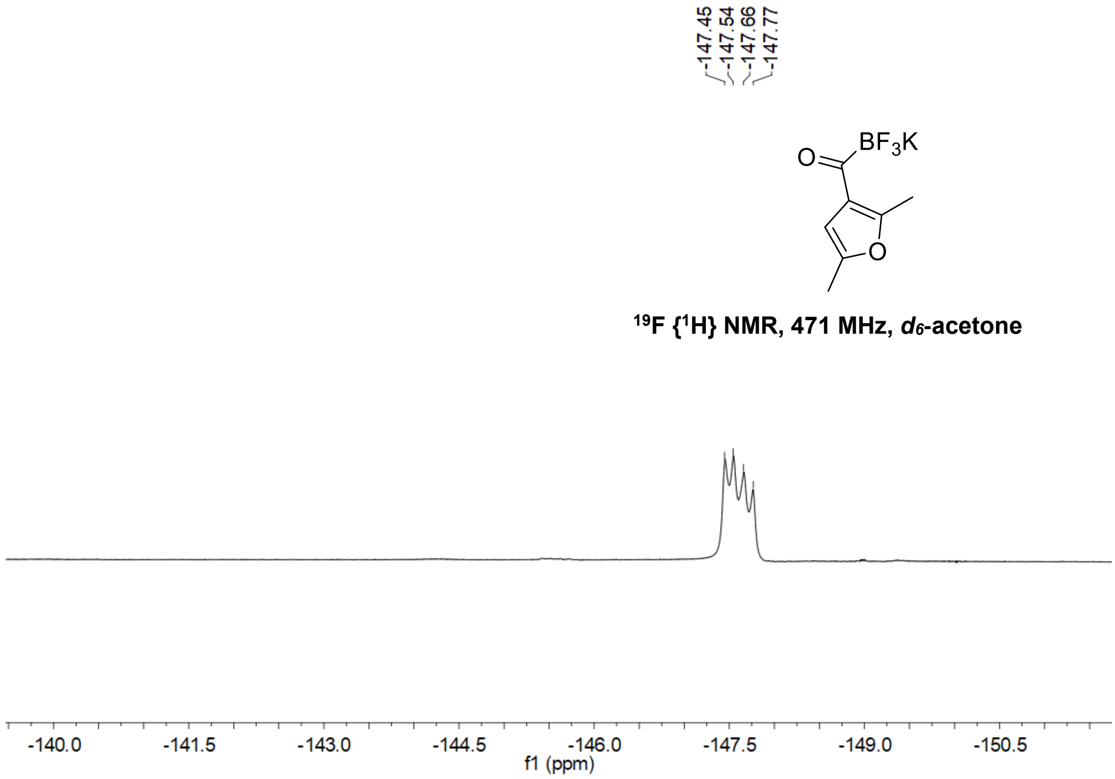
-147.45
-147.54
-147.66
-147.77
BF3K
O
O
19F {1H} NMR, 471 MHz, d6-acetone
-140.0
-141.5
-143.0
-144.5
-146.0
-147.5
-149.0
-150.5
f1 (ppm)

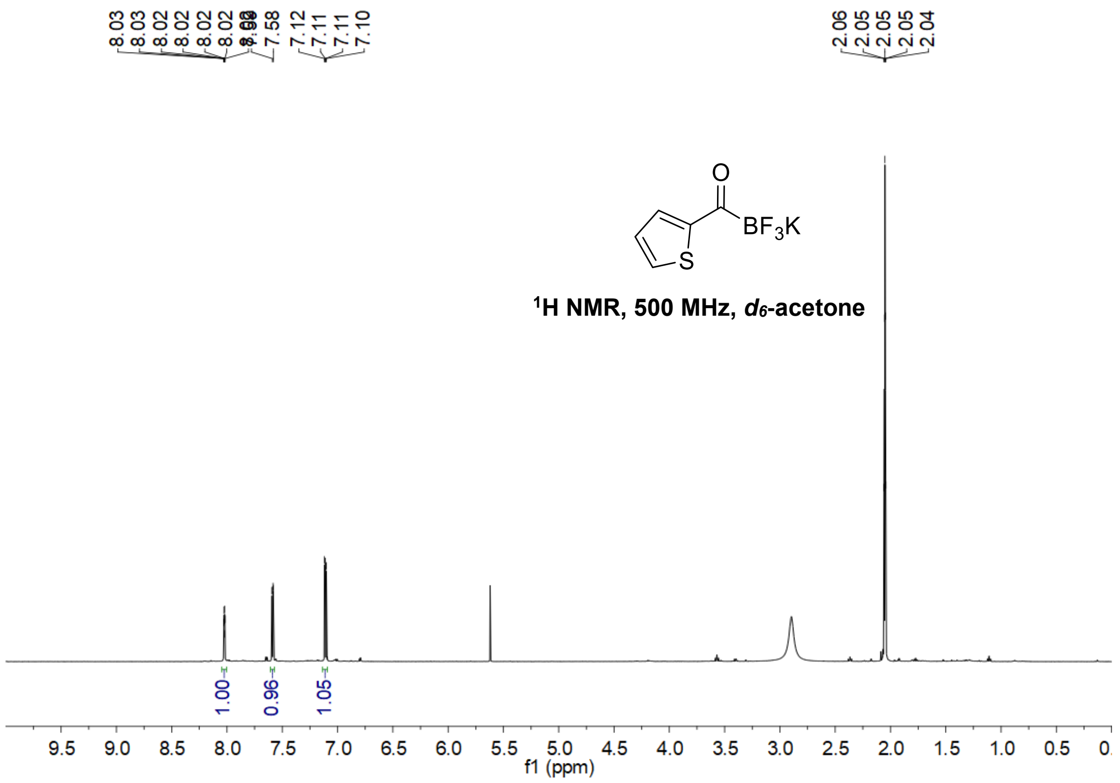
8.03
8.03
8.02
8.02
8.02
8.02
7.58
7.12
7.11
7.11
7.10
2.06
2.05
2.05
2.05
2.04
O
BF3K
S
1H NMR, 500 MHz, d6-acetone
1.00
0.96
1.05
9.5
9.0
8.5
8.0
7.5
7.0
6.5
6.0
5.5
5.0
4.5
4.0
3.5
3.0
2.5
2.0
1.5
1.0
0.5
f1 (ppm)

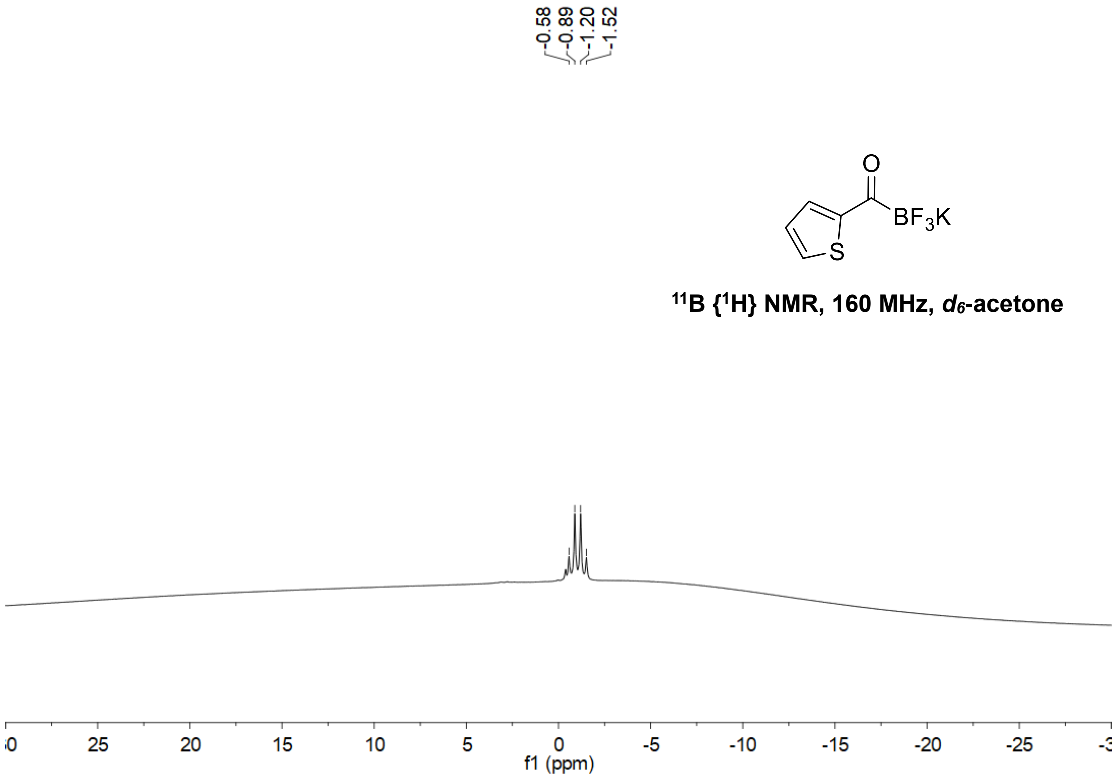
-0.58
-0.89
-1.20
-1.52
O
BF3K
S
11B {1H} NMR, 160 MHz, d6-acetone
25
20
15
10
5
0
-5
-10
-15
-20
-25
f1 (ppm)

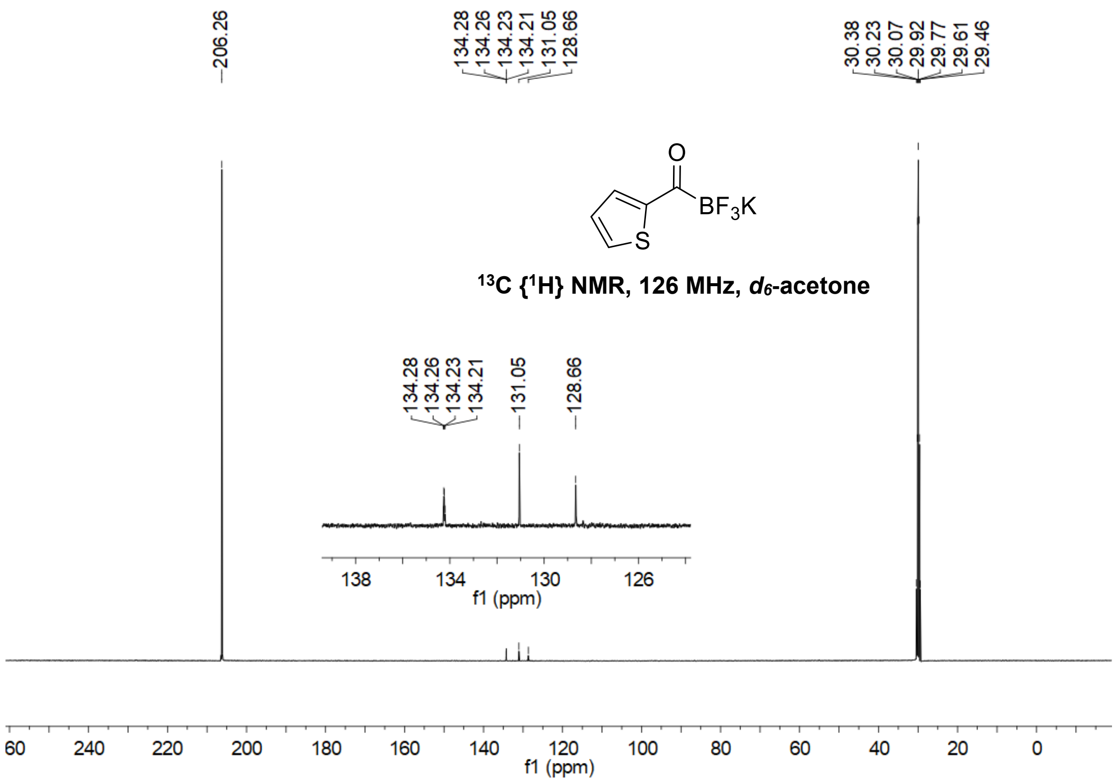
206.26
134.28
134.26
134.23
134.21
131.05
128.66
30.38
30.23
30.07
29.92
29.77
29.61
29.46
O
BF3K
S
13C {1H} NMR, 126 MHz, d6-acetone
138
134
130
126
f1 (ppm)
60
240
220
200
180
160
140
120
100
80
60
40
20
0
f1 (ppm)

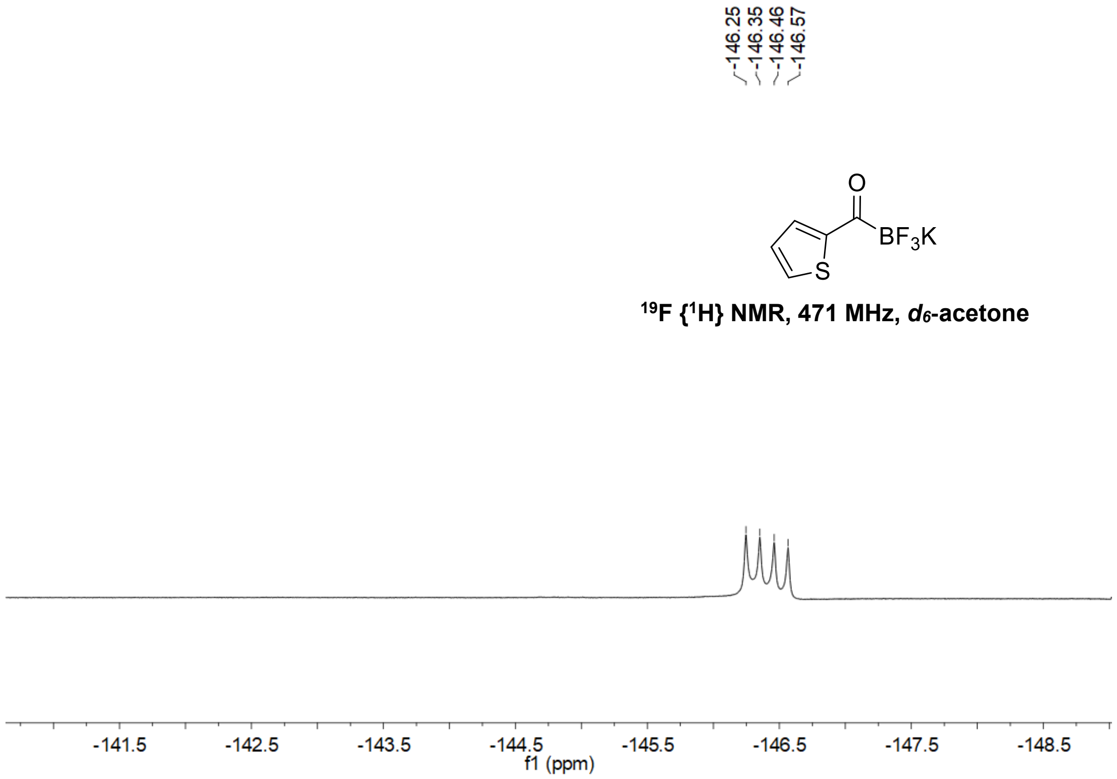
-146.25
-146.35
-146.46
-146.57
O
BF3K
S
19F {1H} NMR, 471 MHz, d6-acetone
-141.5
-142.5
-143.5
-144.5
-145.5
-146.5
-147.5
-148.5
f1 (ppm)

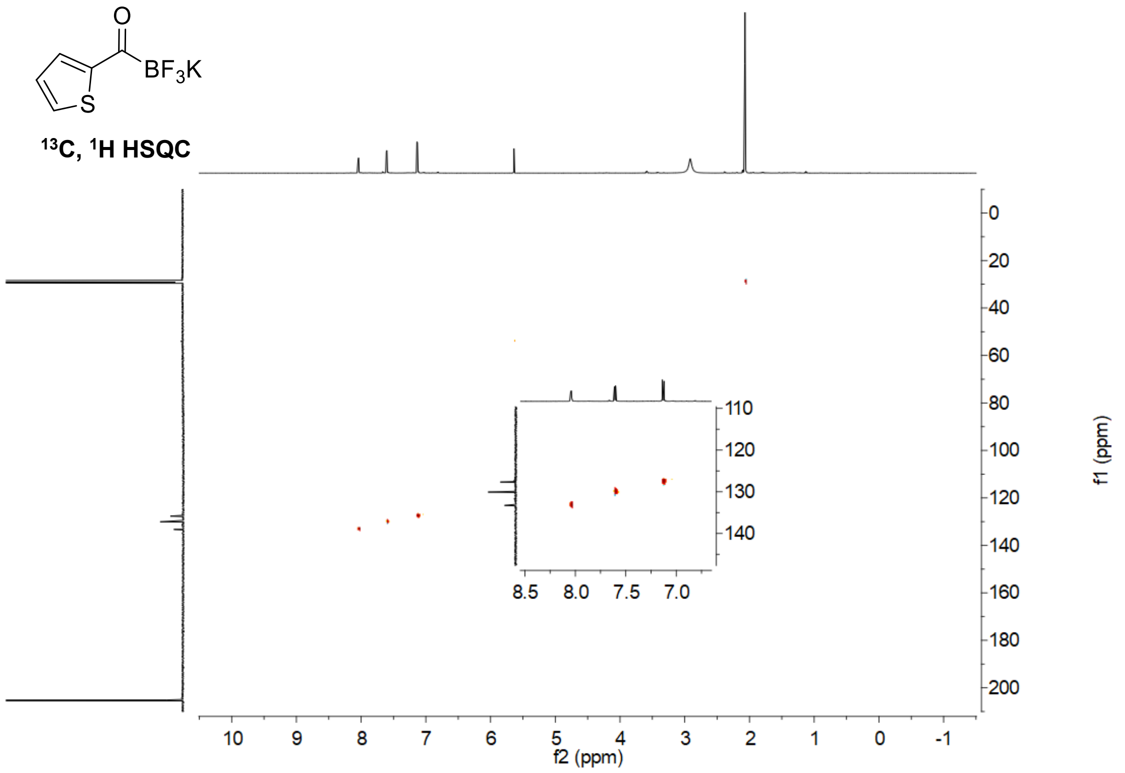
O
BF3K
S
13C, 1H HSQC
f2 (ppm)
f1 (ppm)

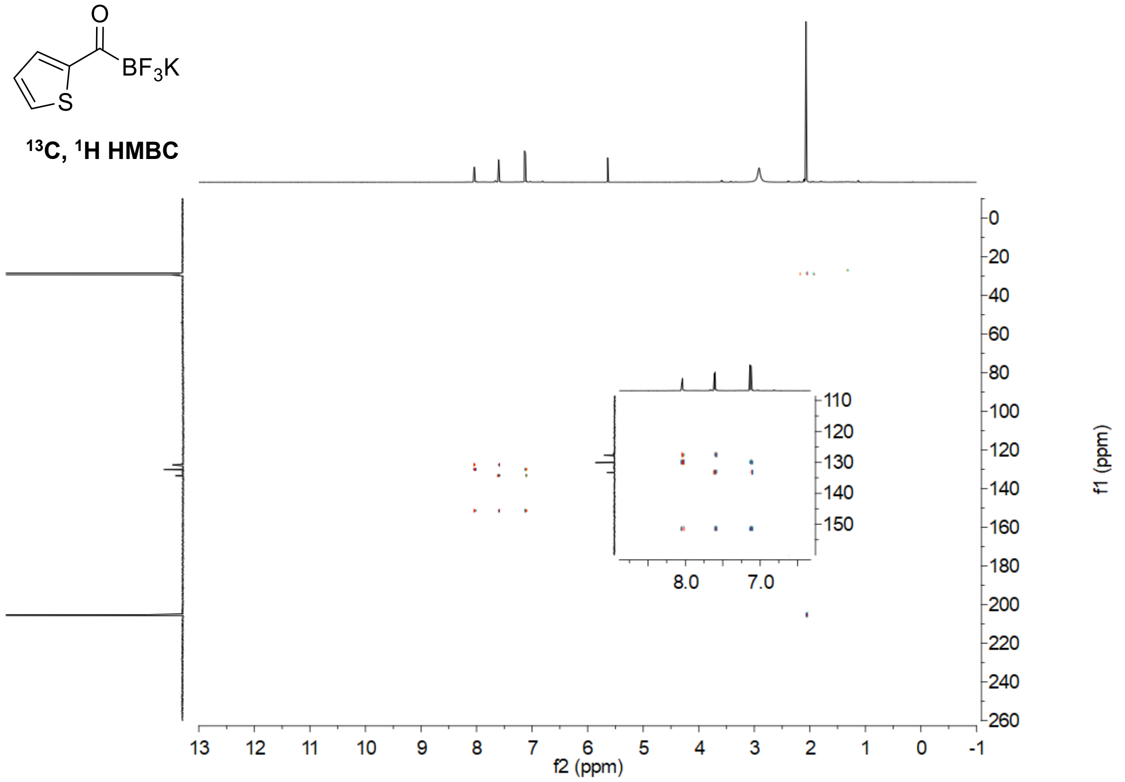
O
BF3K
S
13C, 1H HMBC
f2 (ppm)
f1 (ppm)

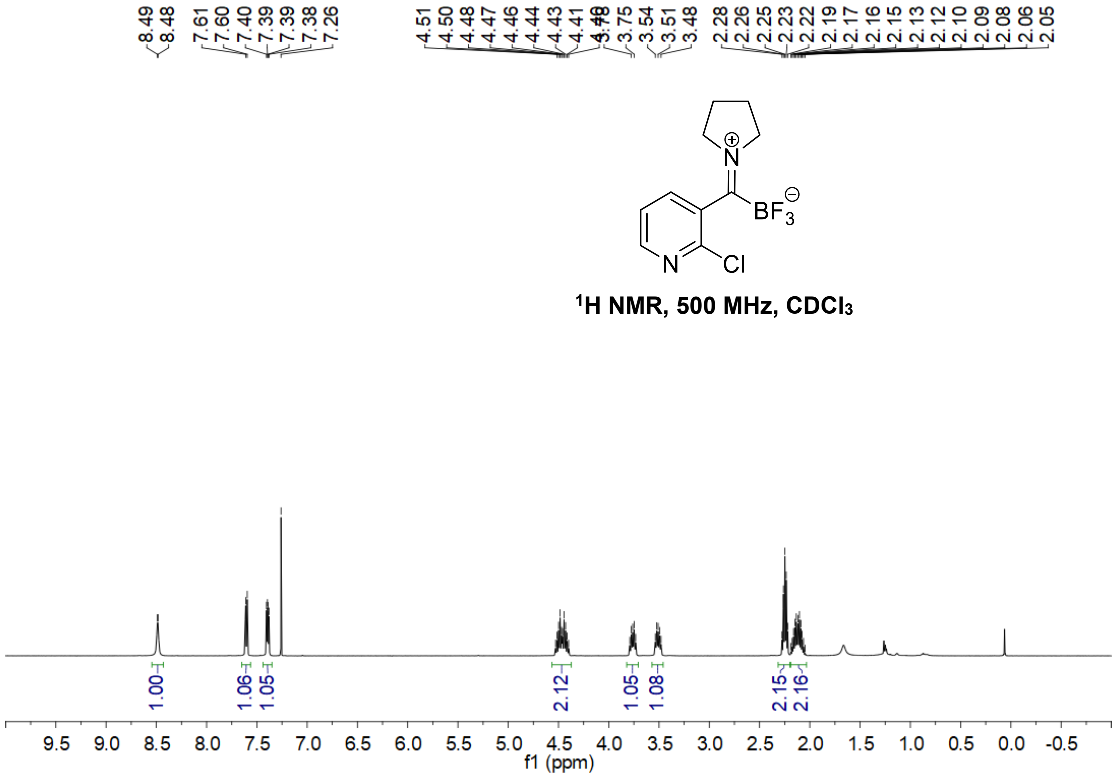
BF3
N
Cl
1H NMR, 500 MHz, CDCl3
1.00
1.06
1.05
2.12
1.05
1.08
2.15
2.16
f1 (ppm)

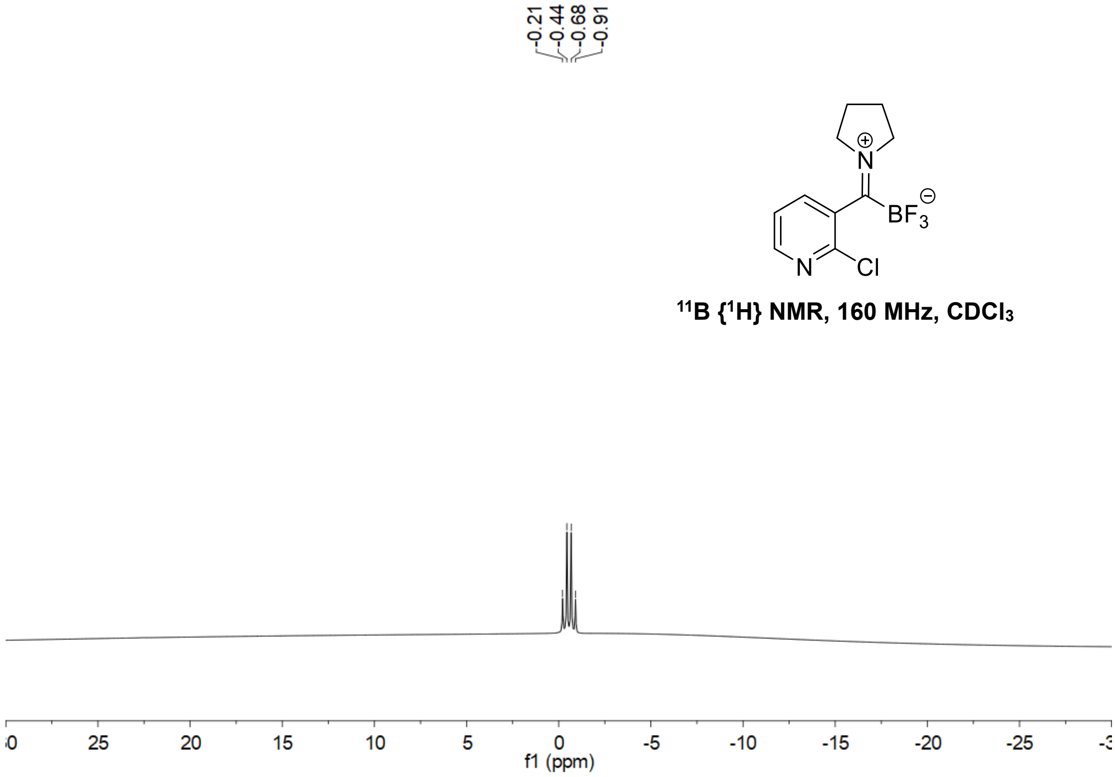
-0.21
-0.44
-0.68
-0.91
BF3
N
Cl
11B {1H} NMR, 160 MHz, CDCl3
f1 (ppm)

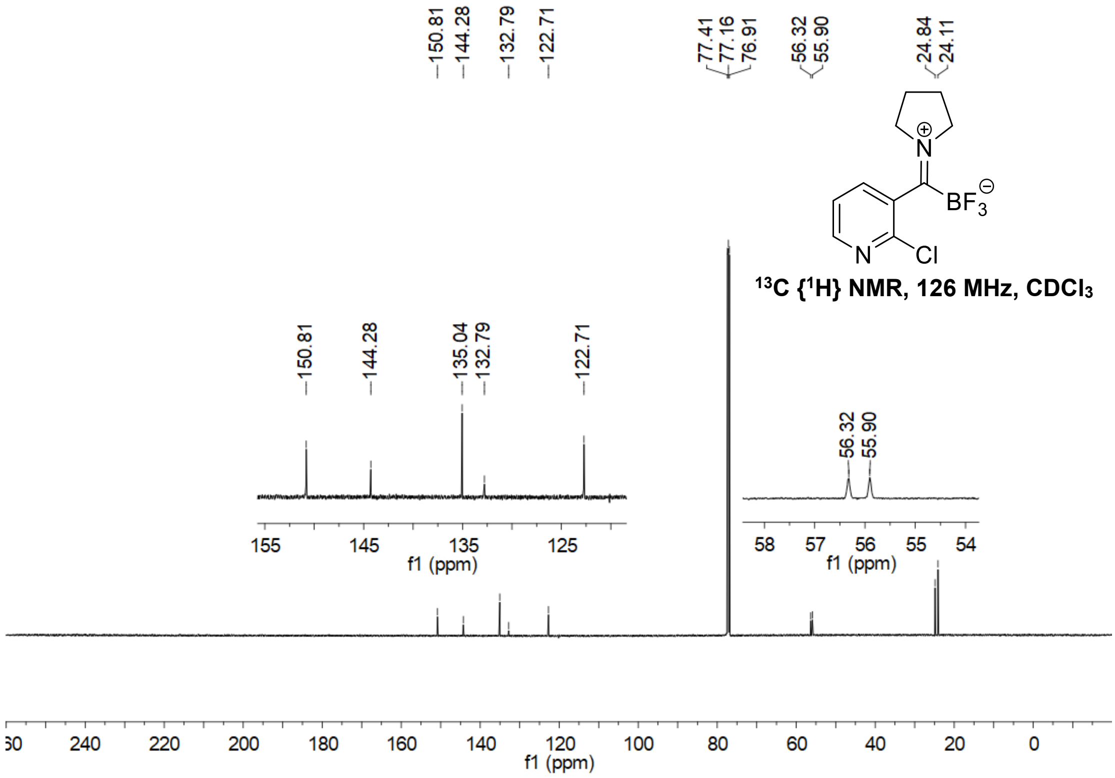

^{13}C {^1H} NMR, 126 MHz, $CDCl_3$

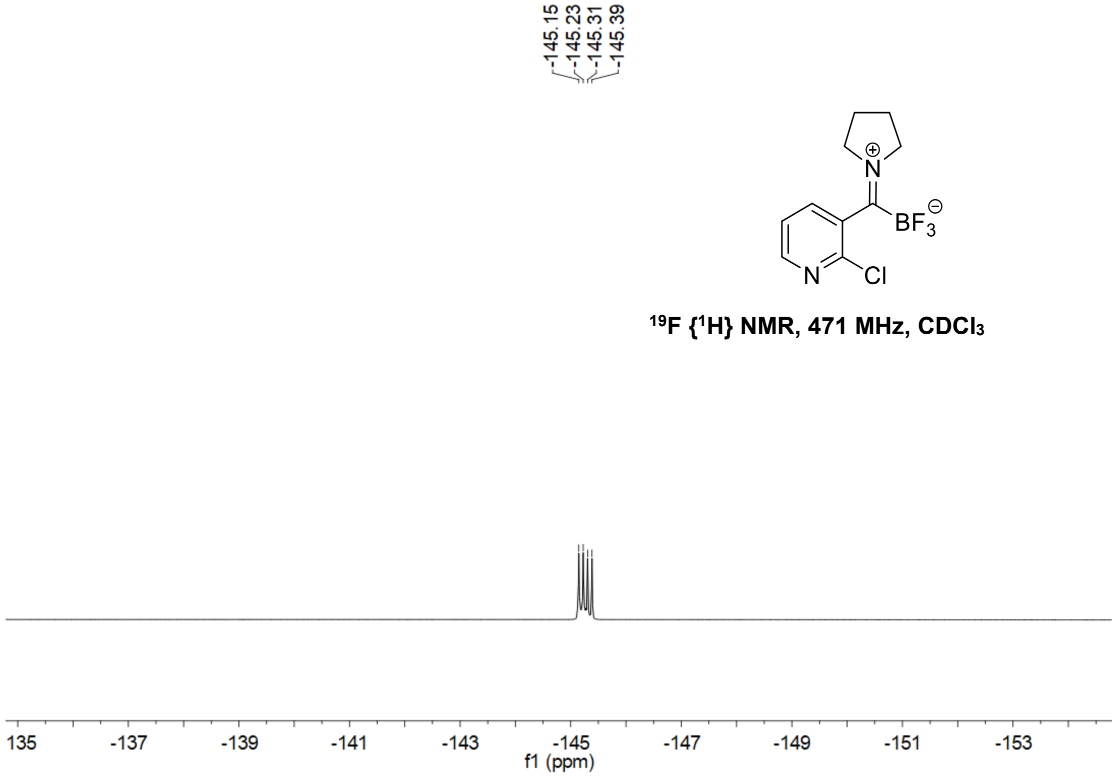

^{19}F {^1H} NMR, 471 MHz, $CDCl_3$

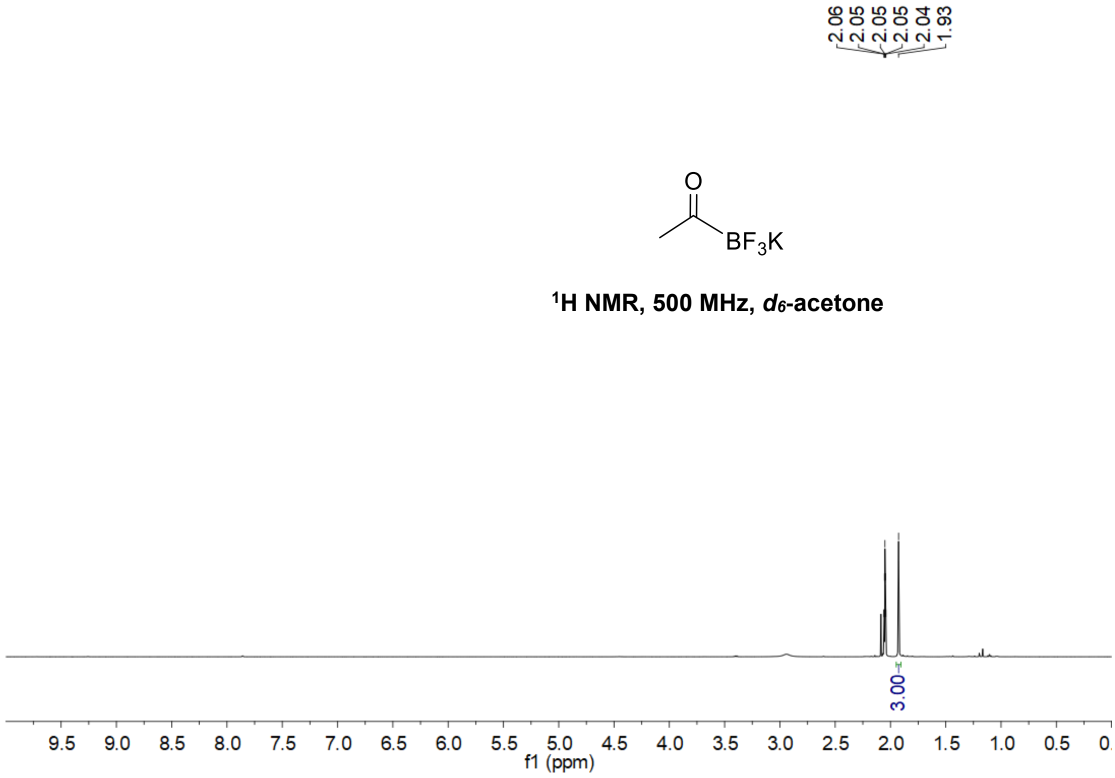
2.06
2.05
2.05
2.05
2.04
1.93
BF3K
O
1H NMR, 500 MHz, d6-acetone
3.00
9.5 9.0 8.5 8.0 7.5 7.0 6.5 6.0 5.5 5.0 4.5 4.0 3.5 3.0 2.5 2.0 1.5 1.0 0.5
f1 (ppm)

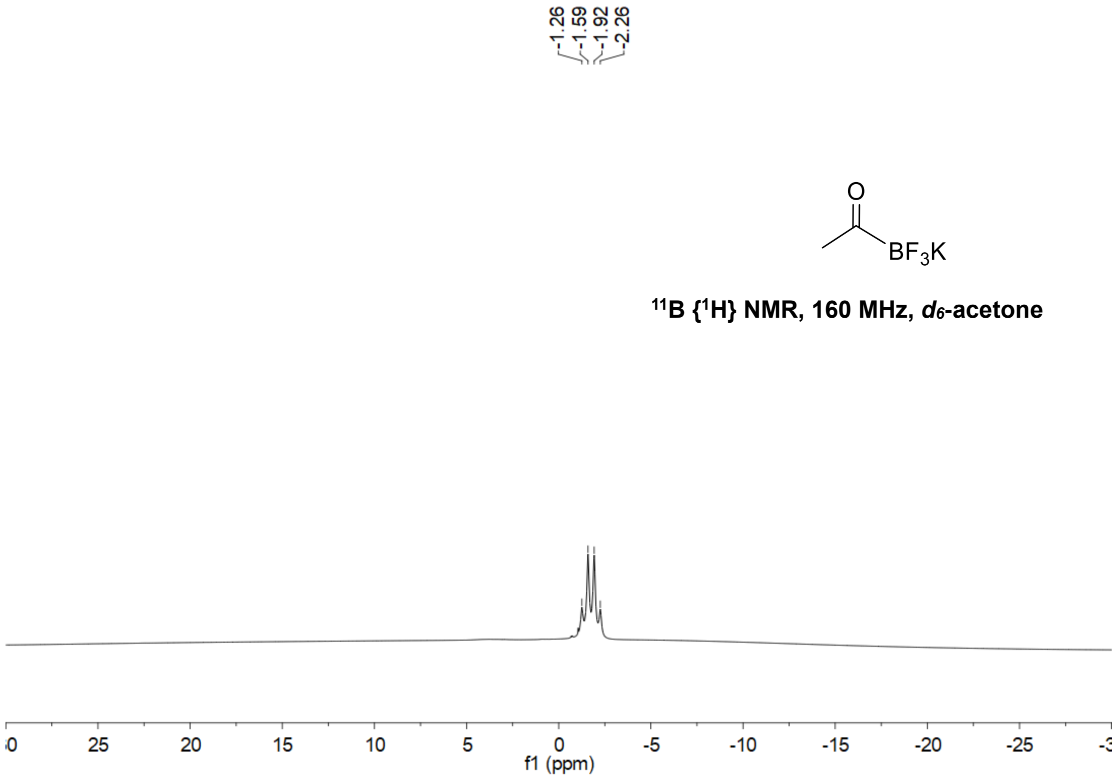
-1.26
-1.59
-1.92
-2.26
BF3K
O
11B {1H} NMR, 160 MHz, d6-acetone
25 20 15 10 5 0 -5 -10 -15 -20 -25
f1 (ppm)

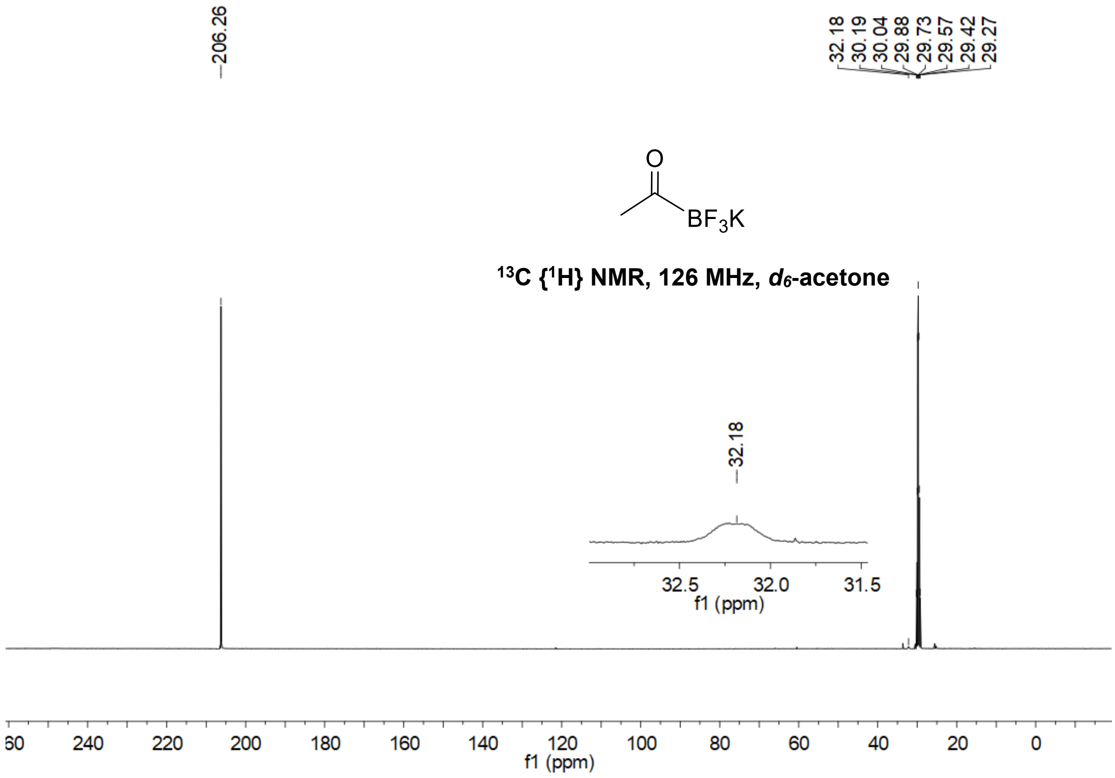
206.26
32.18
30.19
30.04
29.88
29.73
29.57
29.42
29.27
O
BF3K
13C {1H} NMR, 126 MHz, d6-acetone
32.18
32.5
32.0
31.5
f1 (ppm)
240
220
200
180
160
140
120
100
80
60
40
20
0
f1 (ppm)

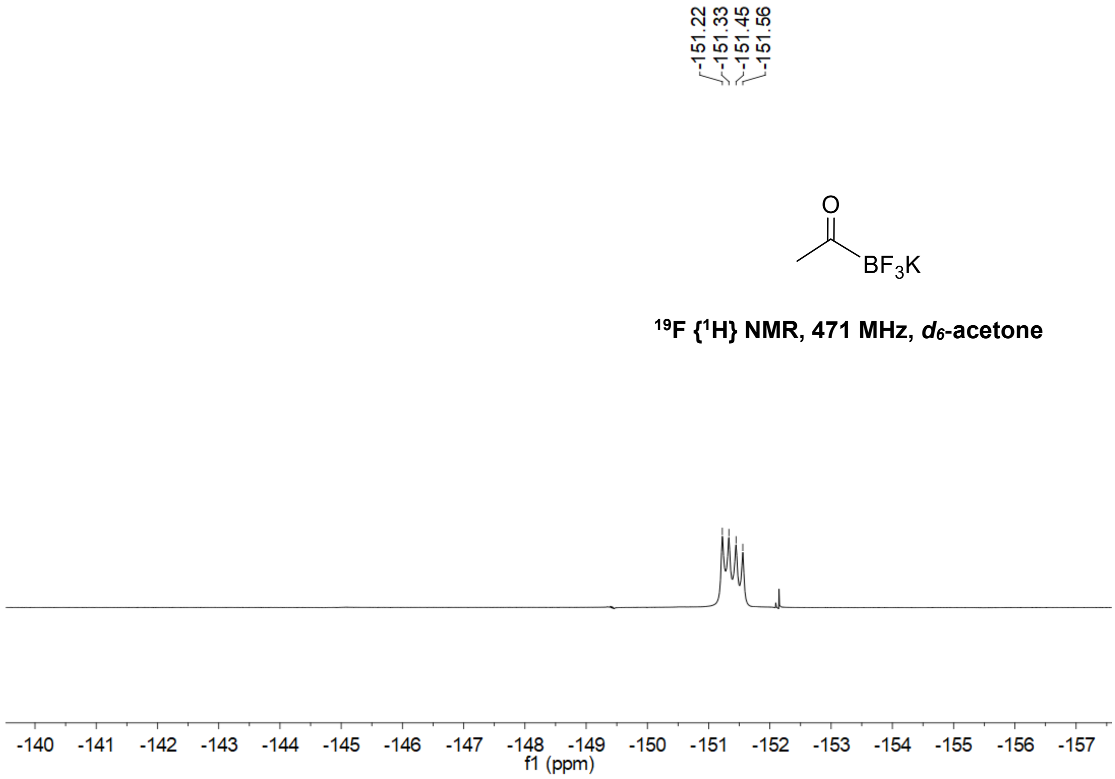
-151.22
-151.33
-151.45
-151.56
O
BF3K
19F {1H} NMR, 471 MHz, d6-acetone
-140
-141
-142
-143
-144
-145
-146
-147
-148
-149
-150
-151
-152
-153
-154
-155
-156
-157
f1 (ppm)

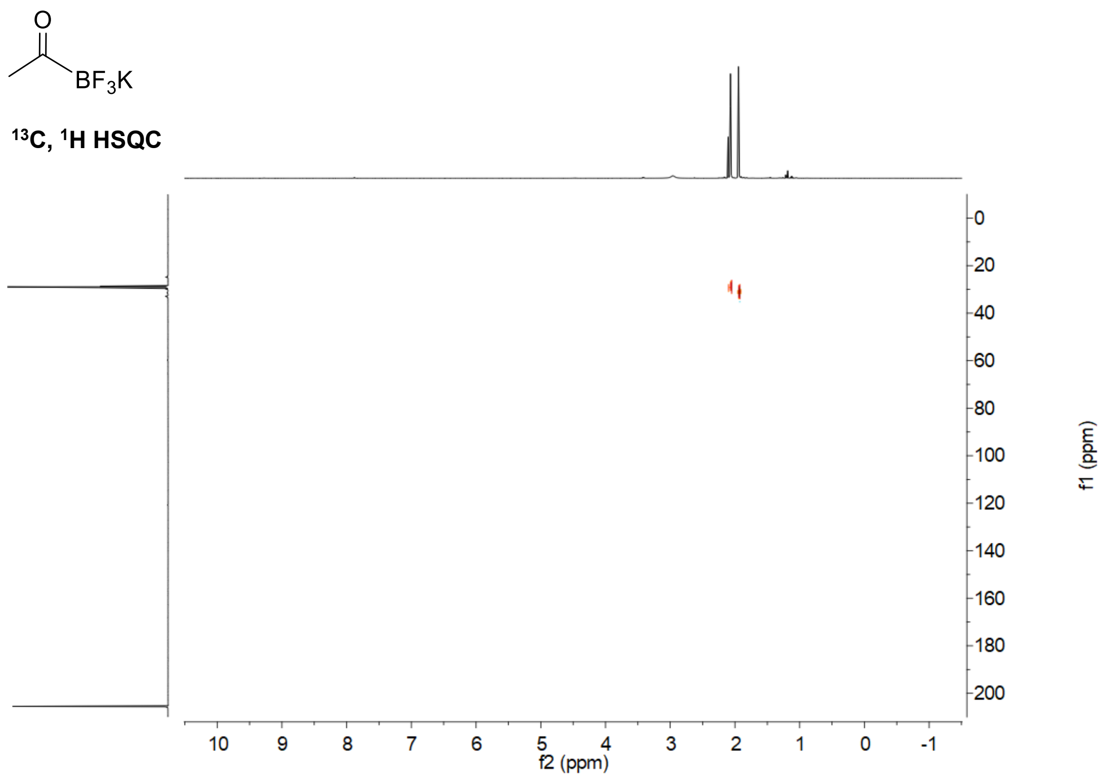
O
BF3K
13C, 1H HSQC
f1 (ppm)
f2 (ppm)

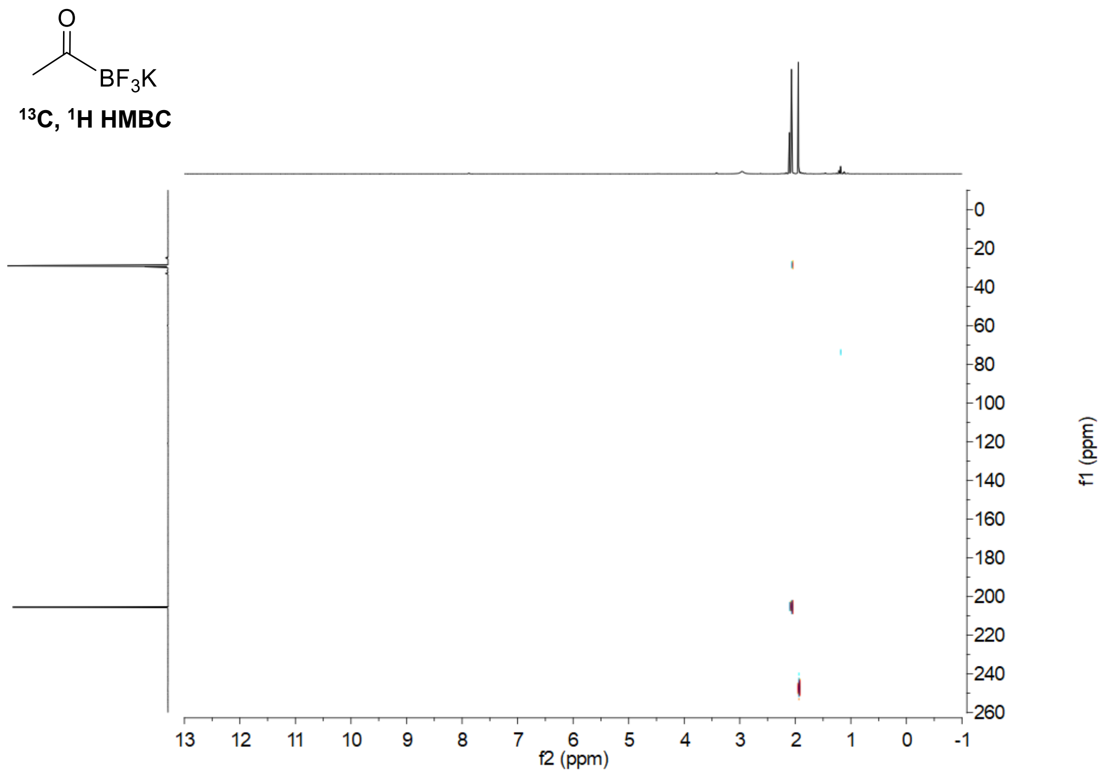
O
BF3K
13C, 1H HMBC
f1 (ppm)
f2 (ppm)

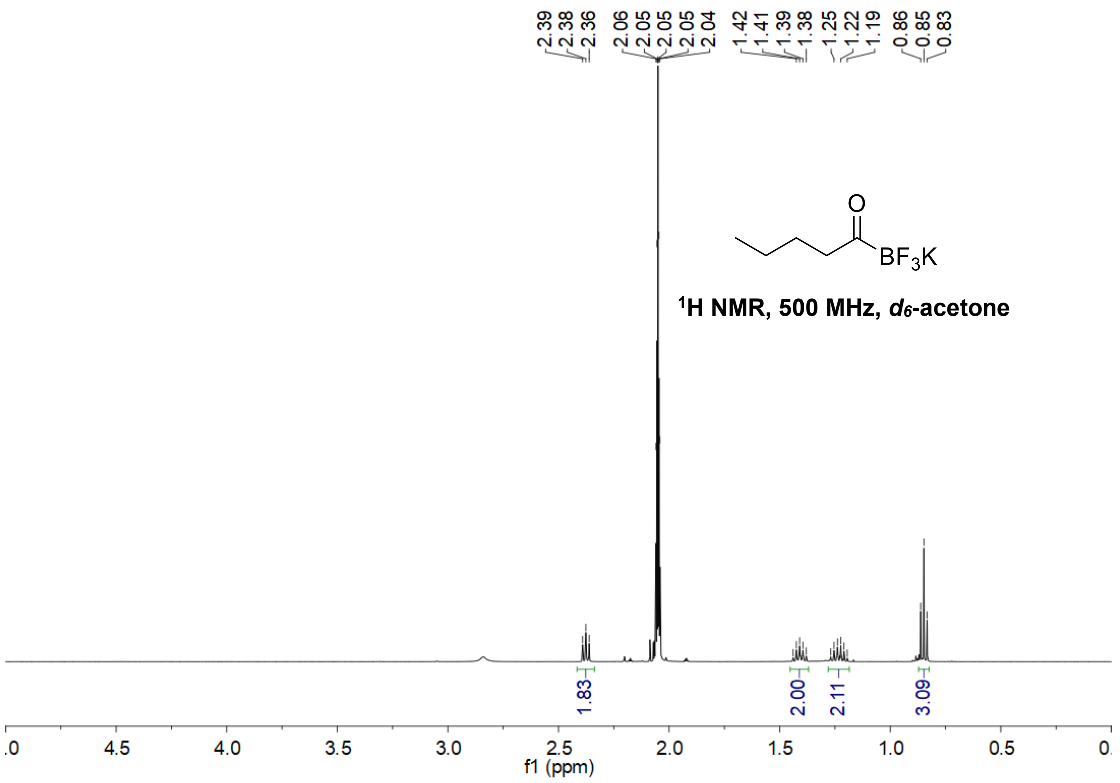
2.39
2.38
2.36
2.06
2.05
2.05
2.05
2.04
1.42
1.41
1.39
1.38
1.25
1.22
1.19
0.86
0.85
0.83
O
BF3K
1H NMR, 500 MHz, d6-acetone
1.83
2.00
2.11
3.09
f1 (ppm)

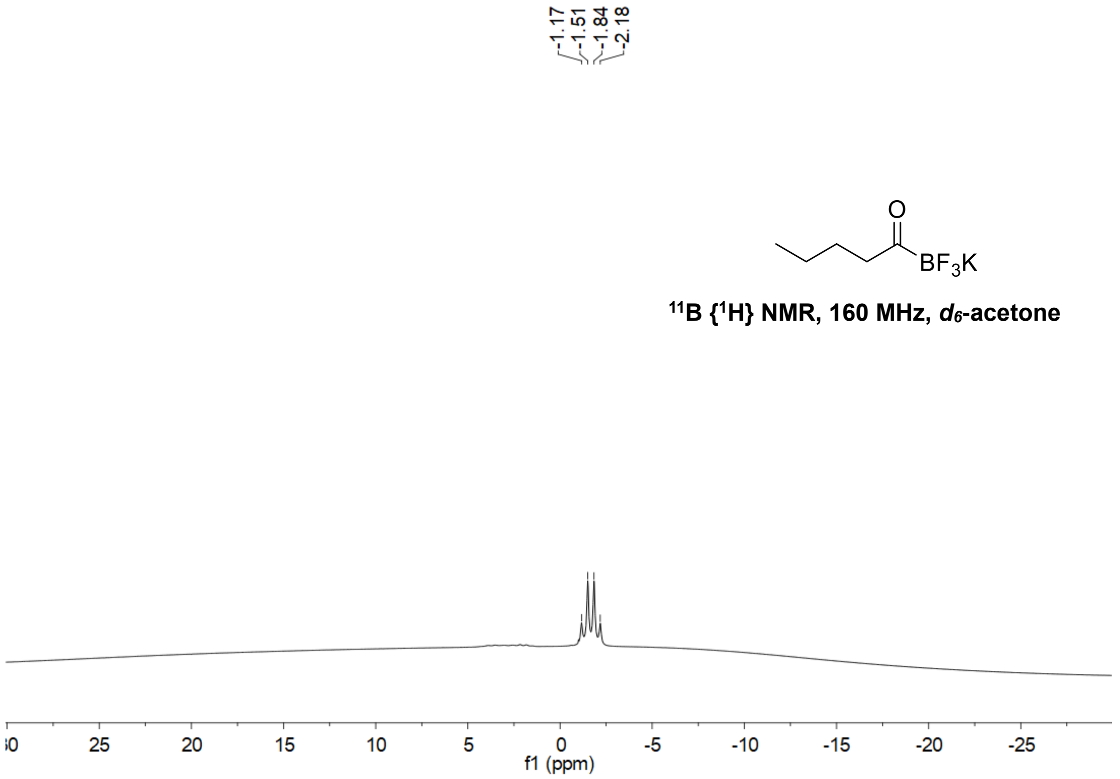
-1.17
-1.51
-1.84
-2.18
O
BF3K
11B {1H} NMR, 160 MHz, d6-acetone
f1 (ppm)

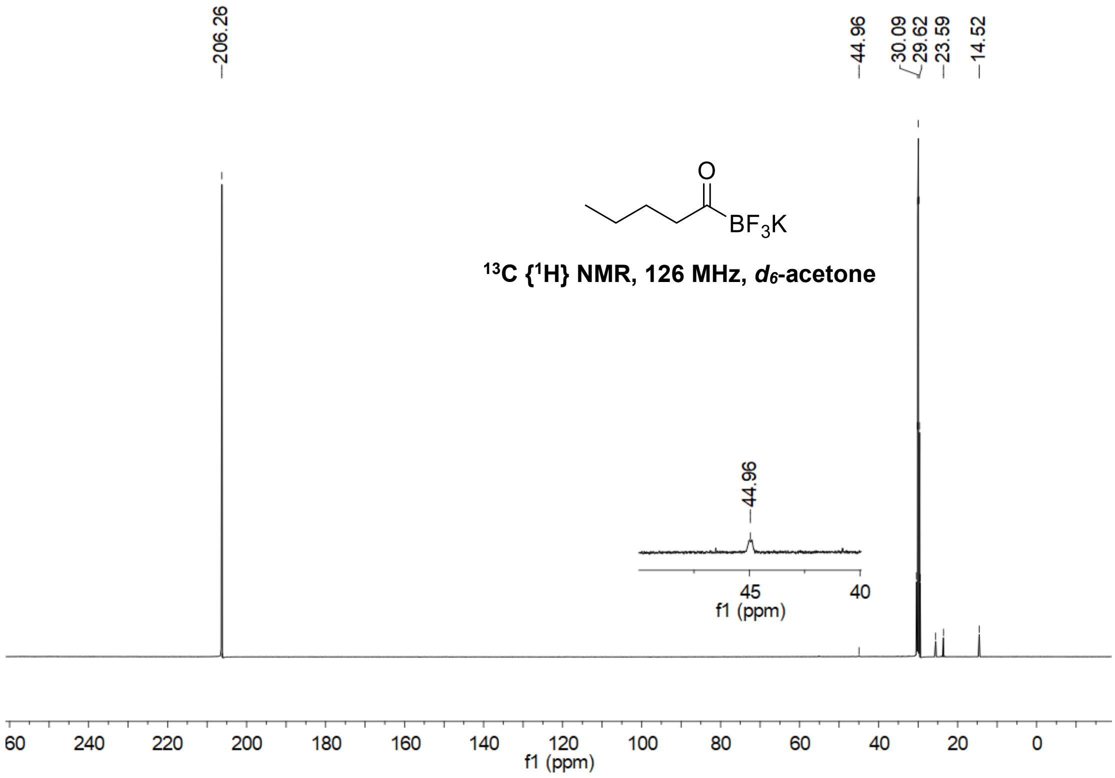

206.26
44.96
30.09
29.62
23.59
14.52
O
BF3K
13C {1H} NMR, 126 MHz, d6-acetone
44.96
45
40
f1 (ppm)
60
240
220
200
180
160
140
120
100
80
60
40
20
0
f1 (ppm)

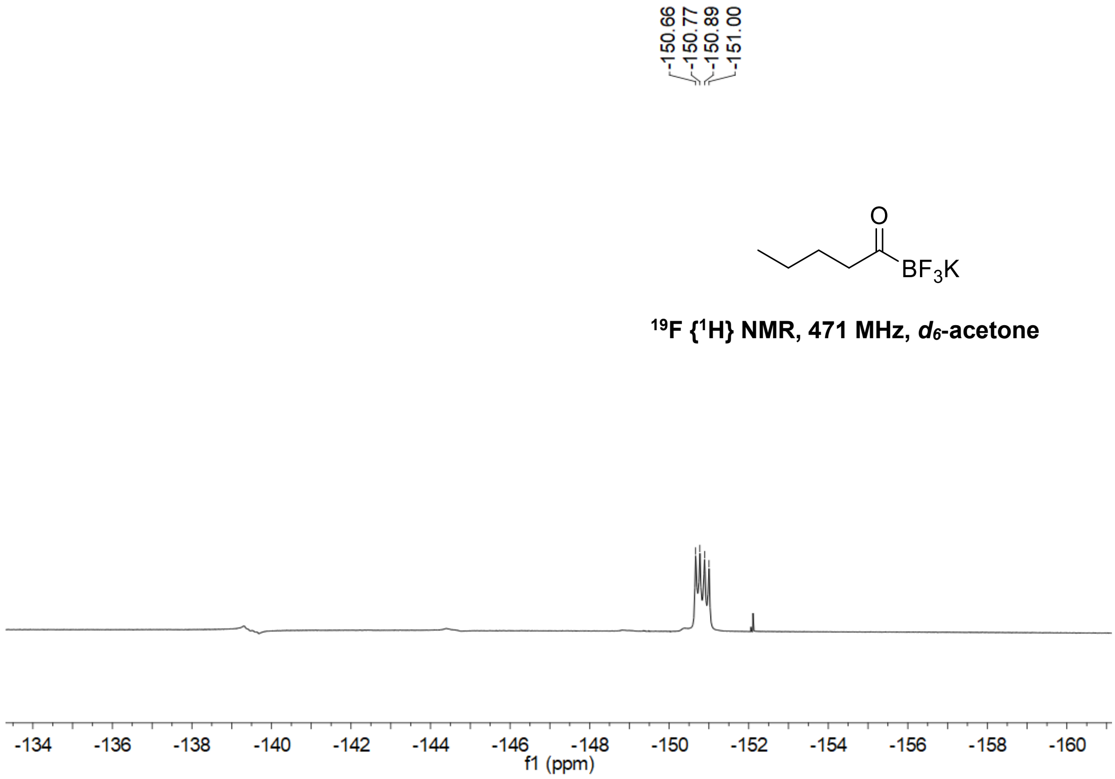

-150.66
-150.77
-150.89
-151.00
O
BF3K
19F {1H} NMR, 471 MHz, d6-acetone
-134
-136
-138
-140
-142
-144
-146
-148
-150
-152
-154
-156
-158
-160
f1 (ppm)

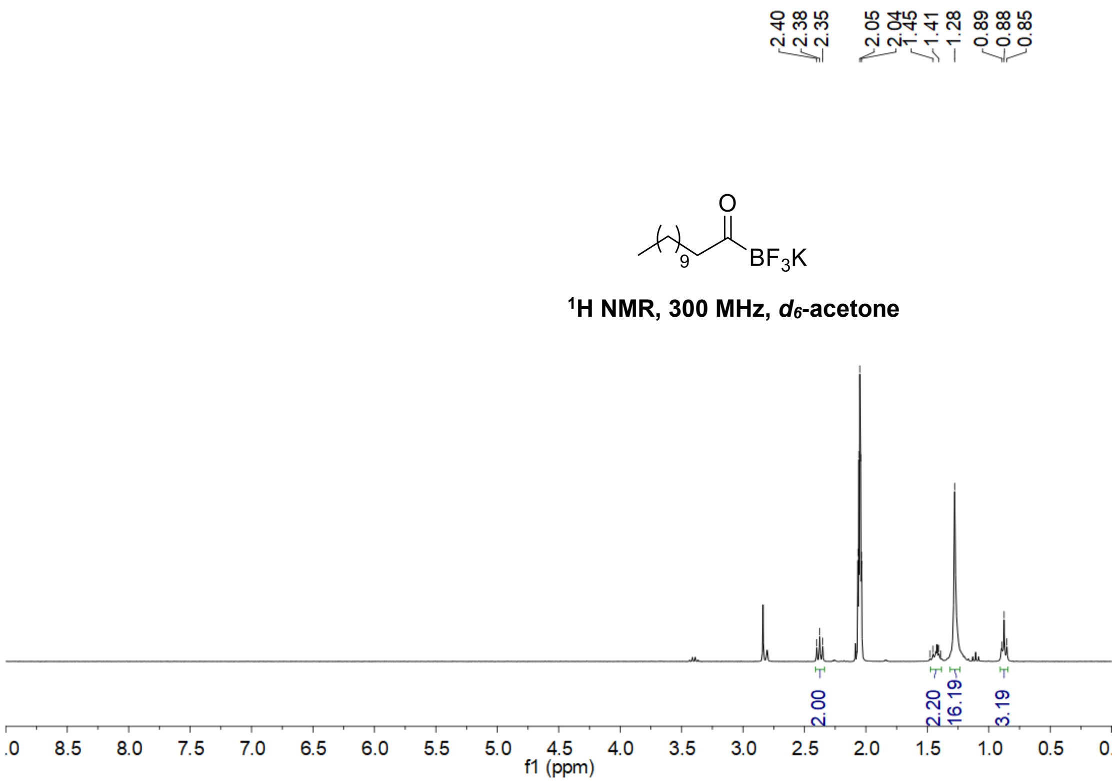

^{1}H NMR, 300 MHz, *d$_6$*-acetone

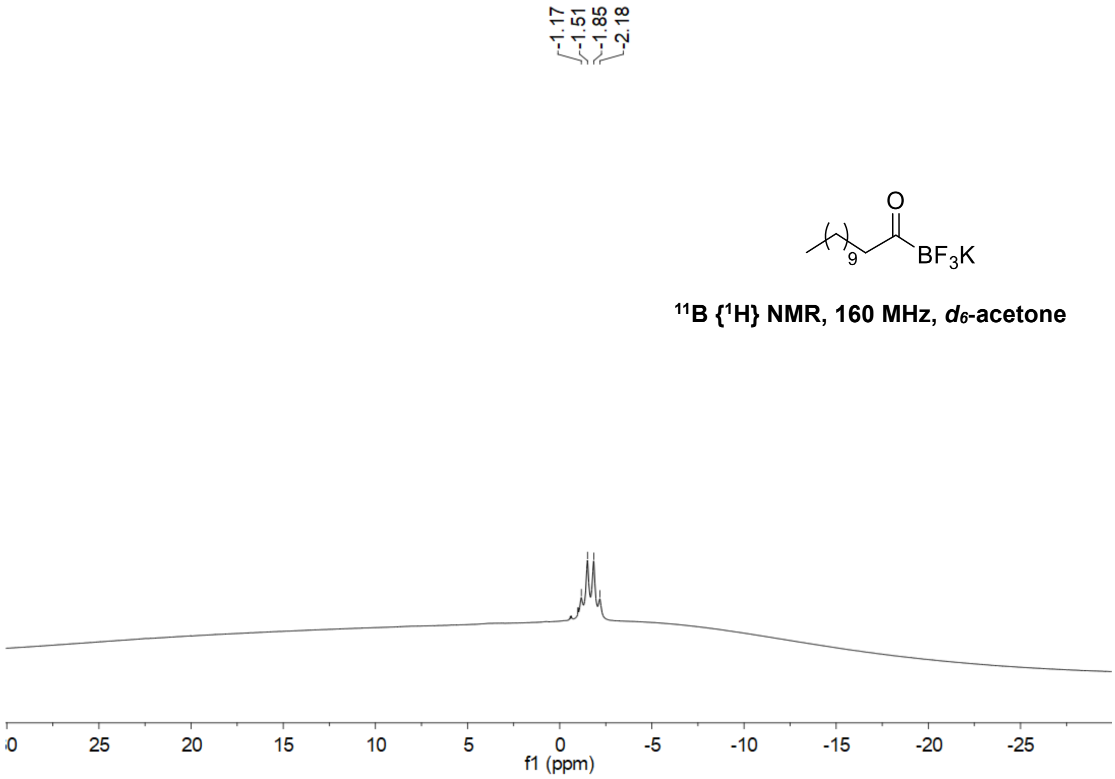

^{11}B {^{1}H} NMR, 160 MHz, *d$_6$*-acetone

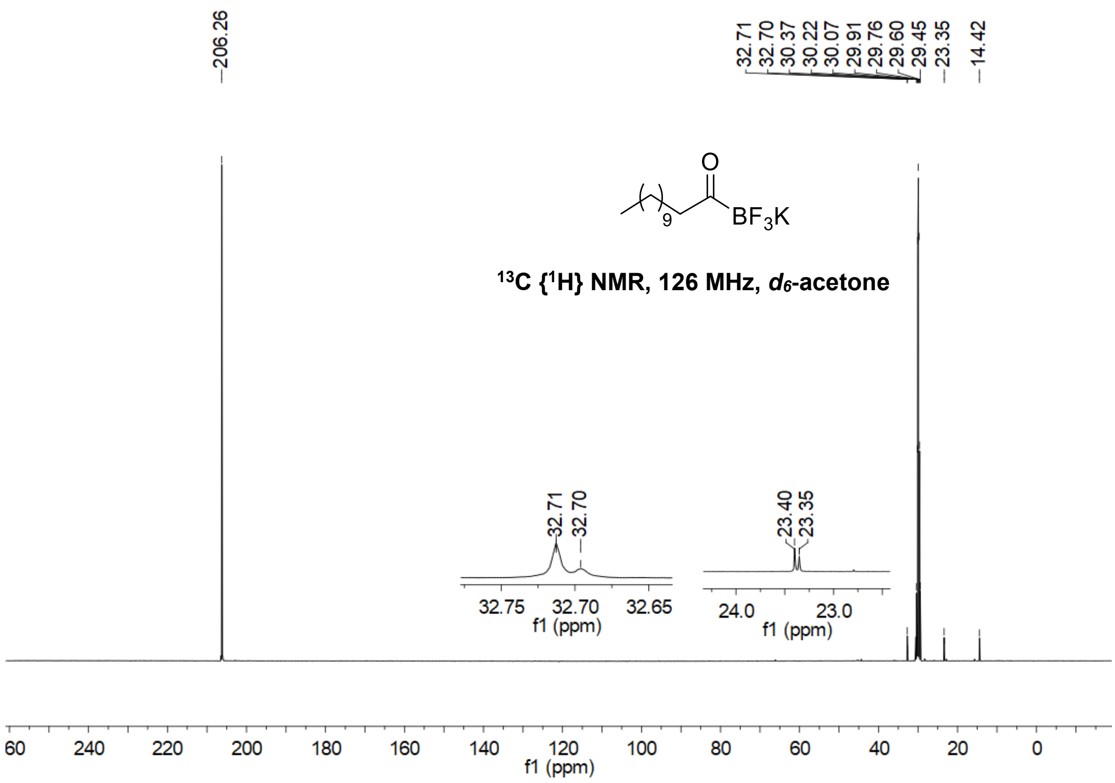

206.26
32.71
32.70
30.37
30.22
30.07
29.91
29.76
29.60
29.45
23.35
14.42
O
9
BF3K
13C {1H} NMR, 126 MHz, d6-acetone
32.71
32.70
32.75
32.70
32.65
f1 (ppm)
23.40
23.35
24.0
23.0
f1 (ppm)
60
240
220
200
180
160
140
120
100
80
60
40
20
0
f1 (ppm)

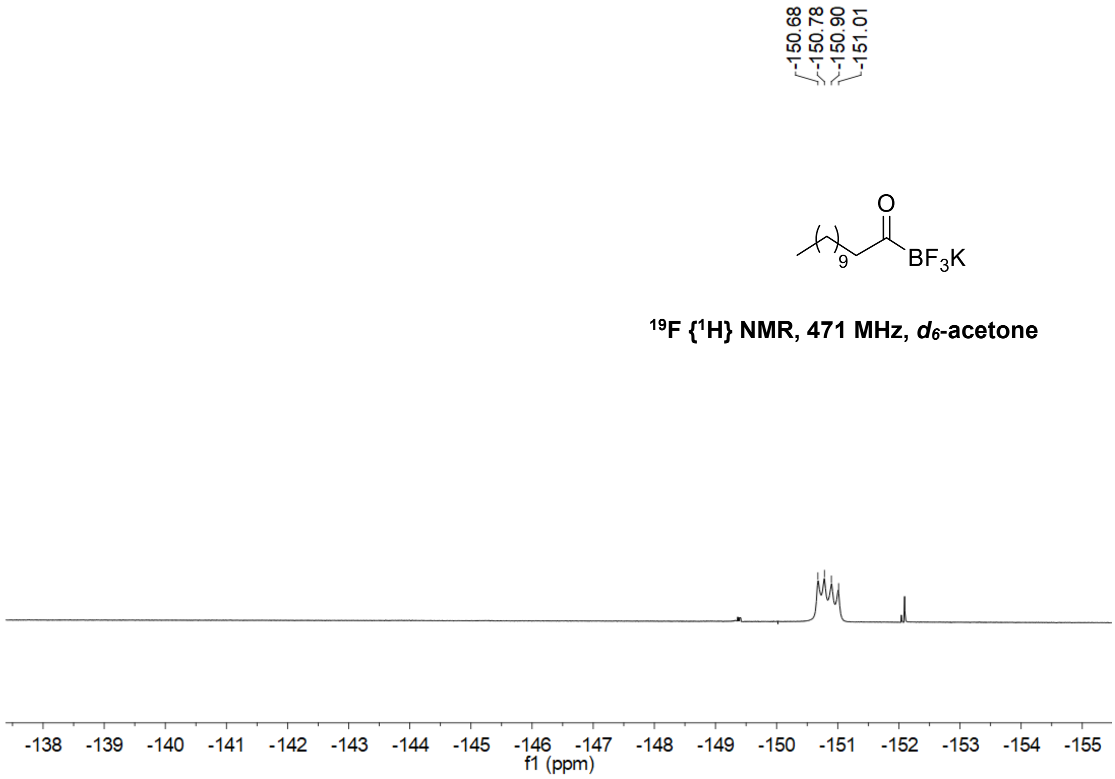

-150.68
-150.78
-150.90
-151.01
O
9
BF3K
19F {1H} NMR, 471 MHz, d6-acetone
-138
-139
-140
-141
-142
-143
-144
-145
-146
-147
-148
-149
-150
-151
-152
-153
-154
-155
f1 (ppm)

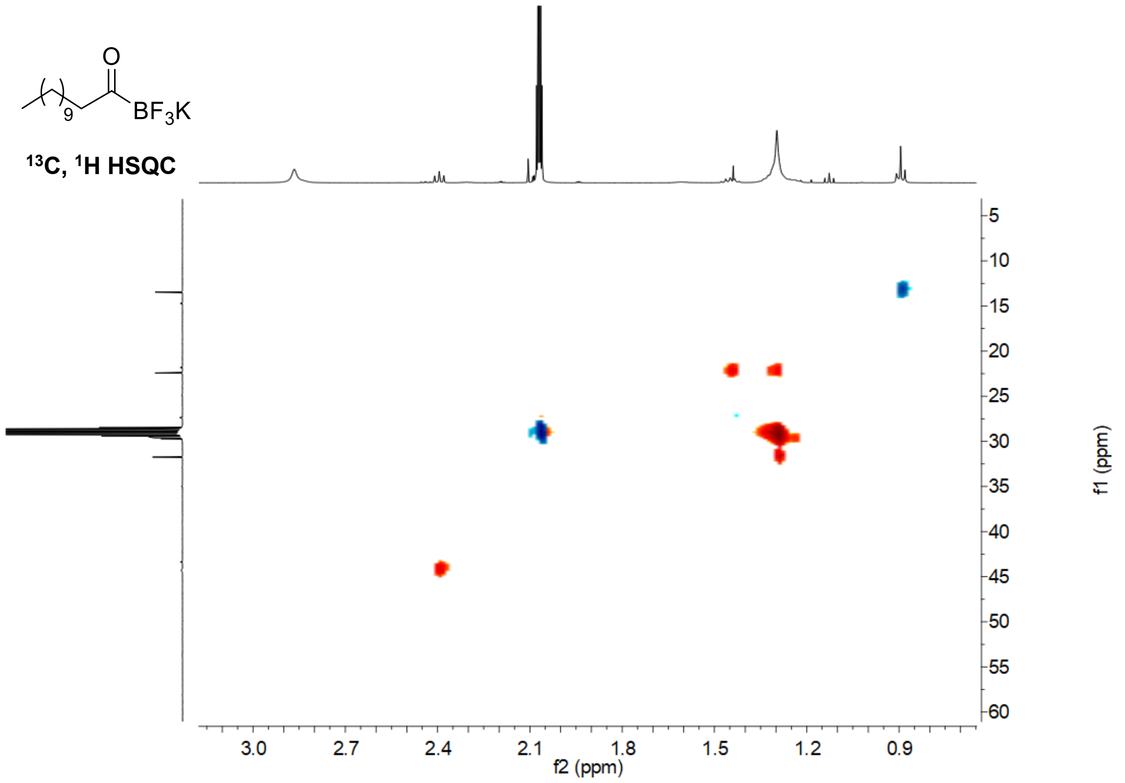
O
9
BF3K
13C, 1H HSQC
f2 (ppm)
f1 (ppm)

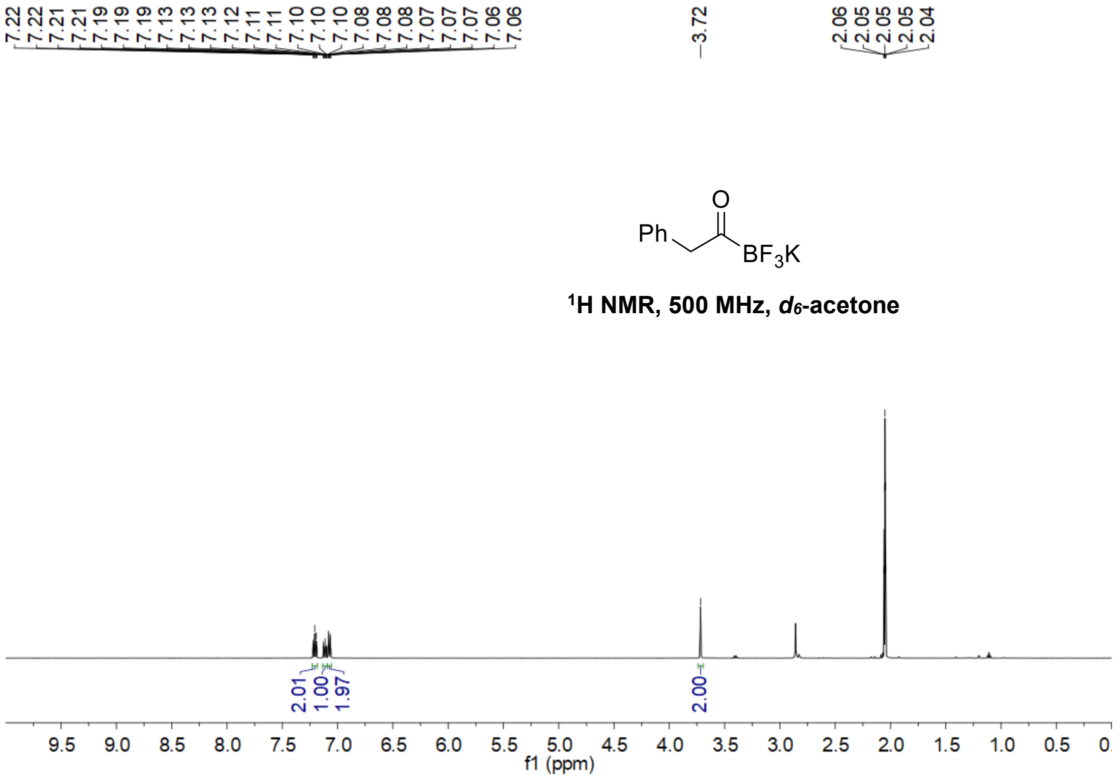
Ph
O
BF3K
1H NMR, 500 MHz, d6-acetone
f1 (ppm)

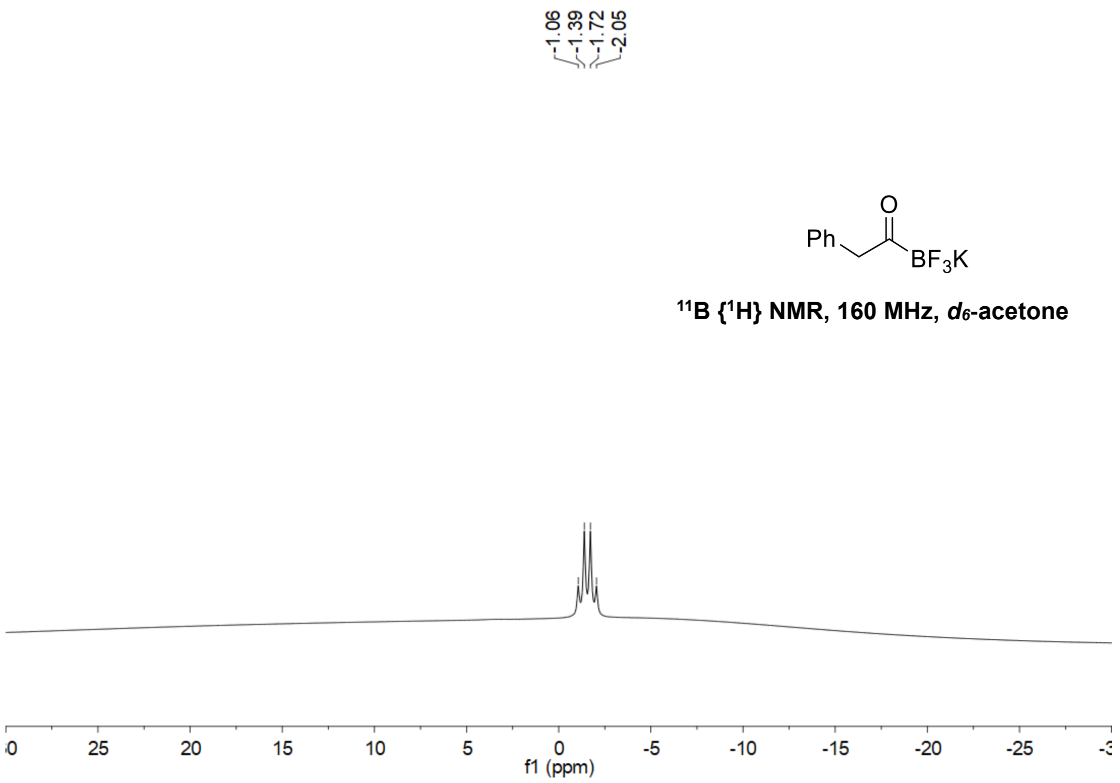

^{11}B {^{1}H} NMR, 160 MHz, *d6*-acetone

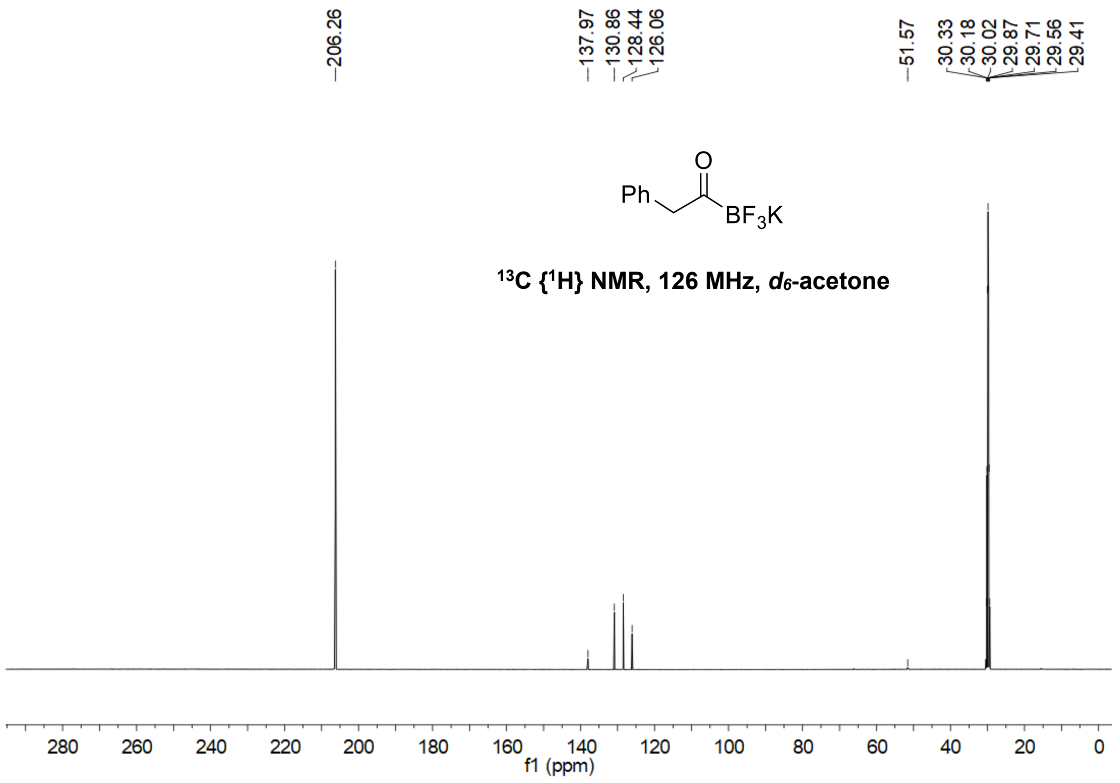

^{13}C {^{1}H} NMR, 126 MHz, *d6*-acetone

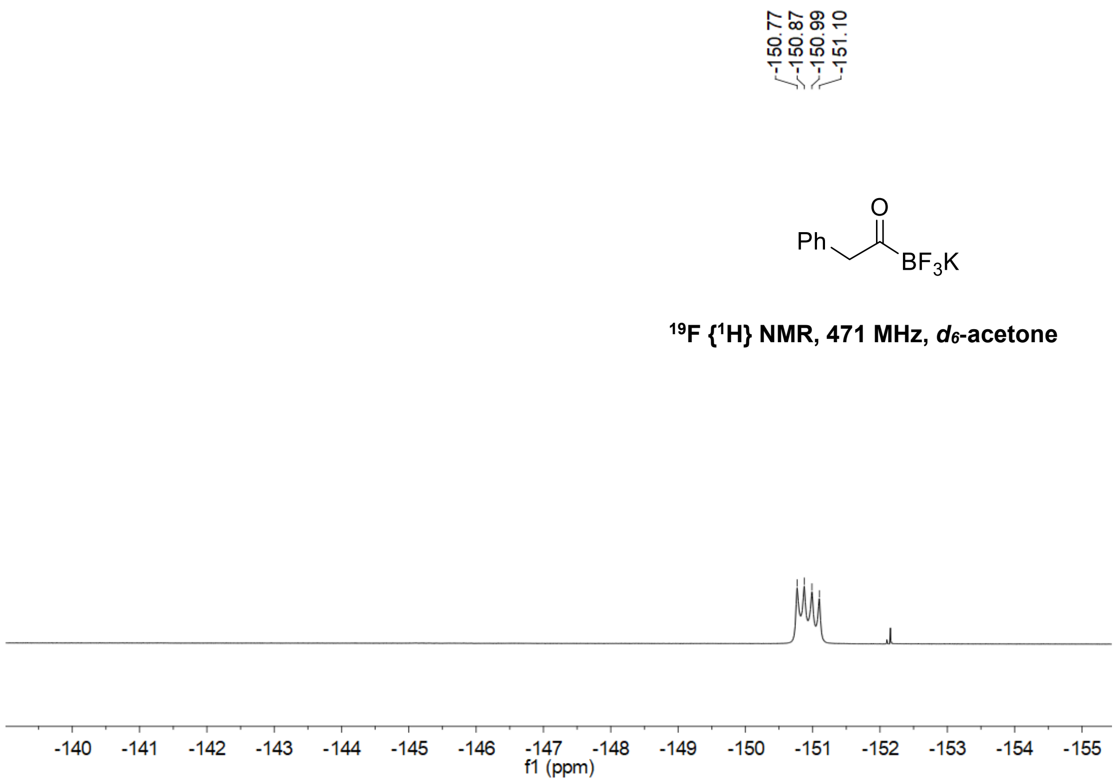

^{19}F {^{1}H} NMR, 471 MHz, d_6-acetone

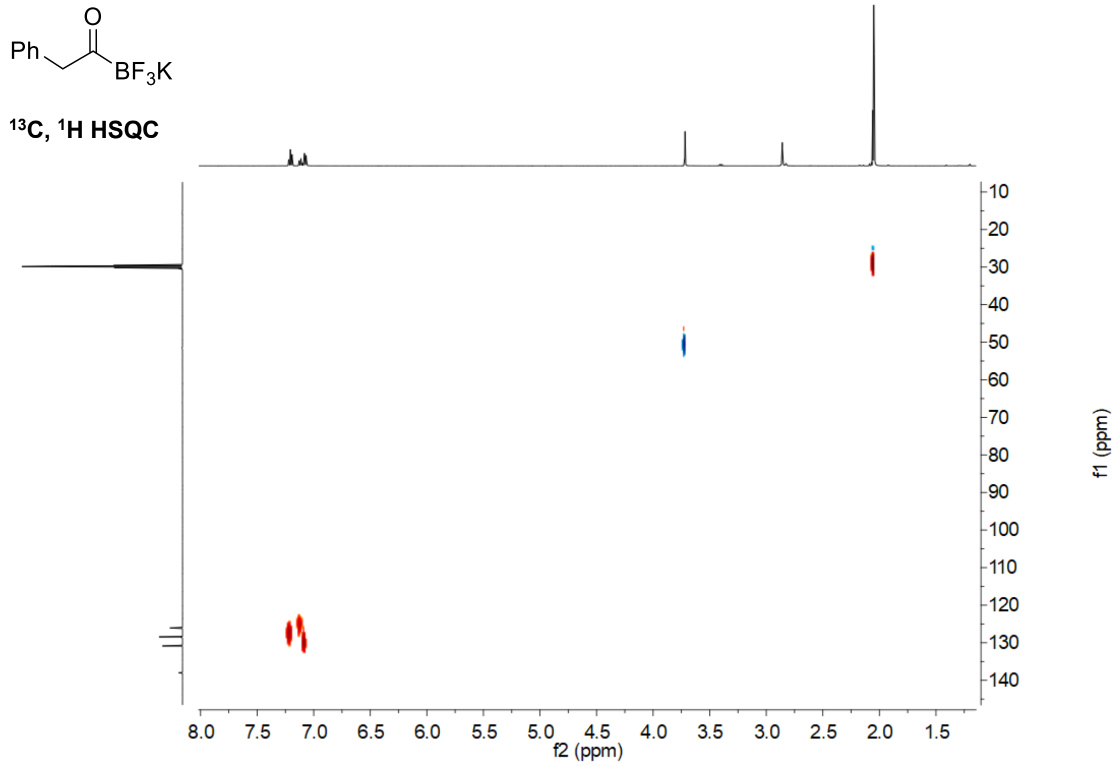

^{13}C, ^{1}H HSQC

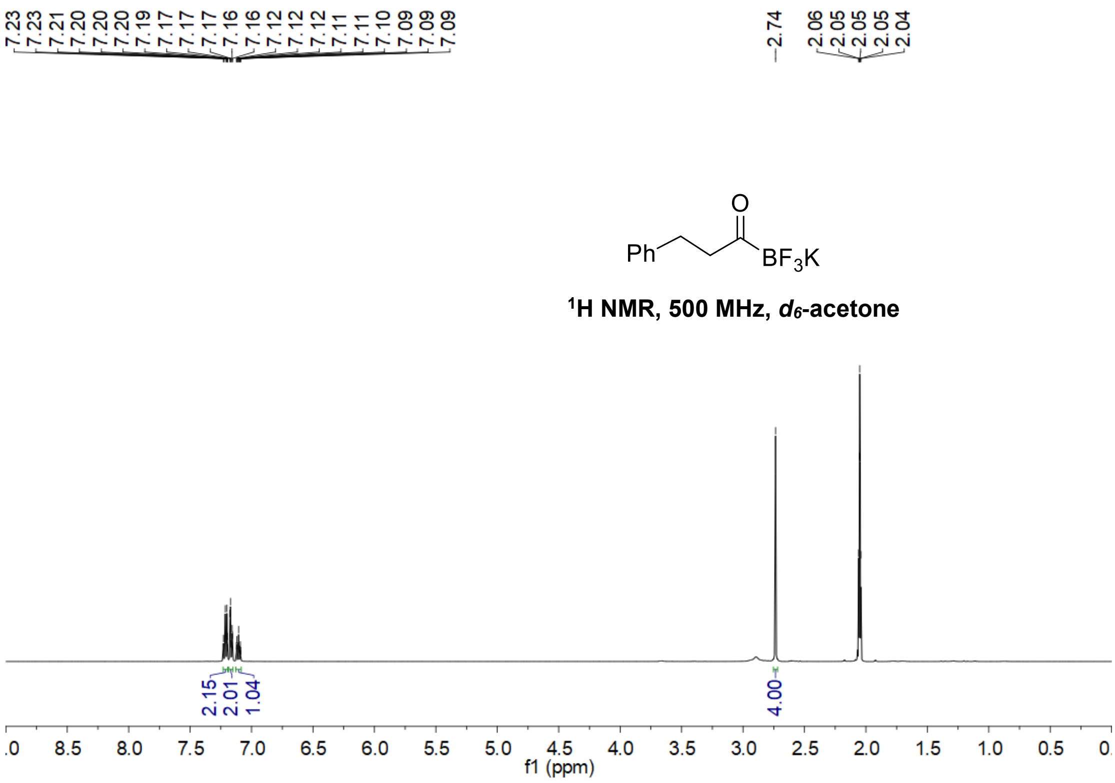
7.23
7.23
7.21
7.20
7.20
7.20
7.19
7.17
7.17
7.17
7.16
7.16
7.12
7.12
7.12
7.11
7.10
7.09
7.09
7.09
2.74
2.06
2.05
2.05
2.05
2.04
Ph
O
BF3K
¹H NMR, 500 MHz, d6-acetone
2.15
2.01
1.04
4.00
8.5
8.0
7.5
7.0
6.5
6.0
5.5
5.0
4.5
4.0
3.5
3.0
2.5
2.0
1.5
1.0
0.5
f1 (ppm)

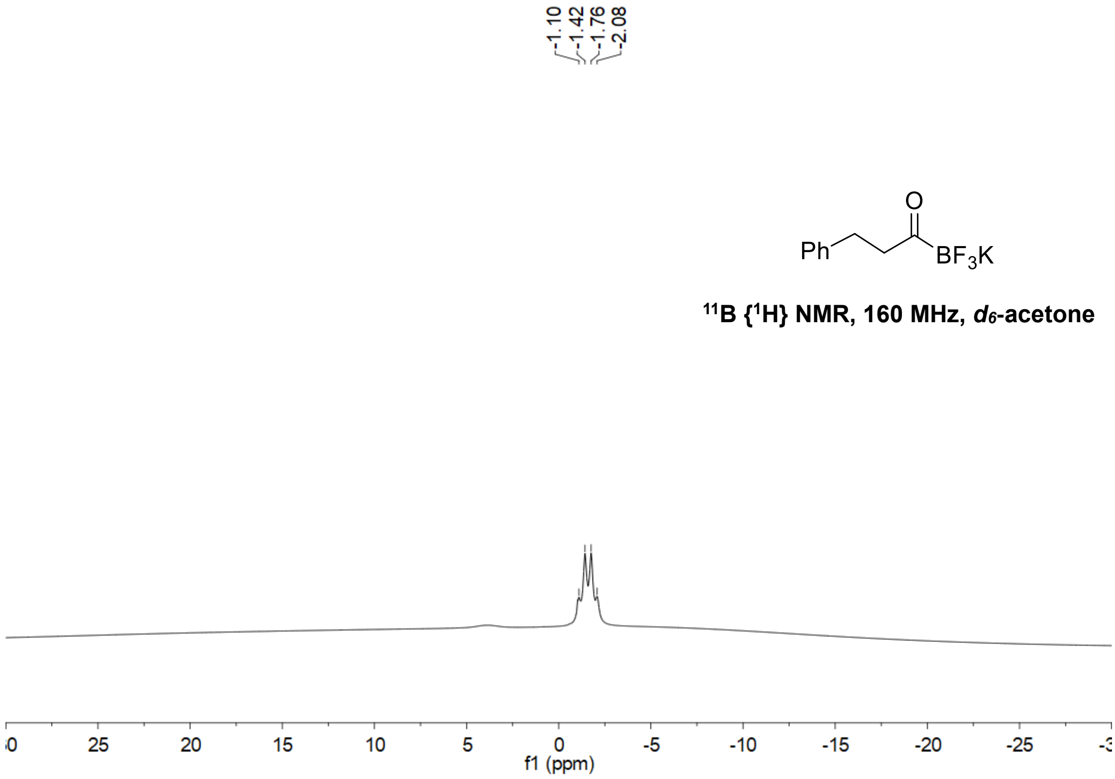
-1.10
-1.42
-1.76
-2.08
Ph
O
BF3K
¹¹B {¹H} NMR, 160 MHz, d6-acetone
25
20
15
10
5
0
-5
-10
-15
-20
-25
f1 (ppm)

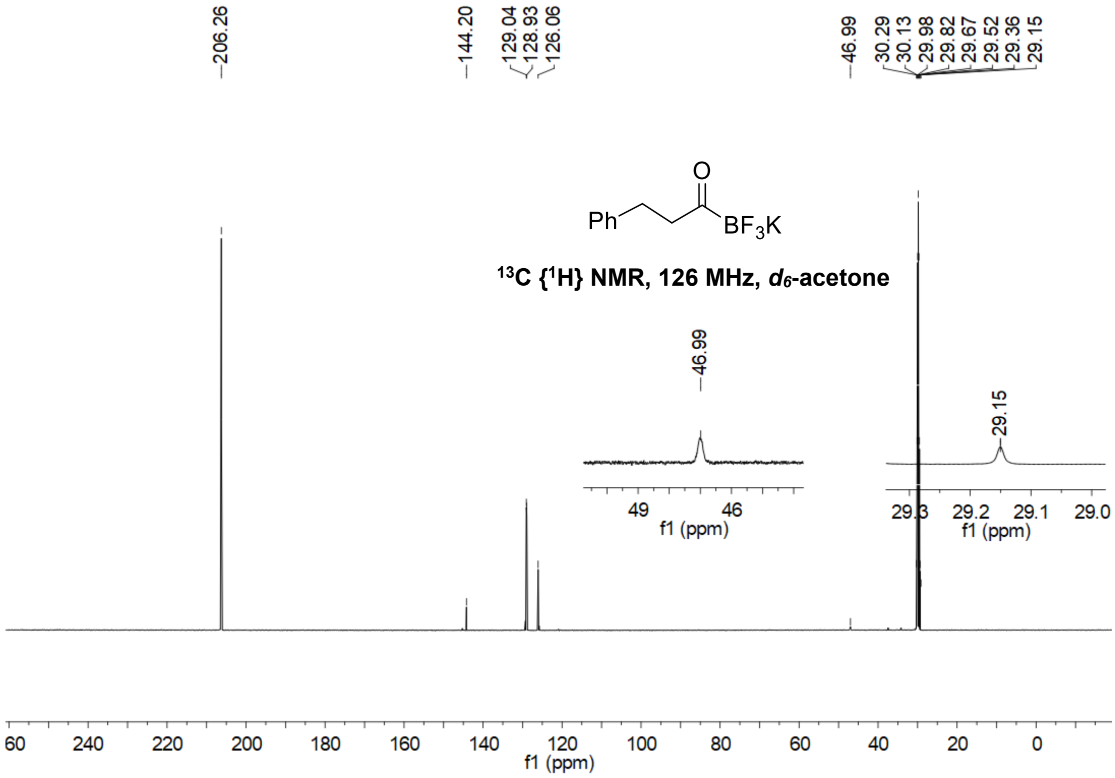
206.26
144.20
129.04
128.93
126.06
46.99
30.29
30.13
29.98
29.82
29.67
29.52
29.36
29.15
Ph
O
BF3K
13C {1H} NMR, 126 MHz, d6-acetone
46.99
49
46
f1 (ppm)
29.15
29.3
29.2
29.1
29.0
f1 (ppm)
60
240
220
200
180
160
140
120
100
80
60
40
20
0
f1 (ppm)

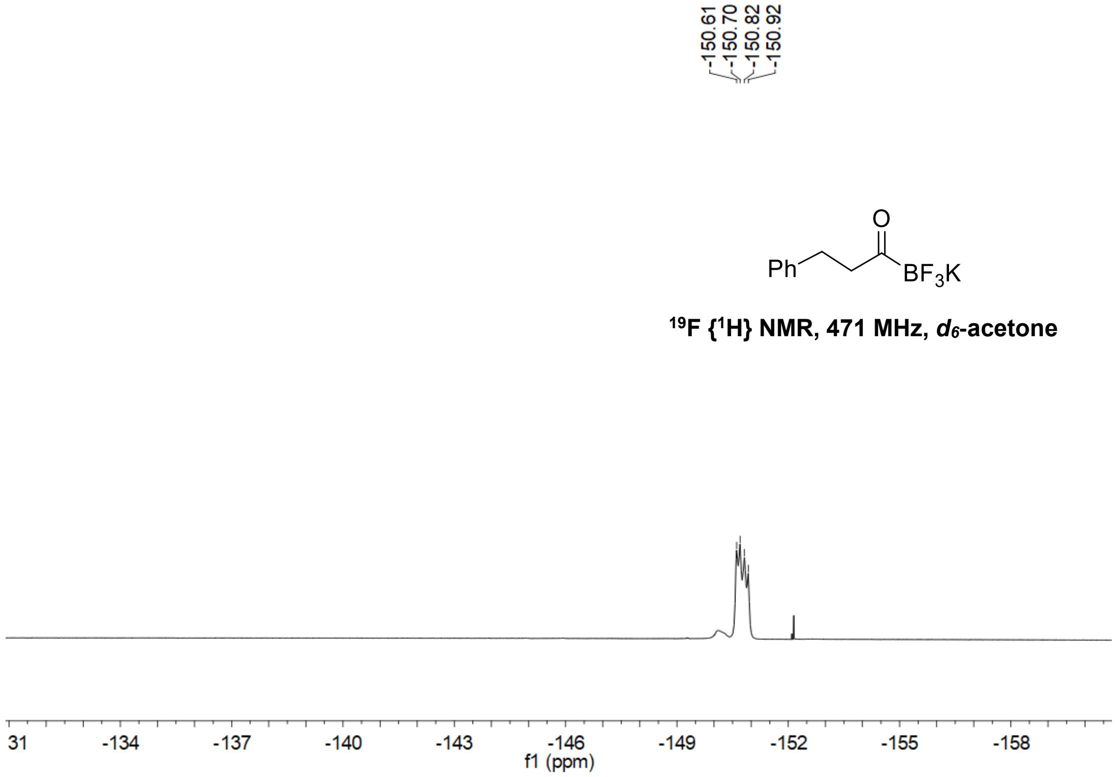
-150.61
-150.70
-150.82
-150.92
Ph
O
BF3K
19F {1H} NMR, 471 MHz, d6-acetone
31
-134
-137
-140
-143
-146
-149
-152
-155
-158
f1 (ppm)

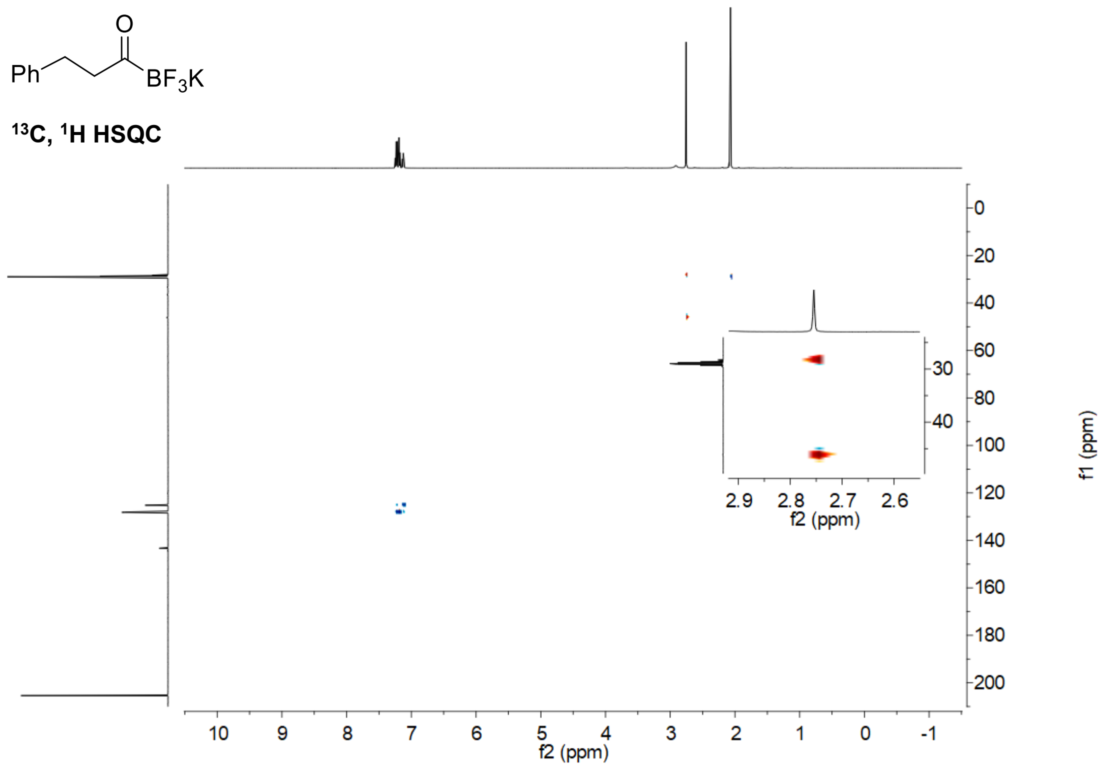
Ph
O
BF3K
13C, 1H HSQC
f1 (ppm)
f2 (ppm)

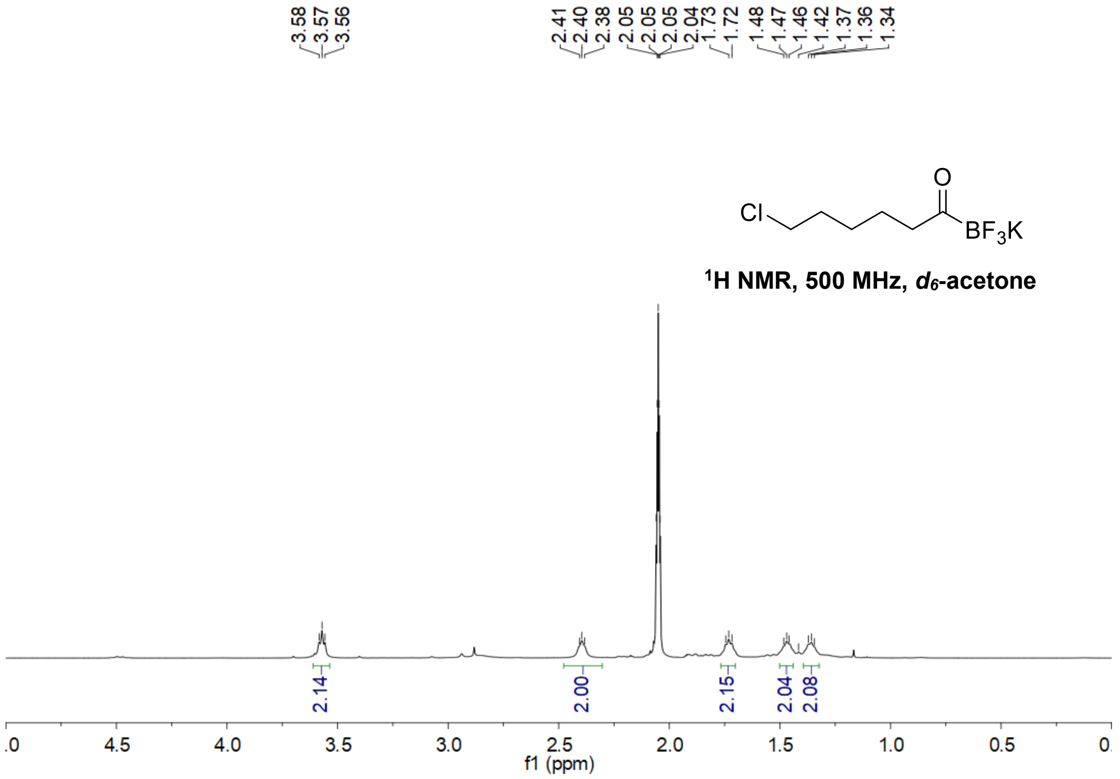
3.58
3.57
3.56
2.41
2.40
2.38
2.05
2.05
2.05
2.04
1.73
1.72
1.48
1.47
1.46
1.42
1.37
1.36
1.34
Cl
O
BF3K
1H NMR, 500 MHz, d6-acetone
2.14
2.00
2.15
2.04
2.08
f1 (ppm)

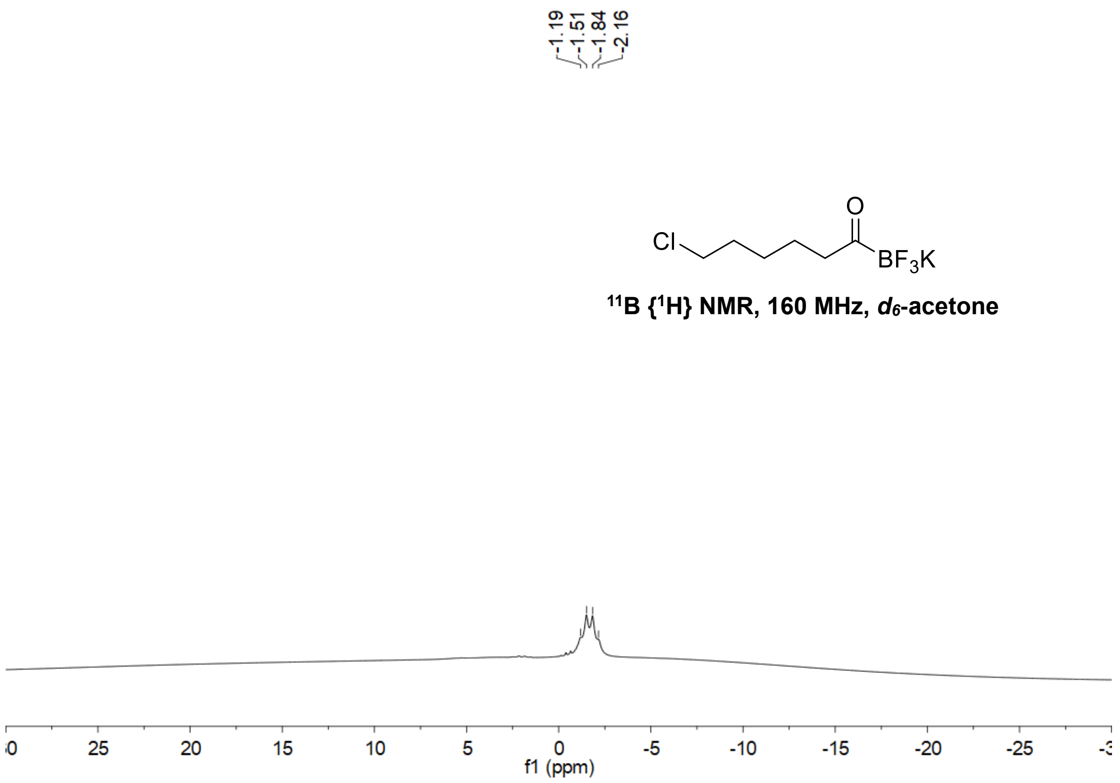
-1.19
-1.51
-1.84
-2.16
Cl
O
BF3K
11B {1H} NMR, 160 MHz, d6-acetone
25
20
15
10
5
0
-5
-10
-15
-20
-25
f1 (ppm)

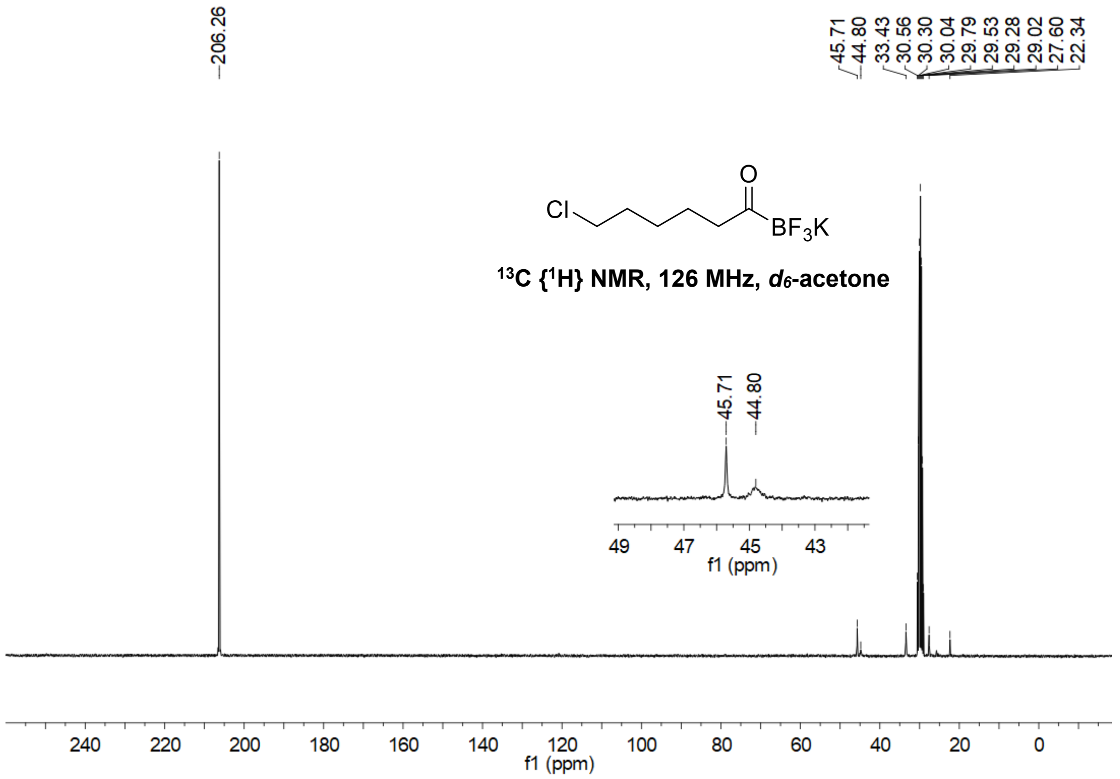
206.26
45.71
44.80
33.43
30.56
30.30
30.04
29.79
29.53
29.28
29.02
27.60
22.34
Cl
O
BF3K
13C {1H} NMR, 126 MHz, d6-acetone
45.71
44.80
49
47
45
43
f1 (ppm)
240
220
200
180
160
140
120
100
80
60
40
20
0
f1 (ppm)

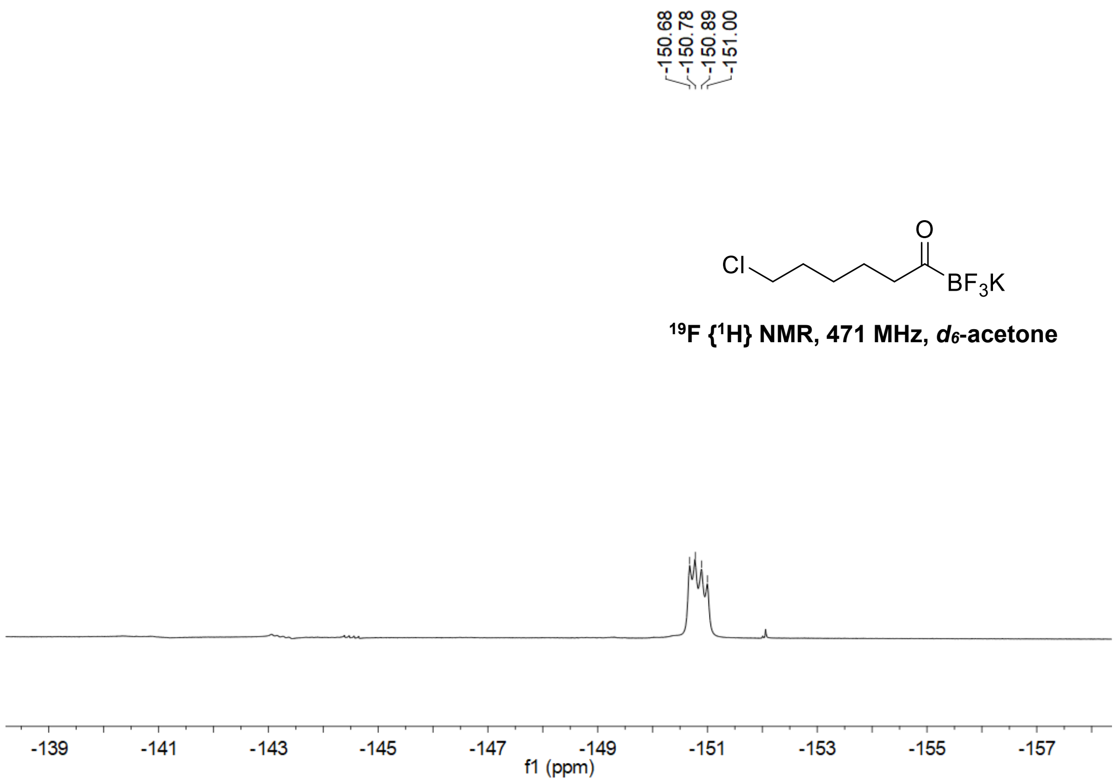

^{19}F {^{1}H} NMR, 471 MHz, *d*$_{6}$-acetone

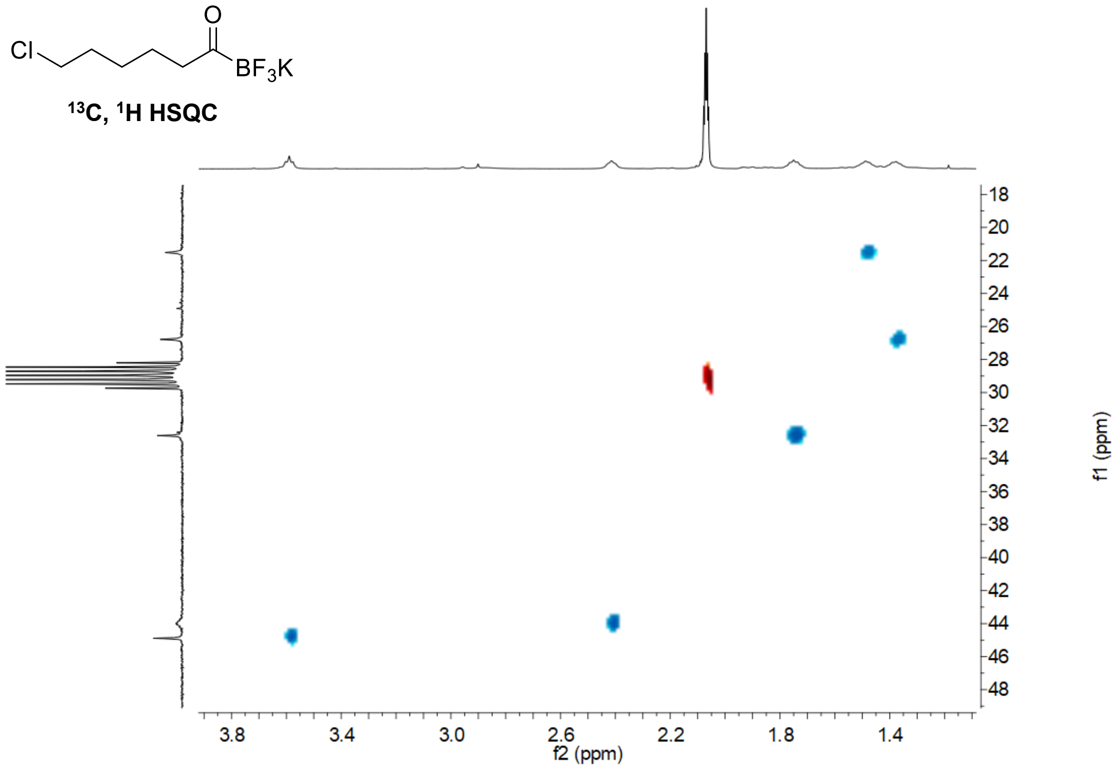

^{13}C, ^{1}H HSQC

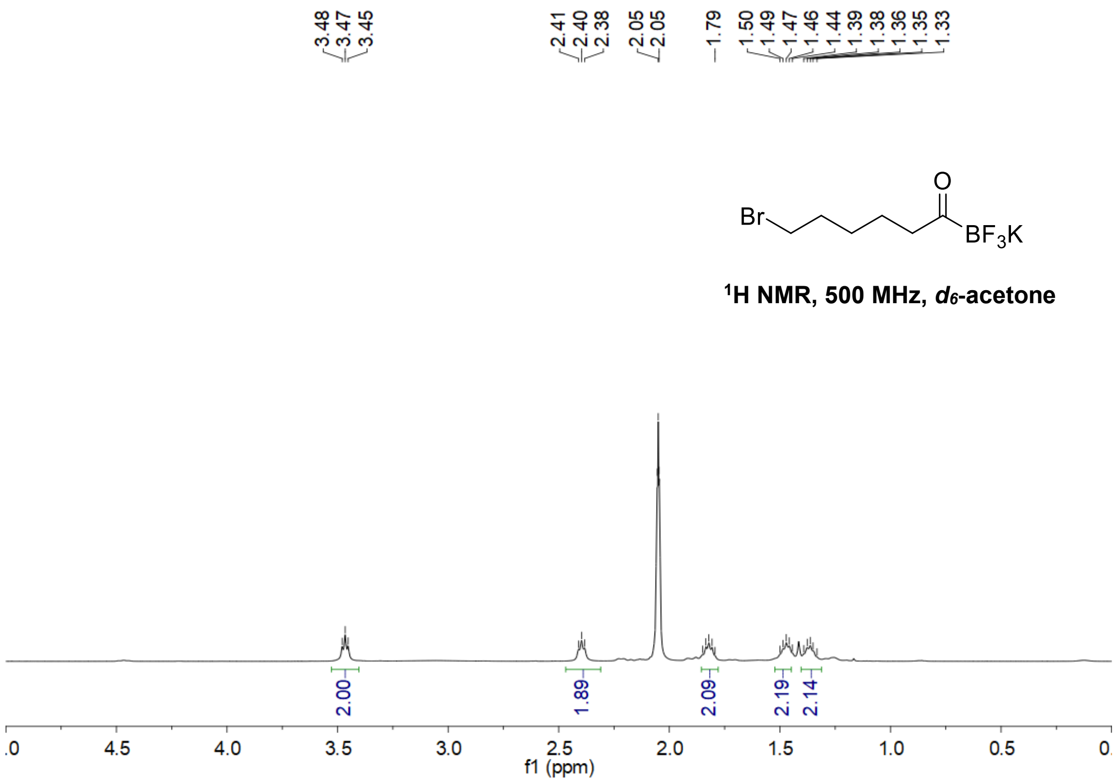
3.48
3.47
3.45
2.41
2.40
2.38
2.05
2.05
1.79
1.50
1.49
1.47
1.46
1.44
1.39
1.38
1.36
1.35
1.33
O
Br
BF3K
1H NMR, 500 MHz, d6-acetone
2.00
1.89
2.09
2.19
2.14
4.5
4.0
3.5
3.0
2.5
2.0
1.5
1.0
0.5
f1 (ppm)

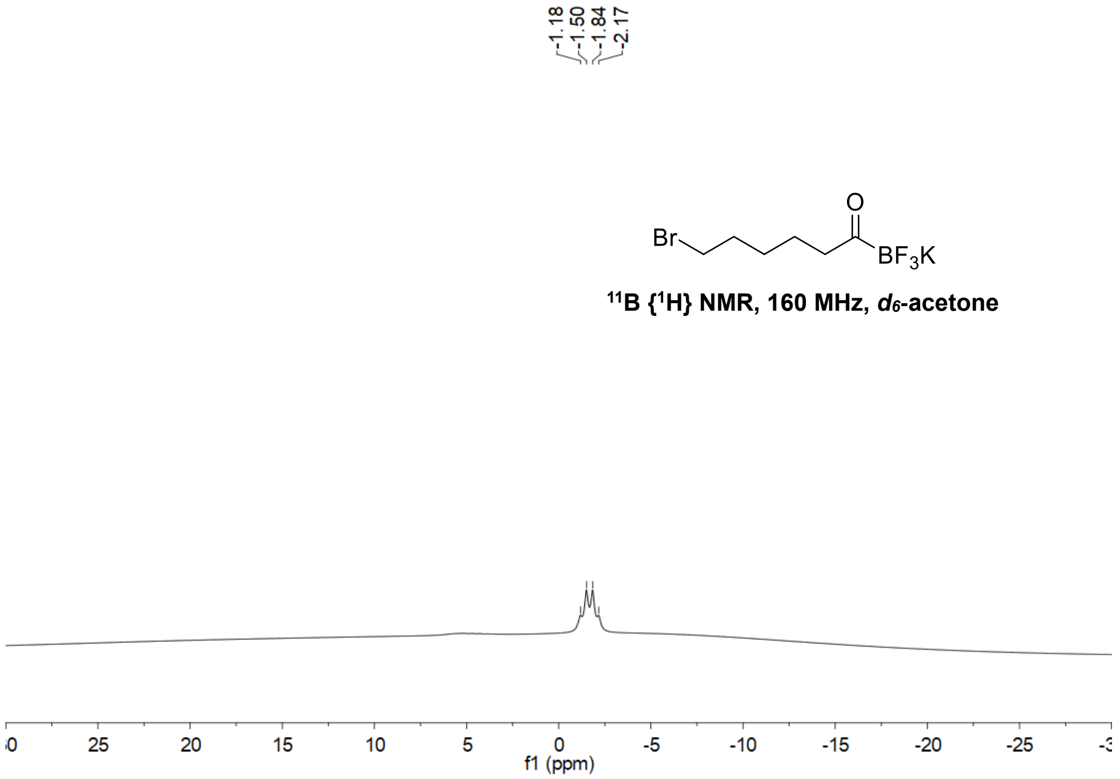
-1.18
-1.50
-1.84
-2.17
O
Br
BF3K
11B {1H} NMR, 160 MHz, d6-acetone
25
20
15
10
5
0
-5
-10
-15
-20
-25
f1 (ppm)

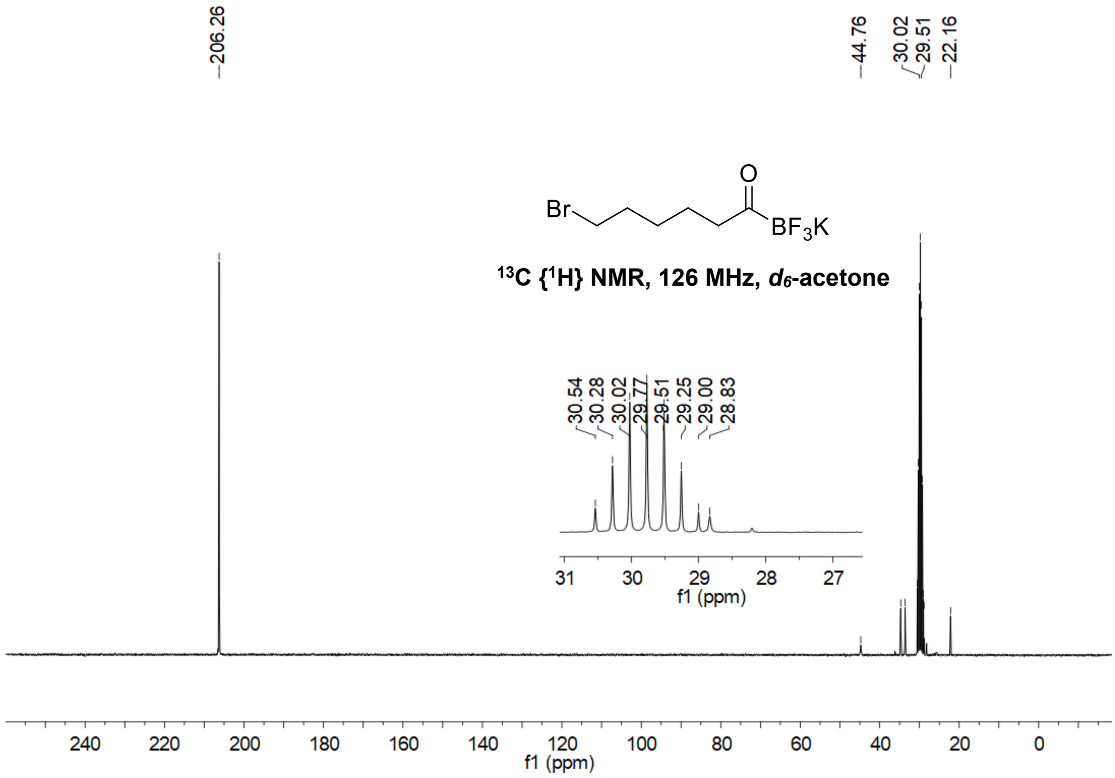
206.26
44.76
30.02
29.51
22.16
Br
O
BF3K
13C {1H} NMR, 126 MHz, d6-acetone
30.54
30.28
30.02
29.77
29.51
29.25
29.00
28.83
31 30 29 28 27
f1 (ppm)
240 220 200 180 160 140 120 100 80 60 40 20 0
f1 (ppm)

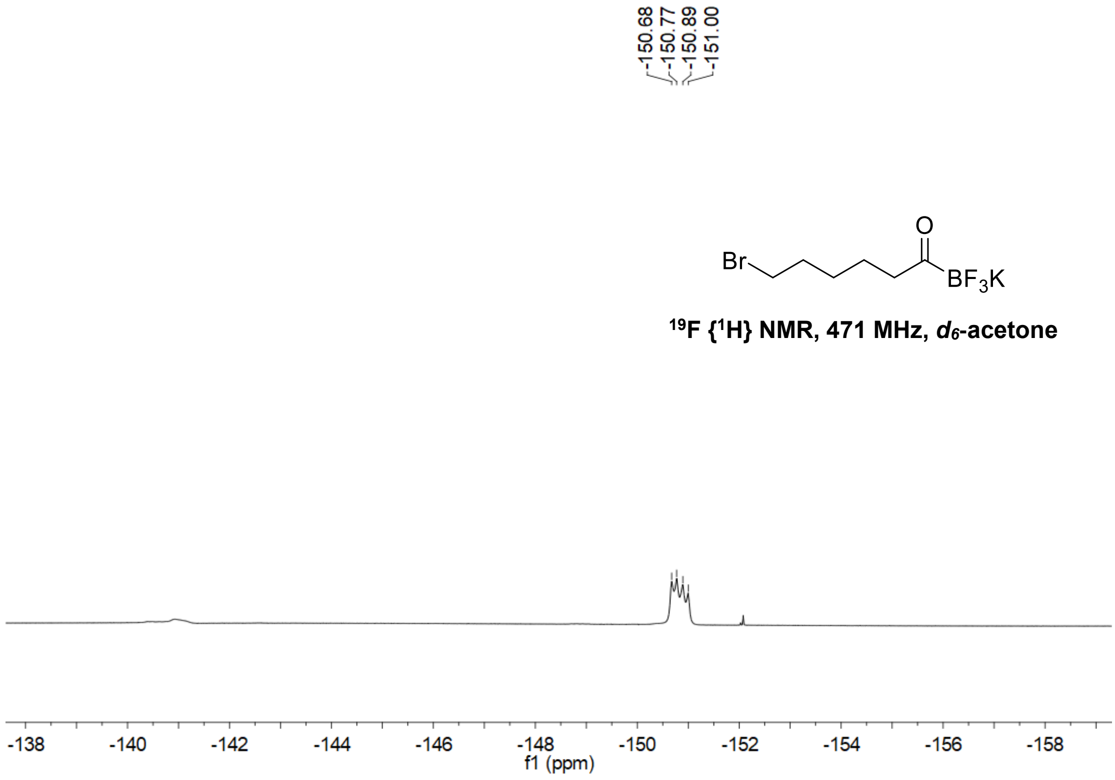
-150.68
-150.77
-150.89
-151.00
Br
O
BF3K
19F {1H} NMR, 471 MHz, d6-acetone
-138 -140 -142 -144 -146 -148 -150 -152 -154 -156 -158
f1 (ppm)

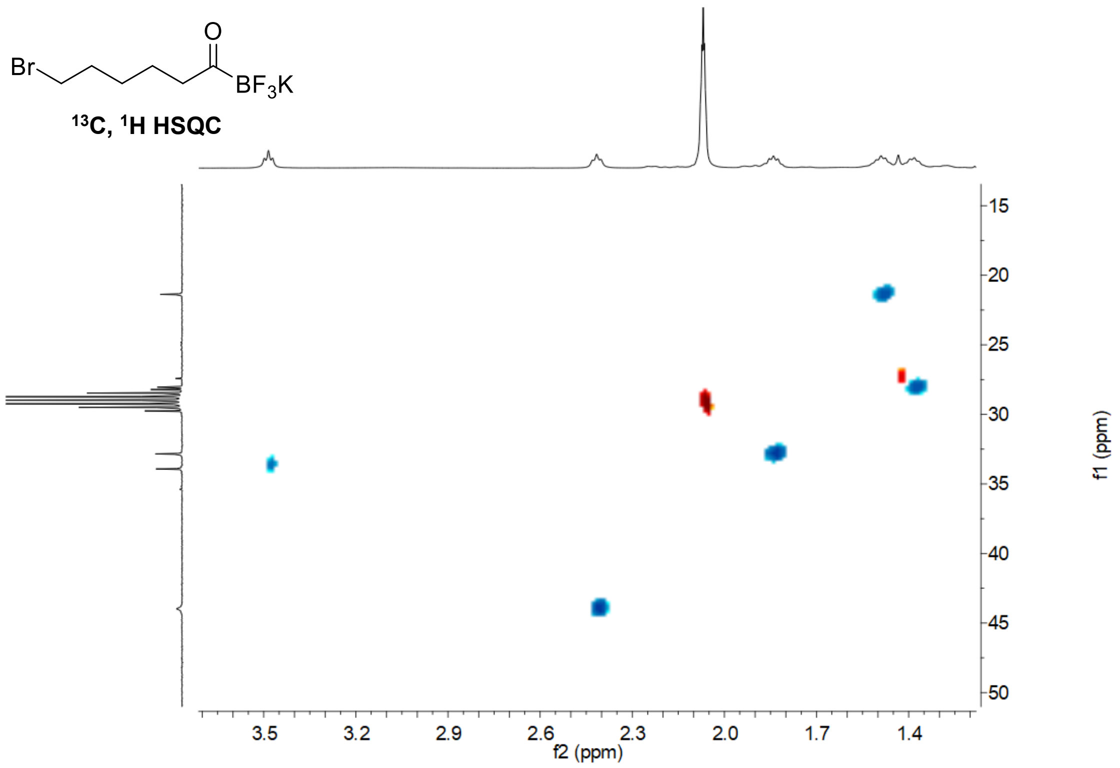
Br
O
BF3K
13C, 1H HSQC
f2 (ppm)
f1 (ppm)

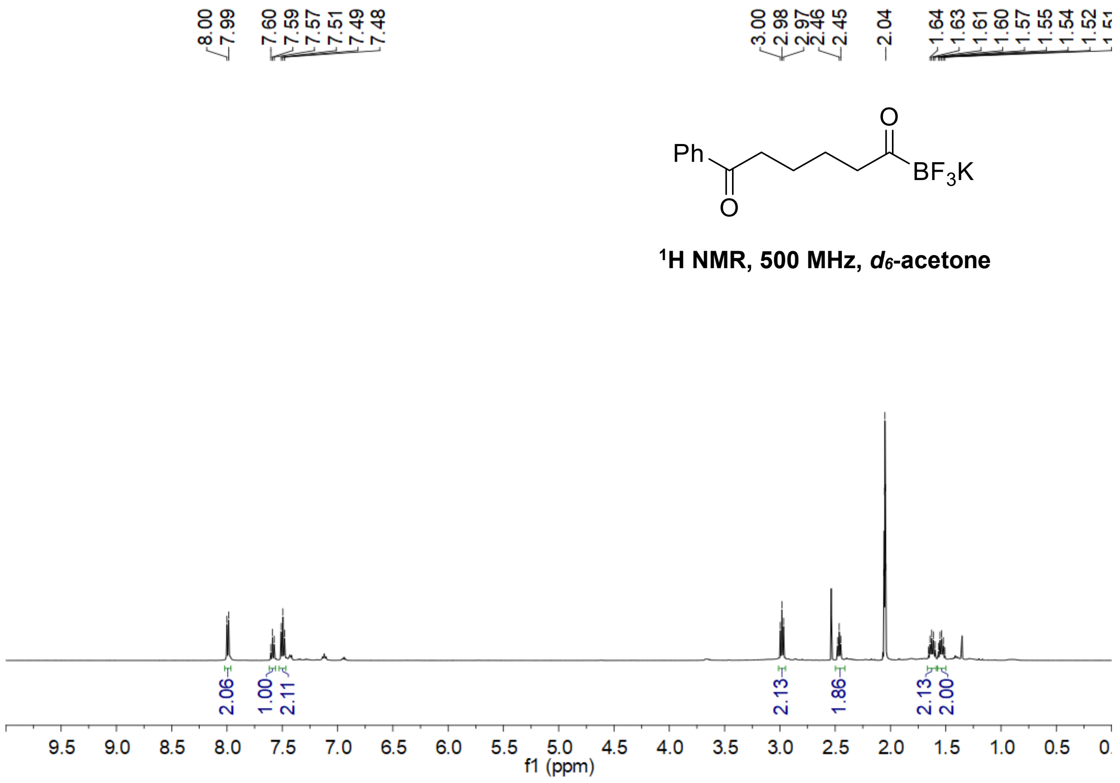
Ph
O
O
BF3K
1H NMR, 500 MHz, d6-acetone
f1 (ppm)

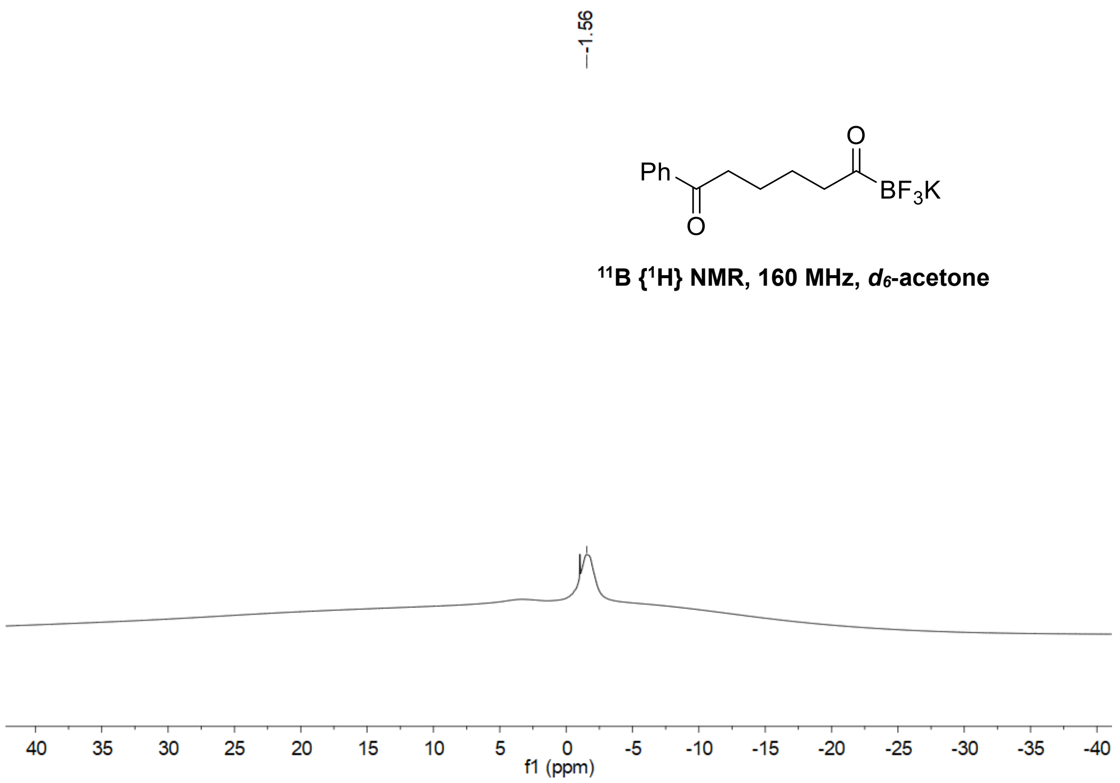
-1.56
Ph
O
O
BF3K
11B {1H} NMR, 160 MHz, d6-acetone
40 35 30 25 20 15 10 5 0 -5 -10 -15 -20 -25 -30 -35 -40
f1 (ppm)

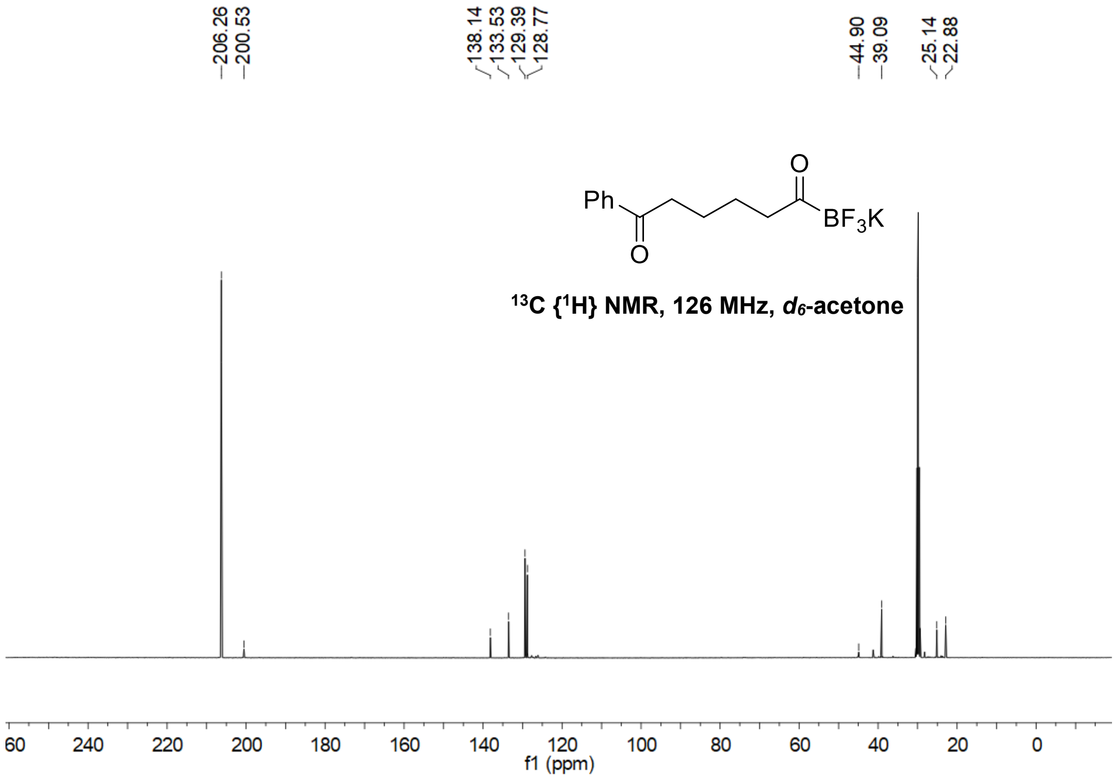
206.26
200.53
138.14
133.53
129.39
128.77
44.90
39.09
25.14
22.88
Ph
O
O
BF3K
13C {1H} NMR, 126 MHz, d6-acetone
60 240 220 200 180 160 140 120 100 80 60 40 20 0
f1 (ppm)

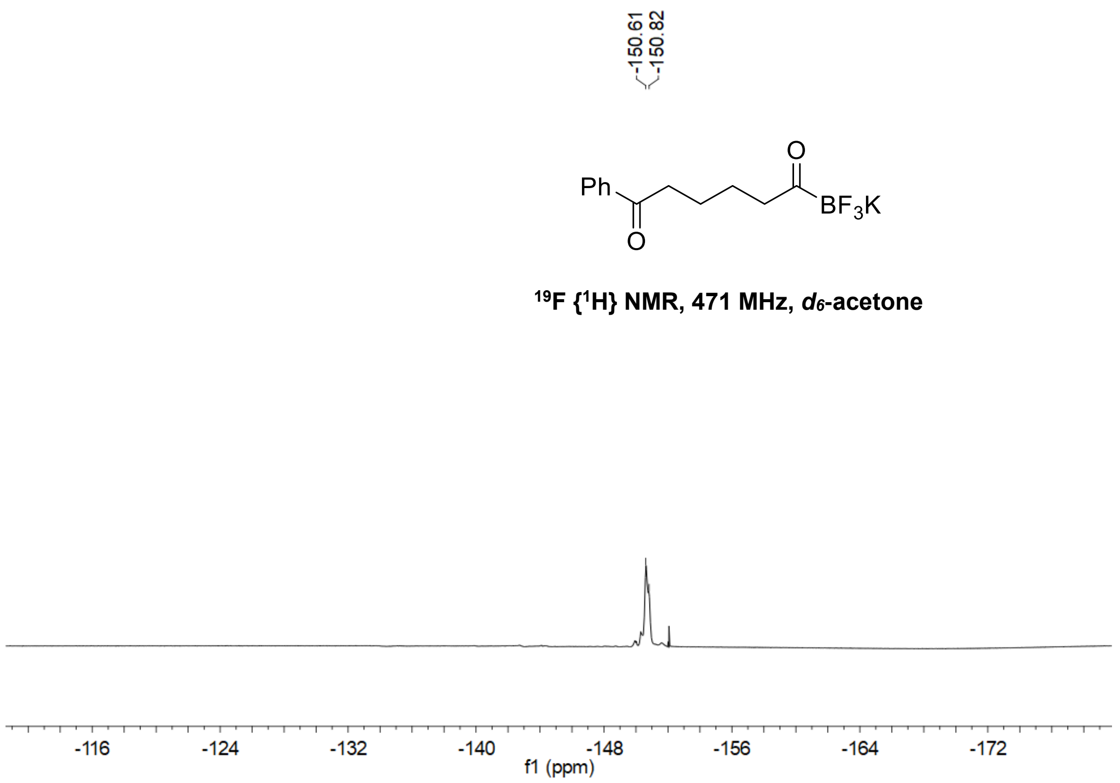
-150.61
-150.82
Ph
O
O
BF3K
19F {1H} NMR, 471 MHz, d6-acetone
-116
-124
-132
-140
-148
-156
-164
-172
f1 (ppm)

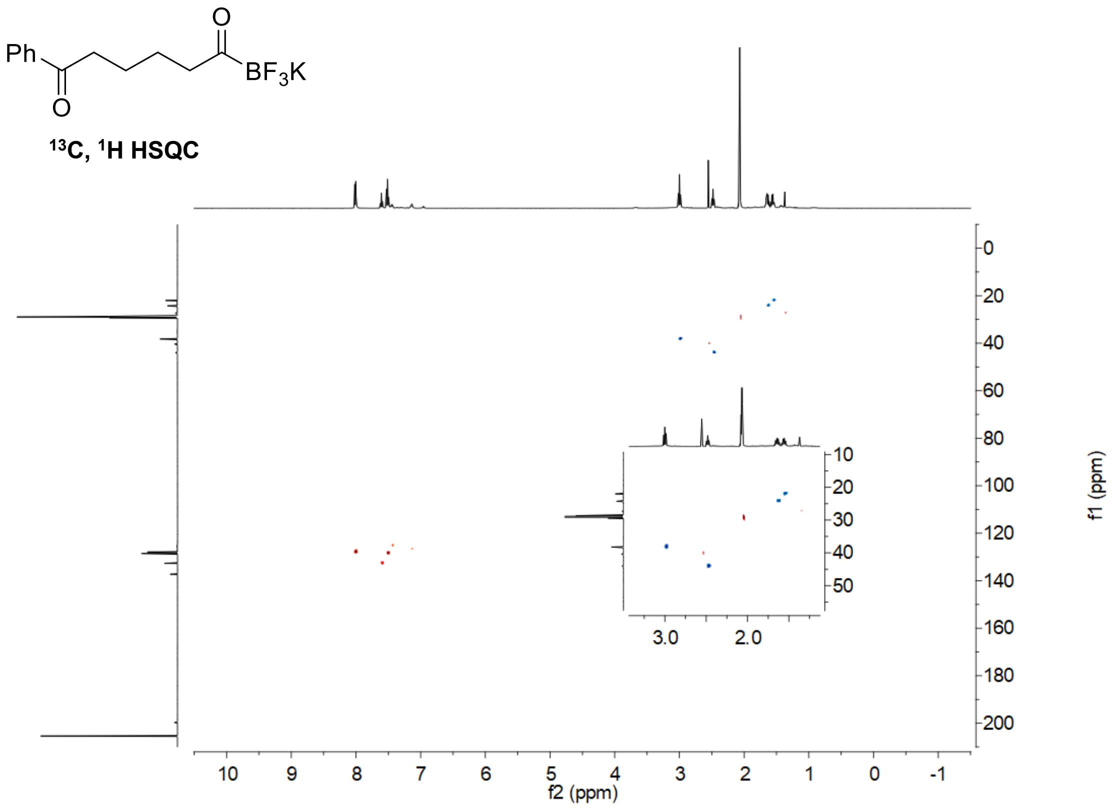
Ph
O
O
BF3K
13C, 1H HSQC
3.0
2.0
f2 (ppm)
f1 (ppm)

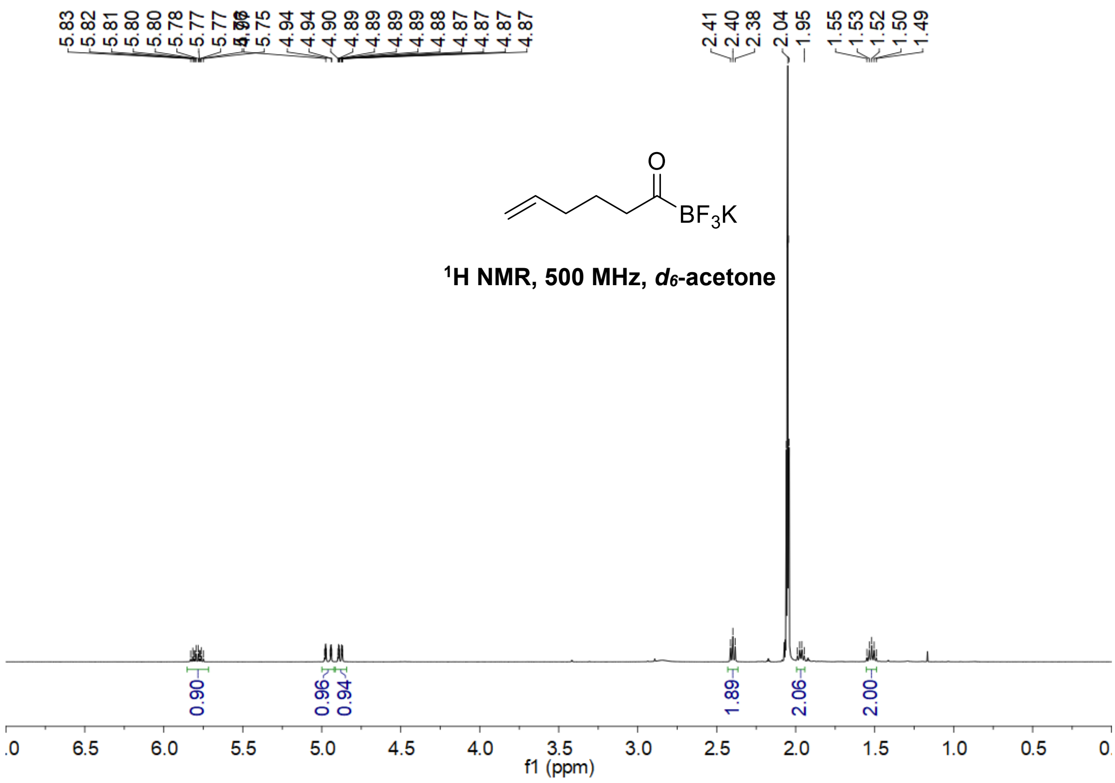
5.83 5.82 5.81 5.80 5.80 5.78 5.77 5.77 5.76 5.75 4.94 4.94 4.90 4.89 4.89 4.89 4.89 4.88 4.87 4.87 4.87 4.87
2.41 2.40 2.38 2.04 1.95 1.55 1.53 1.52 1.50 1.49
O
BF3K
1H NMR, 500 MHz, d6-acetone
0.90 0.96 0.94 1.89 2.06 2.00
6.5 6.0 5.5 5.0 4.5 4.0 3.5 3.0 2.5 2.0 1.5 1.0 0.5
f1 (ppm)

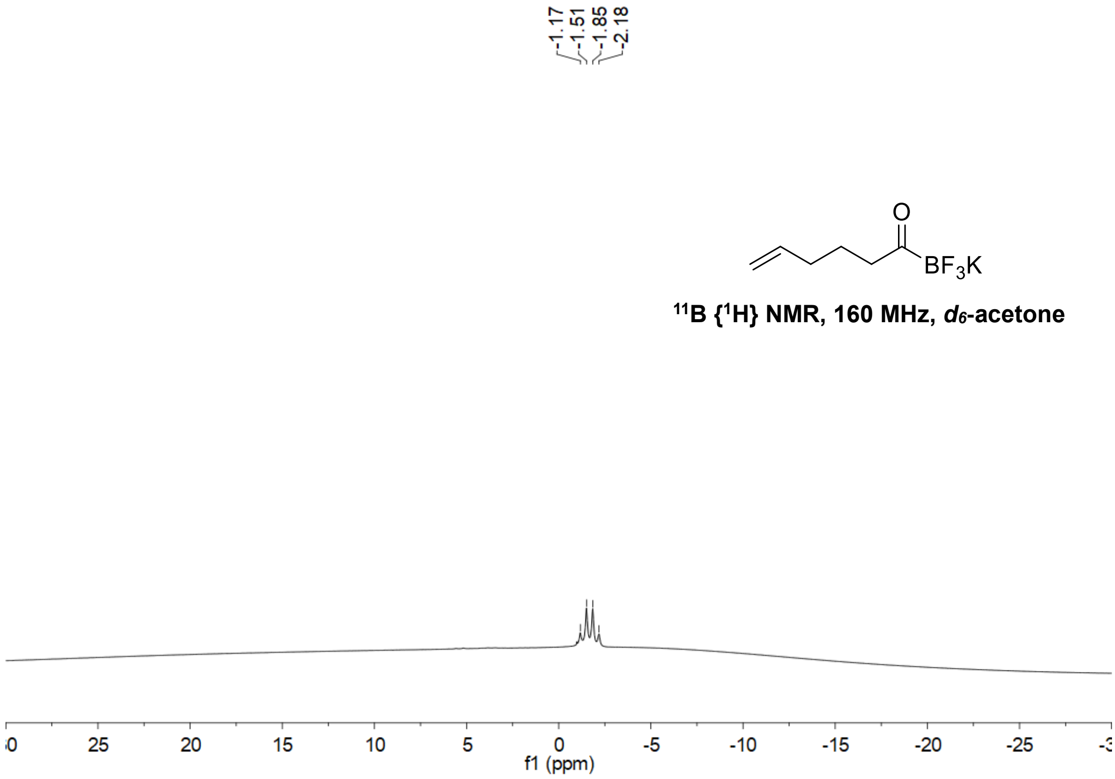
-1.17 -1.51 -1.85 -2.18
O
BF3K
11B {1H} NMR, 160 MHz, d6-acetone
25 20 15 10 5 0 -5 -10 -15 -20 -25
f1 (ppm)

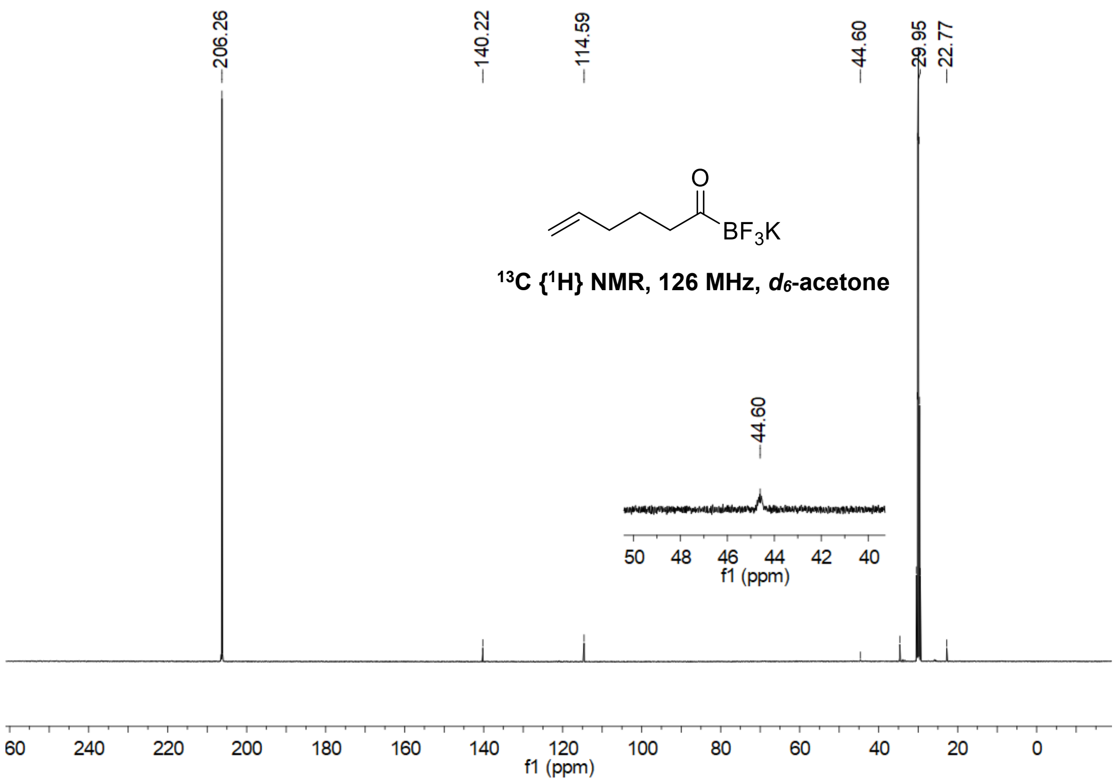

206.26
140.22
114.59
44.60
29.95
22.77
O
BF3K
13C {1H} NMR, 126 MHz, d6-acetone
44.60
50 48 46 44 42 40
f1 (ppm)
60 240 220 200 180 160 140 120 100 80 60 40 20 0
f1 (ppm)

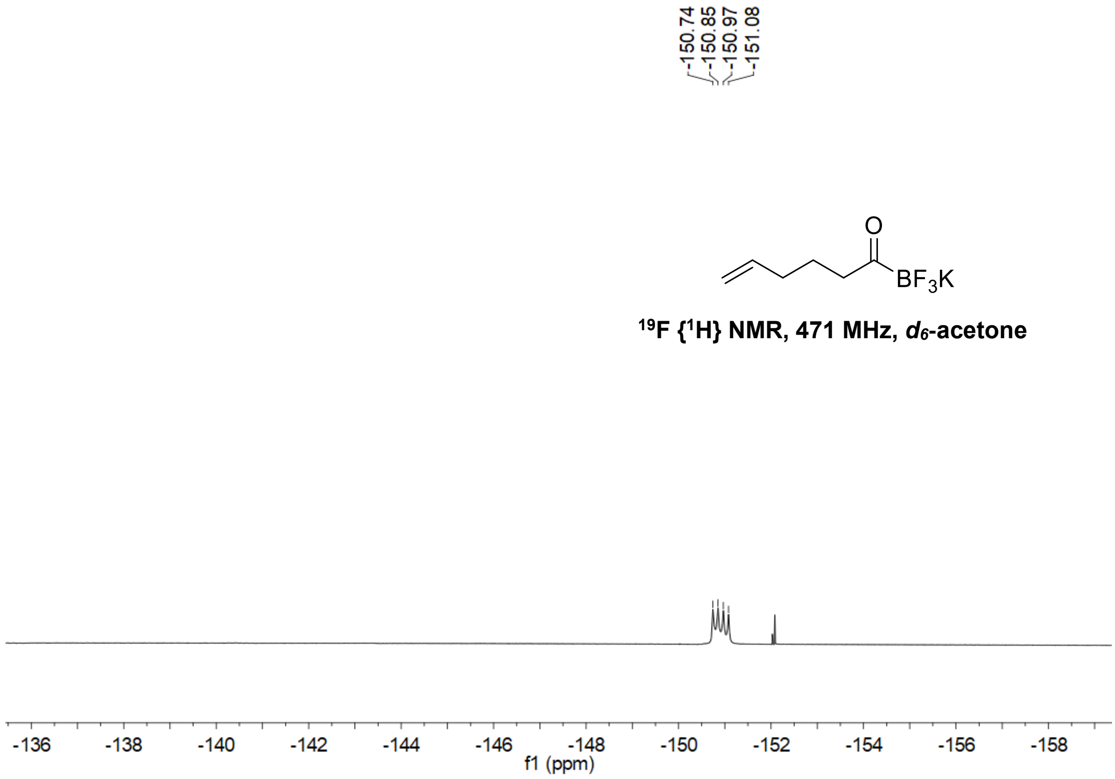

-150.74
-150.85
-150.97
-151.08
O
BF3K
19F {1H} NMR, 471 MHz, d6-acetone
-136 -138 -140 -142 -144 -146 -148 -150 -152 -154 -156 -158
f1 (ppm)

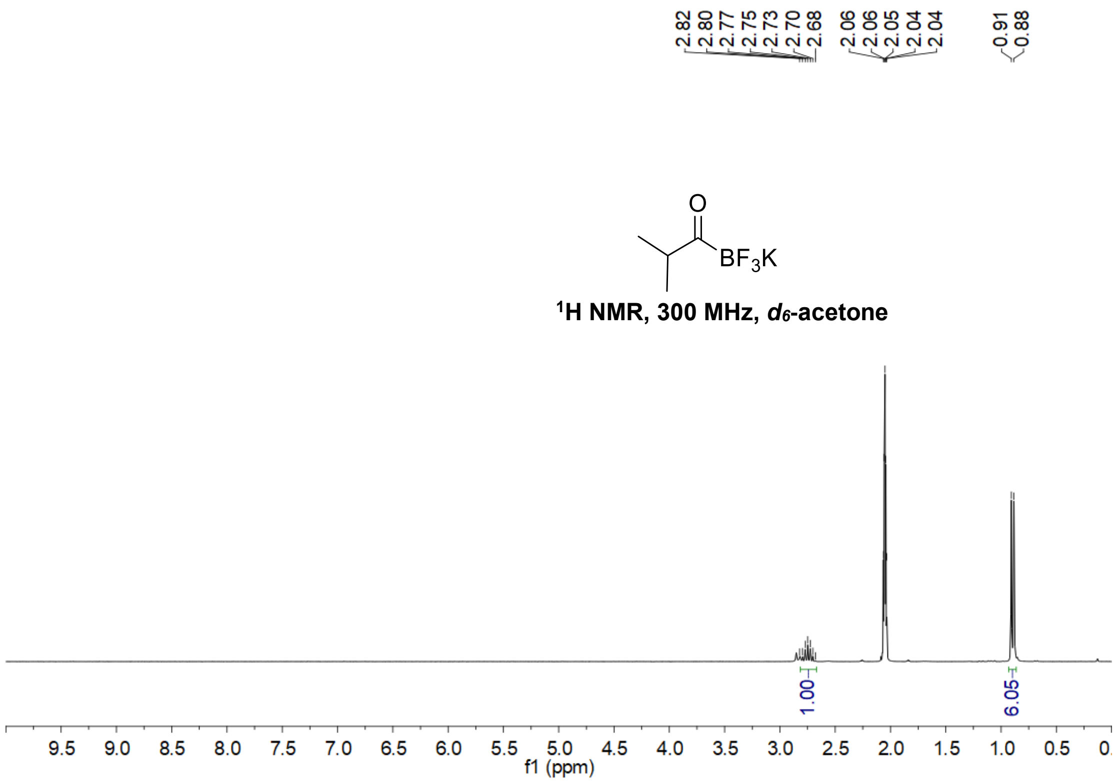

^{1}H NMR, 300 MHz, *d*$_6$-acetone

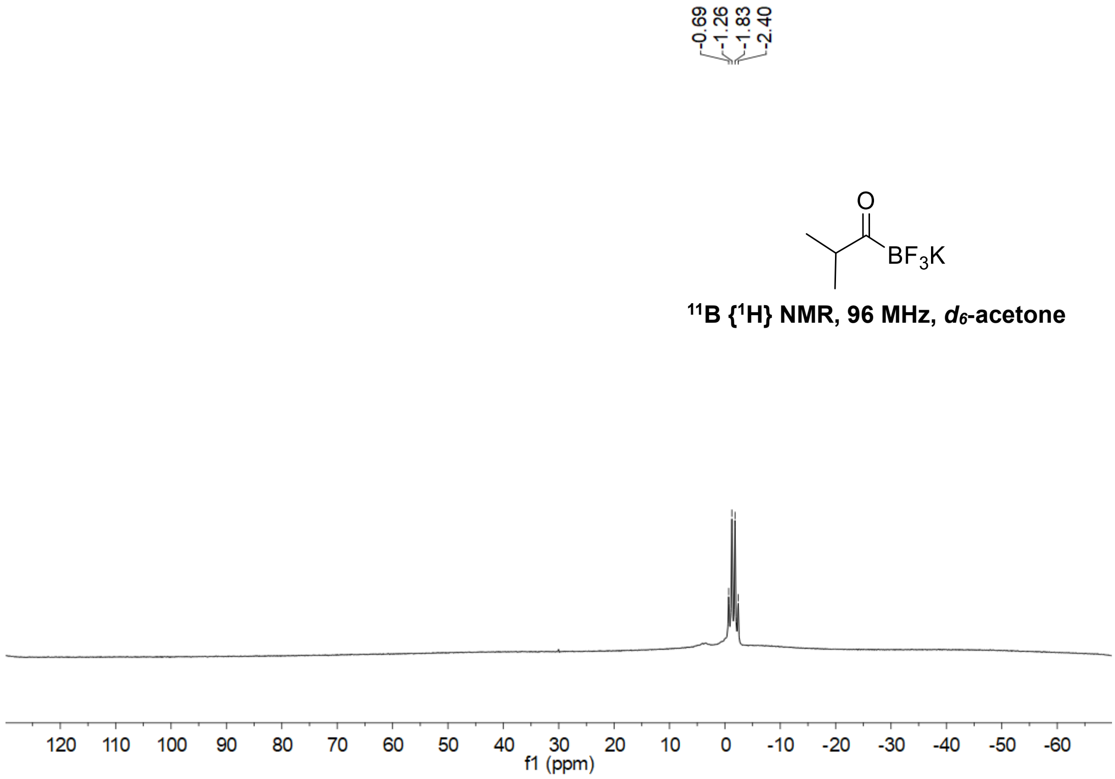

^{11}B {^{1}H} NMR, 96 MHz, *d*$_6$-acetone

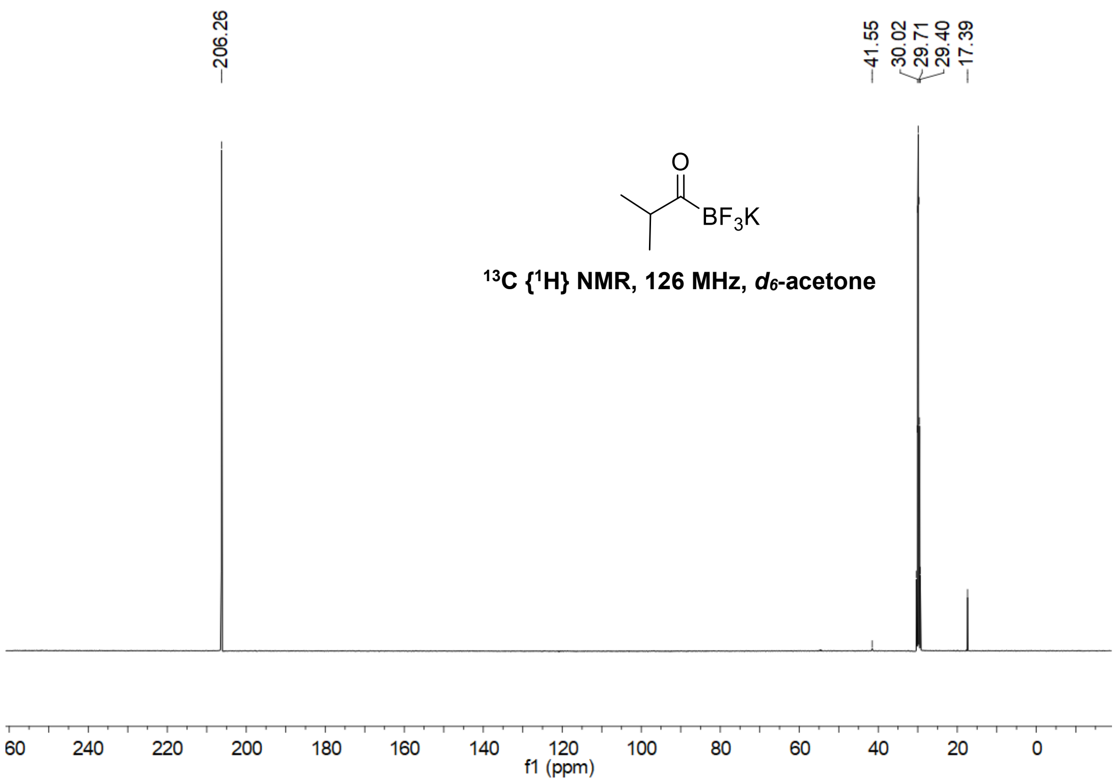

206.26
41.55
30.02
29.71
29.40
17.39
O
BF3K
13C {1H} NMR, 126 MHz, d6-acetone
60
240
220
200
180
160
140
120
100
80
60
40
20
0
f1 (ppm)

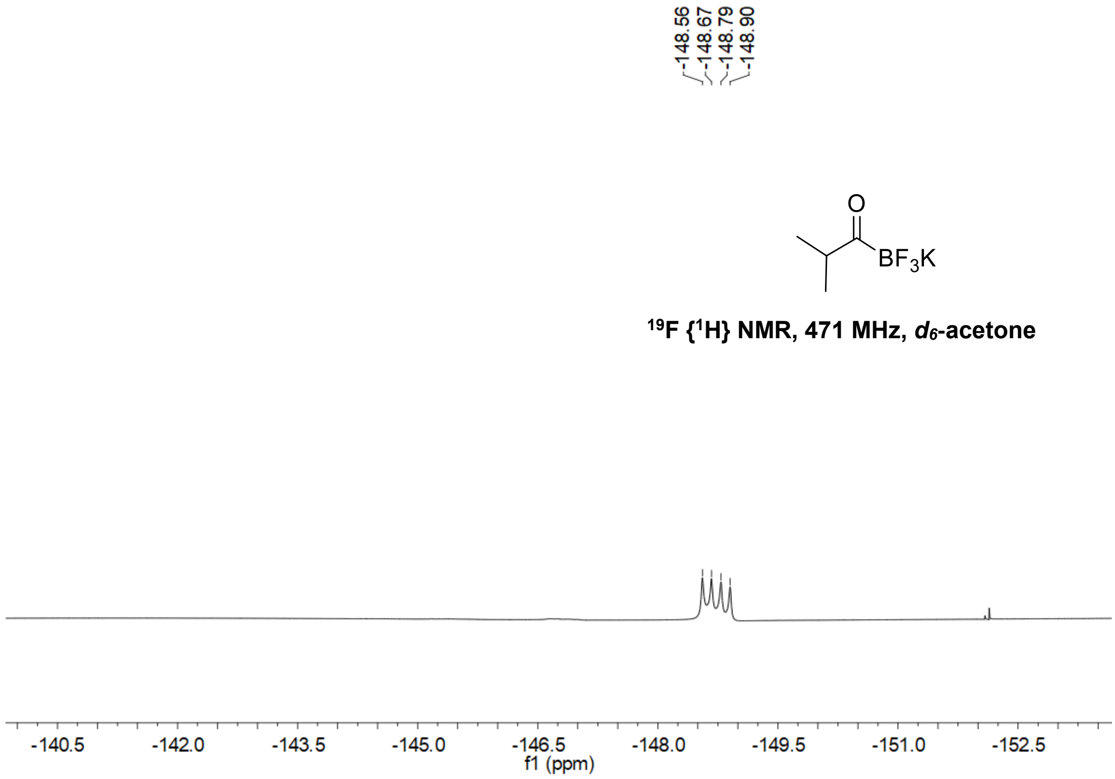

-148.56
-148.67
-148.79
-148.90
O
BF3K
19F {1H} NMR, 471 MHz, d6-acetone
-140.5
-142.0
-143.5
-145.0
-146.5
-148.0
-149.5
-151.0
-152.5
f1 (ppm)

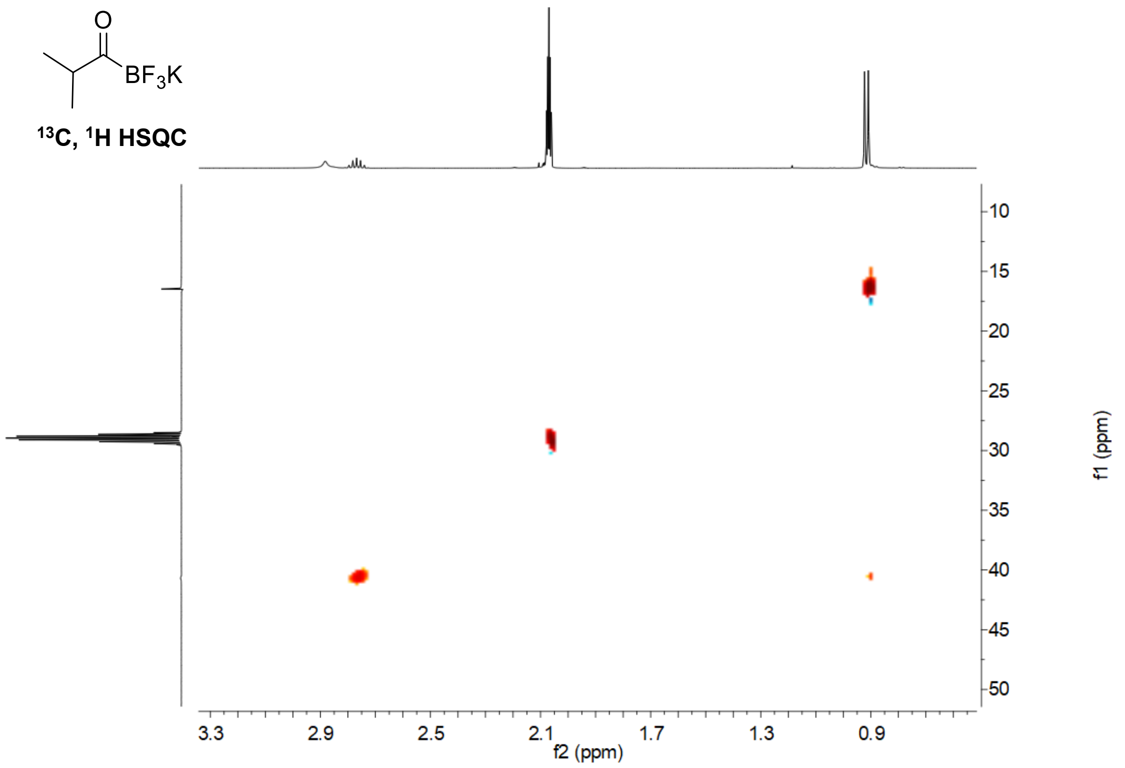
O
BF3K
13C, 1H HSQC
3.3
2.9
2.5
2.1
1.7
1.3
0.9
f2 (ppm)
10
15
20
25
30
35
40
45
50
f1 (ppm)

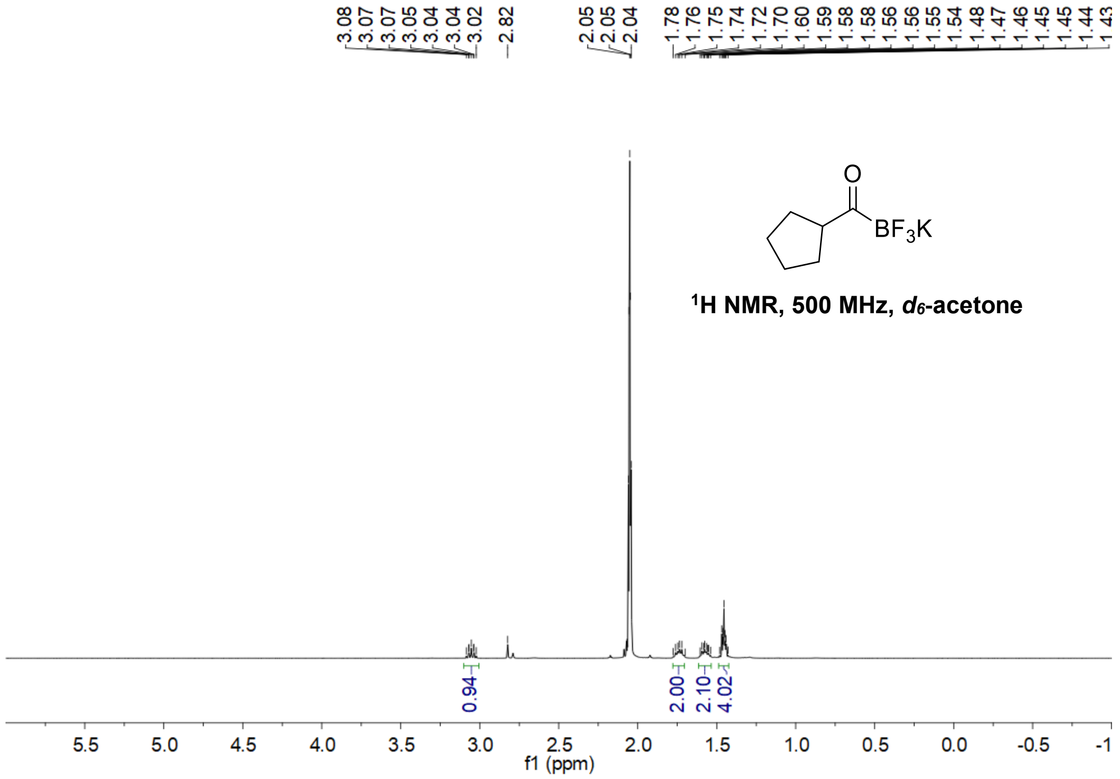
3.08
3.07
3.07
3.05
3.04
3.04
3.02
2.82
2.05
2.05
2.04
1.78
1.76
1.75
1.74
1.72
1.70
1.60
1.59
1.58
1.58
1.56
1.56
1.55
1.54
1.48
1.47
1.46
1.45
1.45
1.44
1.43
O
BF3K
1H NMR, 500 MHz, d6-acetone
0.94
2.00
2.10
4.02
5.5
5.0
4.5
4.0
3.5
3.0
2.5
2.0
1.5
1.0
0.5
0.0
-0.5
-1
f1 (ppm)

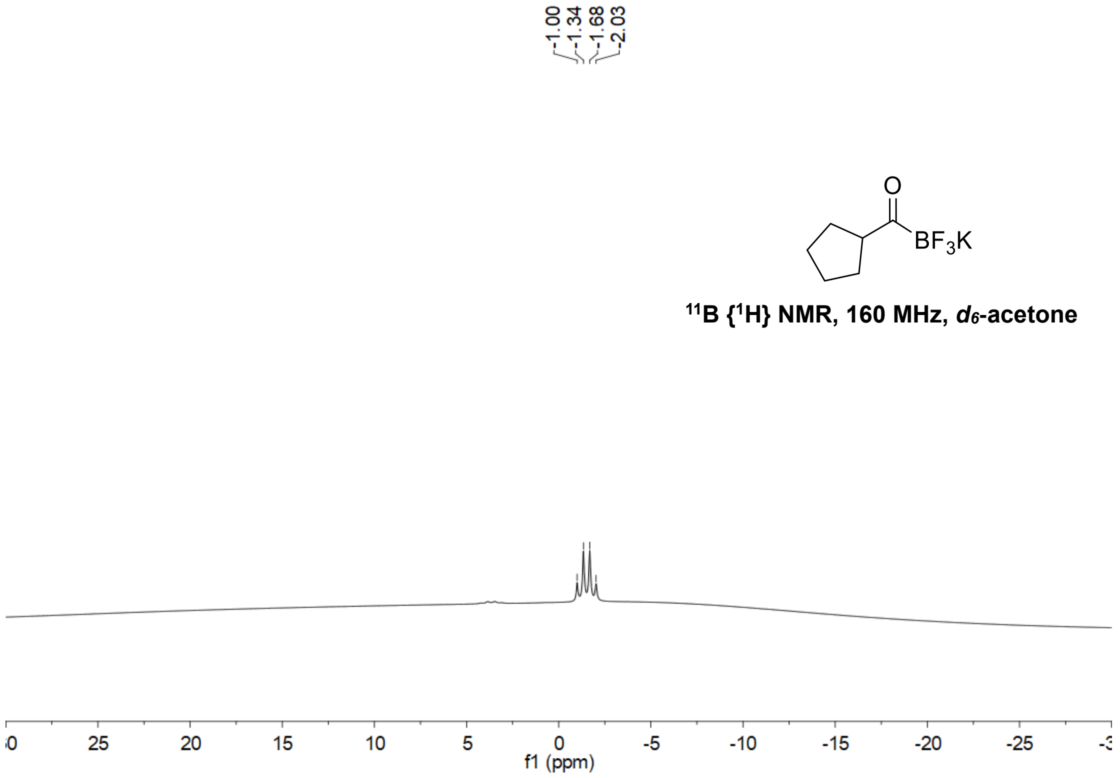
-1.00
-1.34
-1.68
-2.03
O
BF3K
11B {1H} NMR, 160 MHz, d6-acetone
f1 (ppm)

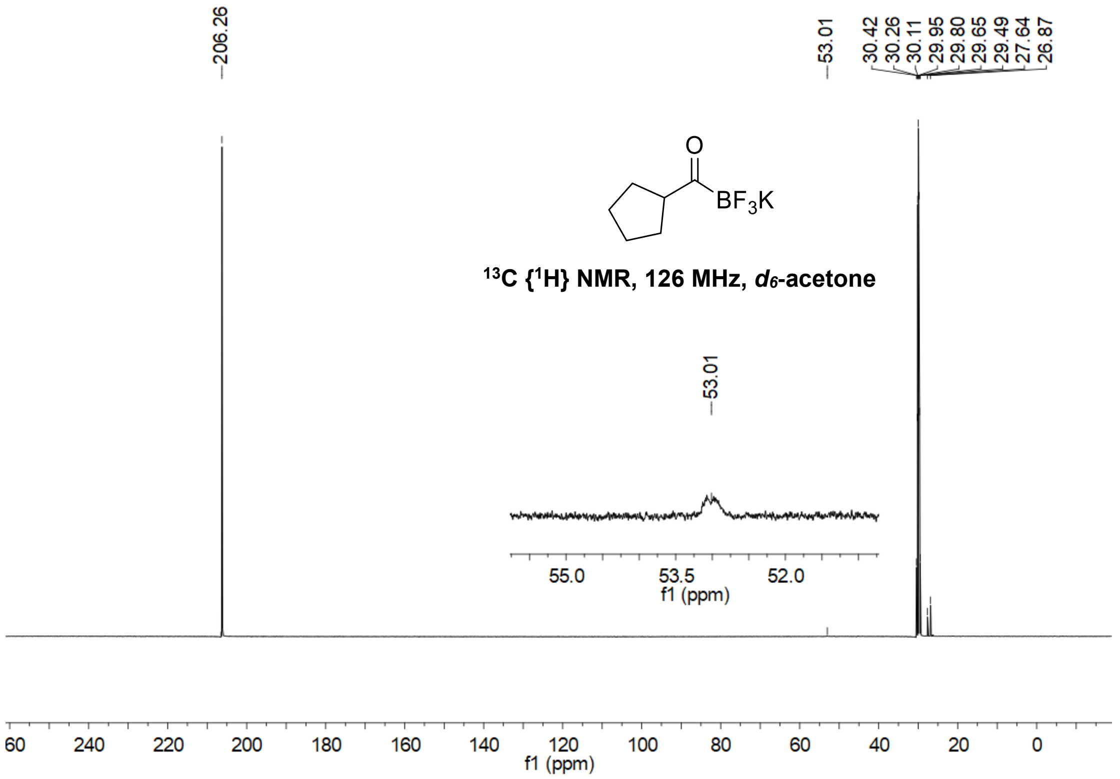
206.26
53.01
30.42
30.26
30.11
29.95
29.80
29.65
29.49
27.64
26.87
O
BF3K
13C {1H} NMR, 126 MHz, d6-acetone
53.01
55.0
53.5
52.0
f1 (ppm)
f1 (ppm)

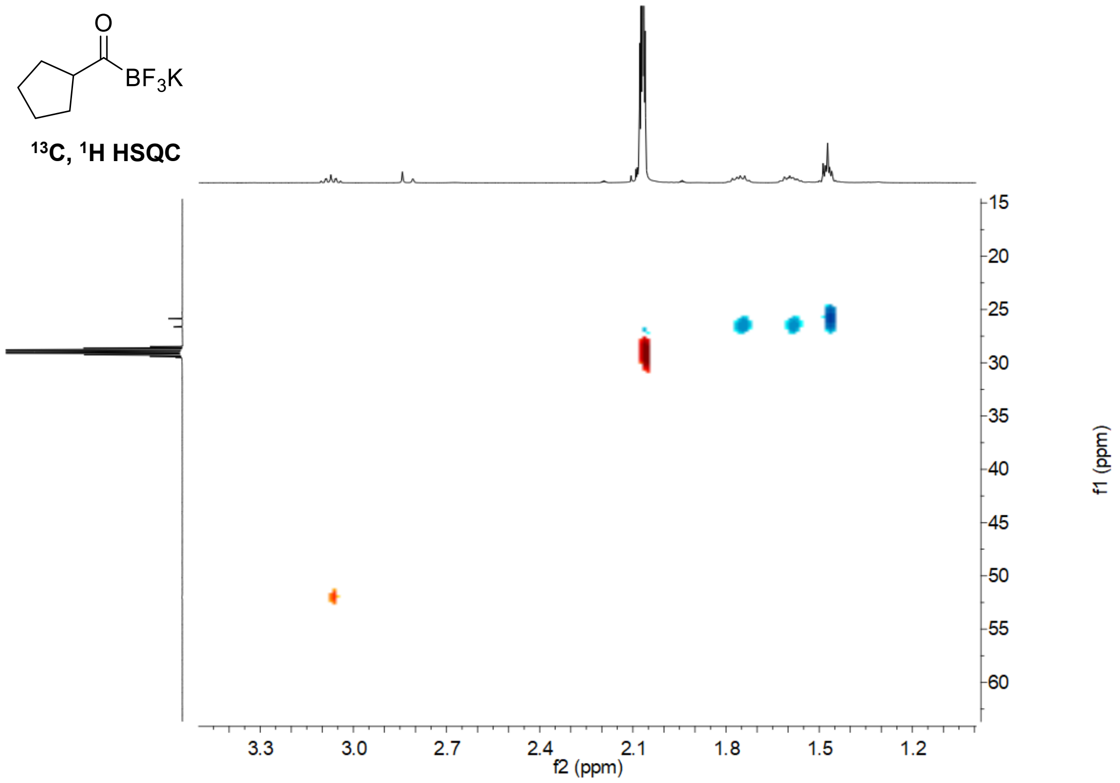
O
BF3K
13C, 1H HSQC
15
20
25
30
35
40
45
50
55
60
f1 (ppm)
3.3
3.0
2.7
2.4
2.1
1.8
1.5
1.2
f2 (ppm)

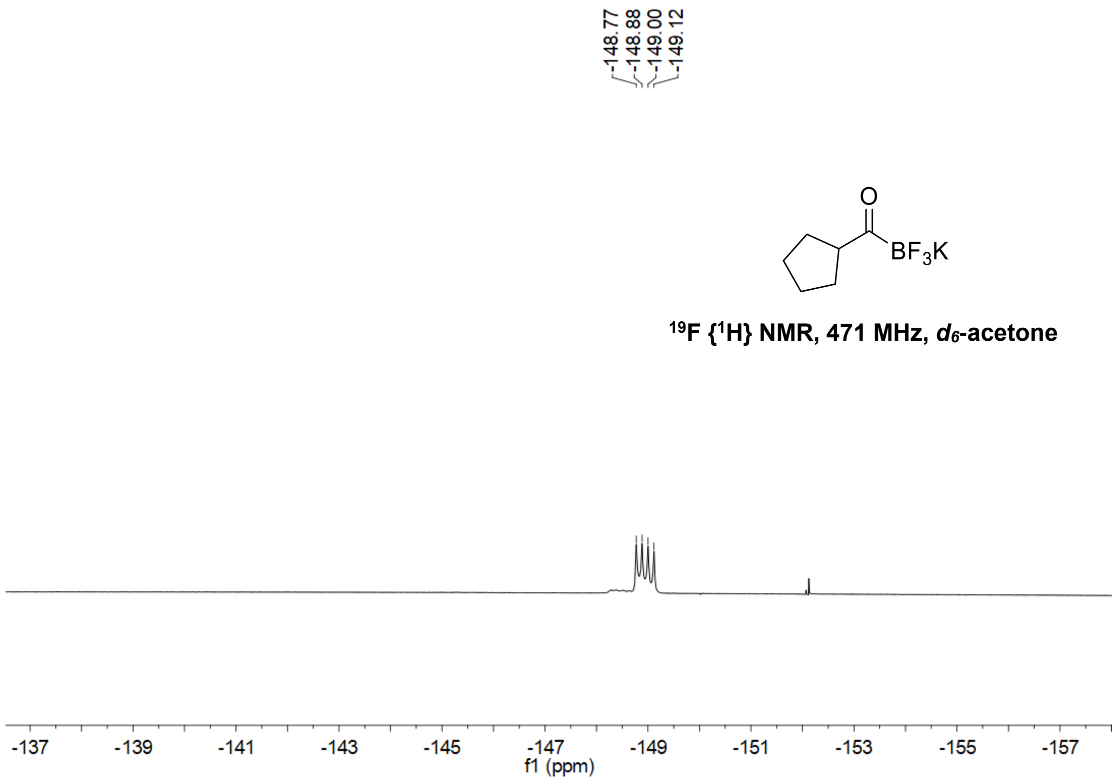
-148.77
-148.88
-149.00
-149.12
O
BF3K
19F {1H} NMR, 471 MHz, d6-acetone
-137
-139
-141
-143
-145
-147
-149
-151
-153
-155
-157
f1 (ppm)

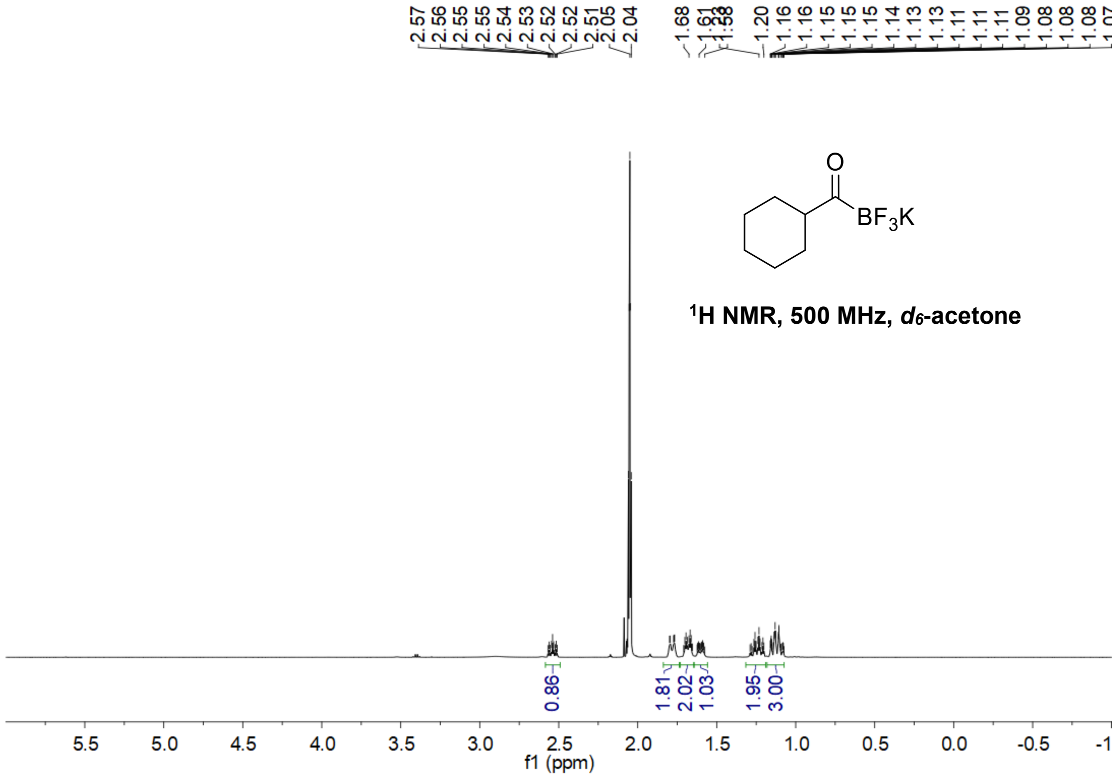
2.57
2.56
2.55
2.55
2.54
2.53
2.52
2.52
2.51
2.05
2.04
1.68
1.61
1.58
1.20
1.16
1.16
1.15
1.15
1.15
1.14
1.13
1.13
1.11
1.11
1.11
1.09
1.08
1.08
1.08
O
BF_3K
^1H NMR, 500 MHz, d_6-acetone
0.86
1.81
2.02
1.03
1.95
3.00
5.5
5.0
4.5
4.0
3.5
3.0
2.5
2.0
1.5
1.0
0.5
0.0
-0.5
f1 (ppm)

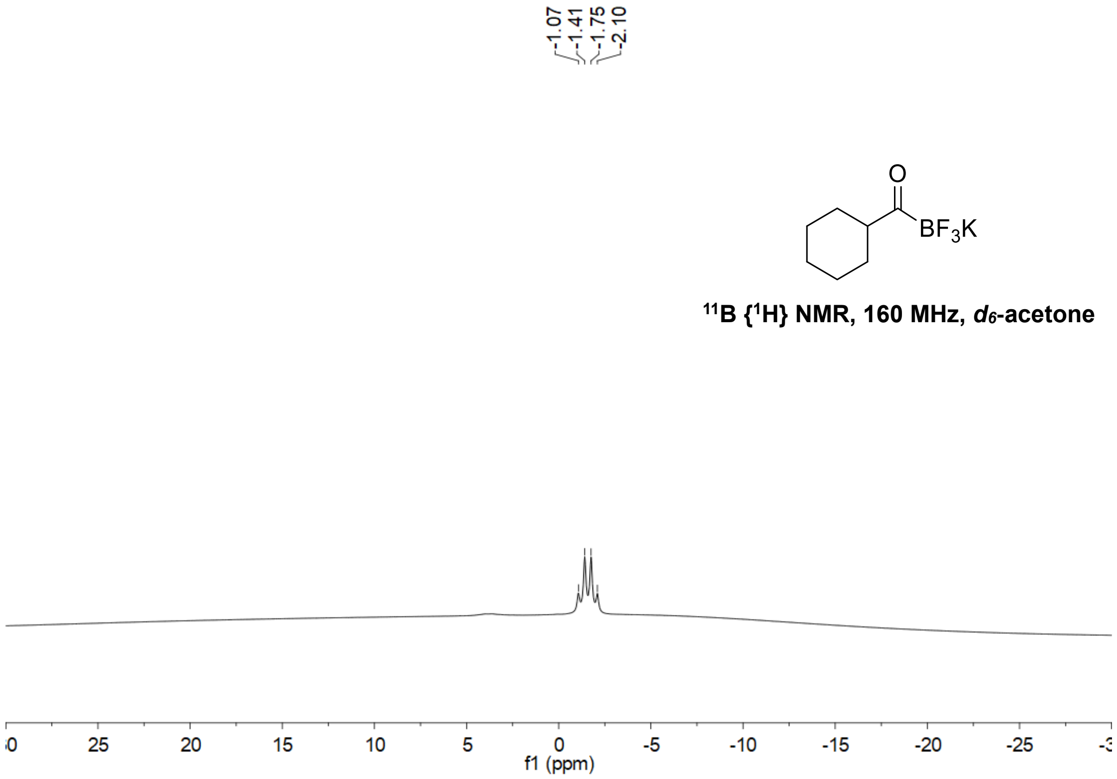
-1.07
-1.41
-1.75
-2.10
O
BF_3K
^{11}B {^1H} NMR, 160 MHz, d_6-acetone
25
20
15
10
5
0
-5
-10
-15
-20
-25
f1 (ppm)

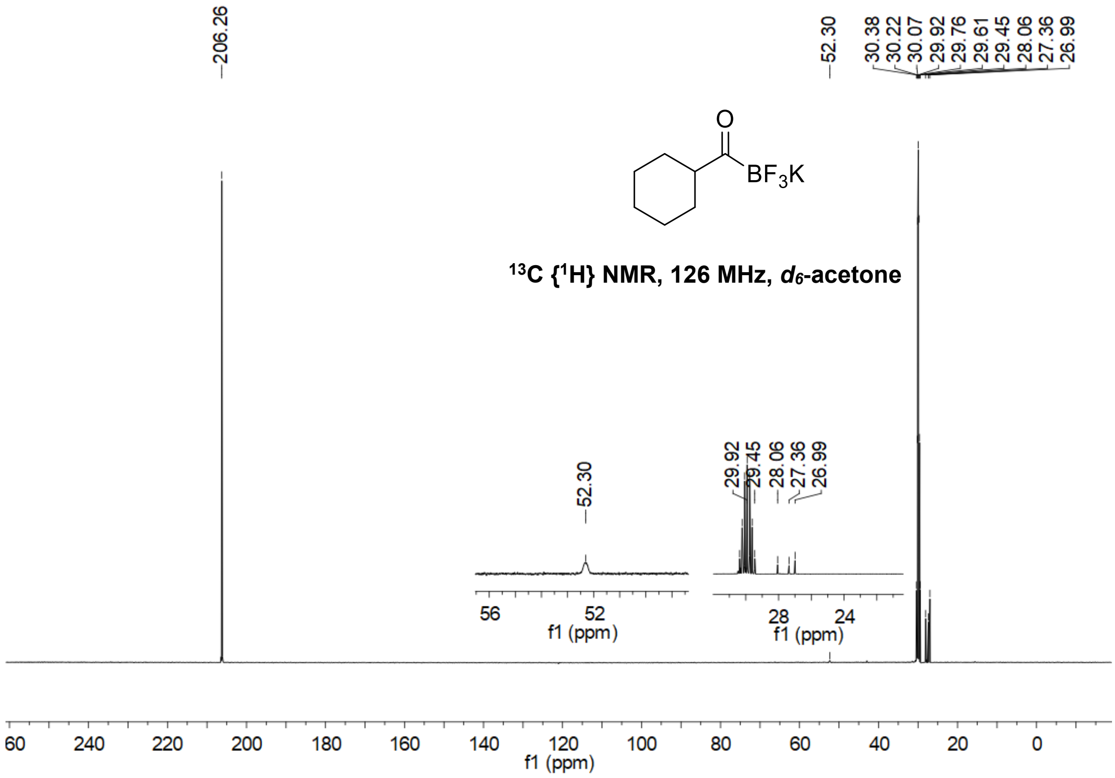
206.26
52.30
30.38
30.22
30.07
29.92
29.76
29.61
29.45
28.06
27.36
26.99
O
BF3K
13C {1H} NMR, 126 MHz, d6-acetone
52.30
29.92
29.45
28.06
27.36
26.99
56
52
f1 (ppm)
28
24
f1 (ppm)
60
240
220
200
180
160
140
120
100
80
60
40
20
0
f1 (ppm)

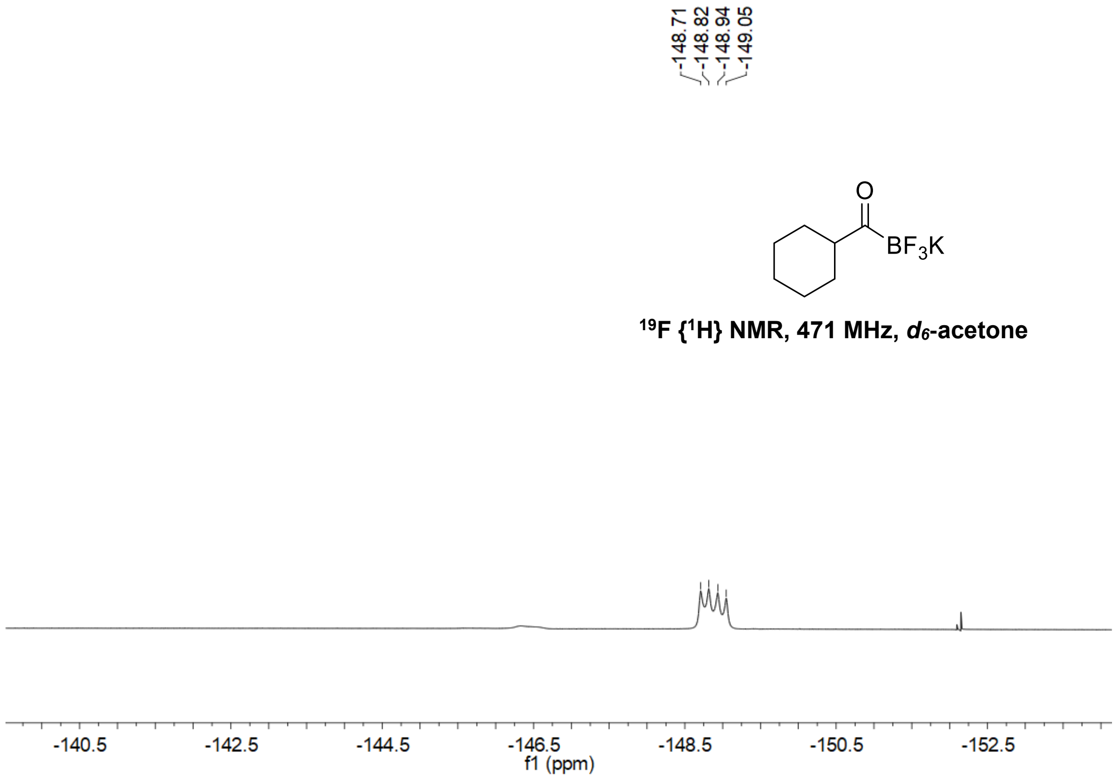
-148.71
-148.82
-148.94
-149.05
O
BF3K
19F {1H} NMR, 471 MHz, d6-acetone
-140.5
-142.5
-144.5
-146.5
-148.5
-150.5
-152.5
f1 (ppm)

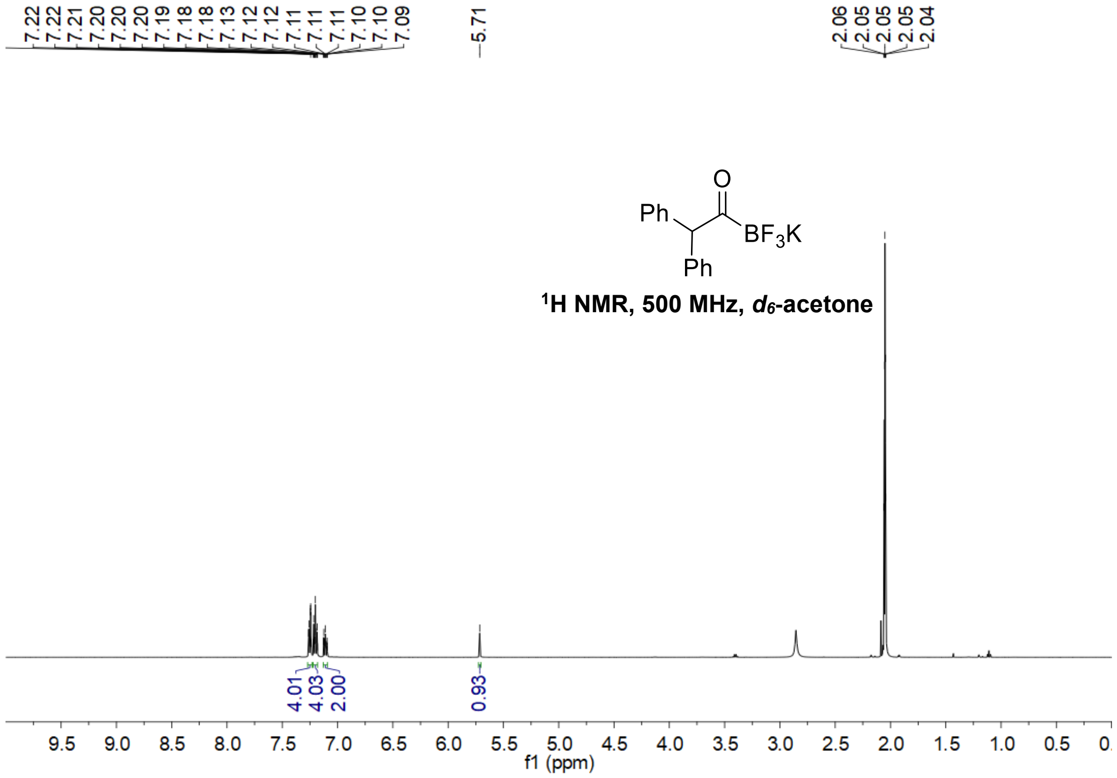
7.22 7.22 7.21 7.20 7.20 7.20 7.19 7.18 7.18 7.13 7.12 7.12 7.11 7.11 7.11 7.10 7.10 7.09
5.71
2.06 2.05 2.05 2.05 2.04
O
Ph
BF3K
Ph
1H NMR, 500 MHz, d6-acetone
4.01
4.03
2.00
0.93
9.5 9.0 8.5 8.0 7.5 7.0 6.5 6.0 5.5 5.0 4.5 4.0 3.5 3.0 2.5 2.0 1.5 1.0 0.5
f1 (ppm)

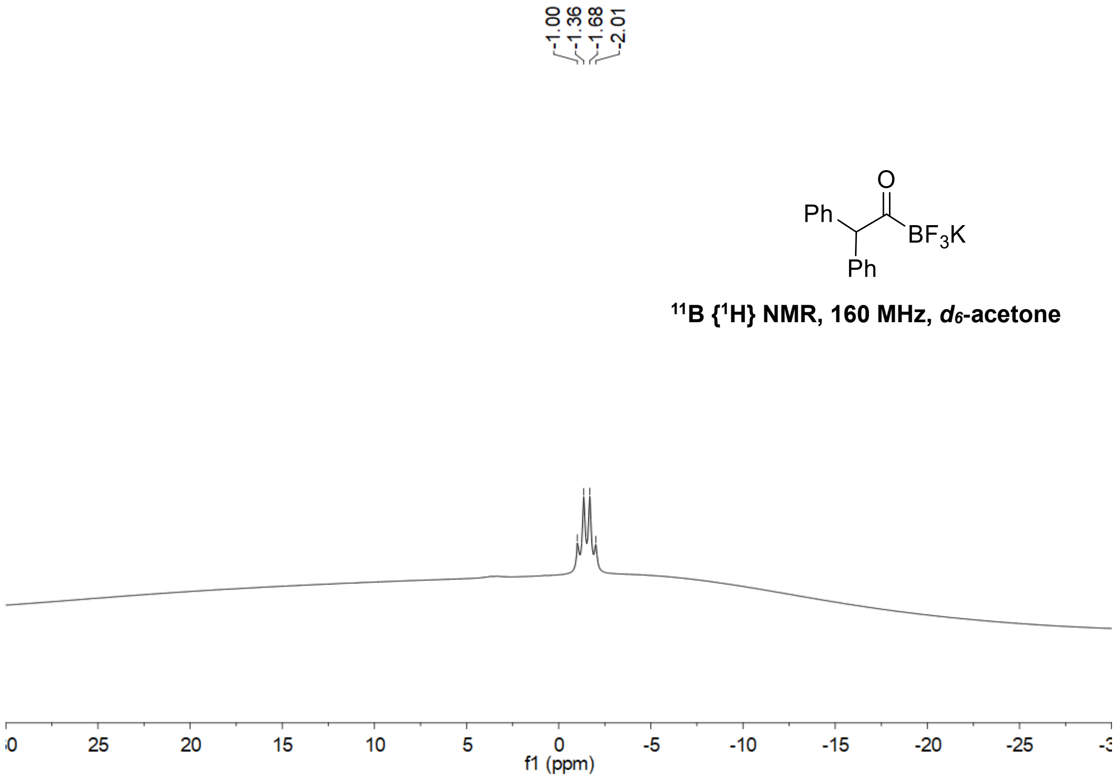
-1.00 -1.36 -1.68 -2.01
O
Ph
BF3K
Ph
11B {1H} NMR, 160 MHz, d6-acetone
25 20 15 10 5 0 -5 -10 -15 -20 -25
f1 (ppm)

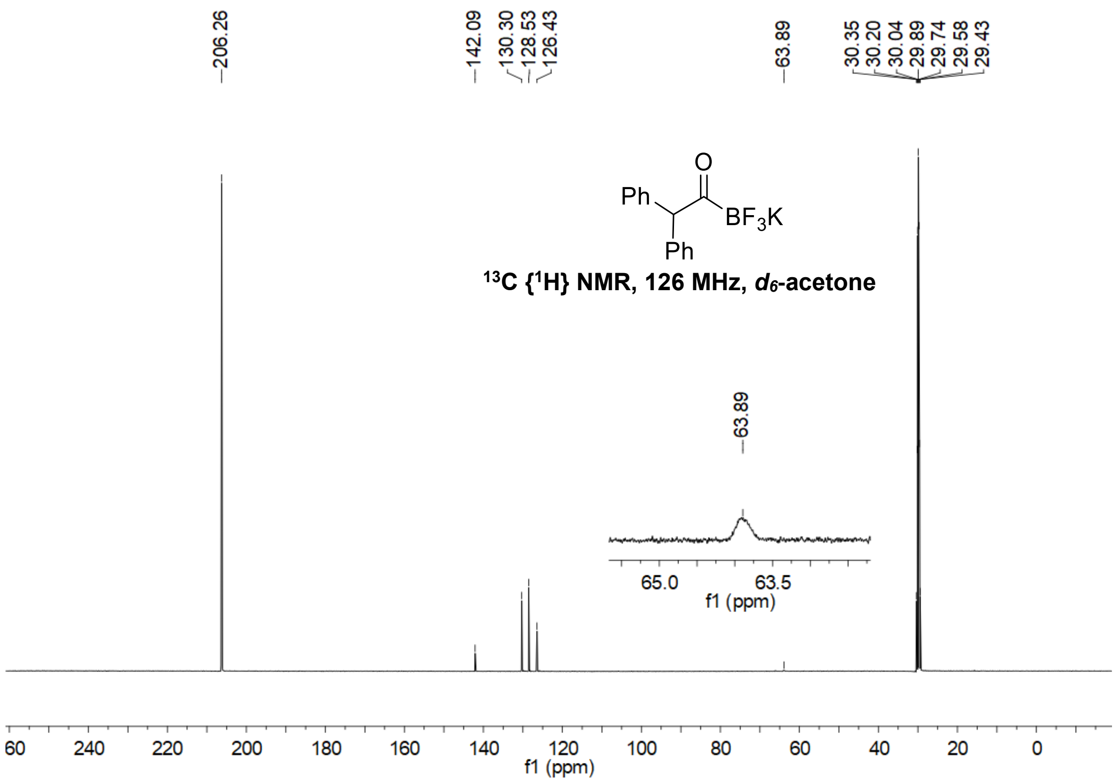
206.26
142.09
130.30
128.53
126.43
63.89
30.35
30.20
30.04
29.89
29.74
29.58
29.43
O
Ph
BF3K
Ph
13C {1H} NMR, 126 MHz, d6-acetone
63.89
65.0
63.5
f1 (ppm)
60
240
220
200
180
160
140
120
100
80
60
40
20
0
f1 (ppm)

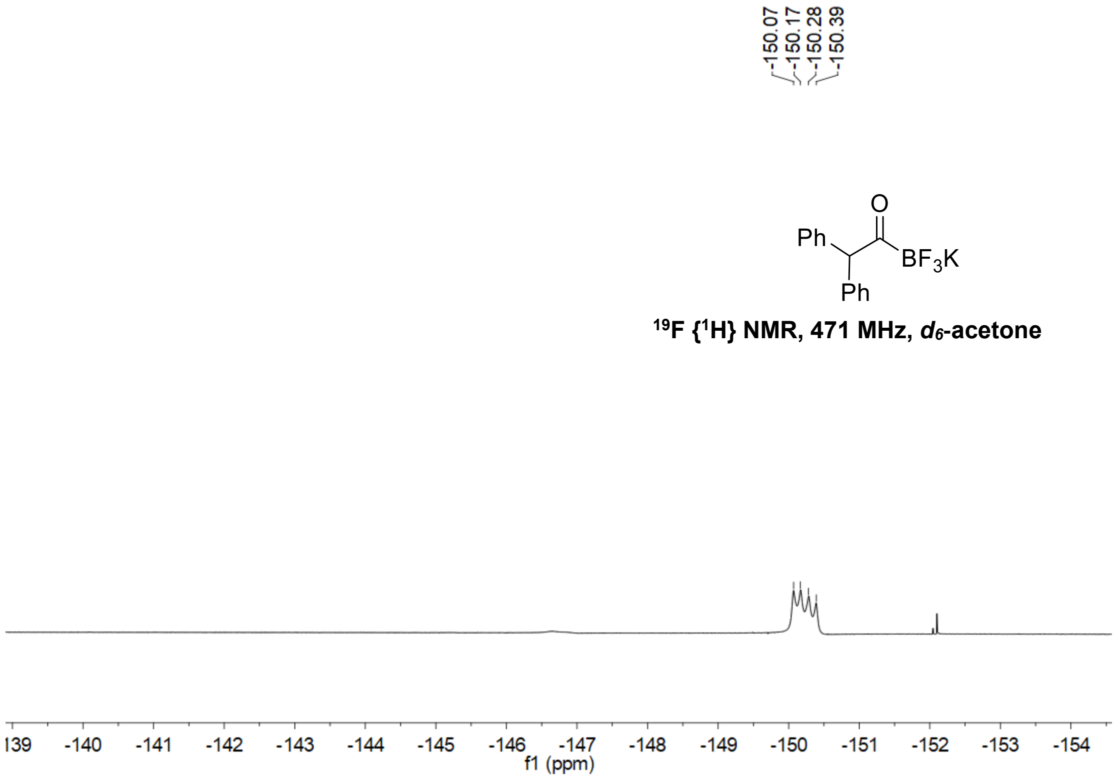
-150.07
-150.17
-150.28
-150.39
O
Ph
BF3K
Ph
19F {1H} NMR, 471 MHz, d6-acetone
139
-140
-141
-142
-143
-144
-145
-146
-147
-148
-149
-150
-151
-152
-153
-154
f1 (ppm)

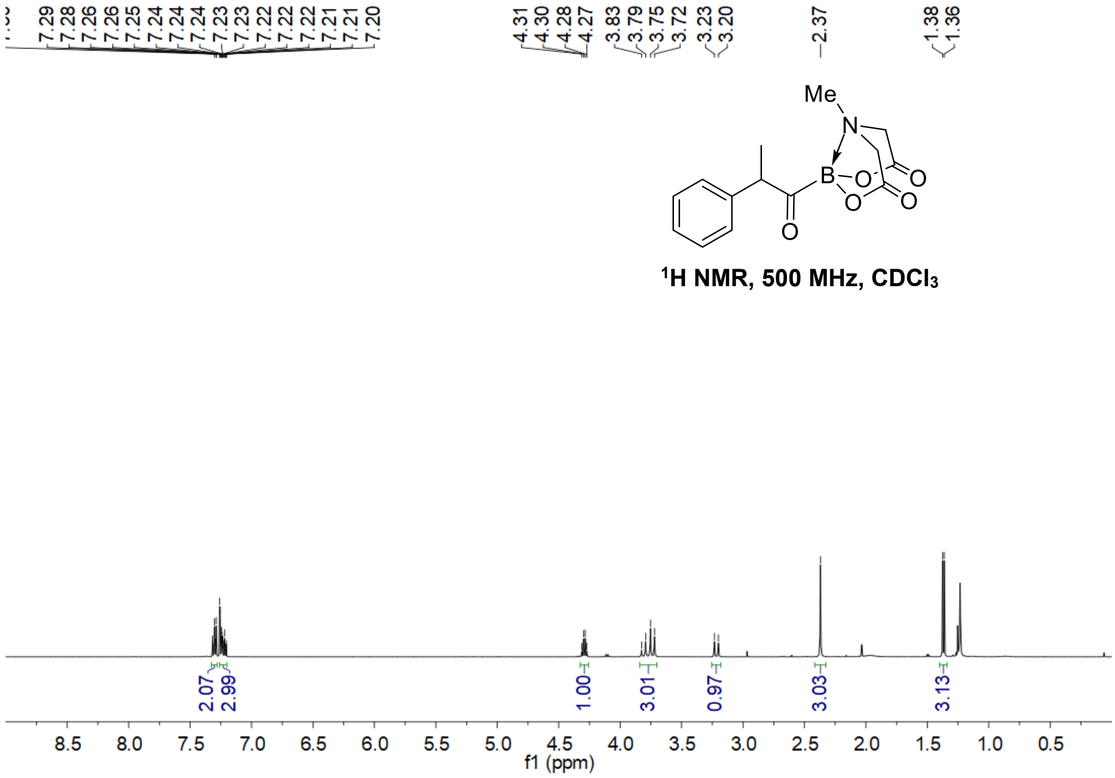
1H NMR, 500 MHz, CDCl3
Me
N
B
O
f1 (ppm)

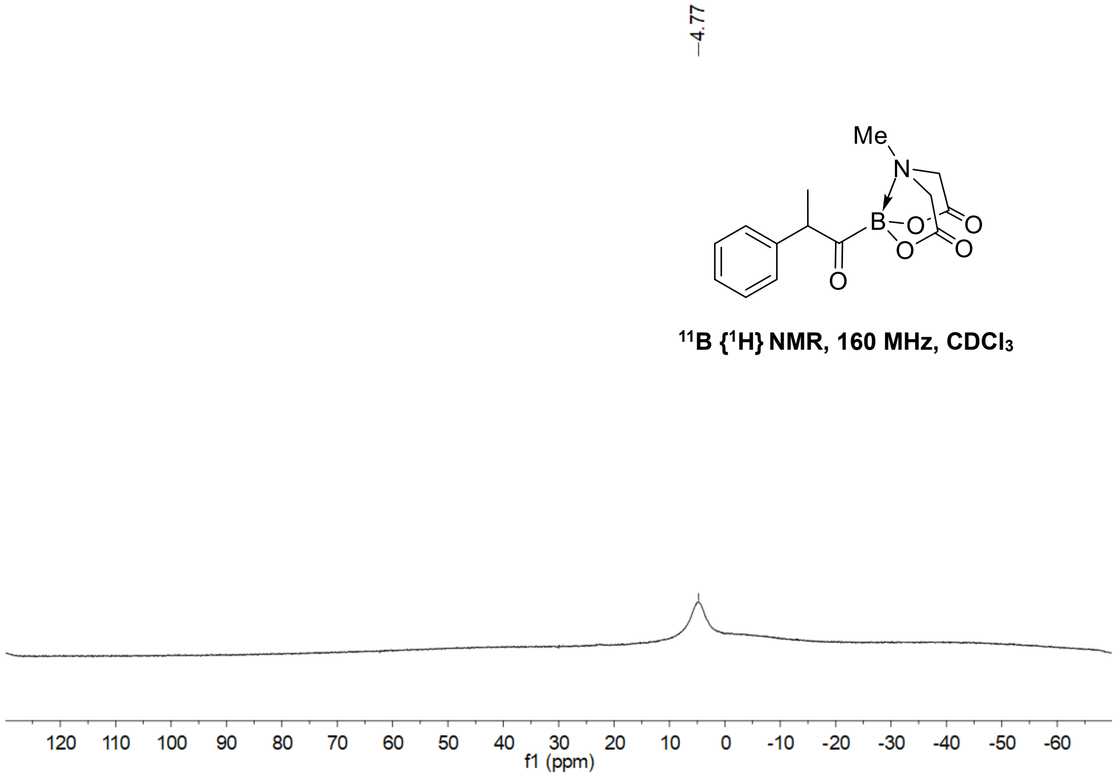
11B {1H} NMR, 160 MHz, CDCl3
Me
N
B
O
-4.77
f1 (ppm)

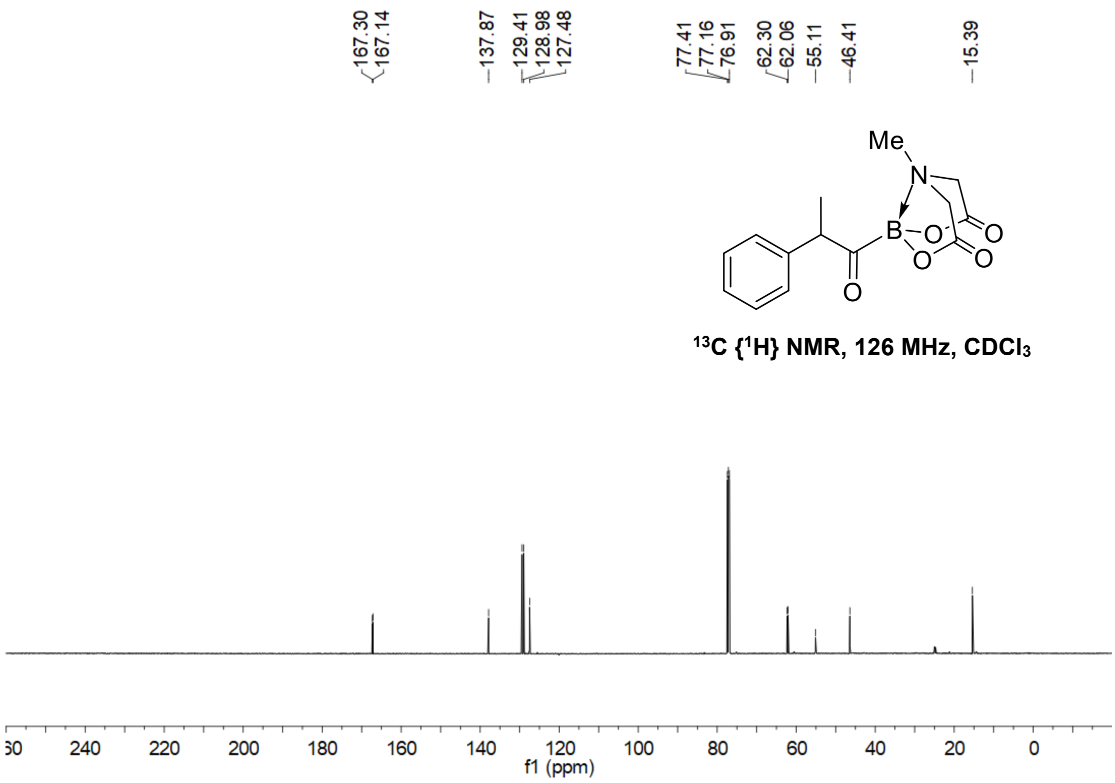
167.30
167.14
137.87
129.41
128.98
127.48
77.41
77.16
76.91
62.30
62.06
55.11
46.41
15.39
Me
N
B
O
O
O
O
O
13C {1H} NMR, 126 MHz, CDCl3
240
220
200
180
160
140
120
100
80
60
40
20
0
f1 (ppm)

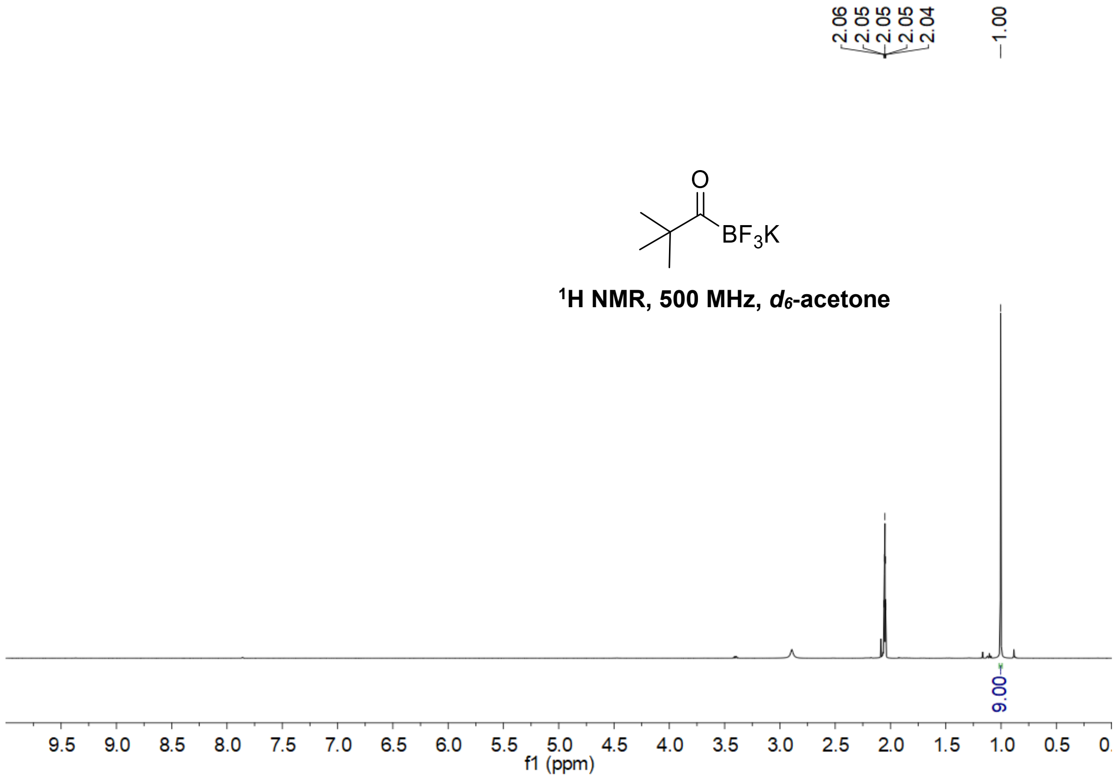
2.06
2.05
2.05
2.05
2.04
1.00
O
BF3K
1H NMR, 500 MHz, d6-acetone
9.00
9.5
9.0
8.5
8.0
7.5
7.0
6.5
6.0
5.5
5.0
4.5
4.0
3.5
3.0
2.5
2.0
1.5
1.0
0.5
f1 (ppm)

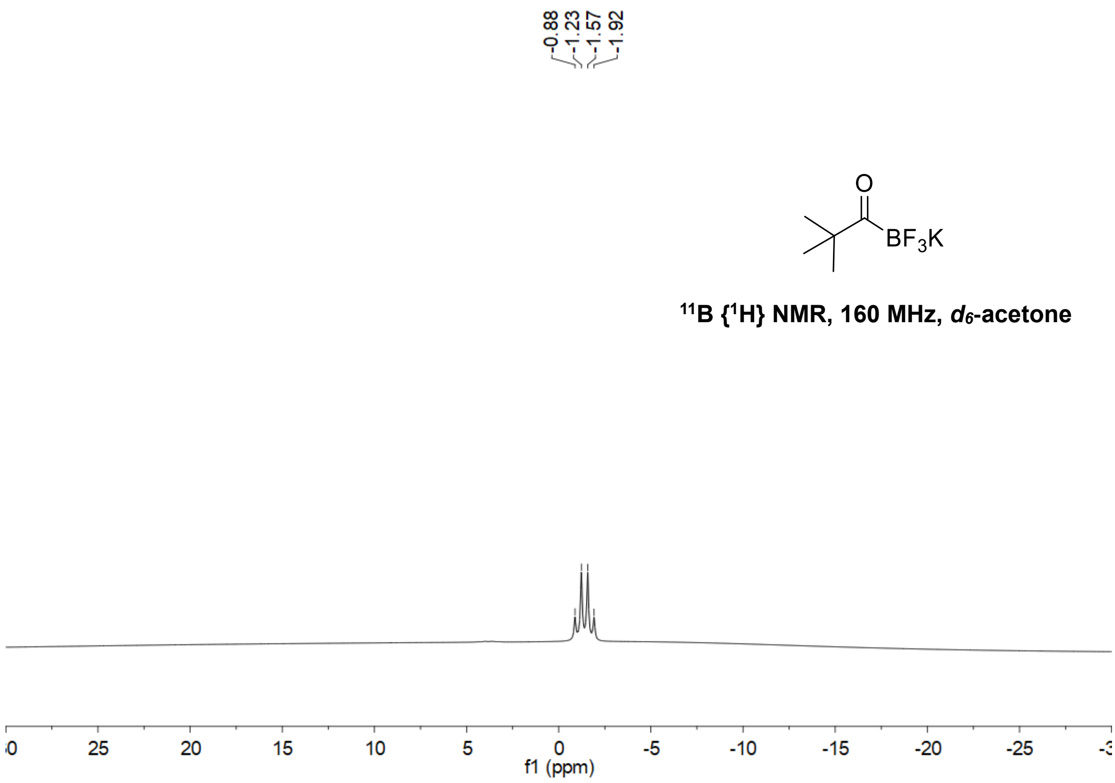

^{11}B {^{1}H} NMR, 160 MHz, *d6*-acetone

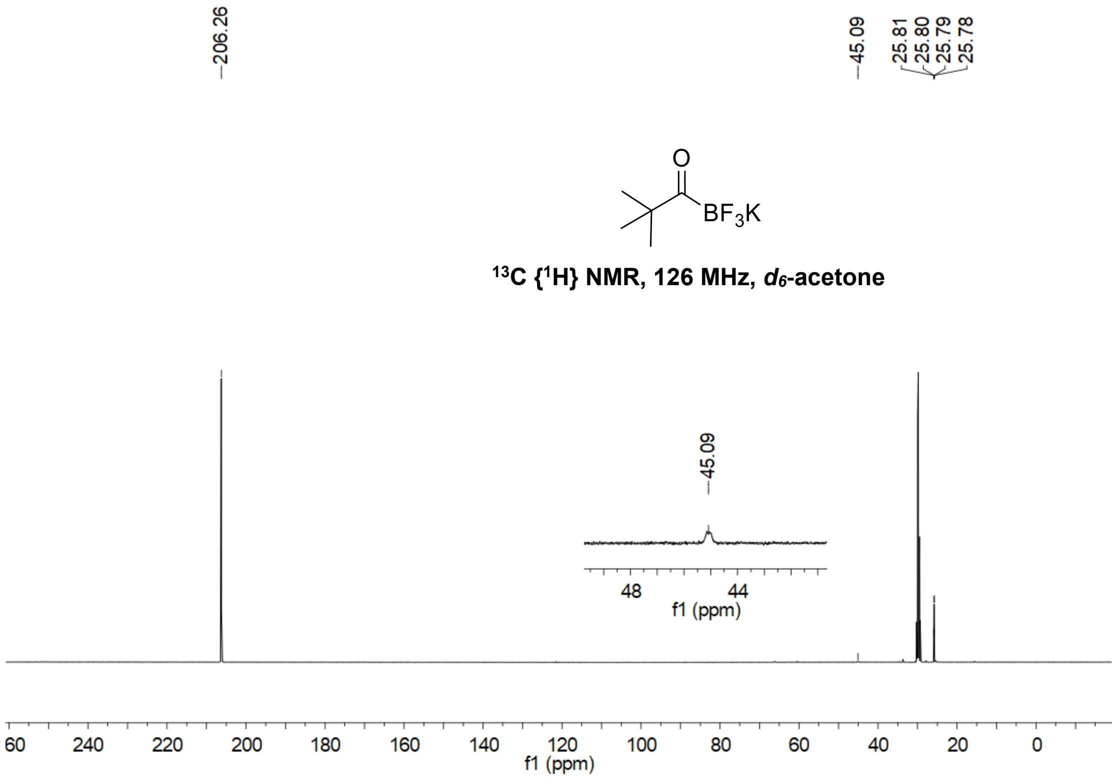

^{13}C {^{1}H} NMR, 126 MHz, *d6*-acetone

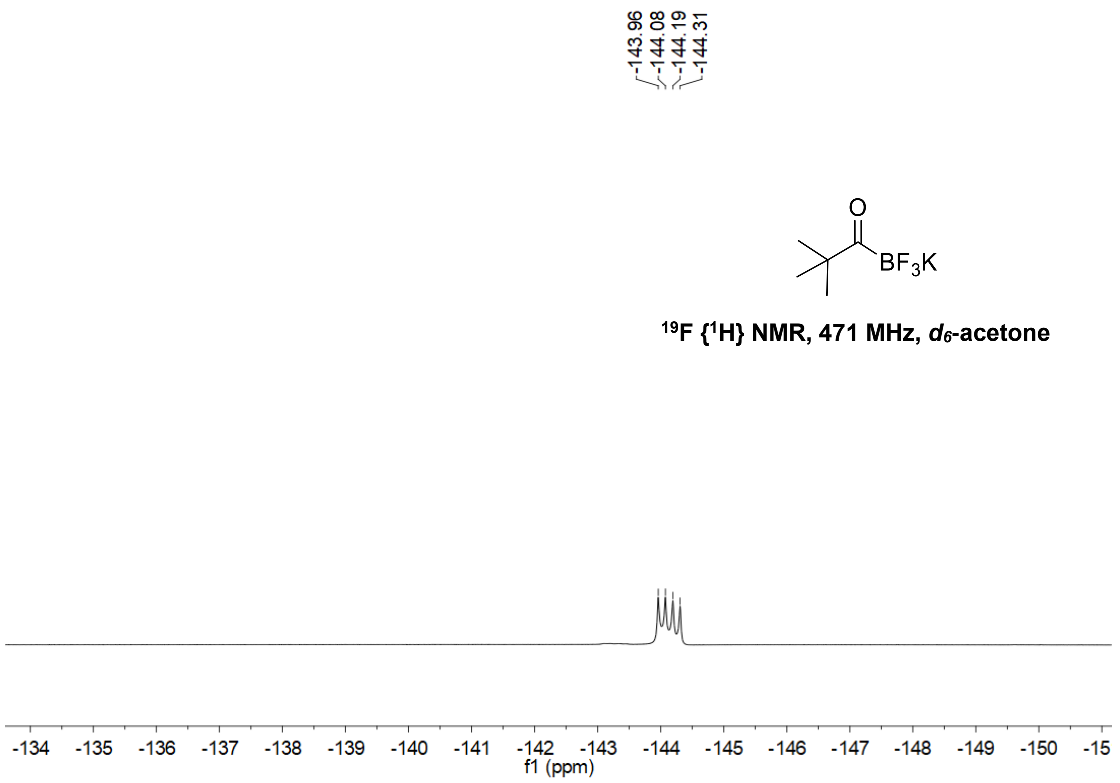

$^{19}F\{^1H\}$ NMR, 471 MHz, d_6-acetone

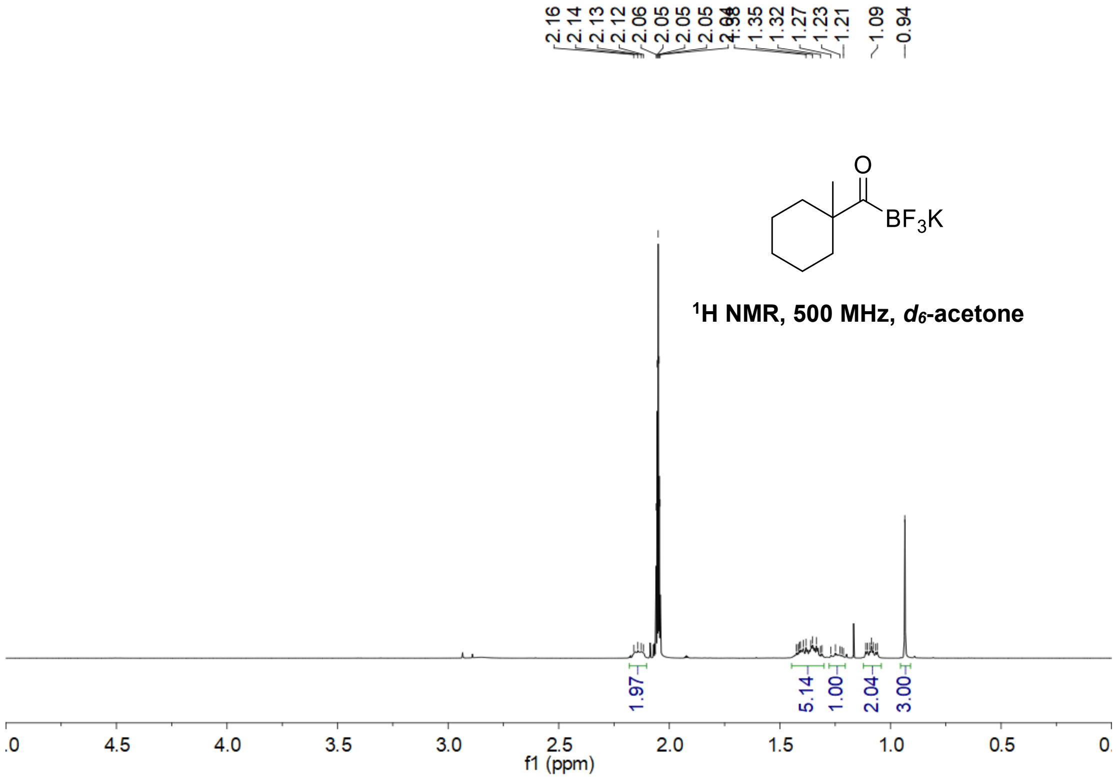

^1H NMR, 500 MHz, d_6-acetone

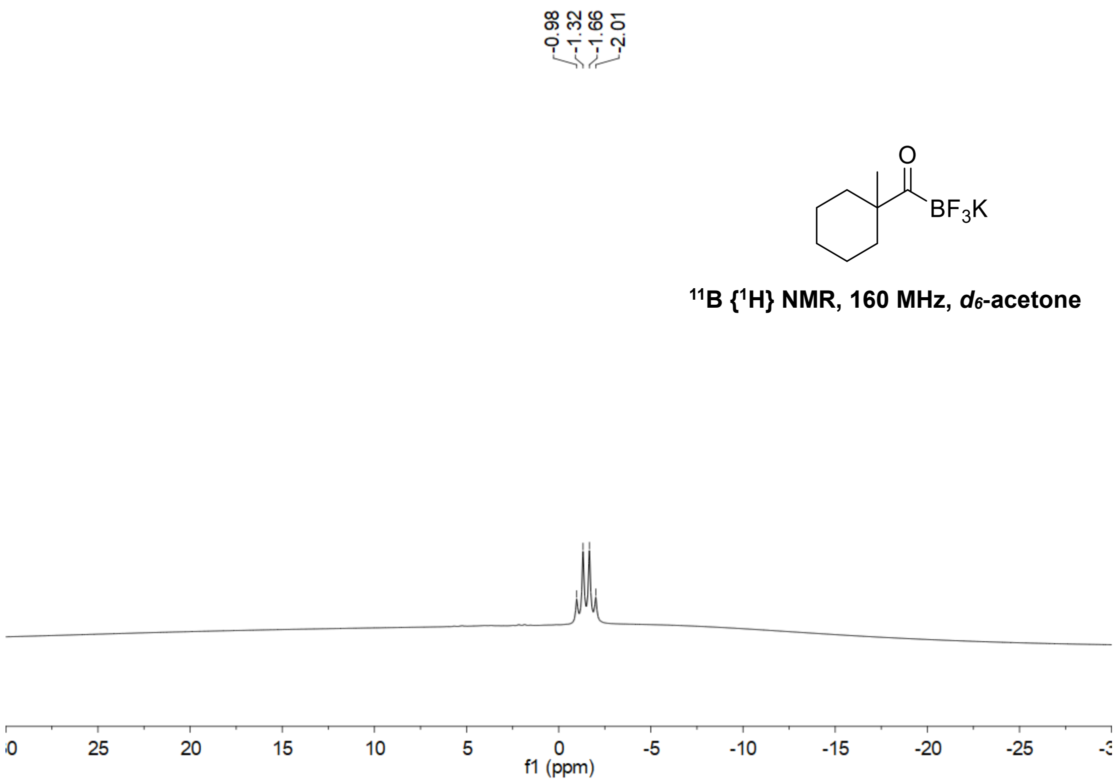
-0.98
-1.32
-1.66
-2.01
O
BF3K
11B {1H} NMR, 160 MHz, d6-acetone
25
20
15
10
5
0
-5
-10
-15
-20
-25
f1 (ppm)

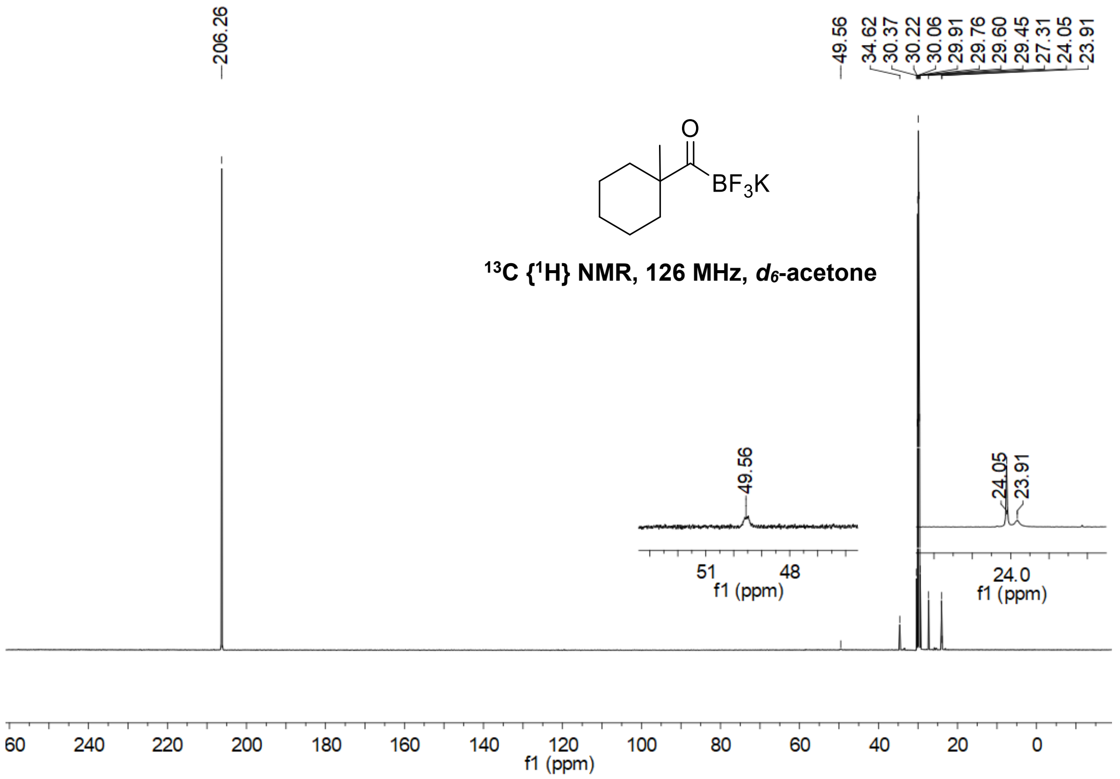
206.26
49.56
34.62
30.37
30.22
30.06
29.91
29.76
29.60
29.45
27.31
24.05
23.91
O
BF3K
13C {1H} NMR, 126 MHz, d6-acetone
49.56
51
48
f1 (ppm)
24.05
23.91
24.0
f1 (ppm)
240
220
200
180
160
140
120
100
80
60
40
20
0
f1 (ppm)

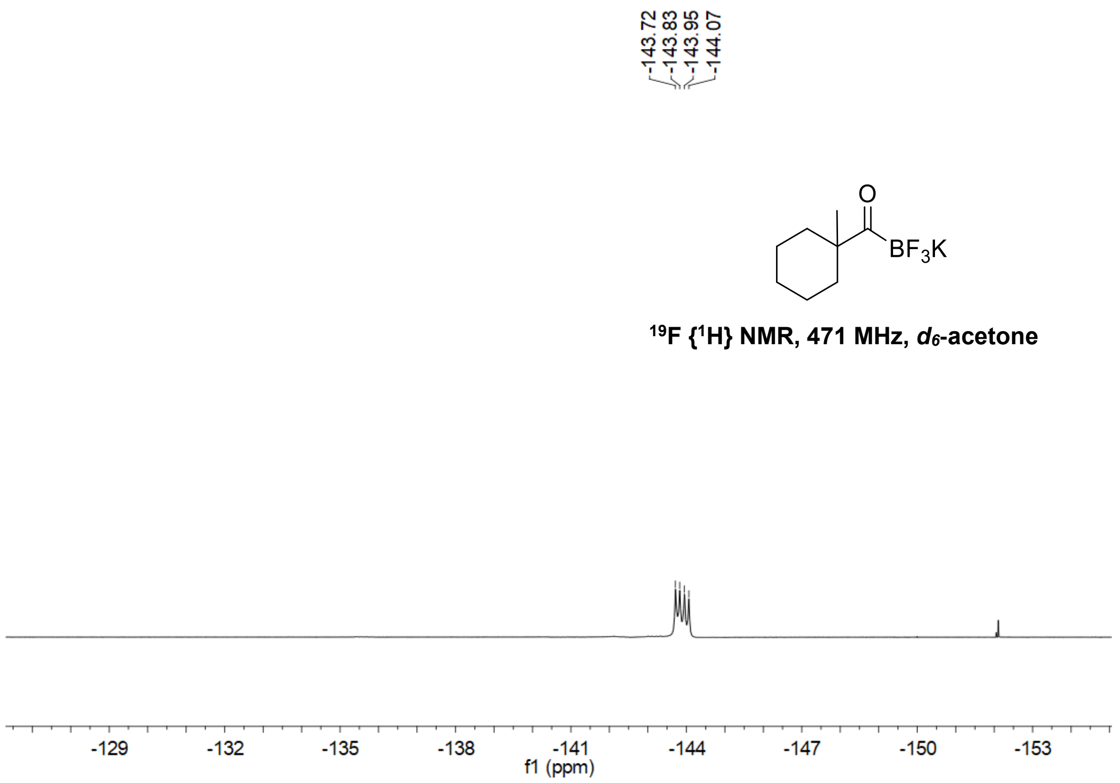

^{19}F {^{1}H} NMR, 471 MHz, *d*₆-acetone

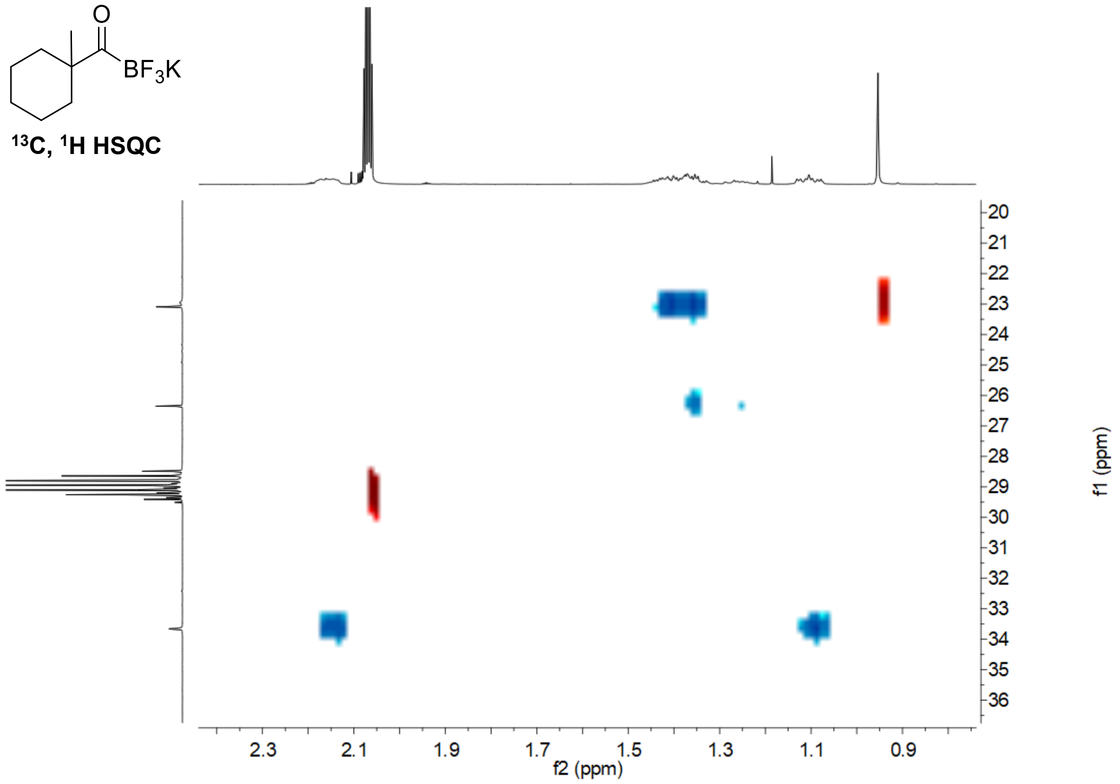

^{13}C, ^{1}H HSQC

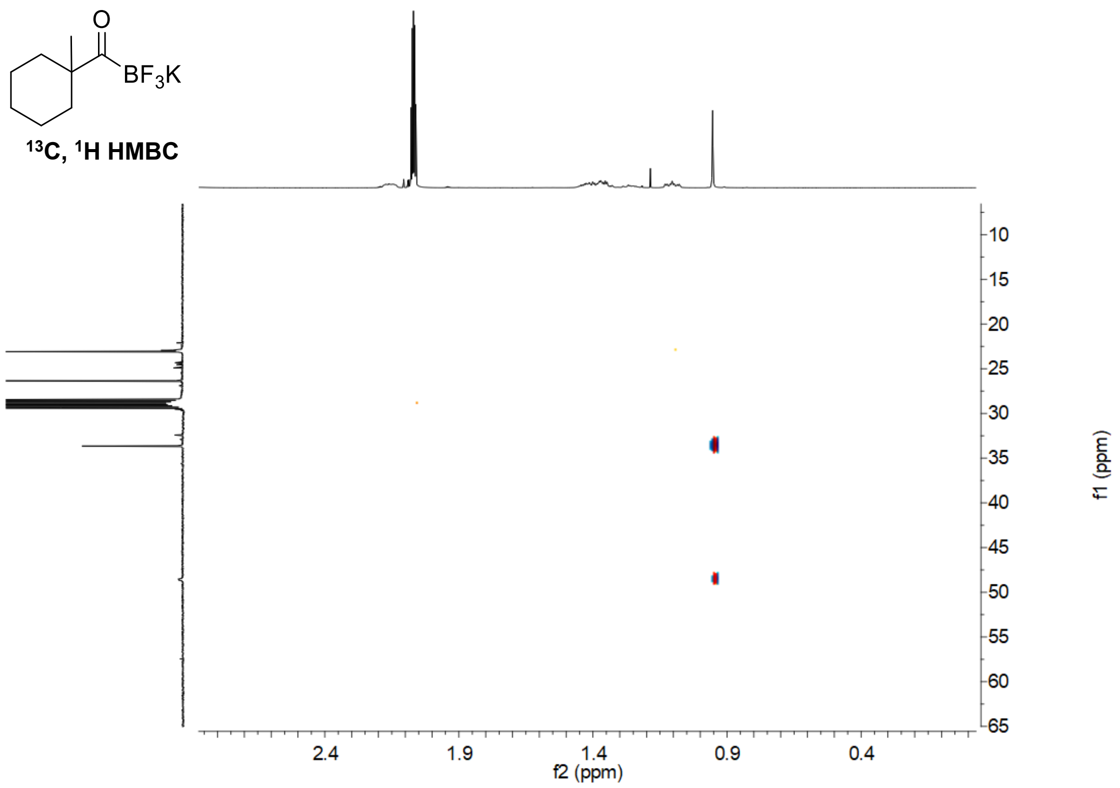
O
BF3K
13C, 1H HMBC
f2 (ppm)
f1 (ppm)

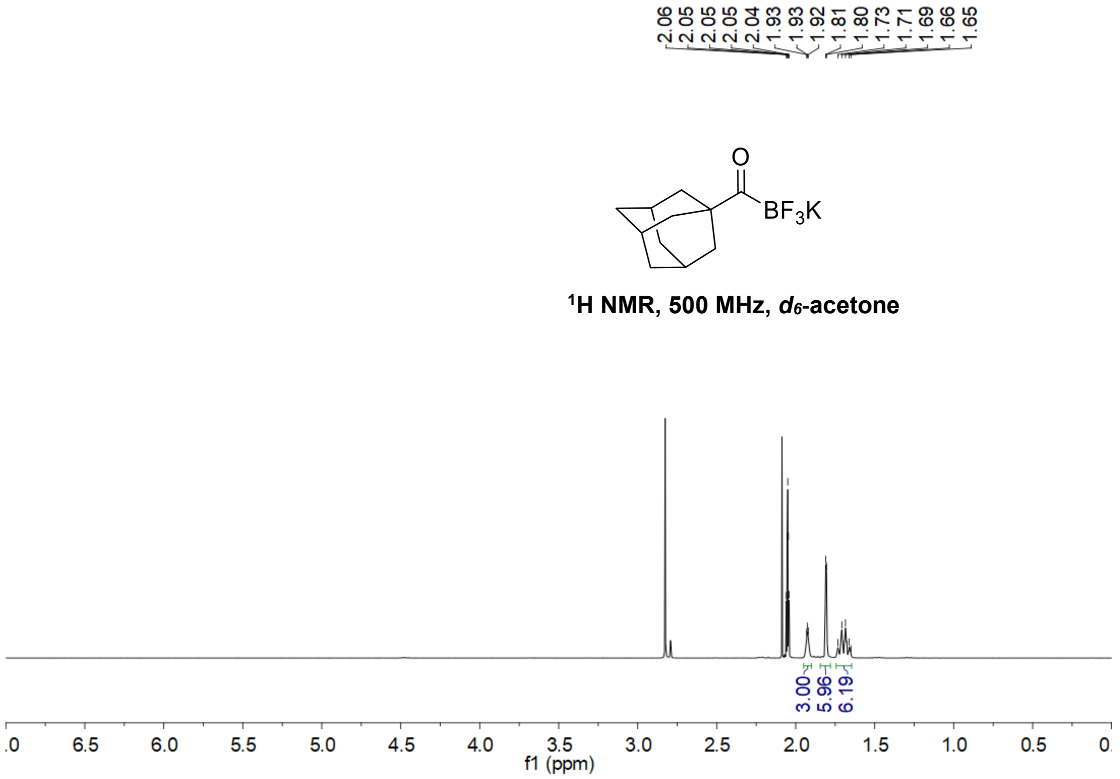
O
BF3K
1H NMR, 500 MHz, d6-acetone
2.06
2.05
2.05
2.05
2.04
1.93
1.93
1.92
1.81
1.80
1.73
1.71
1.69
1.66
1.65
3.00
5.96
6.19
f1 (ppm)

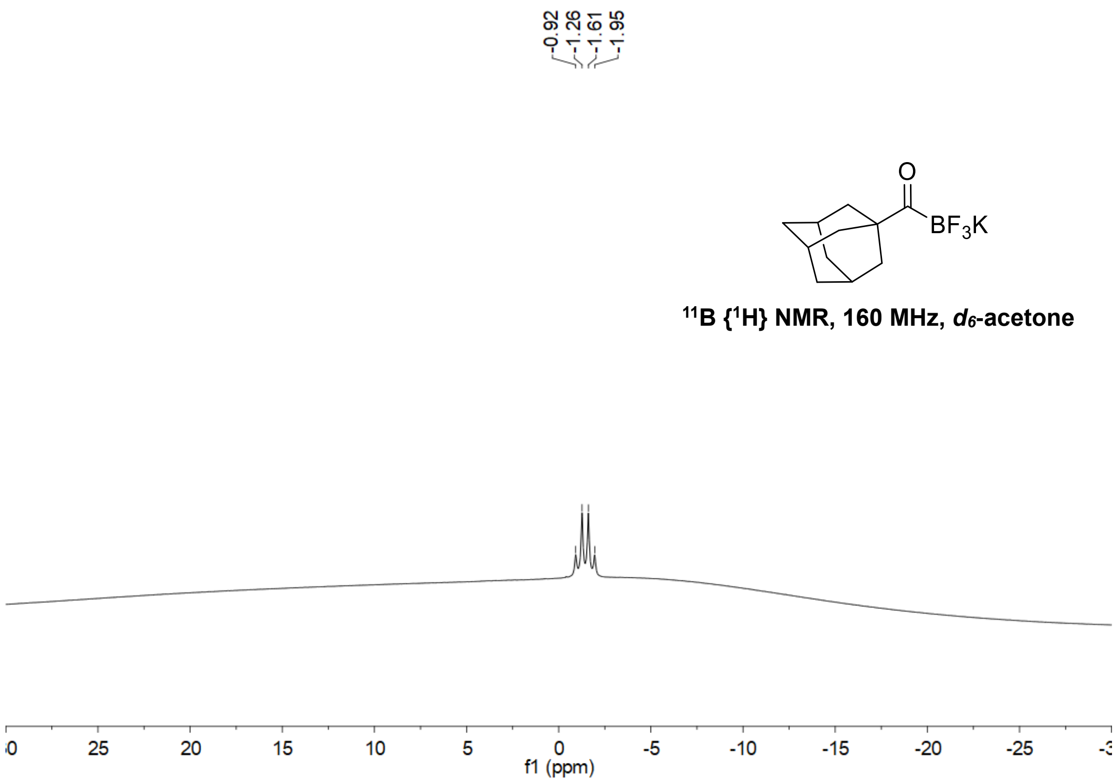
-0.92
-1.26
-1.61
-1.95
O
BF3K
11B {1H} NMR, 160 MHz, d6-acetone
25
20
15
10
5
0
-5
-10
-15
-20
-25
f1 (ppm)

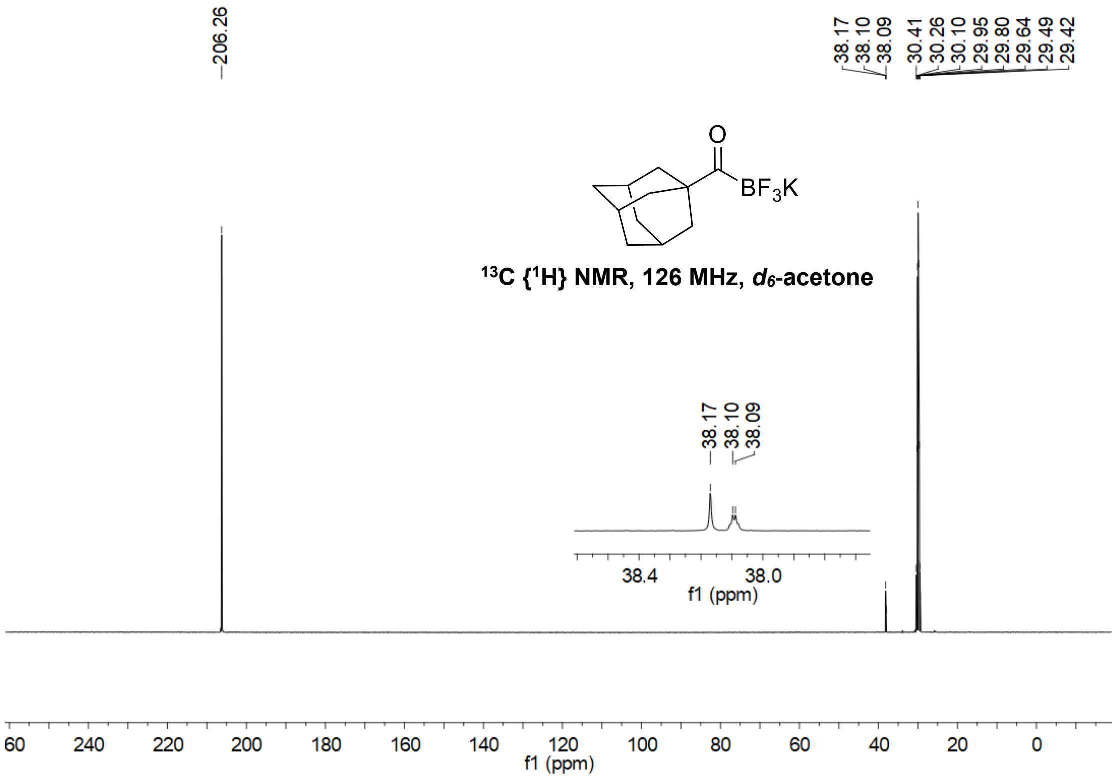
206.26
38.17
38.10
38.09
30.41
30.26
30.10
29.95
29.80
29.64
29.49
29.42
O
BF3K
13C {1H} NMR, 126 MHz, d6-acetone
38.17
38.10
38.09
38.4
38.0
f1 (ppm)
240
220
200
180
160
140
120
100
80
60
40
20
0
f1 (ppm)

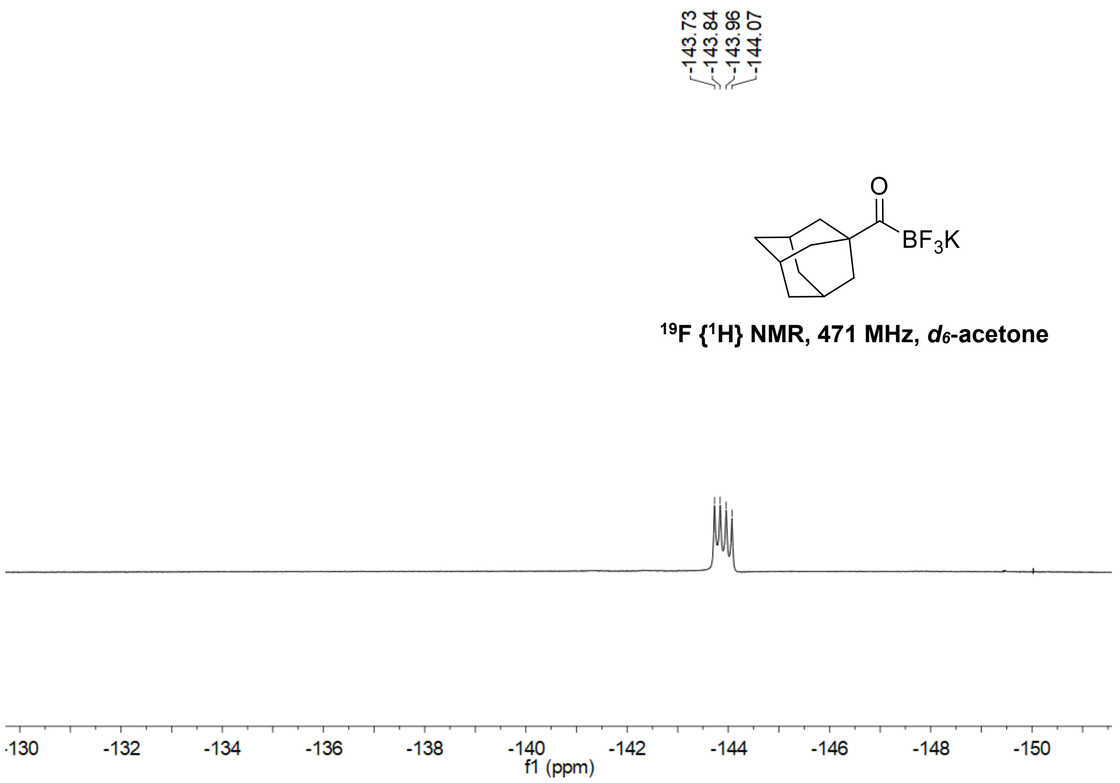
-143.73
-143.84
-143.96
-144.07
BF3K
O
19F {1H} NMR, 471 MHz, d6-acetone
-130 -132 -134 -136 -138 -140 -142 -144 -146 -148 -150
f1 (ppm)

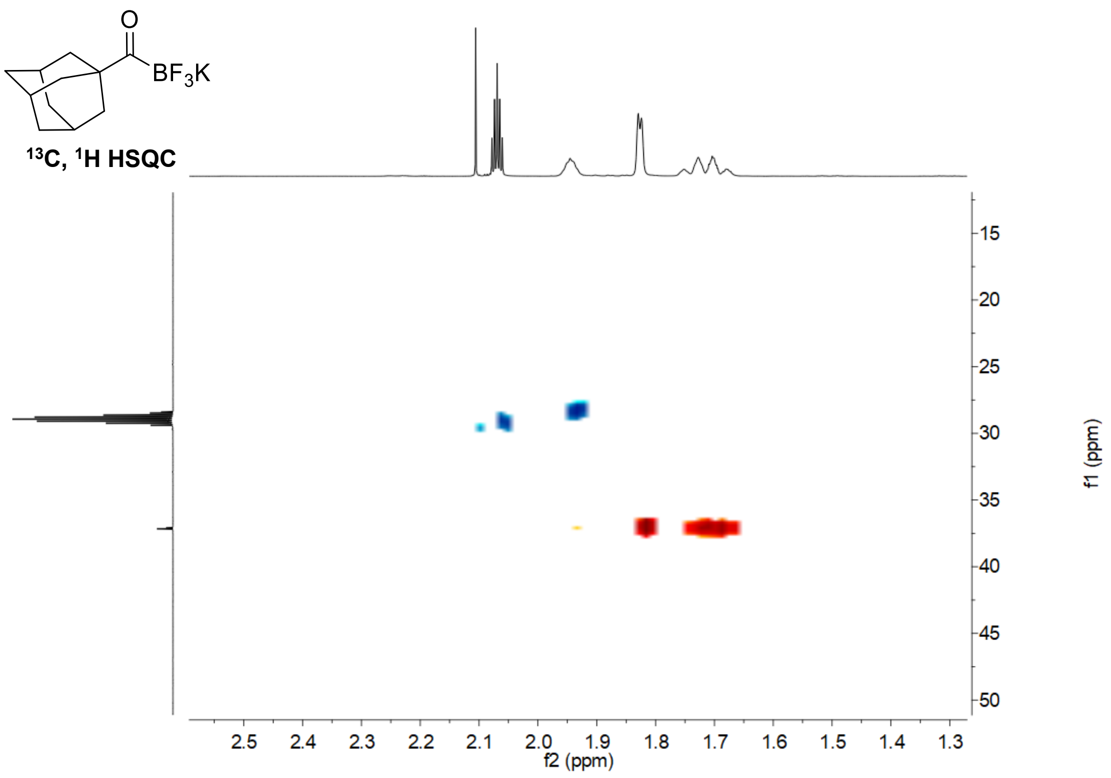
O
BF3K
13C, 1H HSQC
2.5 2.4 2.3 2.2 2.1 2.0 1.9 1.8 1.7 1.6 1.5 1.4 1.3
f2 (ppm)
15 20 25 30 35 40 45 50
f1 (ppm)

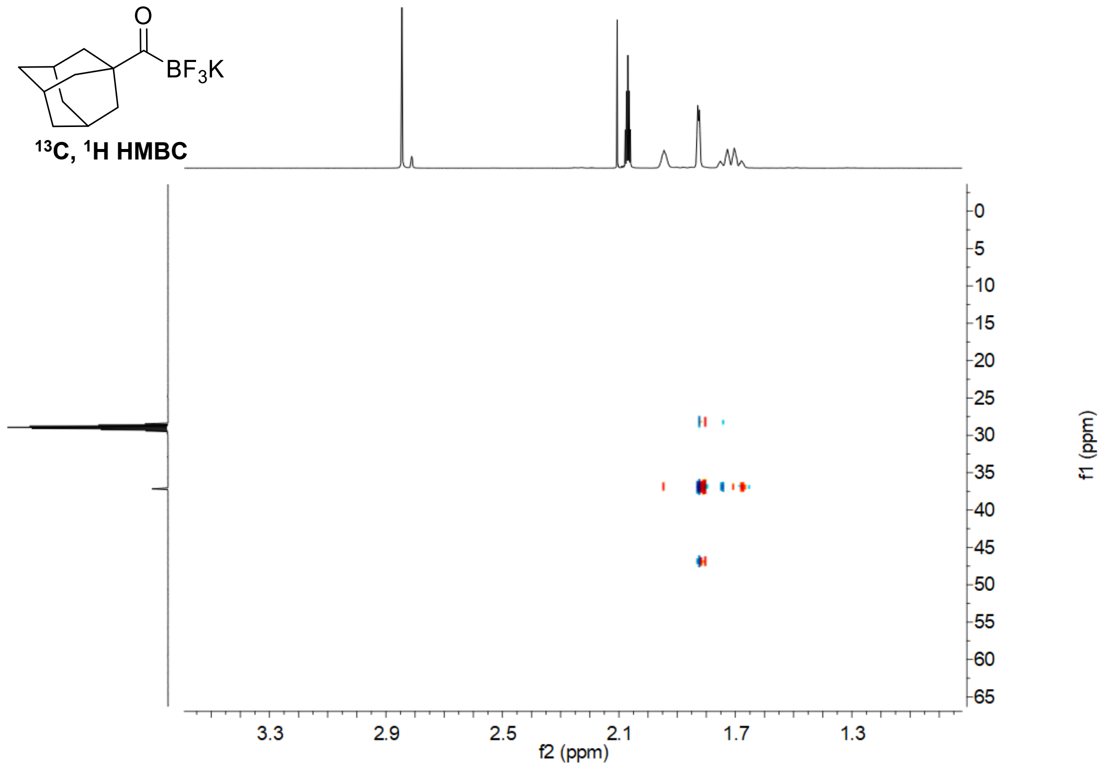

O
BF3K
13C, 1H HMBC
f1 (ppm)
f2 (ppm)

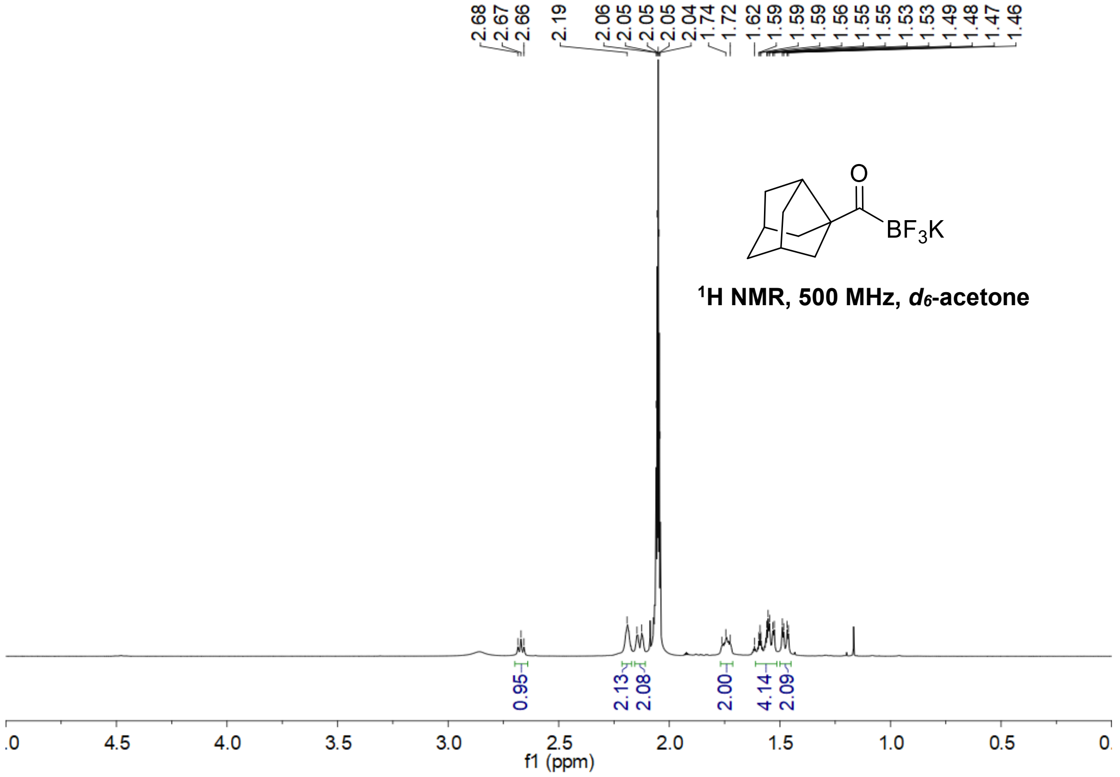

O
BF3K
1H NMR, 500 MHz, d6-acetone
f1 (ppm)

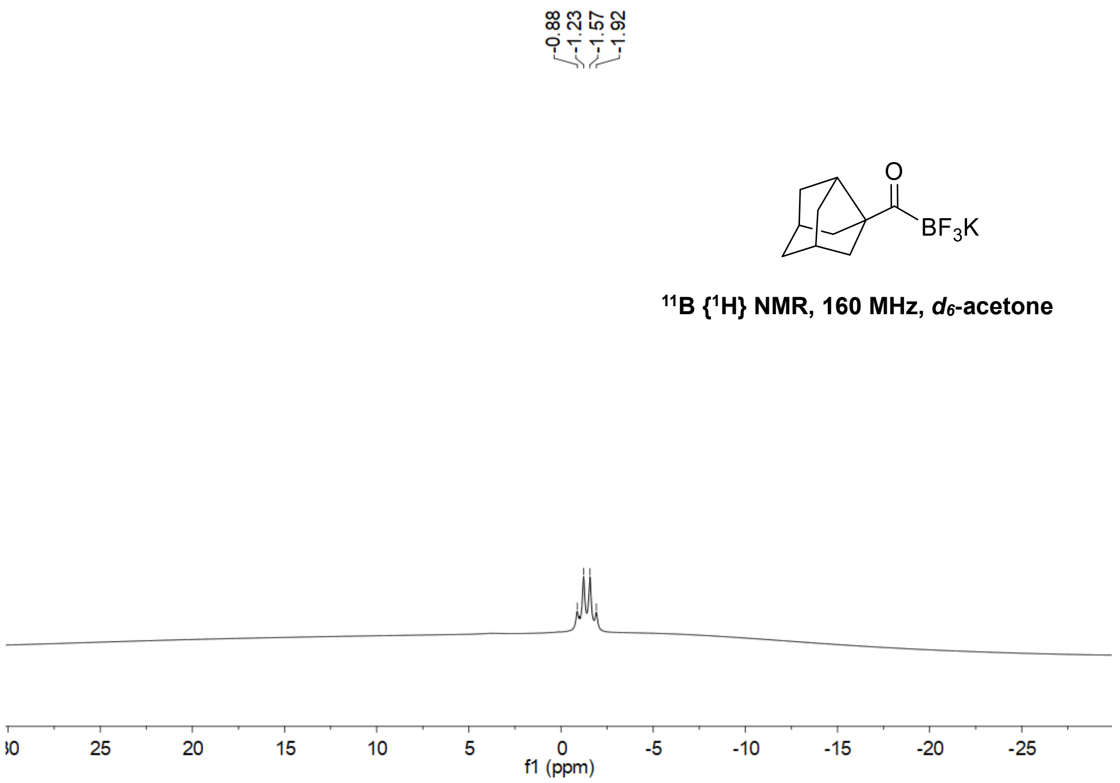
-0.88
-1.23
-1.57
-1.92
O
BF3K
11B {1H} NMR, 160 MHz, d6-acetone
30
25
20
15
10
5
0
-5
-10
-15
-20
-25
f1 (ppm)

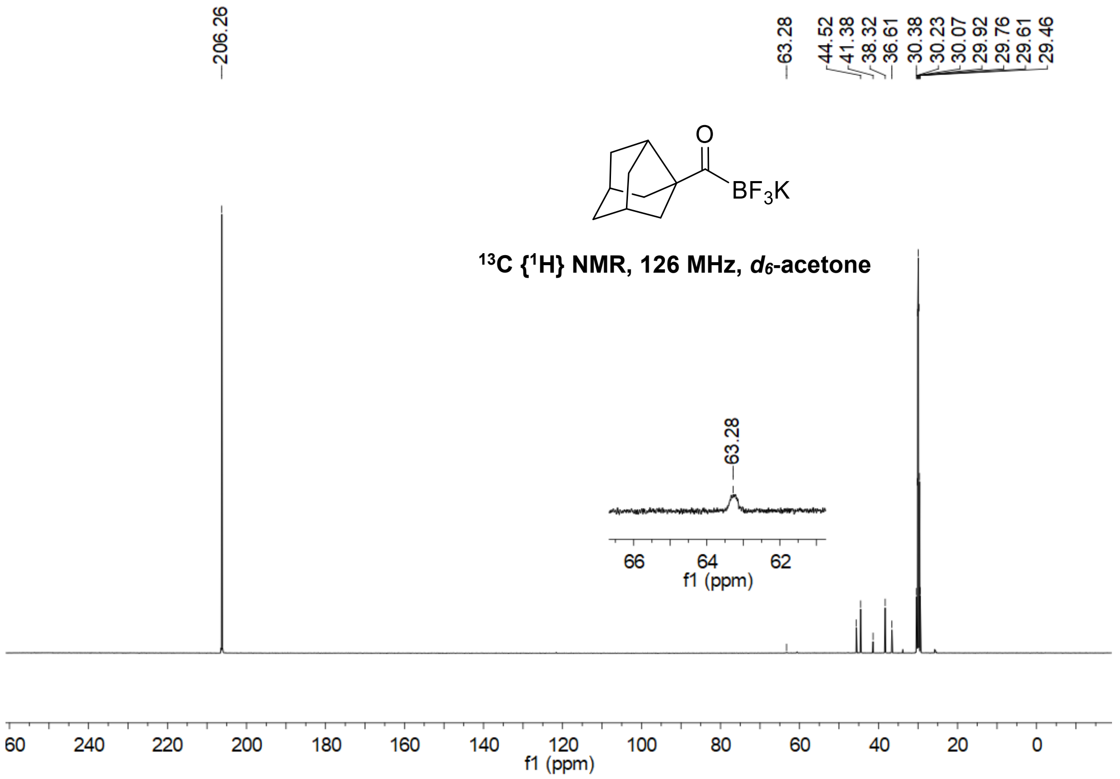
-206.26
-63.28
44.52
41.38
38.32
36.61
30.38
30.23
30.07
29.92
29.76
29.61
29.46
O
BF3K
13C {1H} NMR, 126 MHz, d6-acetone
63.28
66
64
62
f1 (ppm)
260
240
220
200
180
160
140
120
100
80
60
40
20
0
f1 (ppm)

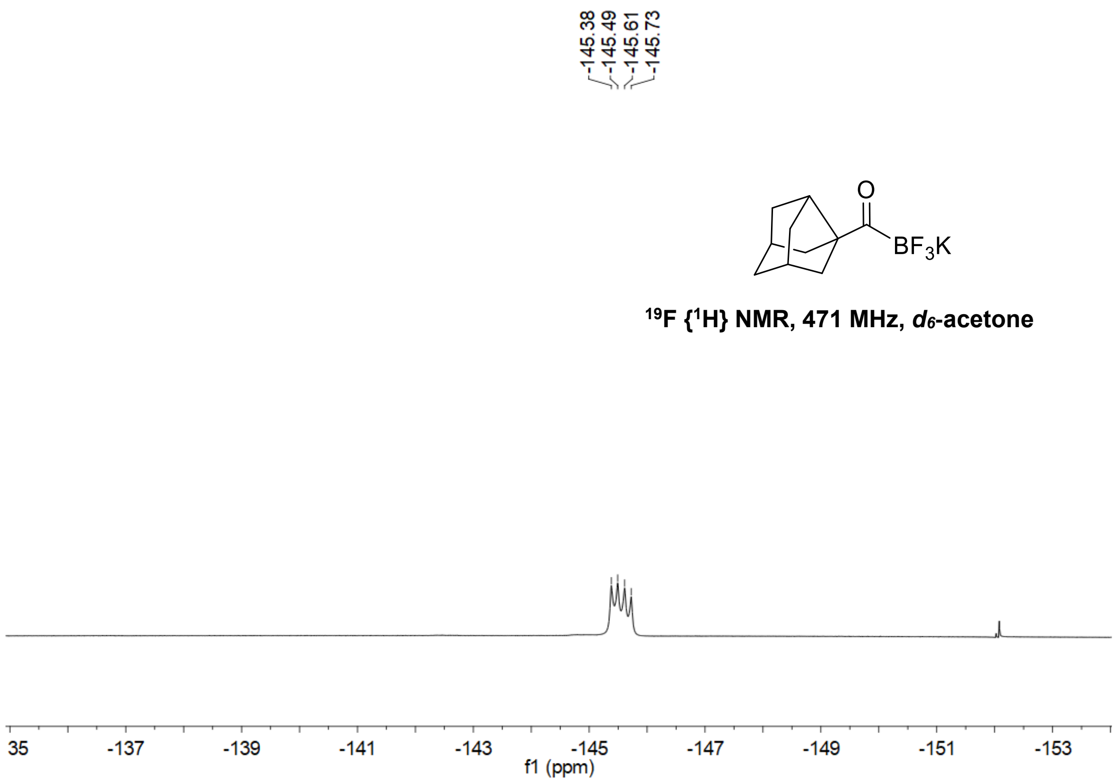

^{19}F {^{1}H} NMR, 471 MHz, *d6*-acetone

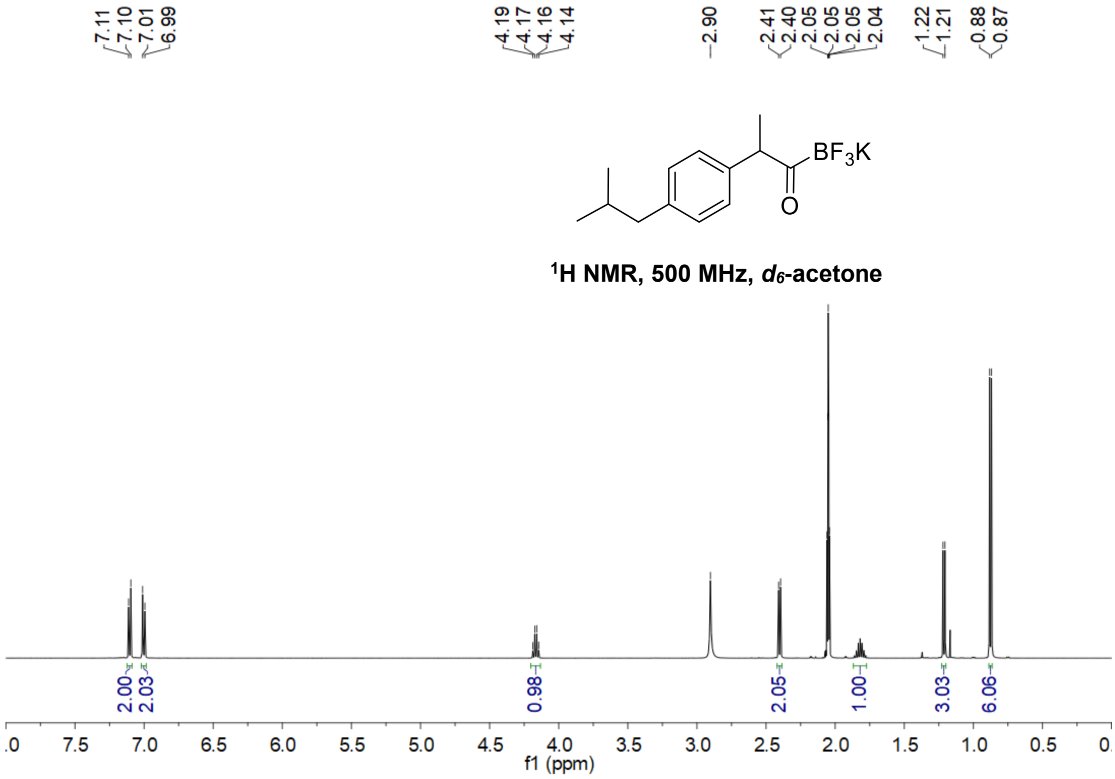

^{1}H NMR, 500 MHz, *d6*-acetone

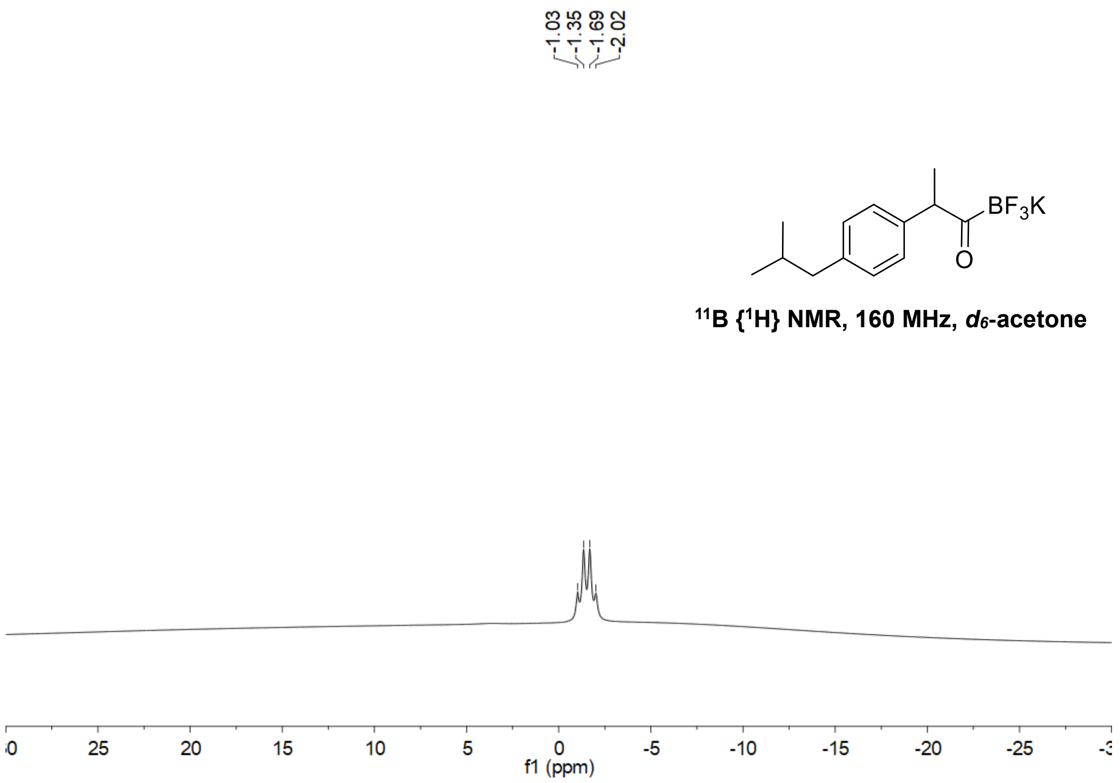
-1.03
-1.35
-1.69
-2.02
BF3K
O
11B {1H} NMR, 160 MHz, d6-acetone
25
20
15
10
5
0
-5
-10
-15
-20
-25
f1 (ppm)

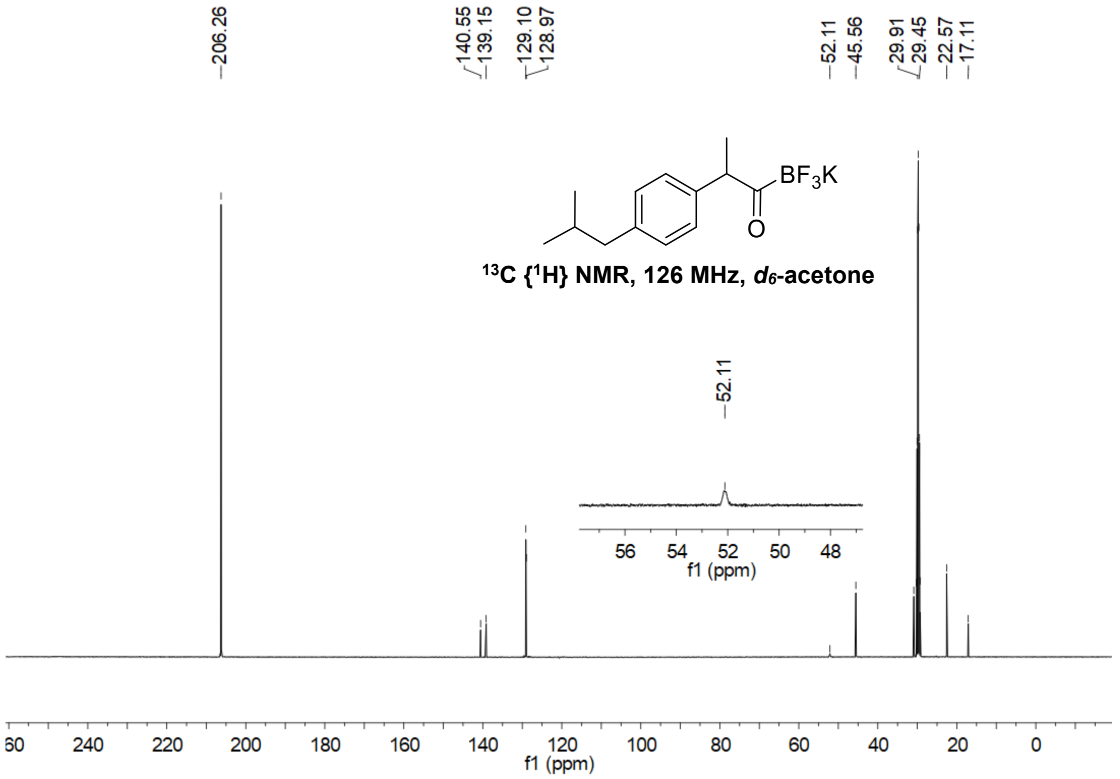
206.26
140.55
139.15
129.10
128.97
52.11
45.56
29.91
29.45
22.57
17.11
BF3K
O
13C {1H} NMR, 126 MHz, d6-acetone
52.11
56
54
52
50
48
f1 (ppm)
240
220
200
180
160
140
120
100
80
60
40
20
0
f1 (ppm)

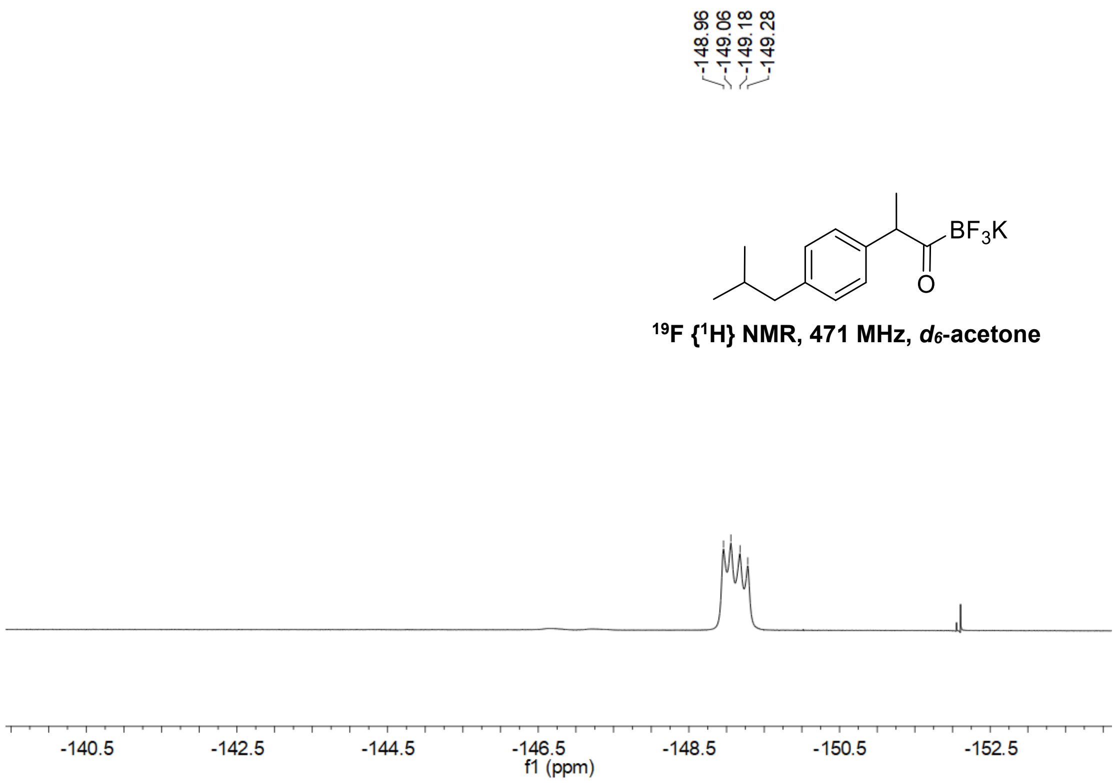
-148.96
-149.06
-149.18
-149.28
BF3K
O
19F {1H} NMR, 471 MHz, d6-acetone
-140.5
-142.5
-144.5
-146.5
-148.5
-150.5
-152.5
f1 (ppm)

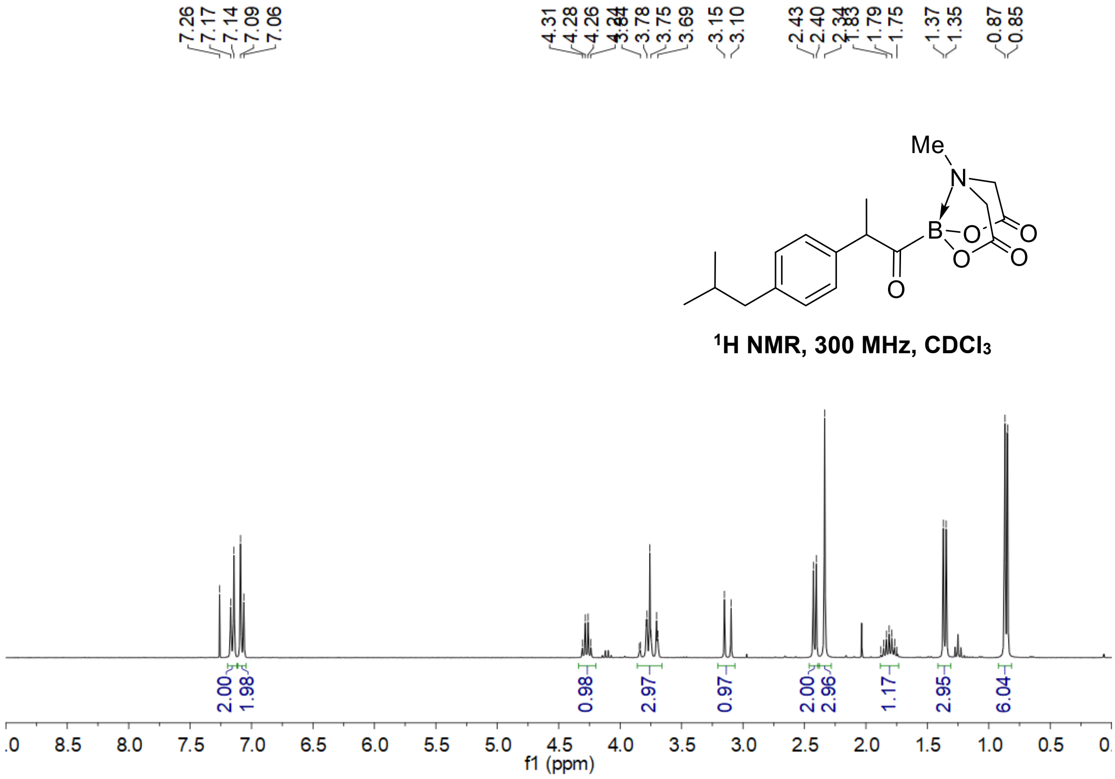
7.26
7.17
7.14
7.09
7.06
4.31
4.28
4.26
3.78
3.75
3.69
3.15
3.10
2.43
2.40
1.83
1.79
1.75
1.37
1.35
0.87
0.85
Me
N
B
O
O
O
O
O
1H NMR, 300 MHz, CDCl3
2.00
1.98
0.98
2.97
0.97
2.00
2.96
1.17
2.95
6.04
8.5
8.0
7.5
7.0
6.5
6.0
5.5
5.0
4.5
4.0
3.5
3.0
2.5
2.0
1.5
1.0
0.5
f1 (ppm)

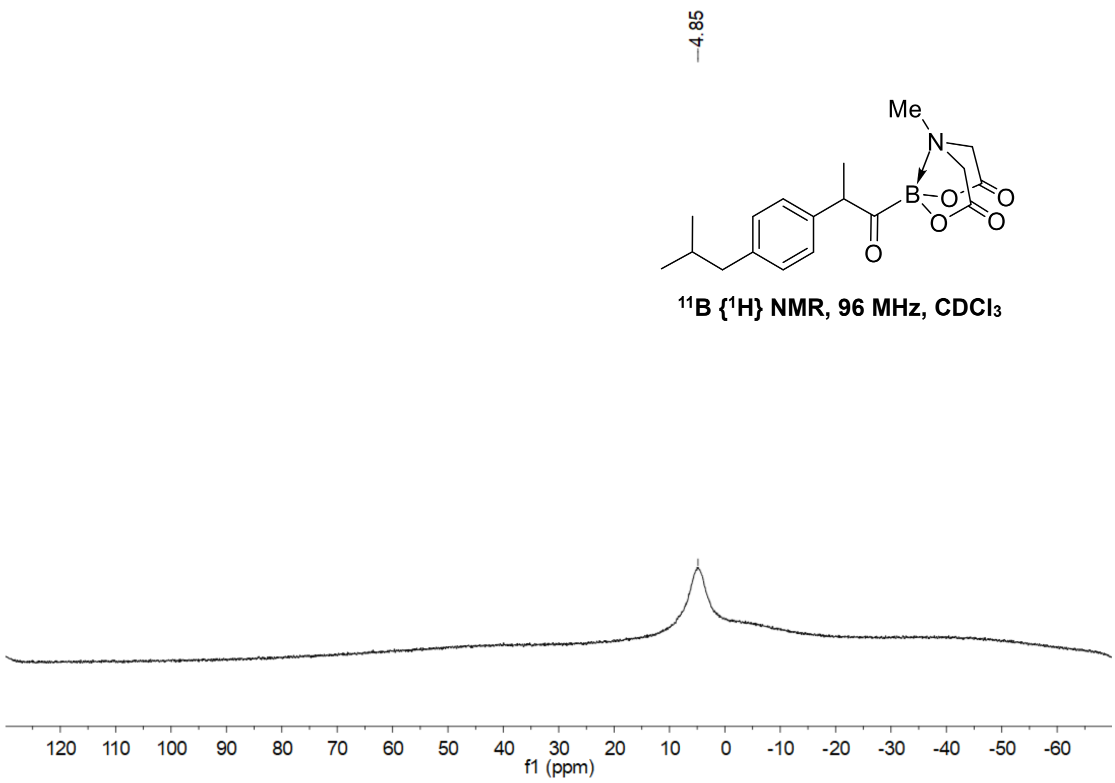

-4.85
Me
N
B
O
O
O
O
O
¹¹B {¹H} NMR, 96 MHz, CDCl₃
120 110 100 90 80 70 60 50 40 30 20 10 0 -10 -20 -30 -40 -50 -60
f1 (ppm)

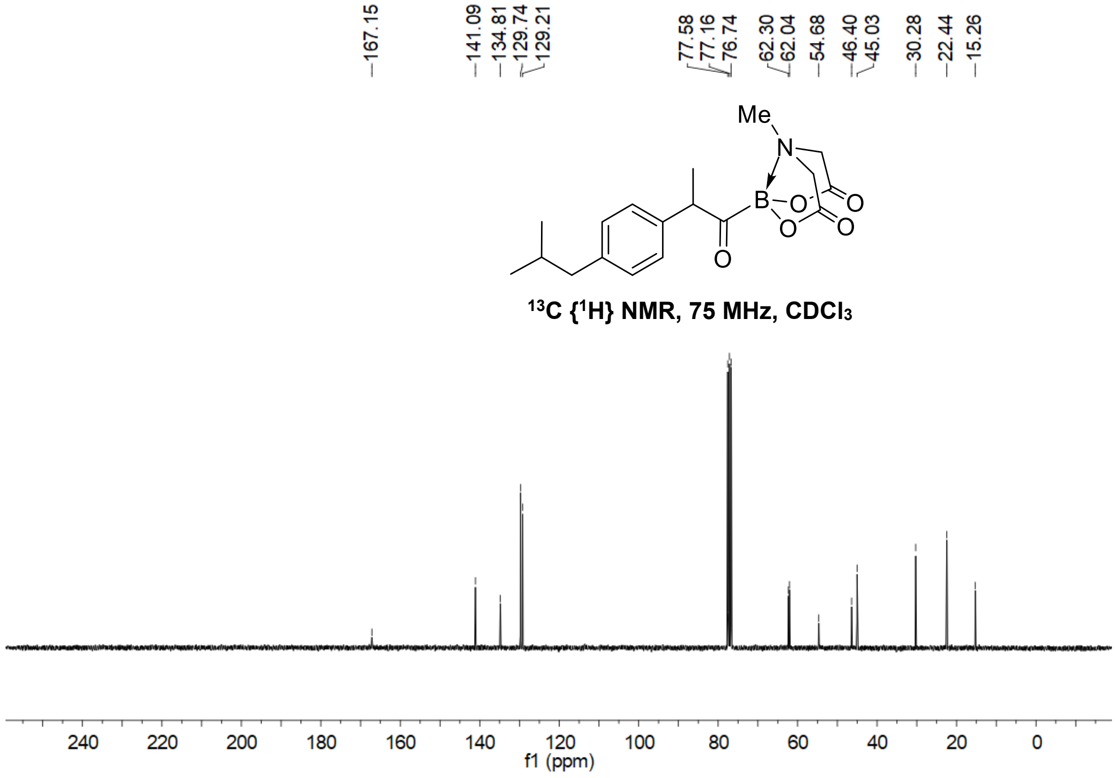

-167.15
-141.09
-134.81
129.74
129.21
77.58
77.16
76.74
62.30
62.04
-54.68
46.40
45.03
-30.28
-22.44
-15.26
Me
N
B
O
O
O
O
O
¹³C {¹H} NMR, 75 MHz, CDCl₃
240 220 200 180 160 140 120 100 80 60 40 20 0
f1 (ppm)

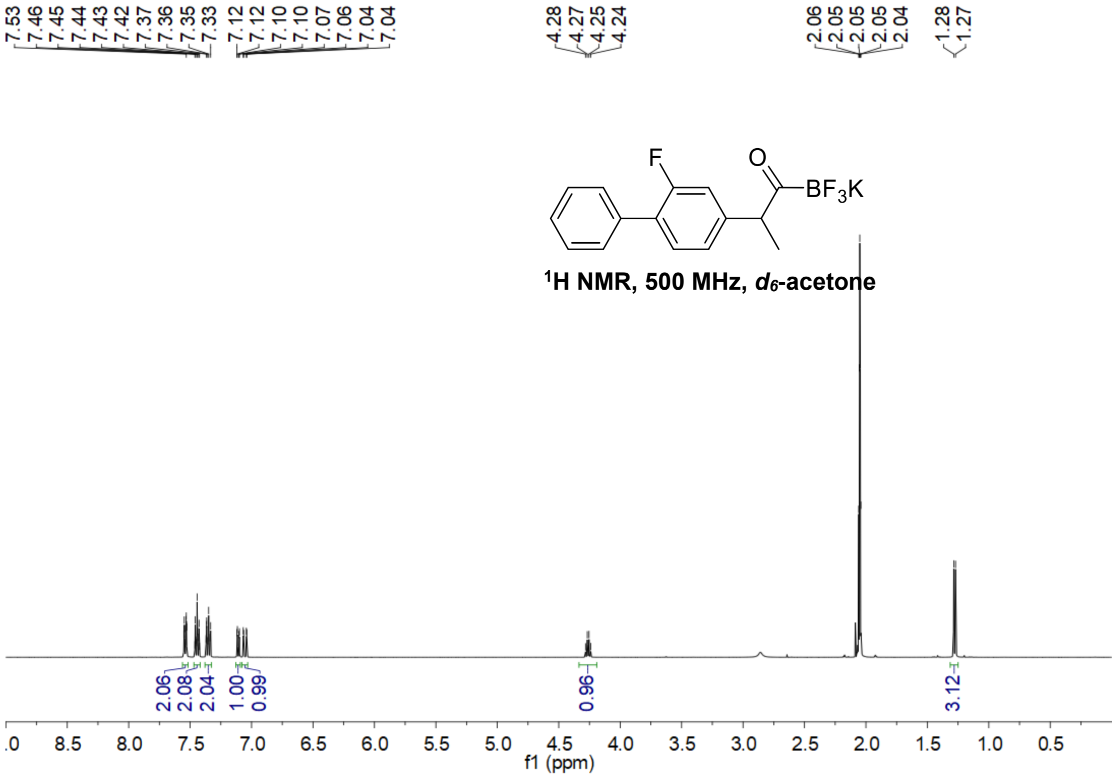
7.53
7.46
7.45
7.44
7.43
7.42
7.37
7.36
7.35
7.33
7.12
7.12
7.10
7.10
7.07
7.06
7.04
7.04
4.28
4.27
4.25
4.24
2.06
2.05
2.05
2.05
2.04
1.28
1.27
F
O
BF3K
1H NMR, 500 MHz, d6-acetone
2.06
2.08
2.04
1.00
0.99
0.96
3.12
8.5
8.0
7.5
7.0
6.5
6.0
5.5
5.0
4.5
4.0
3.5
3.0
2.5
2.0
1.5
1.0
0.5
f1 (ppm)

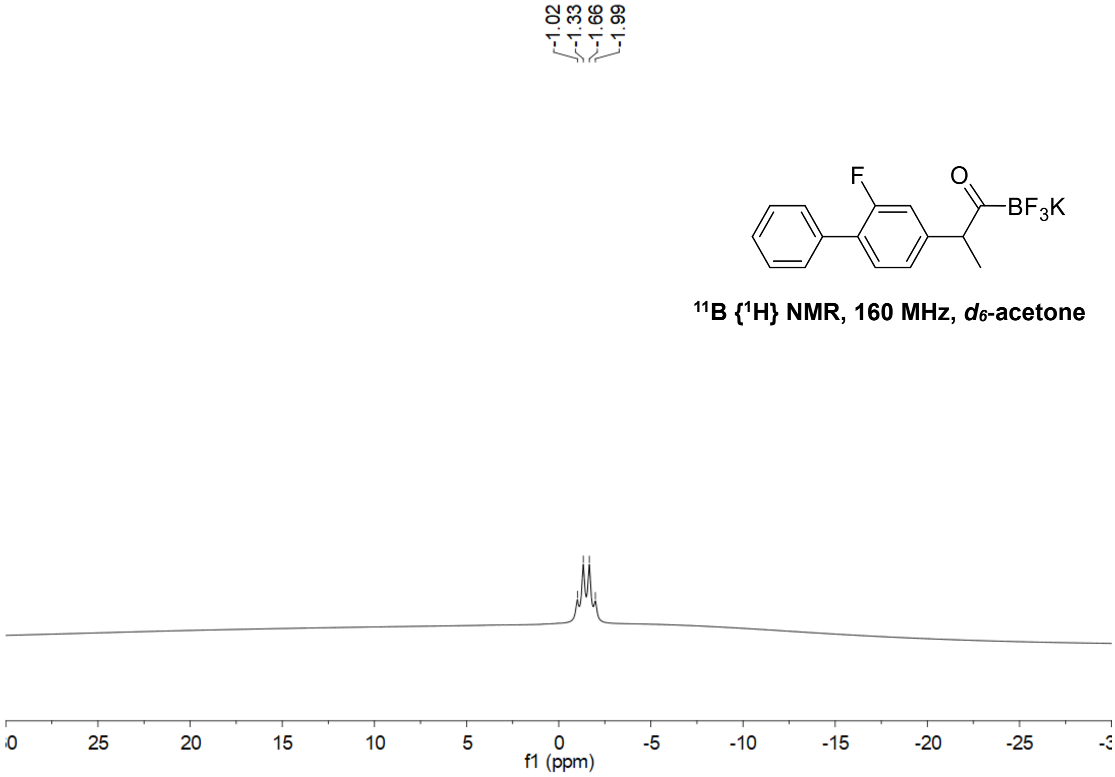
-1.02
-1.33
-1.66
-1.99
F
O
BF3K
11B {1H} NMR, 160 MHz, d6-acetone
25
20
15
10
5
0
-5
-10
-15
-20
-25
f1 (ppm)

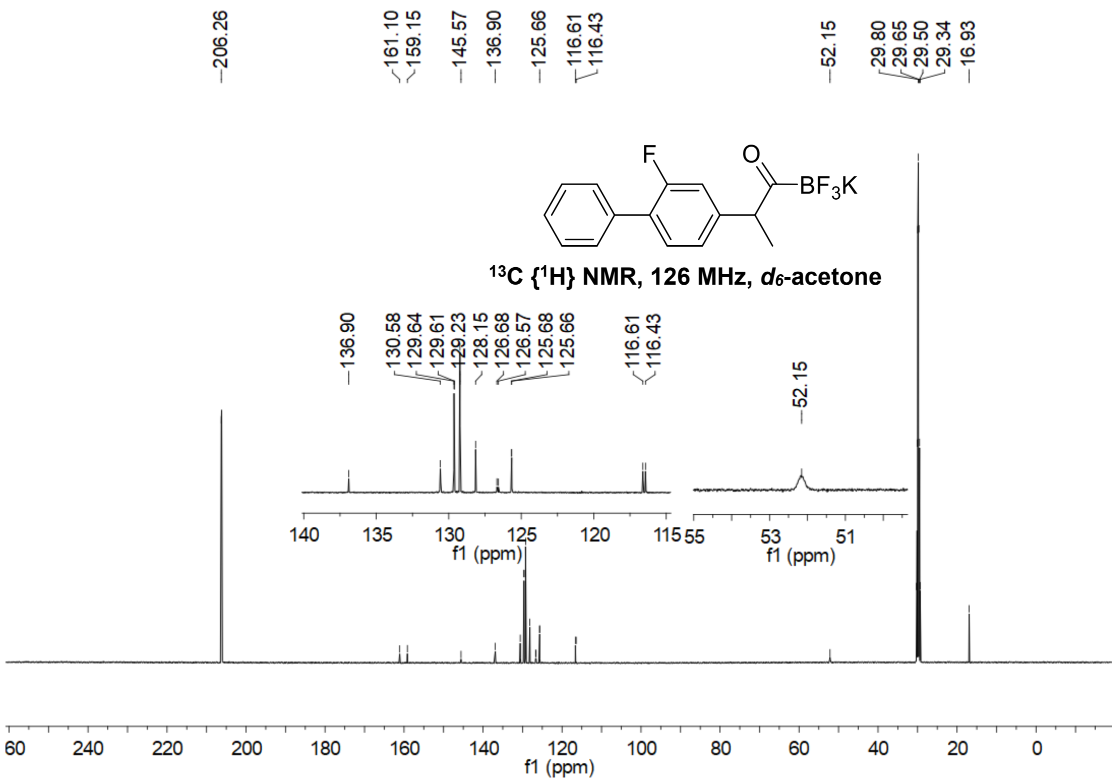
206.26
161.10
159.15
145.57
136.90
125.66
116.61
116.43
52.15
29.80
29.65
29.50
29.34
16.93
F
O
BF3K
13C {1H} NMR, 126 MHz, d6-acetone
136.90
130.58
129.64
129.61
129.23
128.15
126.68
126.57
125.68
125.66
116.61
116.43
52.15
140
135
130
125
120
115
f1 (ppm)
55
53
51
f1 (ppm)
60
240
220
200
180
160
140
120
100
80
60
40
20
0
f1 (ppm)

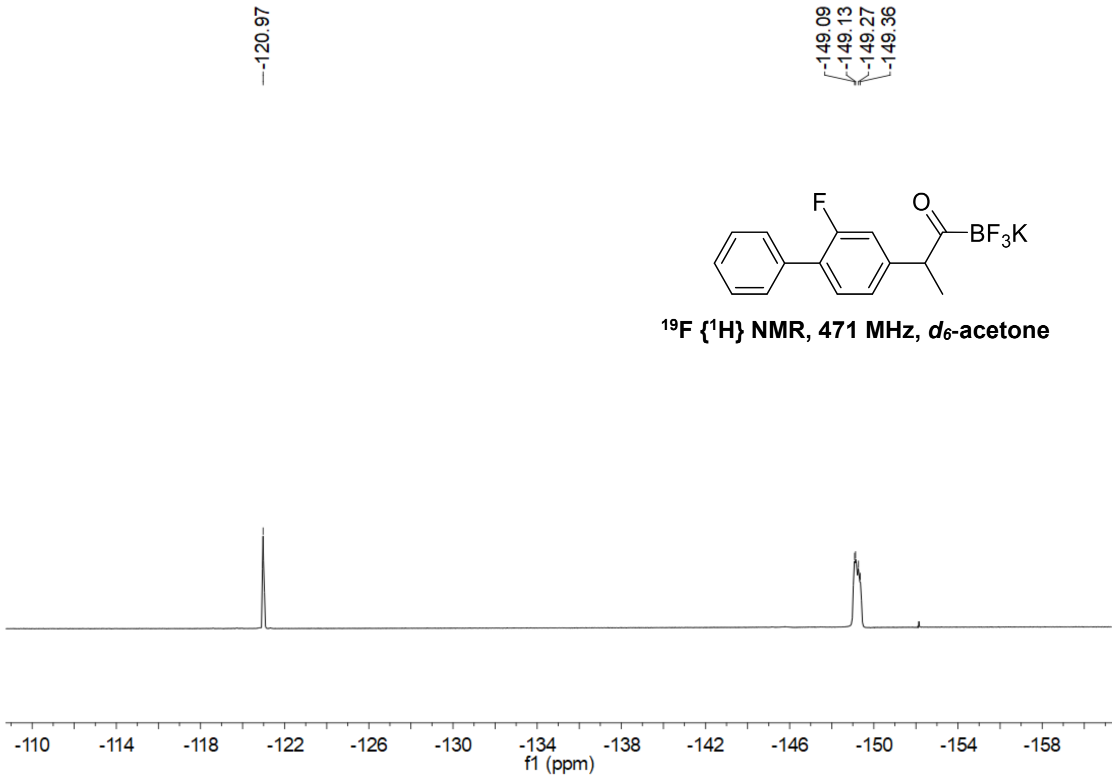
-120.97
-149.09
-149.13
-149.27
-149.36
F
O
BF3K
19F {1H} NMR, 471 MHz, d6-acetone
-110
-114
-118
-122
-126
-130
-134
-138
-142
-146
-150
-154
-158
f1 (ppm)

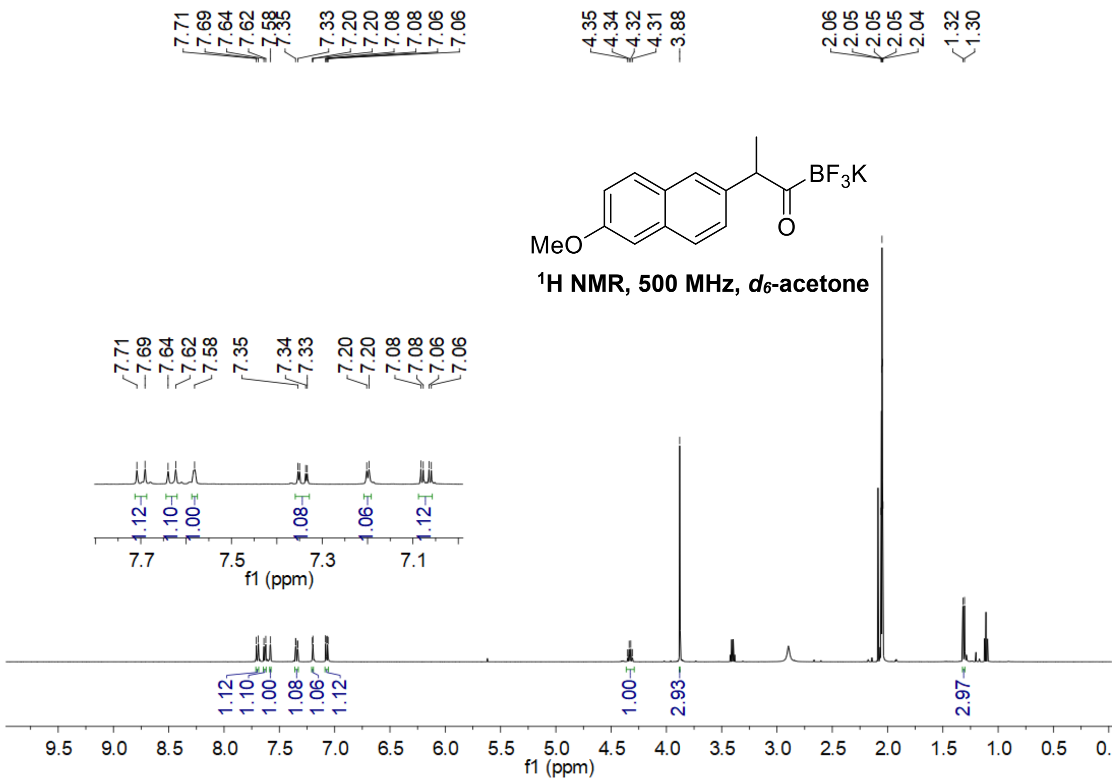
7.71
7.69
7.64
7.62
7.58
7.35
7.33
7.20
7.20
7.08
7.08
7.06
7.06
4.35
4.34
4.32
4.31
3.88
2.06
2.05
2.05
2.05
2.04
1.32
1.30
BF3K
O
MeO
1H NMR, 500 MHz, d6-acetone
7.34
1.12
1.10
1.00
1.08
1.06
7.7
7.5
7.3
7.1
f1 (ppm)
2.93
2.97
9.5
9.0
8.5
8.0
7.5
7.0
6.5
6.0
5.5
5.0
4.5
4.0
3.5
3.0
2.5
2.0
1.5
1.0
0.5

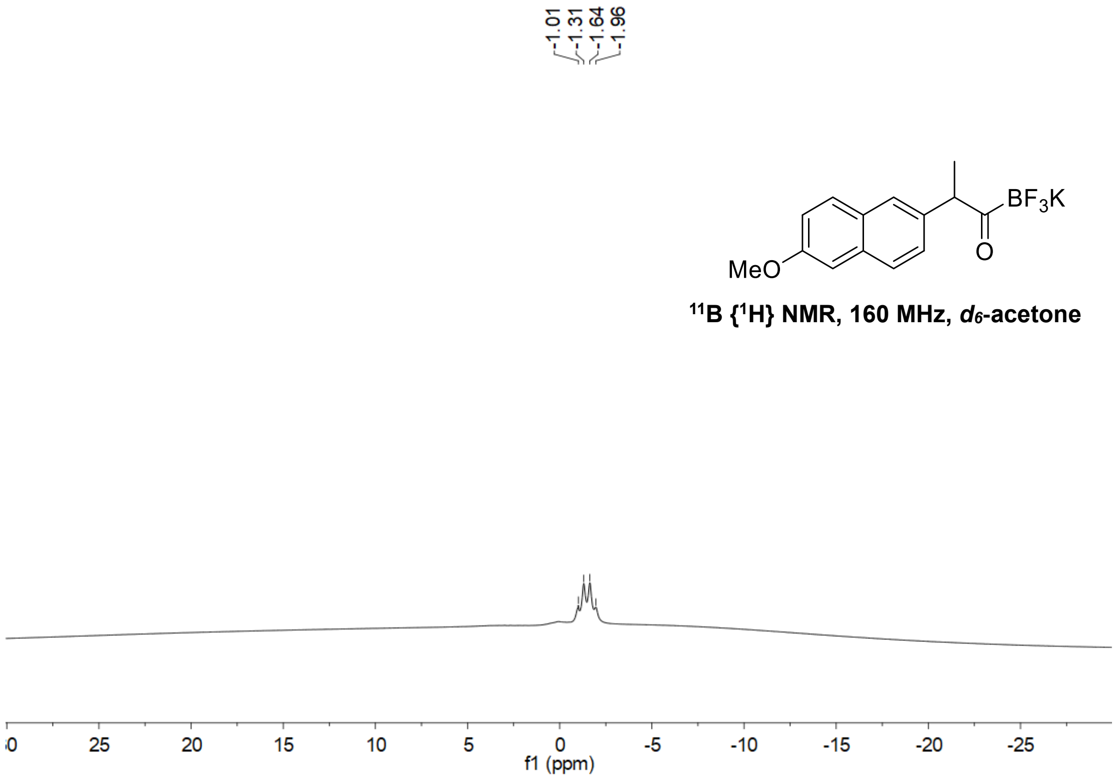
-1.01
-1.31
-1.64
-1.96
BF3K
O
MeO
11B {1H} NMR, 160 MHz, d6-acetone
25
20
15
10
5
0
-5
-10
-15
-20
-25
f1 (ppm)

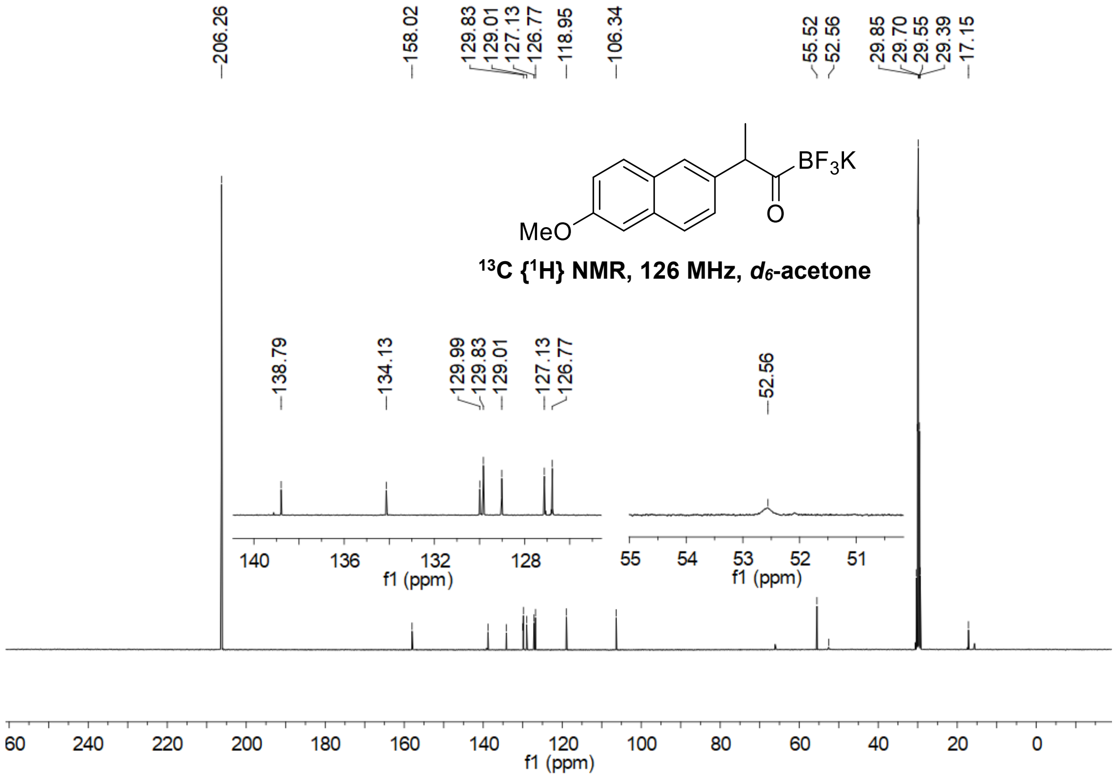
-206.26
-158.02
129.83
129.01
127.13
126.77
-118.95
-106.34
55.52
52.56
29.85
29.70
29.55
29.39
-17.15
BF3K
O
MeO
13C {1H} NMR, 126 MHz, d6-acetone
-138.79
-134.13
129.99
129.83
-129.01
-127.13
126.77
-52.56
140
136
132
128
f1 (ppm)
55
54
53
52
51
f1 (ppm)
60
240
220
200
180
160
140
120
100
80
60
40
20
0
f1 (ppm)

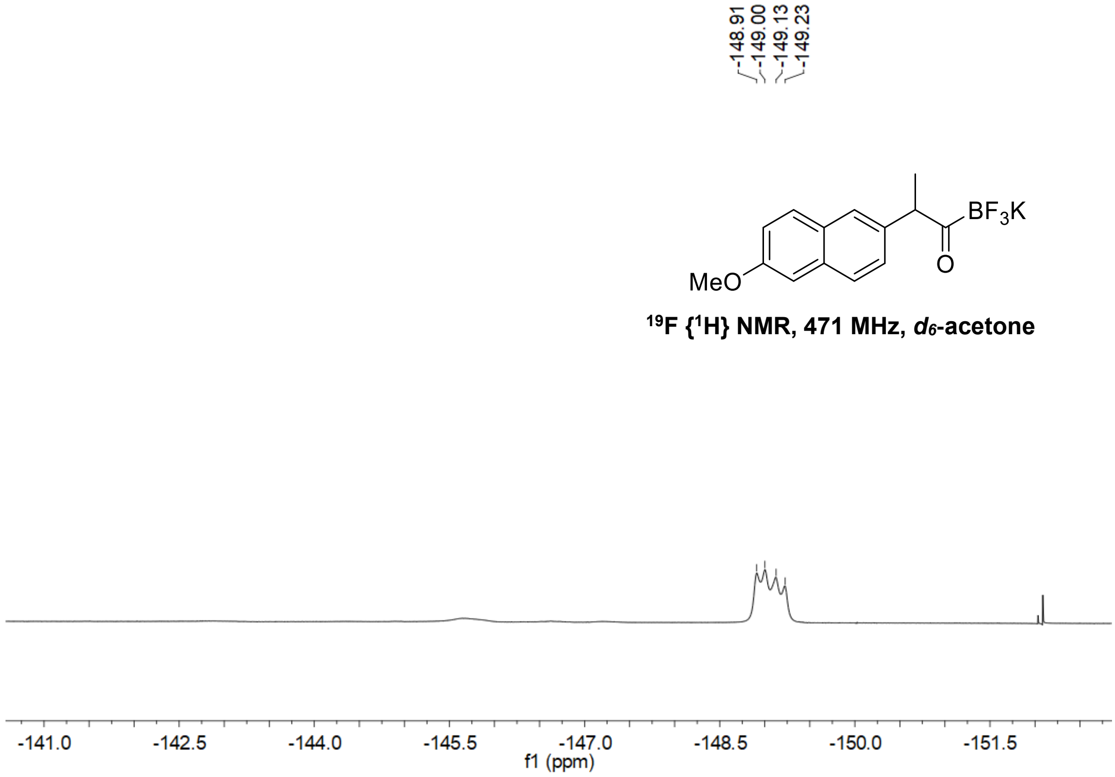
-148.91
-149.00
-149.13
-149.23
BF3K
O
MeO
19F {1H} NMR, 471 MHz, d6-acetone
-141.0
-142.5
-144.0
-145.5
-147.0
-148.5
-150.0
-151.5
f1 (ppm)

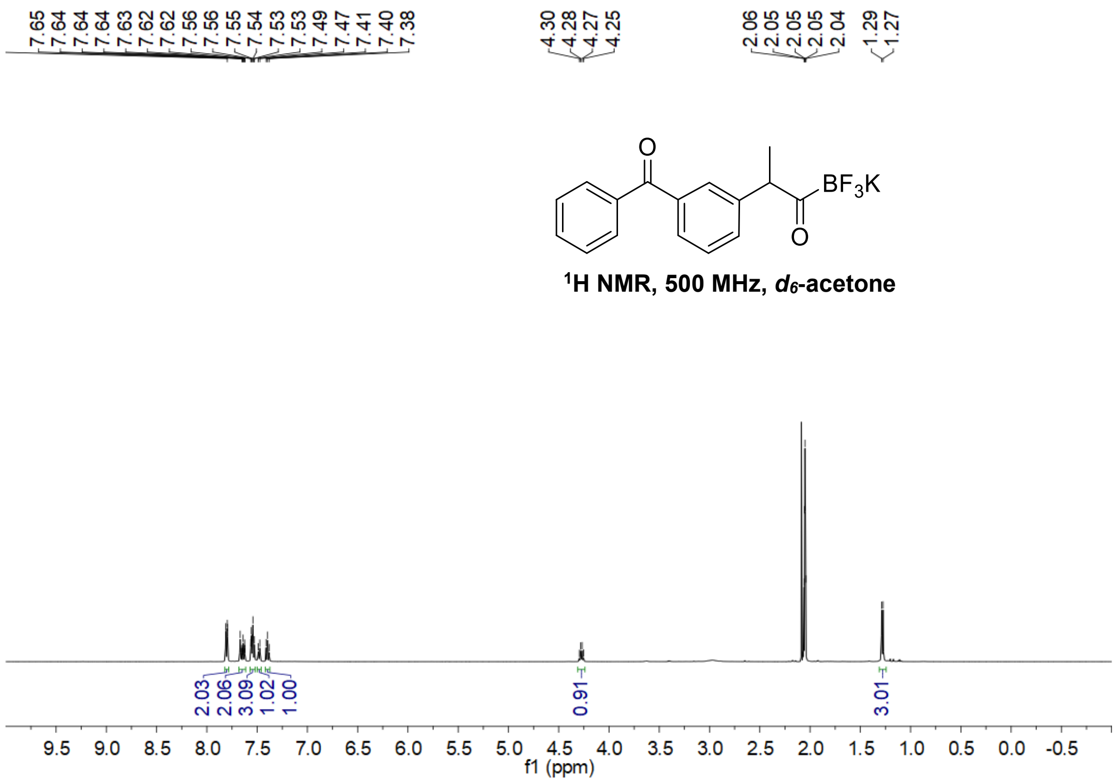
7.65 7.64 7.64 7.64 7.63 7.62 7.62 7.56 7.56 7.55 7.54 7.53 7.53 7.49 7.47 7.41 7.40 7.38
4.30 4.28 4.27 4.25
2.06 2.05 2.05 2.05 2.04 1.29 1.27
O
BF3K
O
1H NMR, 500 MHz, d6-acetone
2.03 2.06 3.09 1.02 1.00
0.91
3.01
9.5 9.0 8.5 8.0 7.5 7.0 6.5 6.0 5.5 5.0 4.5 4.0 3.5 3.0 2.5 2.0 1.5 1.0 0.5 0.0 -0.5
f1 (ppm)

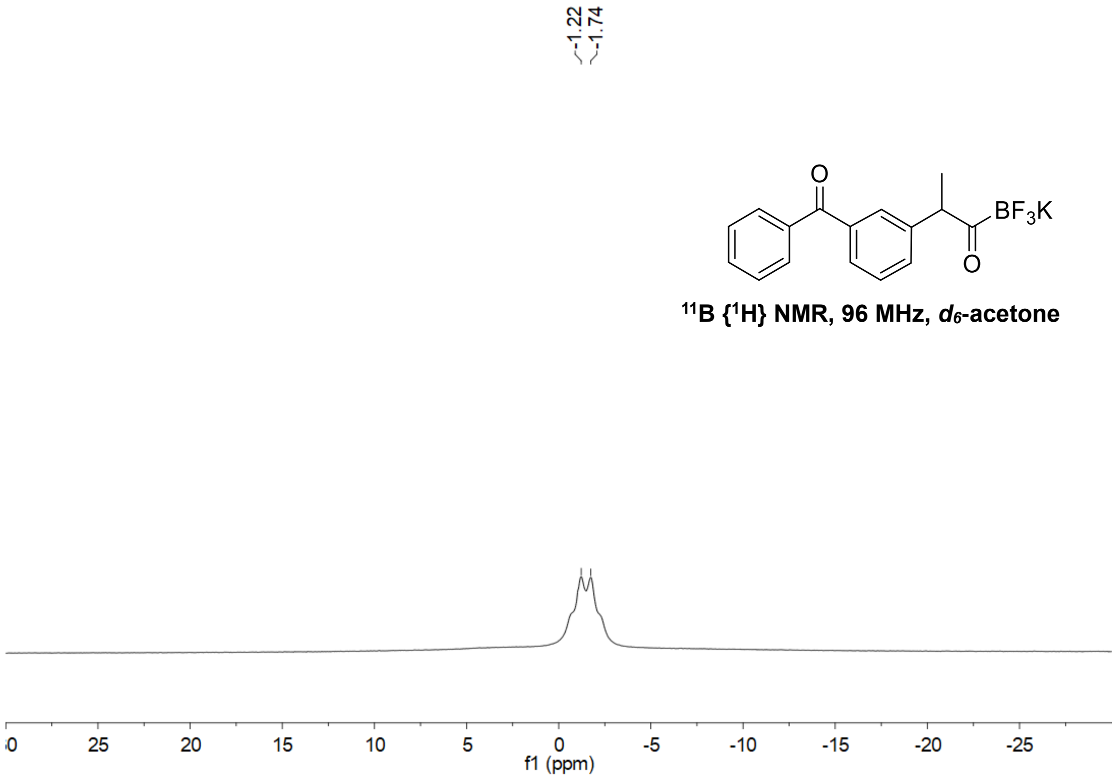
-1.22 -1.74
O
BF3K
O
11B {1H} NMR, 96 MHz, d6-acetone
25 20 15 10 5 0 -5 -10 -15 -20 -25
f1 (ppm)

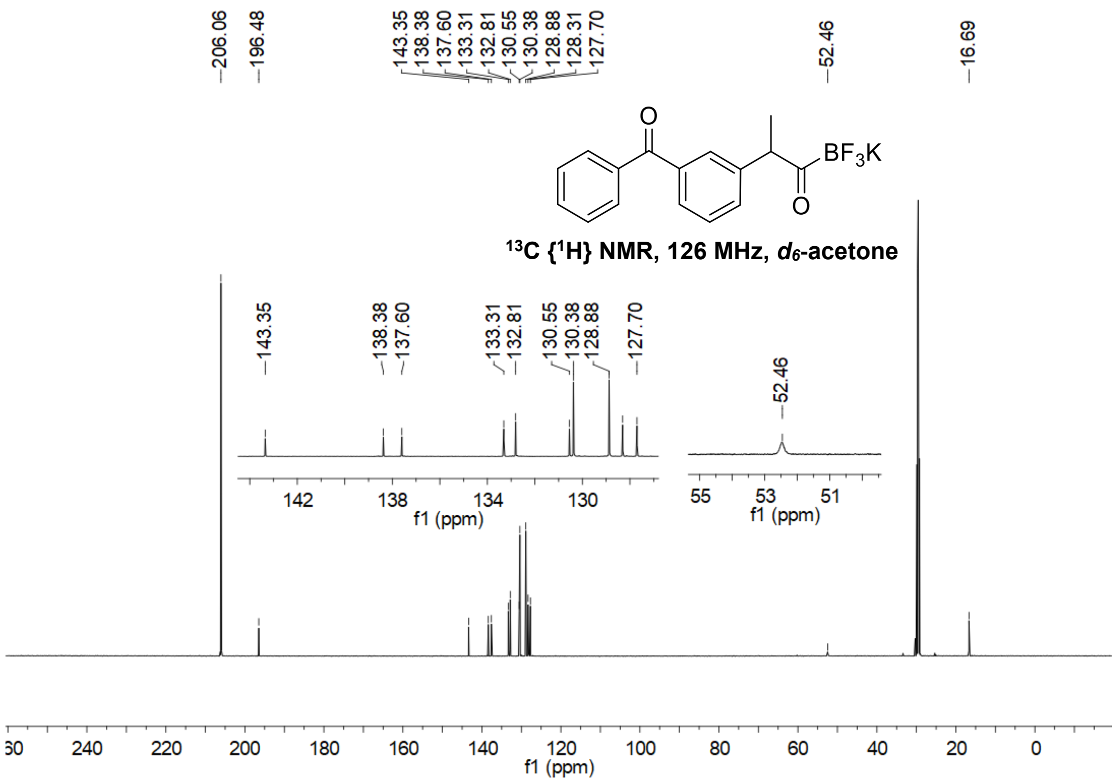
206.06
196.48
143.35
138.38
137.60
133.31
132.81
130.55
130.38
128.88
128.31
127.70
52.46
16.69
BF3K
O
13C {1H} NMR, 126 MHz, d6-acetone
f1 (ppm)

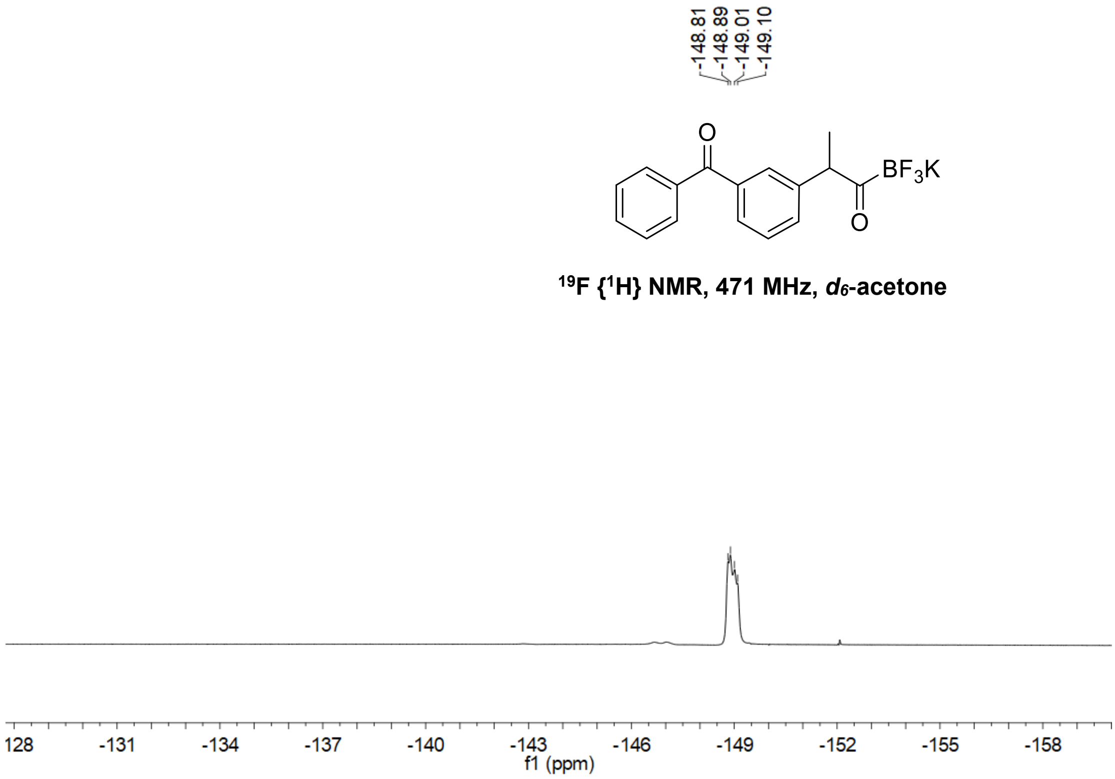
-148.81
-148.89
-149.01
-149.10
BF3K
O
19F {1H} NMR, 471 MHz, d6-acetone
f1 (ppm)

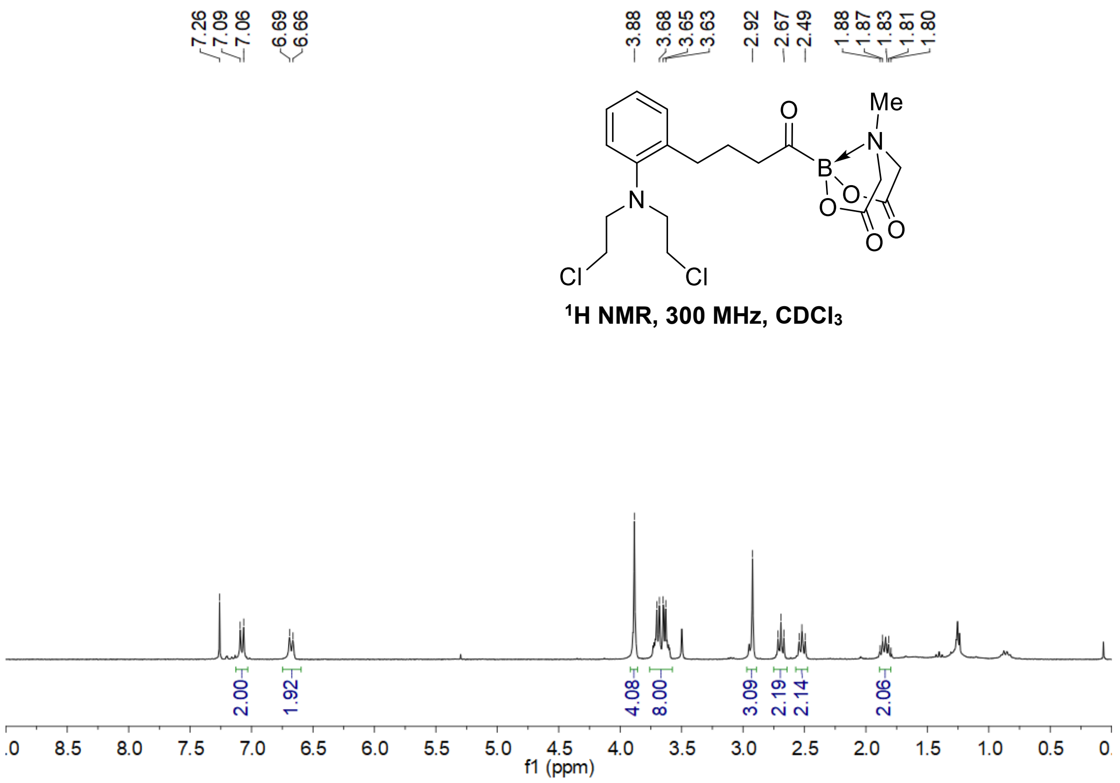
7.26
7.09
7.06
6.69
6.66
3.88
3.68
3.65
3.63
2.92
2.67
2.49
1.88
1.87
1.83
1.81
1.80
Me
O
N
B
O
O
O
O
N
Cl
Cl
^{1}H NMR, 300 MHz, CDCl$_3$
2.00
1.92
4.08
8.00
3.09
2.19
2.14
2.06
f1 (ppm)

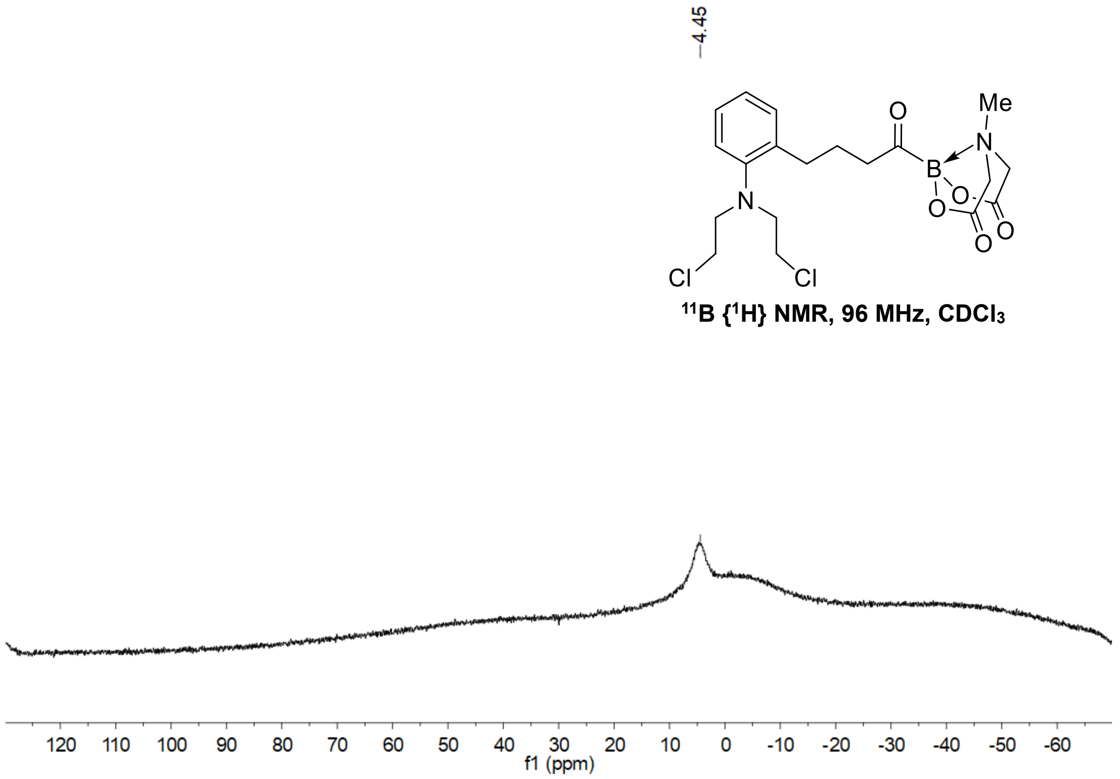
4.45
Me
O
N
B
O
O
O
O
N
Cl
Cl
^{11}B {^{1}H} NMR, 96 MHz, CDCl$_3$
f1 (ppm)

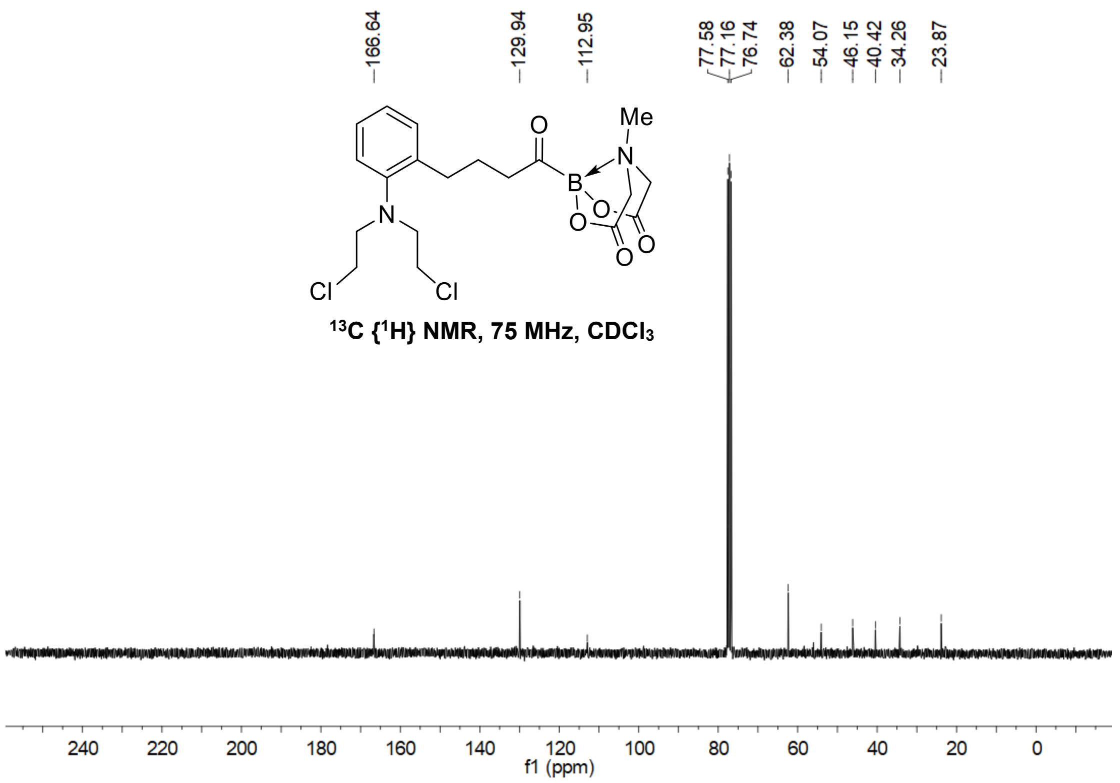
166.64
129.94
112.95
77.58
77.16
76.74
62.38
54.07
46.15
40.42
34.26
23.87
Me
N
B
O
O
O
O
N
Cl
Cl
^{13}C {^{1}H} NMR, 75 MHz, $CDCl_3$
f1 (ppm)

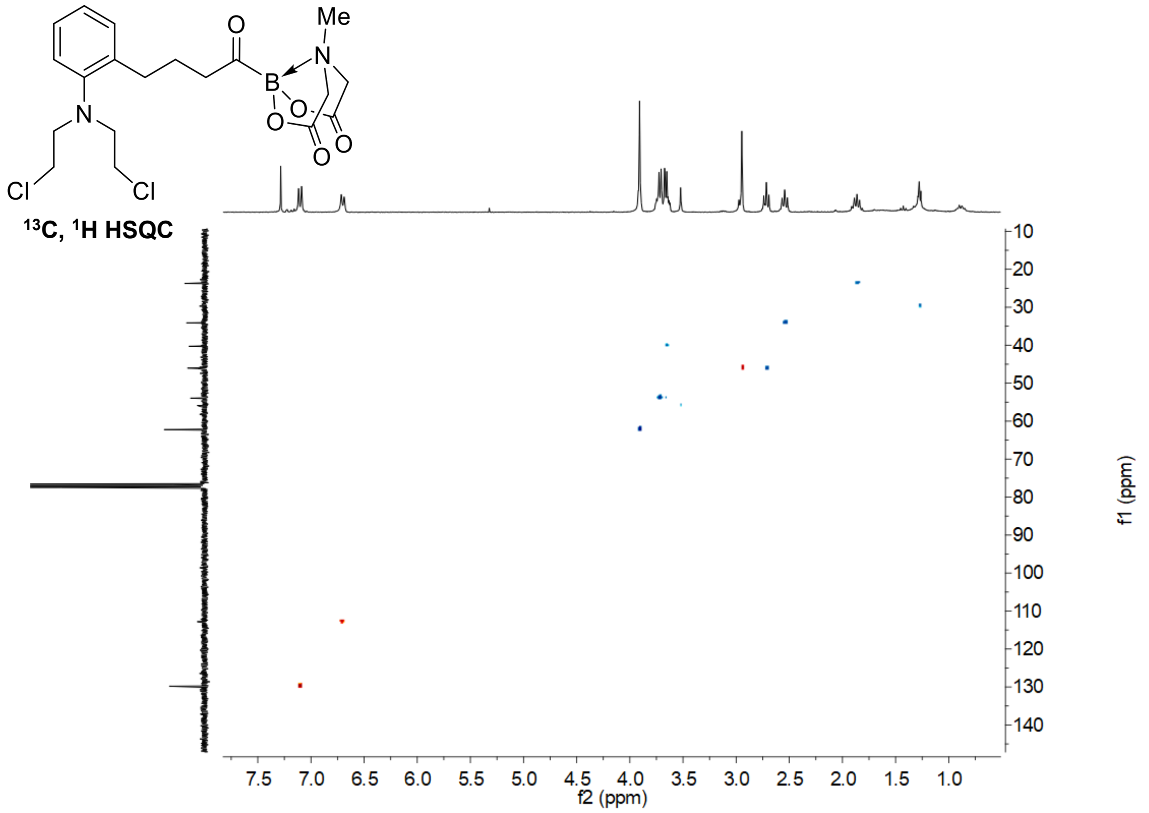
Me
N
B
O
O
O
O
N
Cl
Cl
^{13}C, ^{1}H HSQC
f2 (ppm)
f1 (ppm)

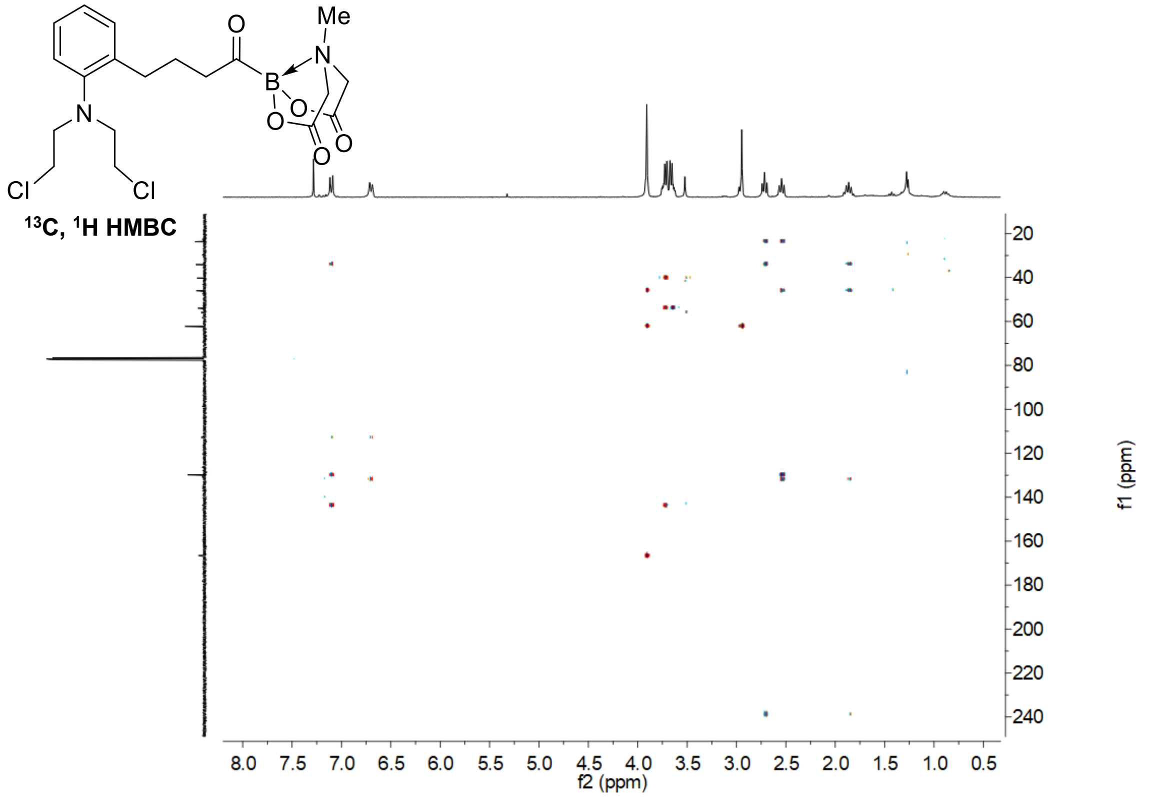

Me
O
N
B
O
Cl
Cl
13C, 1H HMBC
f2 (ppm)
f1 (ppm)

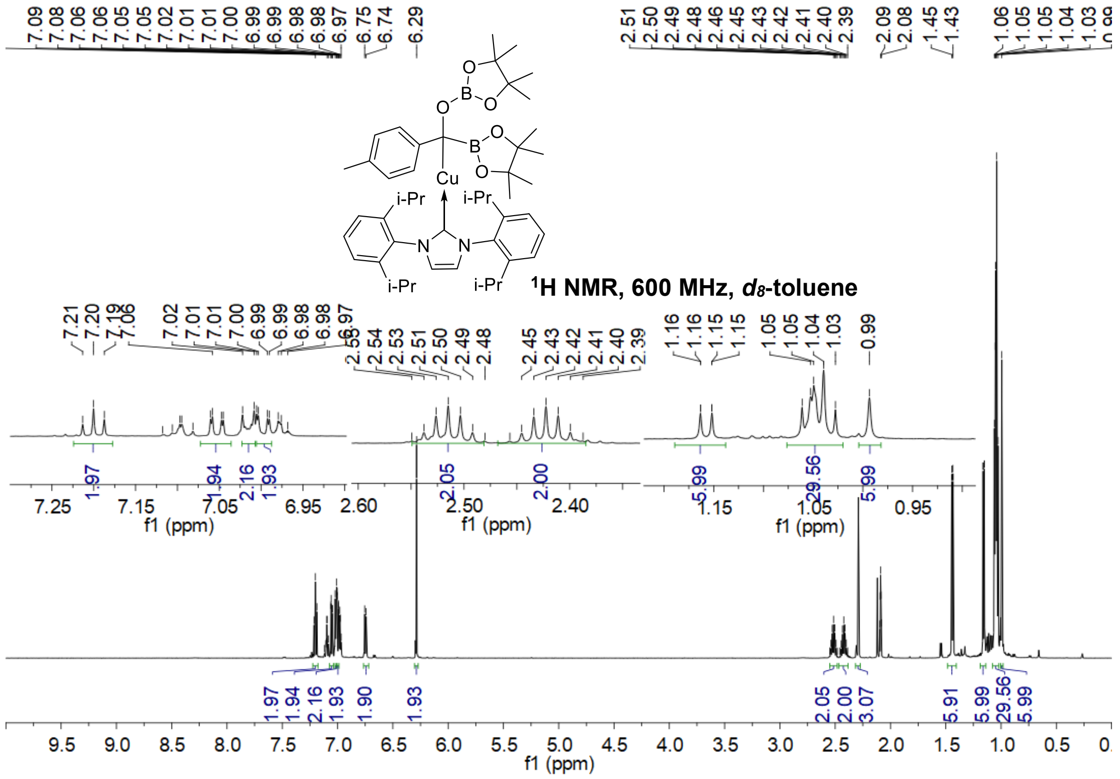

Cu
i-Pr
N
1H NMR, 600 MHz, d8-toluene
f1 (ppm)

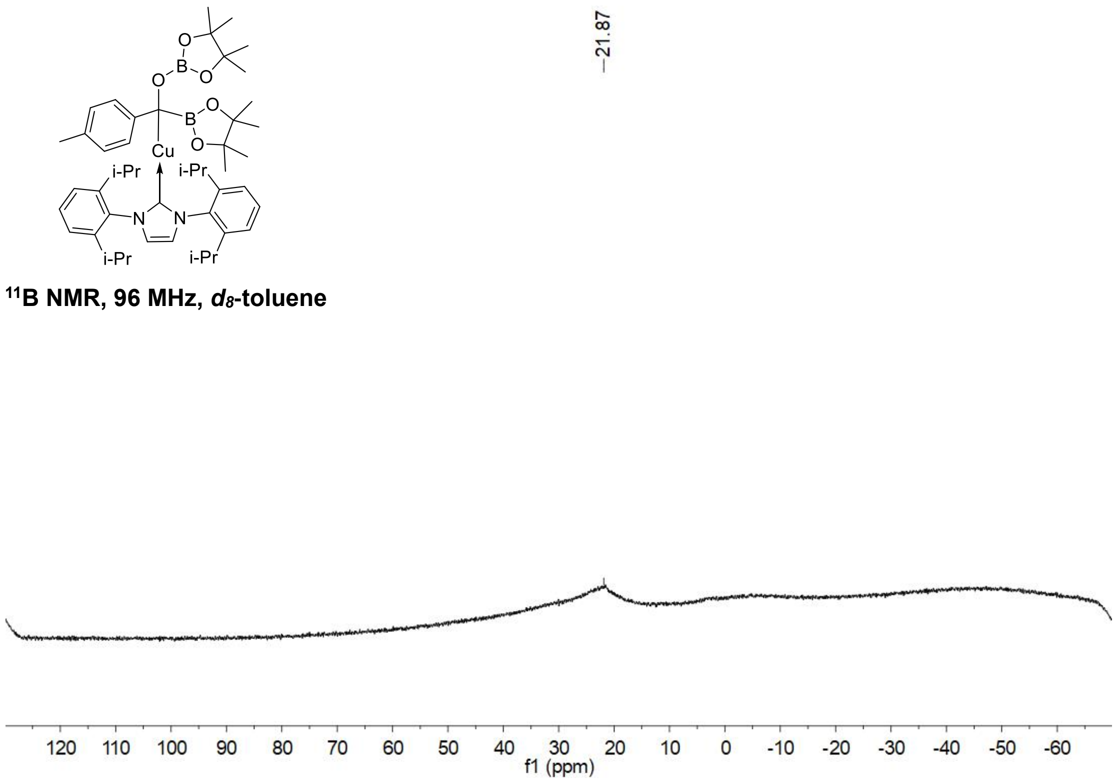

^{11}B NMR, 96 MHz, *d₈*-toluene

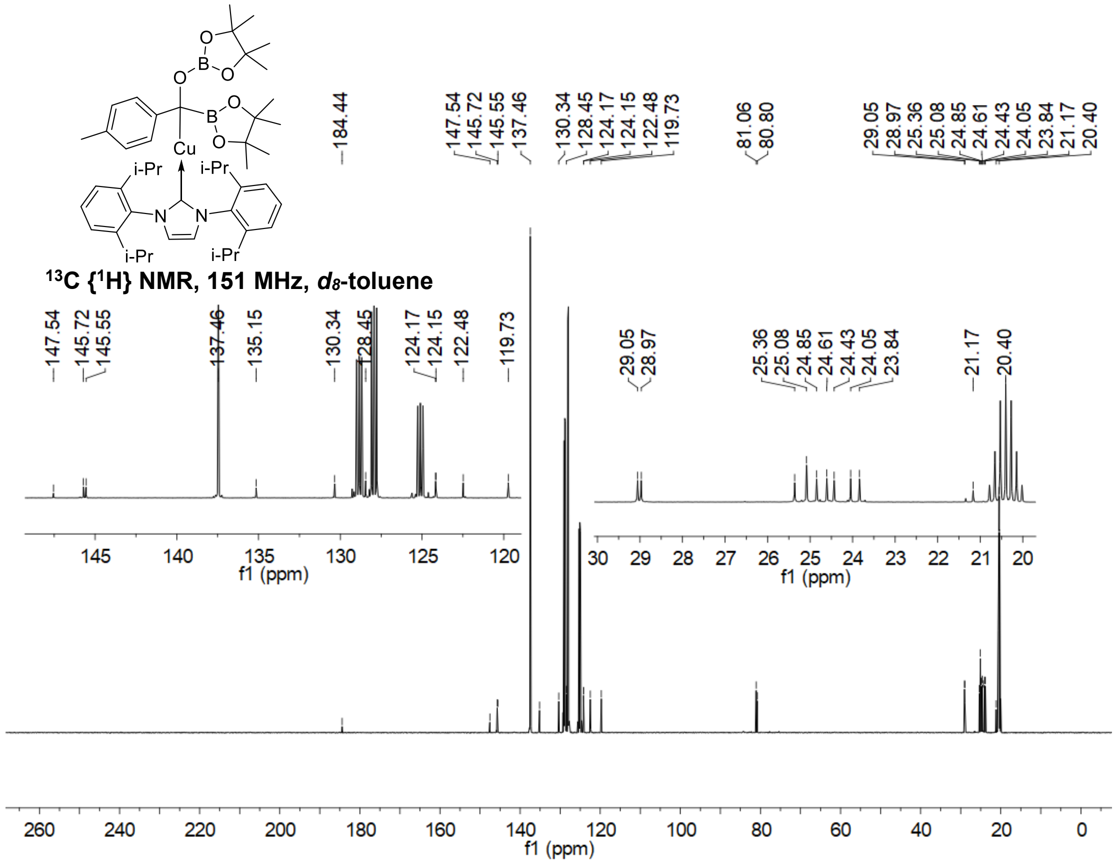

^{13}C {^{1}H} NMR, 151 MHz, *d₈*-toluene

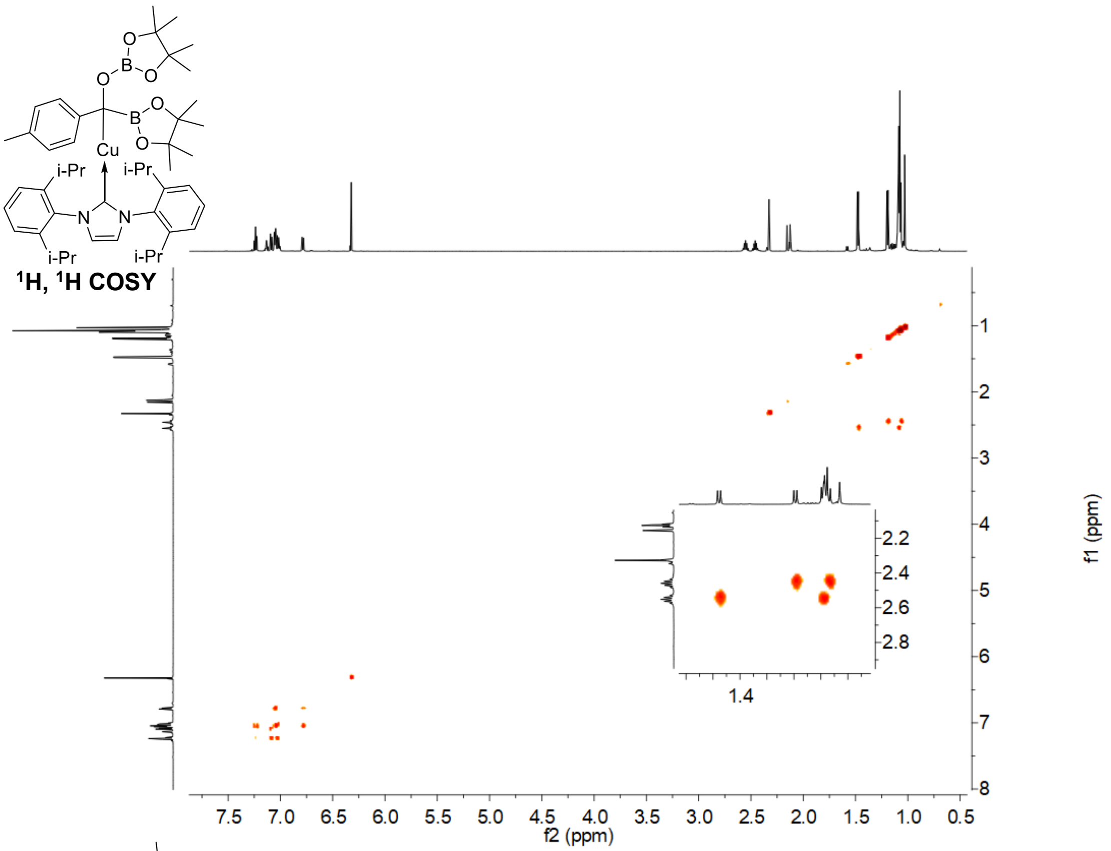

^1H, ^1H COSY

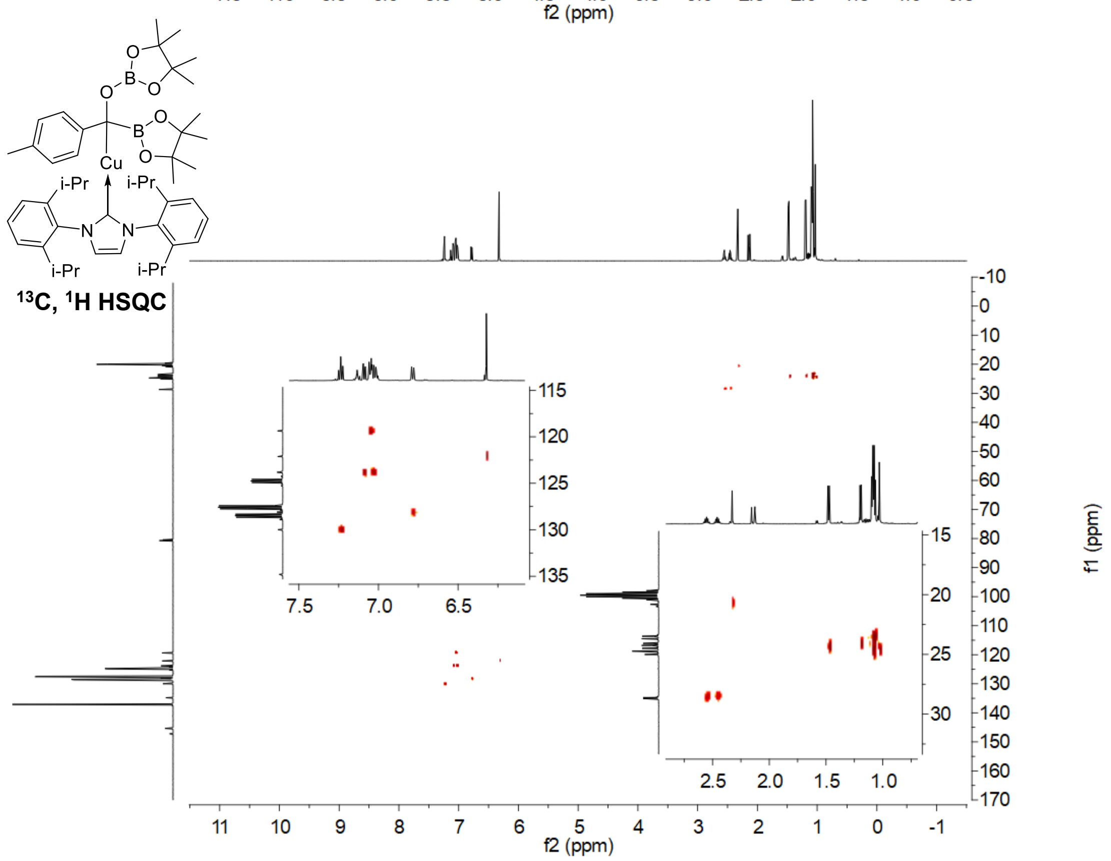

^{13}C, ^1H HSQC

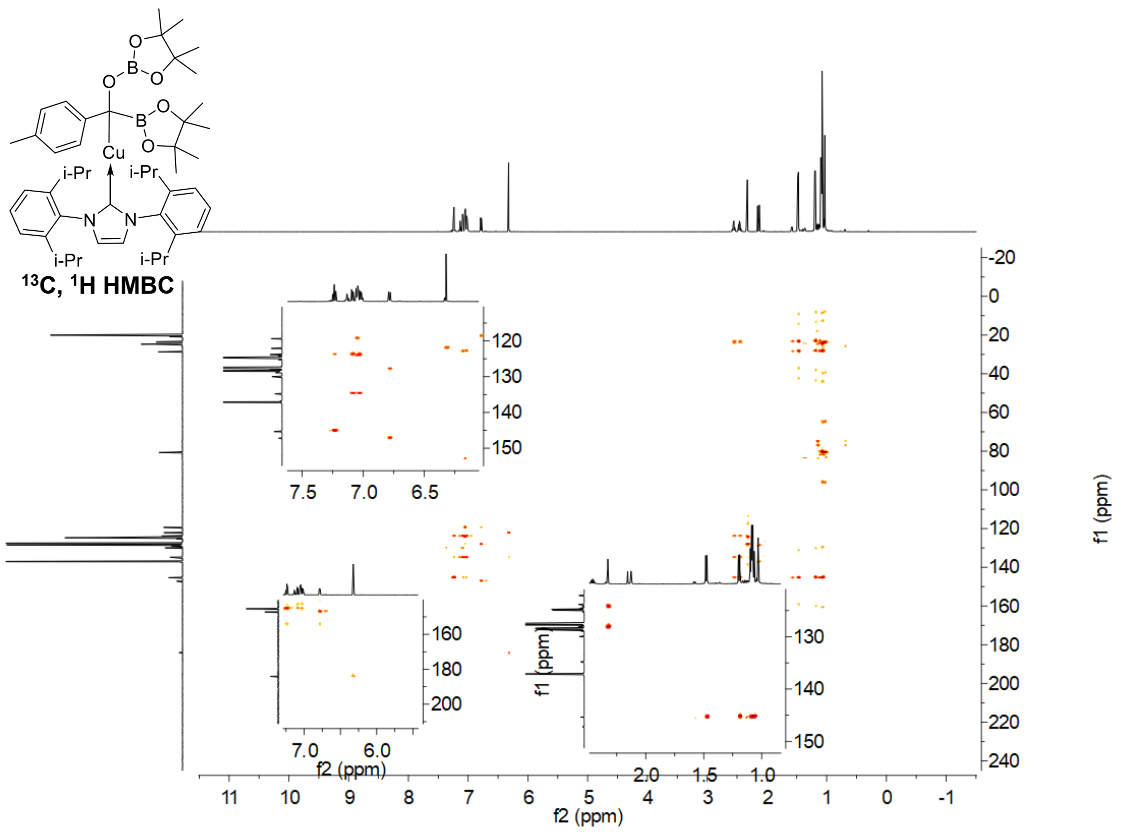

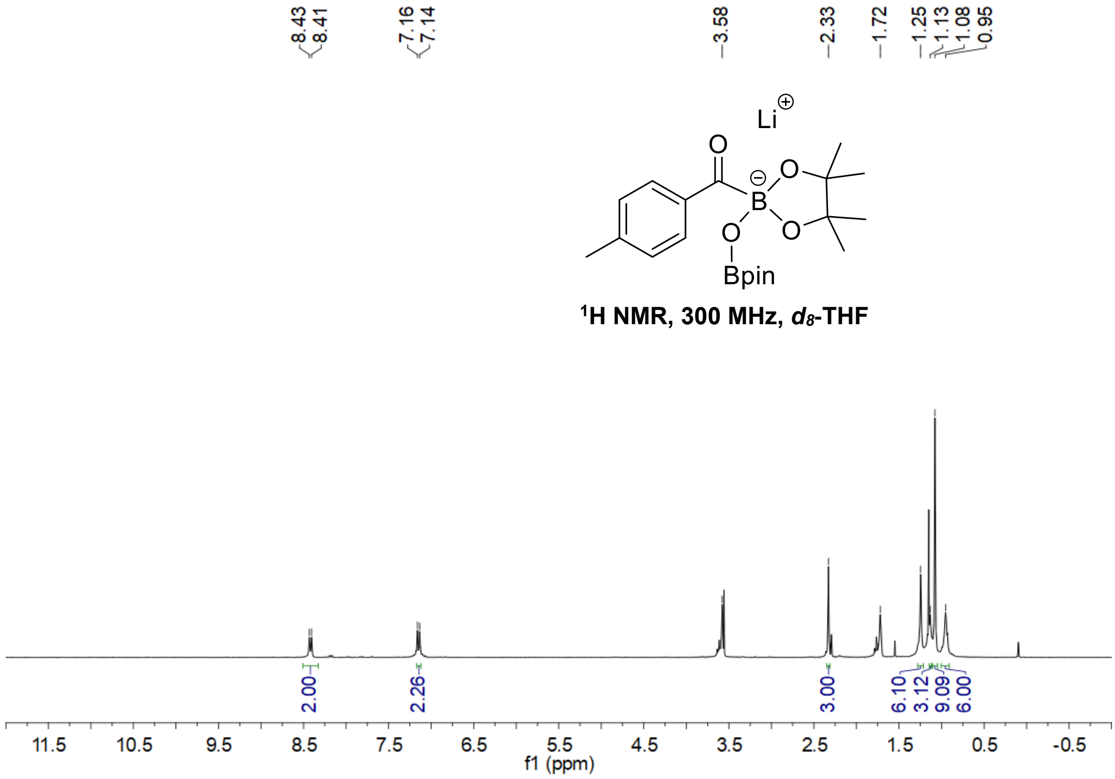

^1H NMR, 300 MHz, d_8-THF

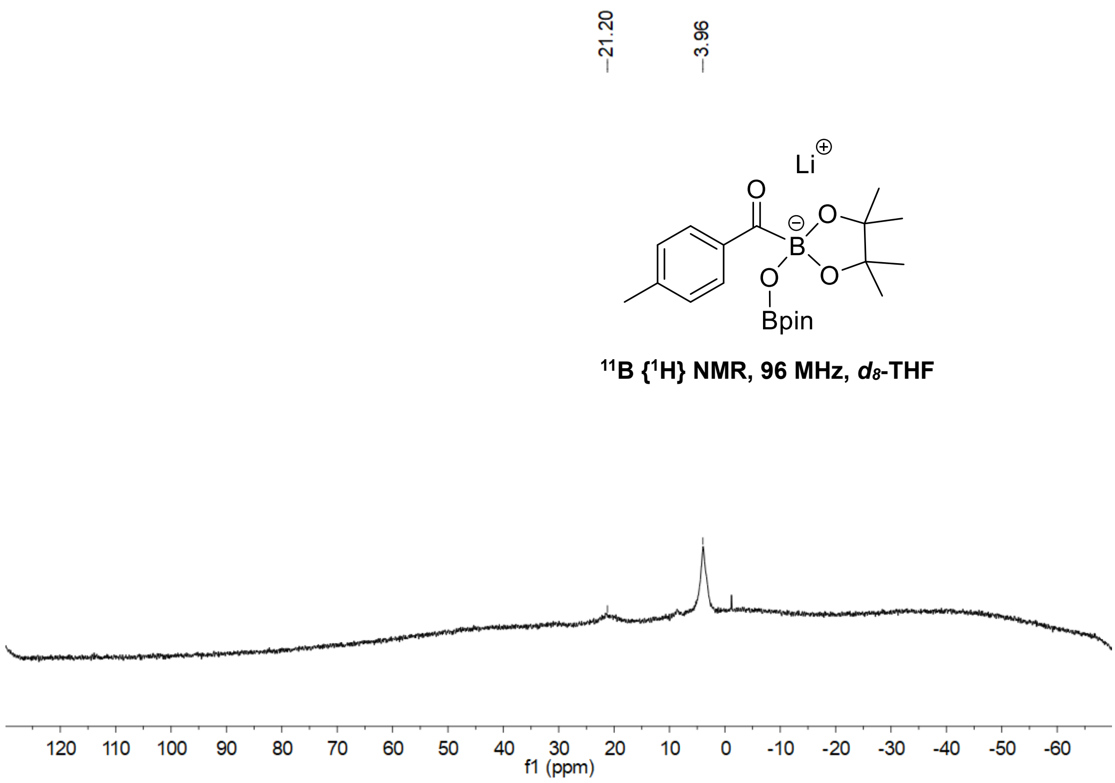
21.20
3.96
Li⊕
O
B
O
O
O
Bpin
^{11}B {^{1}H} NMR, 96 MHz, *d8*-THF
120 110 100 90 80 70 60 50 40 30 20 10 0 -10 -20 -30 -40 -50 -60
f1 (ppm)

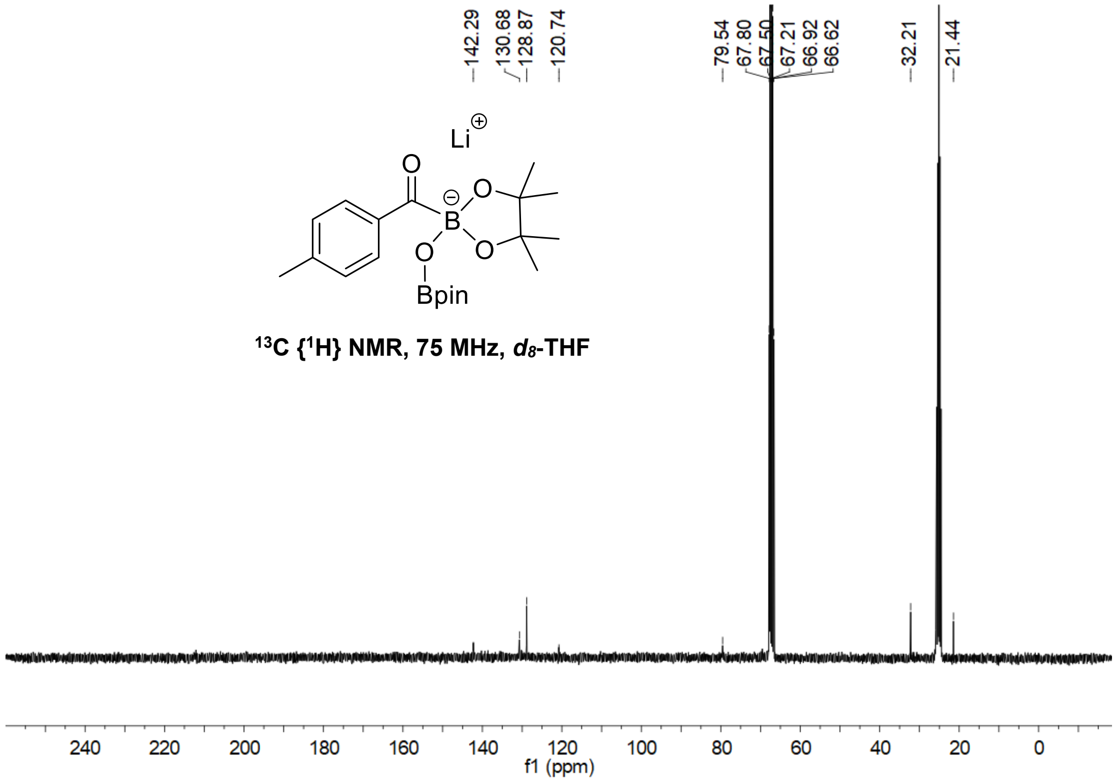
142.29
130.68
128.87
120.74
79.54
67.80
67.50
67.21
66.92
66.62
32.21
21.44
Li⊕
O
B
O
O
O
Bpin
^{13}C {^{1}H} NMR, 75 MHz, *d8*-THF
240 220 200 180 160 140 120 100 80 60 40 20 0
f1 (ppm)
